# Asymmetric syntheses of 8-oxabicyclo[3,2,1]octane and 11-oxatricyclo[5.3.1.0]undecane from glycals†
†Electronic supplementary information (ESI) available: Experimental procedures and characterisation data for all compounds are provided. CCDC 1436794 and 1520731. For ESI and crystallographic data in CIF or other electronic format see DOI: 10.1039/c7sc02625k



**DOI:** 10.1039/c7sc02625k

**Published:** 2017-08-07

**Authors:** Hongze Liao, Wei-Lin Leng, Kim Le Mai Hoang, Hui Yao, Jingxi He, Amanda Ying Hui Voo, Xue-Wei Liu

**Affiliations:** a Division of Chemistry and Biological Chemistry , School of Physical and Mathematical Sciences , Nanyang Technological University , 21 Nanyang Link , Singapore 637371 . Email: xuewei@ntu.edu.sg

## Abstract

Herein, we describe an efficient method to prepare enantiomerically pure 8-oxabicyclo[3.2.1]octanes *via* gold(i)-catalyzed tandem 1,3-acyloxy migration/Ferrier rearrangement of glycal derived 1,6-enyne bearing propargylic carboxylates. The resultant compounds could then undergo interrupted Nazarov cyclization to afford diastereomerically pure 11-oxatricyclo[5.3.1.0]undecanes.

## Abstract 2

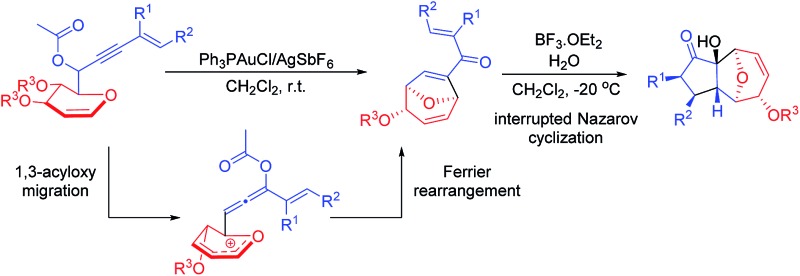
A gold-catalyzed tandem 1,3-acyloxy migration/Ferrier rearrangement to access 8-oxabicyclo[3.2.1]octanes with high efficiency and complete diastereoselectivity was developed successfully. Resultant compounds could undergo the interrupted Nazarov cyclization to afford diastereomerically pure 11-oxatricyclo[5.3.1.0]undecanes.

## 

Chiral 8-oxabicyclo[3.2.1]octane and 11-oxatricyclo[5.3.1.0]-undecane are common structural motifs featured in many classes of natural products (Fig. 1), some of which show interesting biological activities. Englerin A, a potential anti-tumor reagent, is isolated from *Phyllanthus engleri* in Tanzania and shows selective activity to renal cancer cell lines at the nanomolar level.^1^ Balsamiferine J, isolated from *Blumea balsamifera*, represents a novel type of sesquiterpenoids with NO inhibitory activity against murine microglial cell lines.^2^ Homalomenol C, isolated from the roots of *Homalomena aromatica*, is reported as the bioactive component of the Vietnamese traditional medicine as an anti-inflammatory agent.^3^

**Fig. 1:**
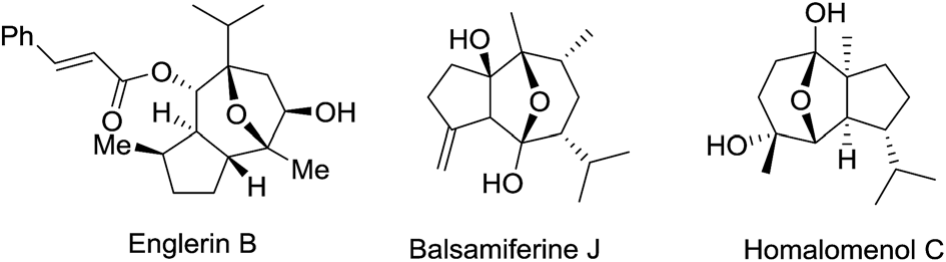
Selected natural products containing 8-oxabicyclo[3.2.1]octane core structures.

Driven by their important bioactivities, many chemists are interested in the synthesis of this type of natural products. Attempts to gain access to this unique 8-oxabicyclo[3.2.1]-octane ring asymmetrically include [4 + 3],^4^ [5 + 2],^5^ [3 + 2]^6^ cycloadditions and cascade reactions.^7^ Despite achieving moderate to good diastereoselectivities, these methods suffer from the drawbacks of requiring expensive chiral metal catalysts, requiring installation of non-atom economical auxiliaries and tedious chiral substrate synthesis. However, to our knowledge, there is still no report on the asymmetric synthesis of 11-oxatricyclo[5.3.1.0]undecane.

Tandem and sequential reactions are useful synthetic approaches as they offer the advantages of efficiency as well as reduction of cost and waste.^8^ Gold-catalyzed transformations of 1,*n*-enyne bearing propargylic carboxylates to form complex molecules have progressed rapidly in the past decade and an impressive series of transformations have been reported.^9^ It is well established that the propargylic esters could undergo 1,2- or 1,3-acyloxy migration to give the corresponding gold vinyl carbenoid or allene intermediates^10^ and these two intermediates could initiate further transformations depending on the reaction conditions and properties of substrates.^11^ Hence, we envisaged that the glycal linked 1,6-enyne bearing propargylic carboxylates **1** could undergo the 1,3-acyloxy migration and the resulting intermediate would be prone to subsequent Ferrier rearrangement, thus furnishing the enantiomerically pure disubstituted 8-oxabicyclo[3.2.1]octanes **2**. If successful, this tandem 1,3-acyloxy migration/Ferrier rearrangement would set the stage for a subsequent Nazarov cyclization^12^ to form the 11-oxatricyclo[5.3.1.0]undecane derivatives **3** (Scheme 1). To the best of our knowledge, this mode of reactivity is unprecedented in reactions involving 1,*n*-enyne bearing propargylic carboxylates. Furthermore, this glycal derived 1,6-enyne could be synthesized from readily available glycals through simple steps, making this chirality source cheap and atom economical.

**Scheme 1:**
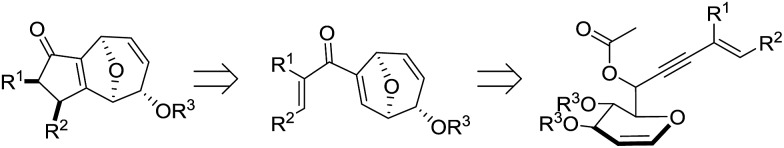
1,3-Acyloxy migration/Ferrier rearrangement and Nazarov cyclization sequence.

d-Glucal derived 1,6-enyne bearing propargylic carboxylates **1a** was selected as the starting material to commence the investigation of this gold-catalyzed tandem reaction.^13^ To our delight, the reaction could go smoothly with compound **1a** as the starting material in the presence of AuCl_3_ to give the desired product **2a** in 69% yield within 5 min (Table 1, entry 1). Other commercially available gold catalysts could also promote this reaction, further proving the feasibility of our strategy (Table 1, entries 2 and 3). Activated Ph_3_PAuSbF_6_ generated *in situ* from Ph_3_PAuCl/AgSbF_6_ was found to be most suitable for this reaction (Table 1, entry 4). Subsequently, the counter-ion effect was investigated through examining various silver salts and the AgSbF_6_ presented the best performance (Table 1, entries 4–6). Attempt to employ the Brønsted acid catalyst, *p*-TsOH resulted in decomposition of the starting material (Table 1, entry 7). Notably, Ph_3_PAuCl and AgSbF_6_ were found to be inactive when they were employed individually (Table 1, entries 8 and 9). Solvent screening showed that CH_2_Cl_2_ was superior to other commonly used solvents (Table 1, entries 10–13). To demonstrate the utility of this method, a reaction on a larger scale (0.5 mmol) was carried out and **2a** was afforded in 81% yield (Table 1, entry 14). Interestingly, when the propargylic ester group was switched from acetyl group to pivaloyl or benzoyl groups, no significant change was observed (Table 1, entries 15 and 16).

**Table 1:** Optimization of gold-catalyzed reaction of **1a**

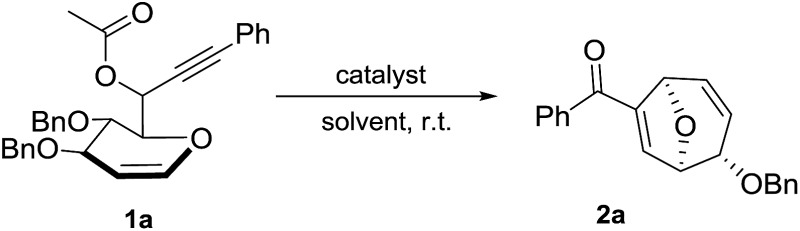				
Entry^*a*^	Catalyst	Solvent	Time	Yield^*b*^
1	AuCl_3_	CH_2_Cl_2_	5 min	69%
2	AuCl	CH_2_Cl_2_	5 min	42%
3	Ph_3_PAuNTf_2_	CH_2_Cl_2_	5 min	72%
4	Ph_3_PAuCl/AgSbF_6_	CH_2_Cl_2_	5 min	80%
5	Ph_3_PAuCl/AgClO_4_	CH_2_Cl_2_	5 min	62%
6	Ph_3_PAuCl/AgOTf	CH_2_Cl_2_	5 min	68%
7	*p*-TsOH	CH_2_Cl_2_	—	Trace
8	Ph_3_PAuCl	CH_2_Cl_2_	—	n.r.
9	AgSbF_6_	CH_2_Cl_2_	—	n.r.
10	Ph_3_PAuCl/AgSbF_6_	DCE	5 min	76%
11	Ph_3_PAuCl/AgSbF_6_	Toluene	2 h	52%
12	Ph_3_PAuCl/AgSbF_6_	CH_3_NO_2_	5 min	66%
13	Ph_3_PAuCl/AgSbF_6_	CH_3_CN	1 h	48%
14^*c*^	Ph_3_PAuCl/AgSbF_6_	CH_2_Cl_2_	5 min	81%
15^*d*^	Ph_3_PAuCl/AgSbF_6_	CH_2_Cl_2_	5 min	79%
16^*e*^	Ph_3_PAuCl/AgSbF_6_	CH_2_Cl_2_	5 min	72%

^*a*^Reaction conditions: propargylic ester **1** (0.1 M in CH_2_Cl_2_), 5 mol% gold catalyst, 10 mol% silver catalyst.

^*b*^Isolated yield.

^*c*^Reaction was carried out on scale of 0.5 M of **1a**.

^*d*^Pivaloyl ester substrate.

^*e*^Benzoyl ester substrate. DCE = ClCH_2_CH_2_Cl, n.r. = no reaction.

Using the optimal conditions, a range of the glycal derived 1,6-enyne bearing propargylic carboxylates were investigated to demonstrate the wide application of this transformation. In general, the desired products were rapidly obtained in good to excellent yields. The stereochemistry was unambiguously confirmed by X-ray analysis of compound **2b**.^14^ A comparison of the *para*-substituents of aryl substrates showed that those with electron donating aryl substituents (**2b**, **c**) afforded higher yields than those with electron withdrawing substituents (**2d**, **e**). Replacing the aryl substituent at the alkyne position with less bulky alkyl substituents, such as cyclohexyl, *n*-butyl and methyl substituent (**2f–h**) furnished the corresponding products in good yields. Alkene substituents were also tolerated (**2i–m**), but the yield decreased when the reaction was carried out on a larger scale (0.5 M). It should be noted that the reaction proceeded readily to give the rearranged products when the benzyl group was changed to methyl or methoxymethyl groups (**2n–r**). To the best of our knowledge, methoxy group has never been used as the leaving group for Ferrier rearrangement. When l-glycal derived propargylic acetates were tested for this reaction, similar results were obtained (*ent*-**2a**, **2b** and **2g**). As expected, d-galactal derived substrate led to a diminished yield due to the steric effect between benzyl group and propargylic ester (*epi*-**2a**). Notably, complete diastereoselectivity was detected in all the examples (Table 2).

**Table 2:** Substrate scope of gold-catalyzed synthesis of **2**

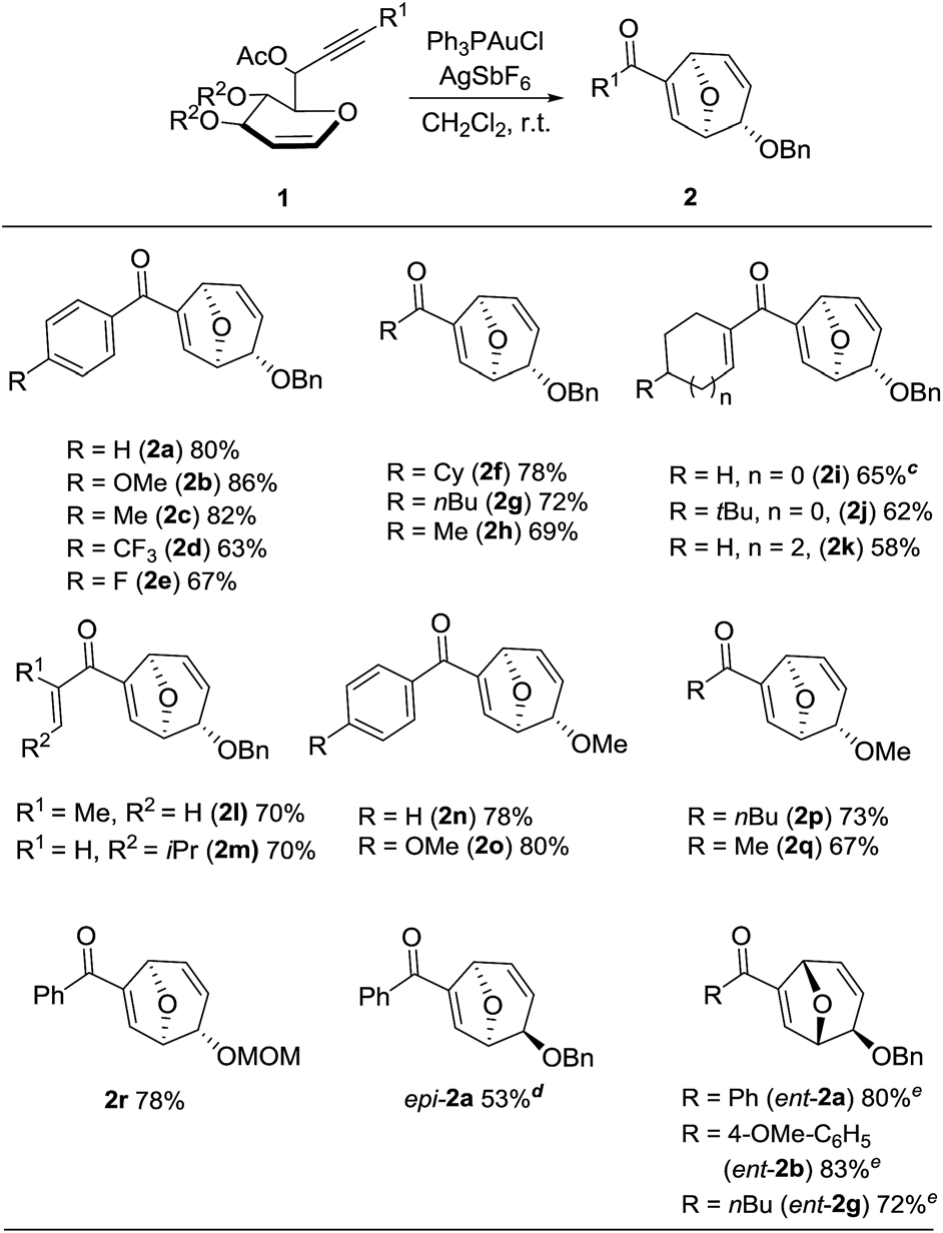

^*a*^Reaction conditions: propargylic ester **1** (0.1 M in CH_2_Cl_2_), 5 mol% PPh_3_AuCl, 10 mol% AgSbF_6_.

^*b*^Isolated yield.

^*c*^53% yield based on scale of 0.5 M of **1i**.

^*d*^ d-Galactal derived substrate.

^*e*^ l-Glucal derived substrate.

Based on the experimental results, a plausible mechanism for the formation of disubstituted 8-oxabicyclo[3.2.1]octane product is proposed with compound **1a** (Scheme 2). The gold catalyst is proposed to have dual role in this transformation. Firstly, it helps to transform the propargylic ester motif of compound **1a** into the nucleophilic allenic intermediate **C** through gold-catalyzed 1,3-acyloxy migration. Secondly, it serves as Lewis acid to facilitate the intramolecular Ferrier reaction by promoting the departure of benzyloxy group and leading to the formation of allylic oxocarbenium ion **E**.^15^ Subsequently, electrophilic attack of the allylic oxocarbenium motif to the allene generated oxa-bridged 7-membered ring intermediate **F**. Finally, the leaving group of Ferrier rearrangement attacks the oxonium species to generate the desired disubstituted 8-oxabicyclo[3.2.1]octane product **2a** with benzyl acetate as the byproduct. The complete diastereoselectivity is attributed to *cis* face attack of allenic ester at C-5 position of glycal derived propargylic esters.

**Scheme 2:**
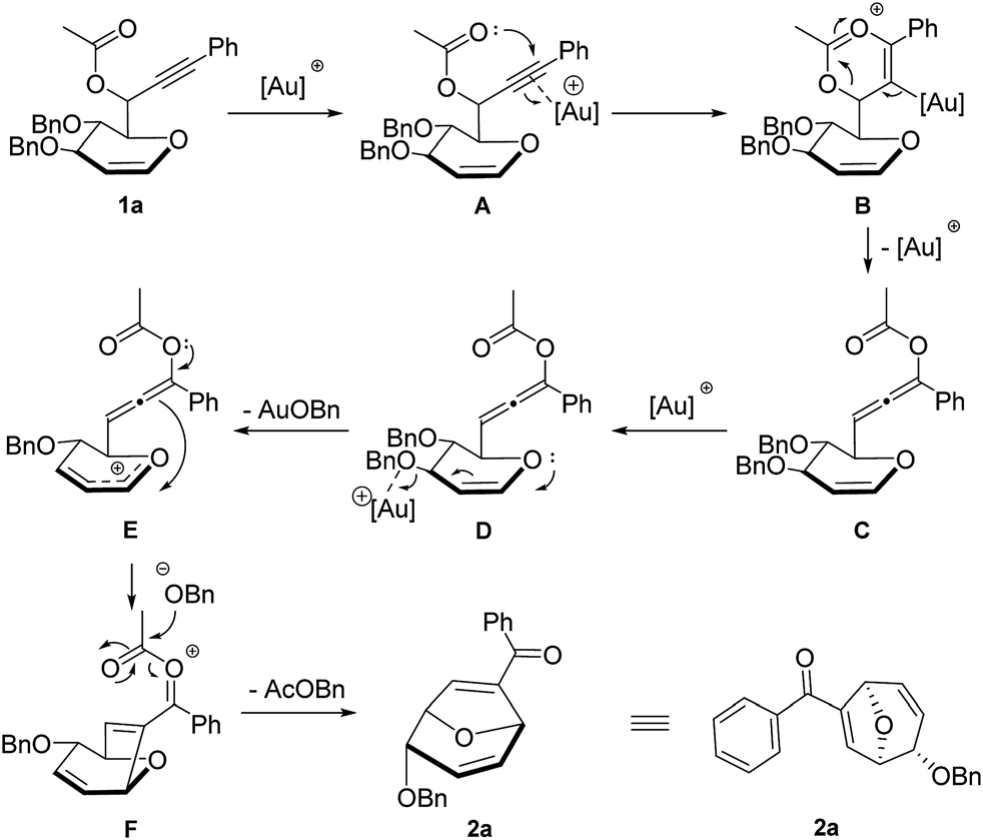
Plausible mechanism for the formation of **2a**.

To confirm the mechanism, isotopic labeling experiments were conducted (Scheme 3). When the propargylic acetate **1a-^18^O** with an ^18^O-enriched carbonyl oxygen atom was synthesized and subjected to the optimized reaction conditions, ^18^O-labeled 8-oxabicyclo[3.2.1]octane **2a-^18^O** was isolated in 78% yield and ^18^O containing carbonyl fragment was detected (PhC^18^O^+^ with *m*/*z* 107.0393). Conversely, ^18^O label was present in benzyl benzoate **6-^18^O** instead of 8-oxabicyclo[3.2.1]octane **2a-^18^O** when propargylic benzoate **1s-^18^O** with an ^18^O-enriched ester oxygen was used as the starting material. The following conclusions could be drawn from the isotopic labeling results detected by high-resolution mass spectrometry: firstly, 8-oxabicyclo[3.2.1]octane was generated exclusively from allene intermediate *via* gold-catalyzed 1,3-acyloxy migration rather than two sequential 1,2-acyloxy migration since scrambling of ^18^O-label was not detected in **5** or **2a**. Secondly, Ferrier rearrangement was initiated by gold-catalyzed 1,3-acyloxy migration and its byproduct benzyl oxoanion quenched the reaction, accounting for the high efficiency and rapid rate for this transformation.

**Scheme 3:**
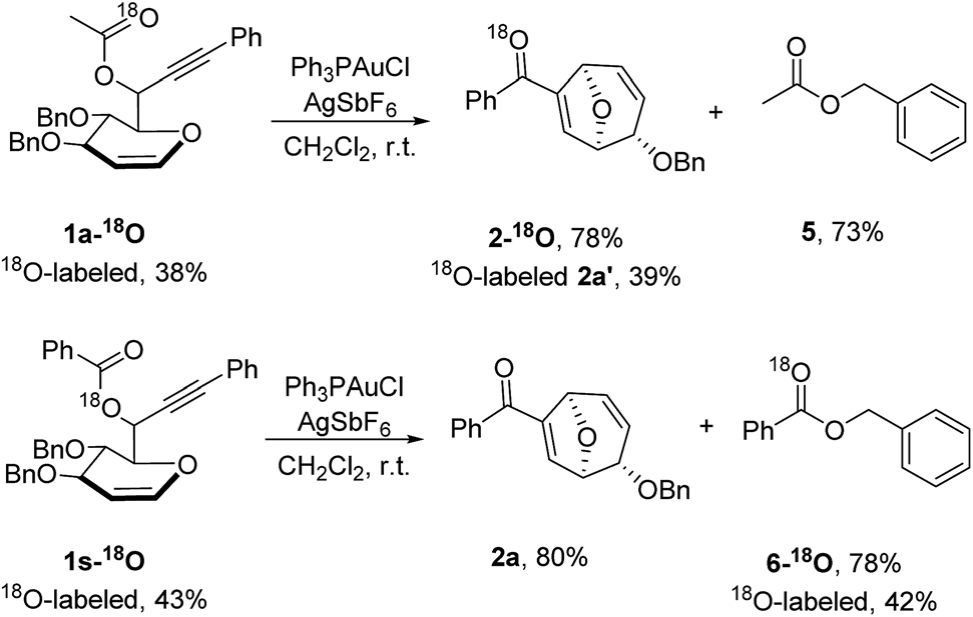
Isotope labeling experiments.

In order to investigate whether Nazarov cyclization could proceed sequentially after tandem 1,3-acyloxy migration/Ferrier rearrangement, a variety of Lewis and Brønsted acids were examined with **2a** but gave either intractable mixtures or incomplete consumption of starting material. To our delight, when the divinyl ketone derivative **2i** was used as the substrate instead, the interrupted Nazarov cyclization product **3i** was furnished. The stereochemistry of compound **3i** was confirmed by X-ray structure analysis of its derivative.^16^ Use of 2 equivalent BF_3_·OEt_2_ with 1 equivalent H_2_O as additive produced the optimal yield of 11-oxatricyclo[5.3.1.0]undecane **3i** (Table 3, entry 3), while using CF_3_SO_3_H or in absence of H_2_O also effected conversion to **3i**, albeit in lower yield (Table 3, entries 2, 4 and 5). H_2_SO_4_ and SnCl_4_ were examined but an intractable mixture was observed even when the reaction was conducted at a lower temperature (Table 3, entries 1 and 6). Reaction on larger scale (0.2 M) also proceeded smoothly and afforded **3i** in 78% yield (Table 3, entry 7).

**Table 3:** Optimization studies of the Nazarov cyclization^*a*^

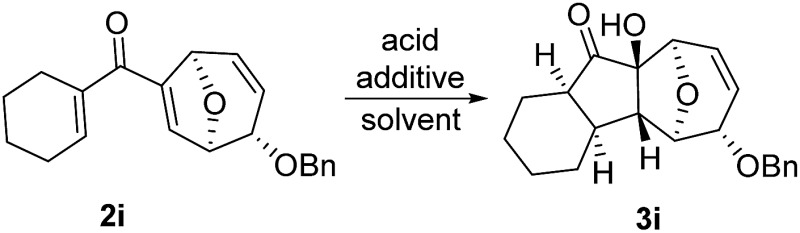					
Entry	Acid	Additive	Solvent	*T*/*t* (h)	Yield^*b*^
1	H_2_SO_4_	—	MeOH	–40 °C/1	Mixture
2	BF_3_·OEt_2_	—	CH_2_Cl_2_	–20 °C/2	Trace
3	BF_3_·OEt_2_	H_2_O	CH_2_Cl_2_	–20 °C/2	77%
4	CF_3_SO_3_H	—	CH_2_Cl_2_	r.t./2	18%
5	CF_3_SO_3_H	H_2_O	CH_2_Cl_2_	r.t./2	32%
6	SnCl_4_	—	CH_2_Cl_2_	–40 °C/1	Mixture
7^*c*^	BF_3_·OEt_2_	H_2_O	CH_2_Cl_2_	–20 °C/2	78%

^*a*^Reaction conditions: divinyl ketone **2i** (0.1 M in CH_2_Cl_2_), acid (2 equiv.), additive (1 equiv.).

^*b*^Isolated yield.

^*c*^Reaction was carried out on scale of 0.2 M of **2i**.

With the optimal interrupted Nazarov cyclization conditions in hand, five 8-oxabicyclo[3.2.1]octane derivatives **2** containing divinyl ketone motif were investigated. Similar with cyclohexenyl ketone **2i**, substituted cyclohexenyl ketone **2j** and cyclooctenyl ketone **2k** could also undergo conversion to the corresponding polycyclic products **3j** and **3k** in good yields (Table 4, entry 3 and 4). In addition, β-substituted ketone **2m** could be applied to form the 11-oxatricyclo[5.3.1.0]undecane **3l**, which was the core structure of homalomenol C (Table 4, entry 5). However, α-substituted ketone **2l** decomposed under the same condition (Table 4, entry 2).

**Table 4:** Substrate scope of interrupted Nazarov cyclization

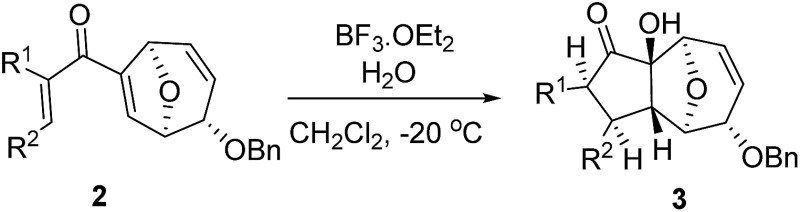			
Entry	Starting material	Solvent	Yield^*b*^
1	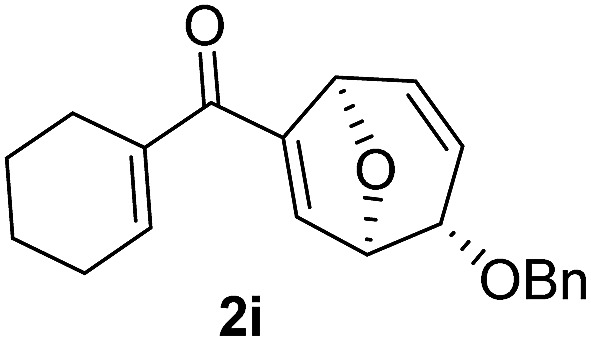	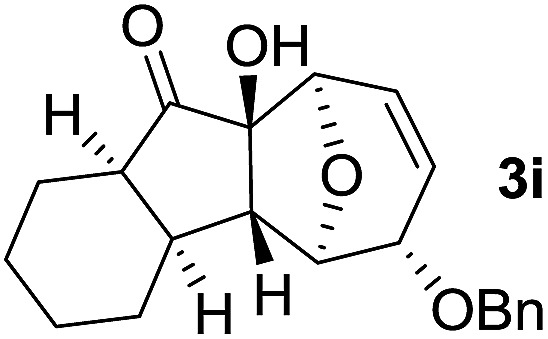	78%
2	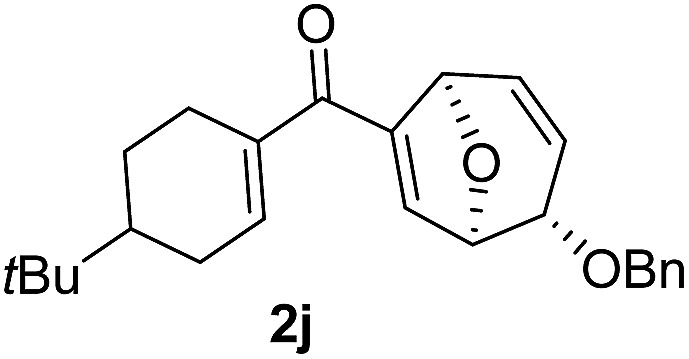	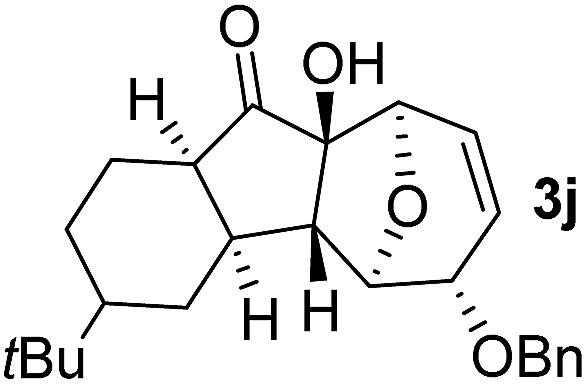	64%
3	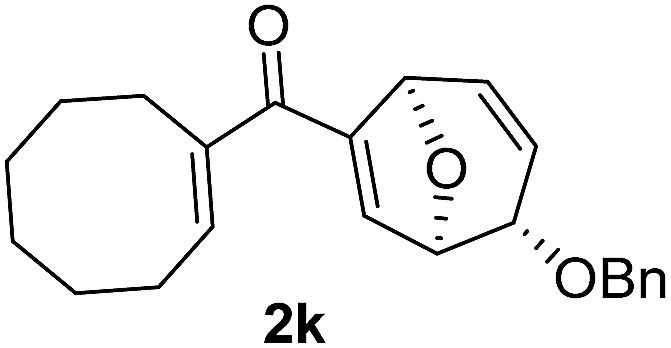	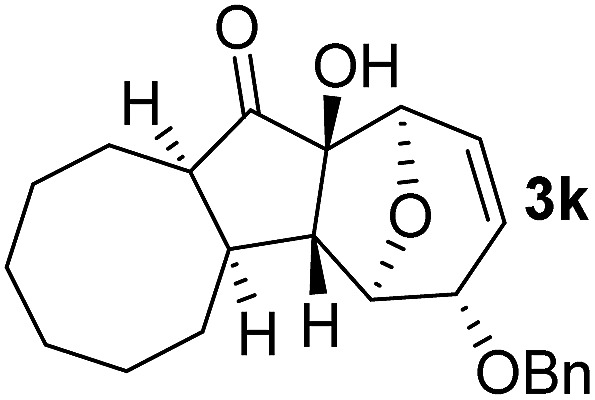	75%
4	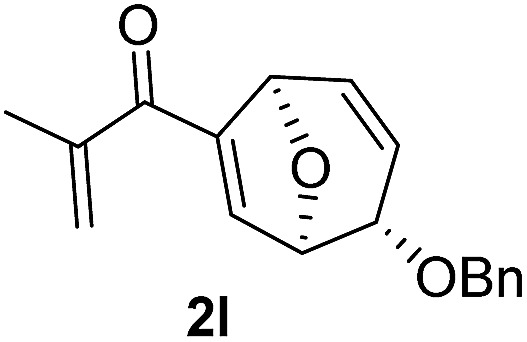	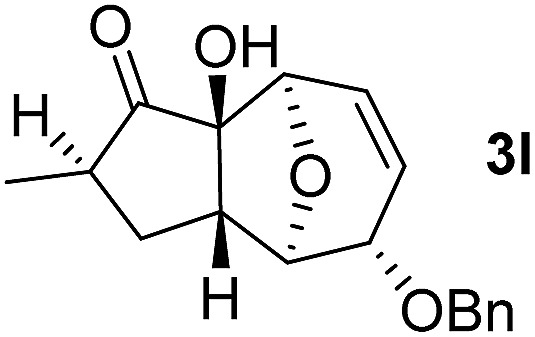	Mixture
5^*c*^	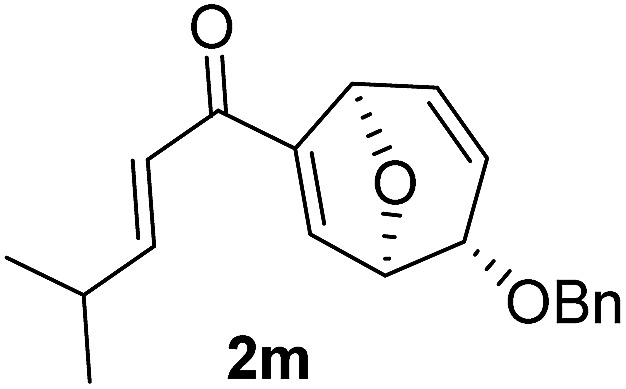	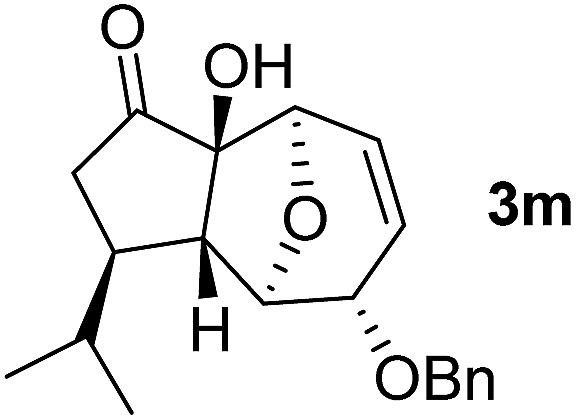	34%

^*a*^Reaction conditions: divinyl ketone **2** (0.2 M in CH_2_Cl_2_), BF_3_·OEt_2_ (2 equiv.), H_2_O (1 equiv.).

^*b*^Isolated yield.

^*c*^Reaction carried out at 0 °C.

The high degree of torquoselectivity of 11-oxatricyclo-[5.3.1.0]undecane **3** is quite interesting in this study and we proposed the mechanism as follow (Scheme 4): activated by the BF_3_·OEt_2_, the pentadienyl cation I was generated and following by a 4π conrotatory electrocyclization to afford the cyclopentenyl cation intermediate **II** with complete exo selectivity. The similiar result about high exo selectivity for Nazarov cyclization of norbornene derivatives^17*b*^ and bicyclo[3.2.1]octane^17g,h^ were previously reported by West *et al.* and it was ascribed to the alkene predistortion such as transition state allylic bond staggering,^17*b*^ combination of alkene pyramidalization,^17*c*^ nonequivalent orbital extension,^17*d*^ steric crowding^17*e*^ and torsional strain.^17*f*^ Houk and co-workers have shown that bicyclo[3.2.1]octane derivatives without through-space interaction would reach quite high exo selectivity in electrocyclization in DFT calculations.^17*a*^ In our case, the nonconjugated alkene does not involve this reaction and the cycloalkene motifs create more steric repulsion and torsional strain in the transition state which further promote the exo stereoselectivity. Sequentially, the external H_2_O trapped the cyclopentenyl cation **II** from the endo face of the polycyclic system which is less bulky after electrocyclization. Finally, the intermediate **IV** was transformed into 11-oxatricyclo[5.3.1.0]undecane **3** through hydrogen shift and enol–keto tautomerism.

**Scheme 4:**
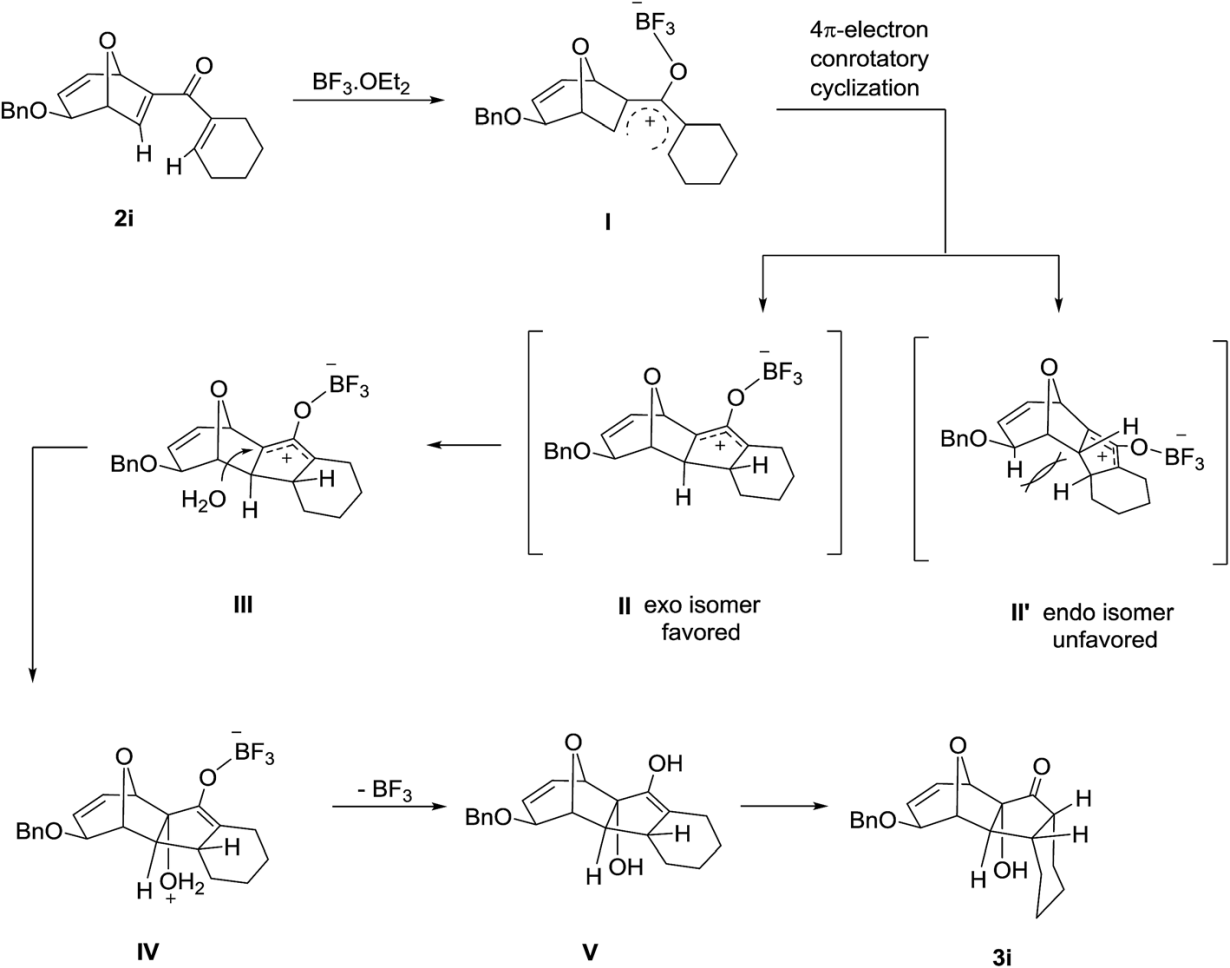
Proposed mechanism of interrupted Nazarov cyclization.

## Conclusion

In summary, a novel homogeneous gold-catalyzed tandem 1,3-acyloxy migration/Ferrier rearrangement was developed successfully to access disubstituted 8-oxabicyclo[3.2.1]octane with high efficiency and complete diastereoselectivity using glycal-derived propargylic esters. The resultant products could then undergo an interrupted Nazarov cyclization serving as an efficient strategy for the facile synthesis of diastereomerically pure 11-oxatricyclo[5.3.1.0]undecanes which was applied to synthesize the core structure of homalomenol C. More studies on mechanism and natural product synthesis are currently in progress.
