# Supplementary material for: Asymmetric syntheses of 8-oxabicyclo[3,2,1]octane and 11-oxatricyclo[5.3.1.0]undecane from glycals
Source: Chem Sci. 2017 Aug 7;8(9):6656–61. doi: 10.1039/c7sc02625k (PMC5625258; doi:10.1039/c7sc02625k)
Supplement: Supplementary file 1 [file SC-008-C7SC02625K-s001.pdf]

Electronic Supplementary Material (ESI) for Chemical Science.
This journal is © The Royal Society of Chemistry 2017

Supporting Information

Asymmetric Syntheses of 8-Oxabicyclo[3,2,1]octane and 11-Oxatricyclo[5.3.1.0]undecane from Glycals

Hongze Liao, Wei Lin Leng, Kim Le Mai Hoang, Hui Yao, Jinxi He, Ying Hui Voo and Xue-Wei Liu*

Division of Chemistry and Biological Chemistry, School of Physical and Mathematical Sciences, Nanyang Technological University, 21 Nanyang Link, Singapore 637371

Supporting Information

Table of Contents：

Section A: General Information

Section B: General procedure for substrate scope synthesis

Section C: Characterization Data for the Isolated Products

Section D: Experimental Procedures and Characterization of Substrates and Products for Mechanistic Studies

Section E: Preparation of 2,4-dinitrophenyl hydrazone S2a for Stereochemistry Identification

Section F: Supplementary Figures

Section A: General Information

General: All the reactions were performed under nitrogen atmosphere. All reagents and solvents were purchased commercially (Alfa Asear, Strem, Merck and Sigma-Aldrich) and used as received. Evaporation of organic solvent was achieved by rotary evaporation with a water bath temperature below 40 ^{o}C. Thin layer chromatography (TLC) with Merck TLC silica gel 60 F254 plate was used to check reaction progress. UV light at 254 nm and basic solution of potassium permanganate were used to visualize compounds on TLC plates. Flash column chromatography with silica gel 60 (0.010-0.063 mm) was used for product purification. ^{1}H and ^{13}C NMR spectra were obtained using 300 MHz Bruker ACF 300, 400 MHz, Bruker AVIII 400 and 400 MHz Bruker DPX 400 spectrometer. Tetramethylsilane (TMS) was used as the internal standard for the measurement of chemical shifts (δ) in ppm. The following abbreviations classify the multiplicity: s (singlet), d (doublet), t (triplet), q (quartet), m (multiplet or unsolved), br s (broad singlet), dd (doublet of doublets), dt (doublet of triplet). The coupling constants were reported as *J* values in units of Hz. HRMS (ESI) spectra were obtained using a Waters Q-Tof premier^{TM} mass spectrometer. X-ray crystallographic data was collected by using a Bruker X8Apex diffractometer with Mo K/α radiation. Characterization data for known compounds were checked in comparison with literature for consistency and not presented in this report.

Section B: General procedure for substrate scope synthesis

General procedure A for preparation of 3,4-protected glycals:

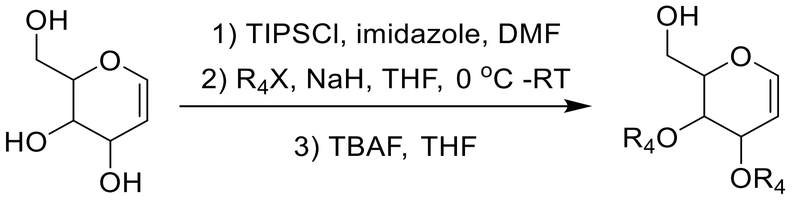

7 **8**

To a solution of glycal **7** (20 mmol, 1 equiv) and imidazole (40 mmol, 2 equiv) in anhydrous DMF (100 mL), TIPSCl (21 mmol, 1.05 equiv) was slowly added dropwise under a N_2 atmosphere and stirred overnight at room temperature. The reaction was poured in to H_2O (150 mL) and extracted with ether (3 × 80 mL), the combined organic phases were washed with brine (80 mL), dried over anhydrous Na_2SO_4, filtered and concentrated *in vacuo* to afford the crude silylated derivative.

The crude silylated compound was dissolved in THF (50 mL) under N_2. NaH (60% in mineral oil, 50 mmol, 2.5 equiv) was added slowly at 0 °C and the solution was stirred at the same temperature for 30 min. R_4X (48 mmol, 2.4 equiv) was added dropwise at 0 °C and the solution was the stirred at room temperature overnight. Methanol (3 mL) was added and the mixture was concentrated *in vacuo*. The residue was dissolved in diethyl ether (200 mL), washed with brine (3 × 80 mL) and concentrated *in vacuo*. The residue was used in next step without other purification.

The fully protected glycal derivative was dissolved in a 1M THF solution of TBAF (45 mmol, 2.25 equiv) and stirred overnight. The mixture was concentrated *in vacuo*

and the product **8** was purified by flash chromatography on silica gel (*n*-Hexane/EtOAc).

General procedure B for preparation of propargylic esters:

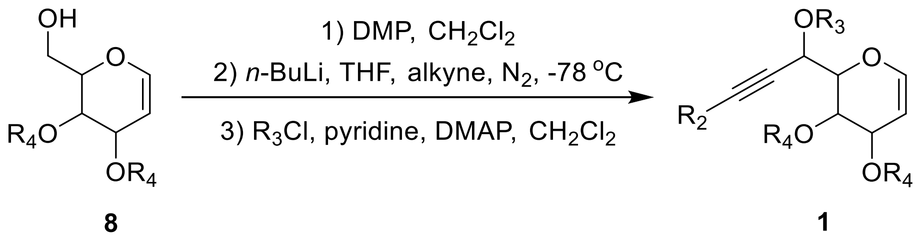

A solution of **8** (1.22 mmol, 1 equiv) in anhydrous CH_2Cl_2 (10 mL) at 0 ^oC was treated with Dess-Martin periodinate (1.72 mmol, 1.4 equiv). The suspension was stirred for 4 h at room temperature under nitrogen. Saturated aqueous NaS_2O_3 (10 mL) and $NaHCO_3$ (10 mL) were added to the mixture and it was stirred until the cloudiness disappeared. The resulting solution was separated and the organic layer was washed with saturated $NaHCO_3$ solution and brine, dried over Na_2SO_4, filtered and concentrated *in vacuo* to afford crude aldehyde as colorless oil.

A solution of alkyne (3.45 mmol, 2.8 equiv) in 5 mL THF was treated at -80 ^oC with a 3.45 mL of a 1 M solution of *n*-BuLi in cyclohexane, stirred for 30 min at -78 ^oC, then treated with a solution of the crude aldehyde in 3 mL THF and stirred overnight. The reaction mixture was quenched by H_2O and extracted with ethyl acetate (3 ×10 mL). The combined organic layers were dried over Na_2SO_4, filtered and concentrated *in vacuo*. After removing the solvent, the residue was purified by flash chromatography on silica gel (*n*-Hexane/EtOAc) to afford the propargylic alcohol.

To a mixture of propargylic alcohol in CH_2Cl_2 (4 mL) was added pyridine (622.3 mg, 8 mmol) and acyl chloride (1.6 mmol) at 0 °C, the reaction was stirred for 4 h. The mixture was diluted with CH_2Cl_2 (10 mL), washed with H_2O (5 mL), saturated $NaHCO_3$ solution (5 mL) and brine (5 mL). The organic layer was dried over Na_2SO_4 and filtered. Evaporation and flash chromatography on silica gel (*n*-Hexane/EtOAc) afforded the propargylic acetate **1**.

General procedure C for preparation of 8-Oxabicyclo[3.2.1]octanes:

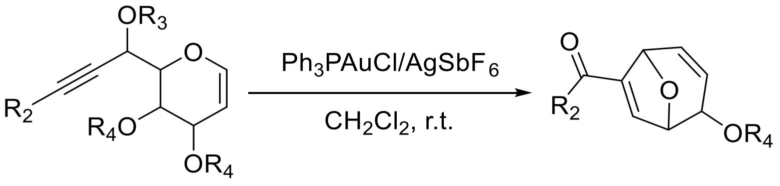

1 **2**

To solution of Ph_3PAuCl (2.5 mg, 5 mol %) and $AgSbF_6$ (3.4 mg, 10 mol %) in distilled CH_2Cl_2 (1 mL), the solution of propargylic acetate **1** (0.1 mmol, 1 equiv) in distilled CH_2Cl_2 (1 mL) was added. The reaction was stirred at room temperature until the starting material was completely consumed. The mixture was filtered through a plug of silica and concentrated *in vacuo*. Purification of the residue by flash chromatography on silica gel (*n*-Hexane/EtOAc) afforded the product **2**.

General procedure D for preparation of 11-oxatricyclo[5.3.1.0]-undecanes:

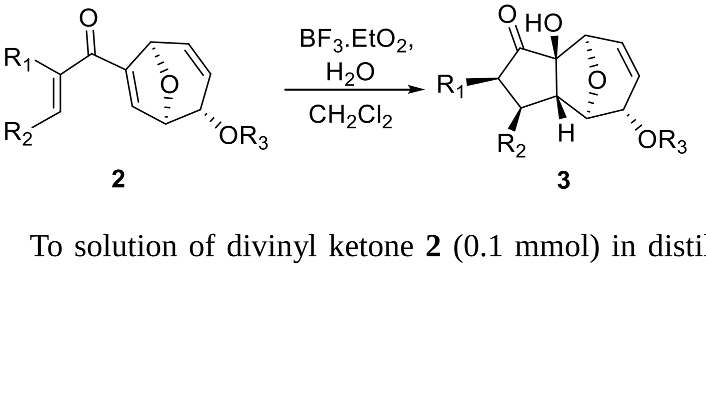

2 **3**

To solution of divinyl ketone **2** (0.1 mmol) in distilled CH_2Cl_2 (1 mL), BF_3•OEt_2

(26.0 μL, 0.2 mmol) and H_2O 1。8 μL, 0.1 mmol) was added dropwise at -78 °C under nitrogen atmosphere successively. The reaction was warmed to -20 °C and stirred until the starting material was completely consumed. Saturated $NaHCO_3$ solution was added and the mixture was stirred vigorously for 10 min. The aqueous layer was extracted with CH_2Cl_2 (3 ×10 mL), the combined organic layers were dried over Na_2SO_4 and filtered. Evaporation and flash chromatography on silica gel (*n*-Hexane/EtOAc) afforded the cyclization product **3**.

Section C: Characterization Data for the Isolated Products

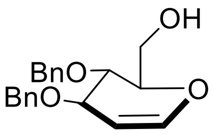

1,5-anhydro-3,4-bis-*O*-benzyl-2-deoxy-D-arabino-hex-1-enitol (8a): Compound was prepared following the general procedure **A**, **8a** was obtained (5.01 g, 76%) after flash chromatography on silica (10:1, *n*-Hexane/EtOAc). $[\alpha]_D^{22}$ = -31.0; (c = 1.0, $CHCl_3$); ^{1}H NMR (400 MHz, $CDCl_3$): δ 7.38-7.29 (m, 10H), 6.41 (dd, J = 0.92, 6.2 Hz, 1H), 4.88-4.83 (m, 2H), 4.73 (d, J = 12.0 Hz, 1H), 4.67-4.64 (m, 2H), 4.19-4.17 (m, 1H), 4.14-4.10 (m, 1H), 4.01-3.96 (m, 2H), 3.78-3.72 (m, 1H), 2.33 (dd, J = 4.1, 8.4 Hz, 1H); ^{13}C NMR (100 MHz, $CDCl_3$): 145.0, 138.2, 138.0, 128.5, 128.5, 128.1, 128.0, 127.8, 127.6, 98.8, 75.7, 72.6, 72.0, 71.2, 69.4, 61.3; HRMS (ESI) calcd. for $[C_{20}H_{23}O_4]^+$, 327.1596; found 327.1596.

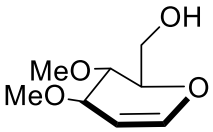

1,5-anhydro-2-deoxy-3,4-bis-*O*-methyl-D-arabino-hex-1-enitol (8b): Compound was prepared following the general procedure **A**, iodomethane (6.80 g, 48 mmol), **8b** was obtained (2.51 g, 72%) after flash chromatography on silica (8:1, *n*-Hexane/EtOAc). $[\alpha]_D^{22}$ = -13.8; (*c* = 1.0, $CHCl_3$); ^1H NMR (400 MHz, $CDCl_3$): δ 6.39 (d, *J* = 6.2 Hz, 1H), 4.86 (dd, *J* = 2.8, 6.1 Hz, 1H), 3.93-3.84 (m, 4H), 3.56 (s, 3H), 3.48 (dd, *J* = 6.0, 7.8 Hz, 1H), 3.41 (s, 3H), 2.10 (t, *J* = 6.6 Hz, 1H); ^{13}C NMR (100 MHz, $CDCl_3$): δ 144.5, 99.6, 77.2, 76.8, 76.4, 61.8, 59.3, 55.8; HRMS (ESI) calcd. for $[C_8H_{14}O_4]^+$, 175.0970; found 175.0972.

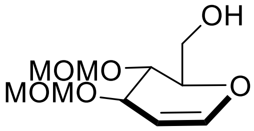

1,5-anhydro-2-deoxy-3,4-bis-*O*-methoxymethyl-D-arabino-hex-1-enitol 8c): Compound was prepared following the general procedure **A**, chloromethyl methyl ether (3.8 g, 48 mmol), **8c** was obtained (3.61 g, 77%) after flash chromatography on silica (10:1, *n*-Hexane/EtOAc). $[\alpha]_D^{22}$ = 1.4; (*c* = 1.0, $CHCl_3$); ^1H NMR (400 MHz, $CDCl_3$): δ 6.39 (d, *J* = 6.1 Hz, 1H), 4.91 (d, *J* = 6.5 Hz, 1H), 4.84 (dd, *J* = 2.7, 6.1 Hz, 1H), 4.76-4.72 (m, 3H), 4.25-4.24 (m, 1H), 3.97-3.81 (m, 4H), 3.44 (s, 3H), 3.40 (s, 3H), 2.67 (dd, *J* = 5.7, 8.1 Hz, 1H); ^{13}C NMR (100 MHz, $CDCl_3$): 144.6, 100.6, 97.8, 95.7, 77.6, 73.8, 73.7, 61.3, 56.2, 55.6; HRMS (ESI) calcd. for $[C_{10}H_{18}O_6Na]^+$, 257.1001; found 257.1004.

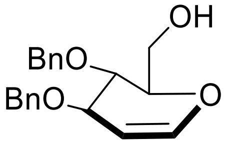

1,5-anhydro-3,4-bis-*O*-benzyl-2-deoxy-D-arabino-hex-5-enitol (8d): Compound was prepared following the general procedure **A**, D-Galactal (2.9 g, 20 mmol), **8d** was obtained (5.18 g, 78%) after flash chromatography on silica (10:1, *n*-Hexane/EtOAc). $[\alpha]_D^{22}$ = -83.1; (c = 1.0, $CHCl_3$); ^1H NMR (400 MHz, $CDCl_3$): δ 7.35-7.29 (m, 10H), 6.41 (dd, J = 1.0 Hz, 6.1 Hz, 1H), 4.90 (dd, J = 2.7, 6.2 Hz, 1H), 4.88 (d, J = 11.8 Hz, 1H), 4.73 (d, J = 11.4 Hz, 1H), 4.68 (d, J = 11.6 Hz, 1H), 4.58 (d, J = 11.6 Hz, 1H), 4.25-4.23 (m, 1H), 3.97-3.93 (m, 1H), 3.88-3.86 (m, 2H), 3.82 (dd, J = 6.2 Hz, 8.5 Hz, 1H), 2.0 (t, J = 6.3 Hz, 1H); ^{13}C NMR (125 MHz, $CDCl_3$): δ 144.6, 138.1, 138.0, 128.5, 128.5, 128.0, 127.9, 127.8, 100.1, 75.5, 74.5, 73.7, 70.6, 61.8; HRMS (ESI) calcd. for $[C_{20}H_{23}O_4]^+$, 327.1596; found 327.1599.

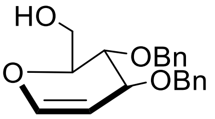

1,5-anhydro-3,4-bis-*O*-benzyl-2-deoxy-L-arabino-hex-5-enitol (8e): Compound was prepared following the general procedure **A**, *L*-Glucal (2.9 g, 20 mmol), **8e** was obtained (5.15 g, 77%) after flash chromatography on silica (10:1, *n*-Hexane/EtOAc). $[\alpha]_D^{22}$ = 23.6; (c = 1.0, $CHCl_3$); ^1H NMR (400 MHz, $CDCl_3$): δ 7.35-7.30 (m, 3H), 6.41 (d, J = 6.1 Hz, 1H), 4.90 (dd, J = 2.7, 6.2 Hz, 1H), 4.87 (d, J = 11.7 Hz, 1H), 4.73 (d, J = 11.5 Hz, 1H), 4.68 (d, J = 11.6 Hz, 1H), 4.58 (d, J = 11.7 Hz), 4.25 (d, J = 5.2 Hz, 1H), 3.97-3.94 (m, 1H), 3.88-3.86 (m, 2H), 3.82 (dd, J = 6.3, 8.5 Hz, 1H), 1.98 (t, J = 6.4 Hz, 1H); ^{13}C NMR (100 MHz, $CDCl_3$): δ 144.6, 138.2, 138.0, 128.5, 128.5, 128.0, 127.9, 127.8, 77.3, 75.6, 74.6, 73.8, 70.6, 61.8; HRMS (ESI) calcd. for $[C_{20}H_{23}O_4]^+$, 327.1596; found 327.1596.

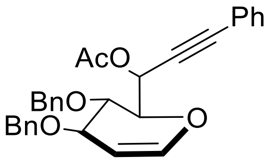

1,5-anhydro-3,4-bis-*O*-benzyl-2-deoxy-6-(1-*O*-acetyl-3-phenylprop-2-yn-1-yl)-D-arabino-hex-1-enitol (1a): Compound was prepared following the general procedure **B**, acyl chloride (124.8 mg, 1.6 mmol), phenylacetylene (352 mg, 3.45 mmol), **1a** was obtained (350.6 mg, 61%, 3 steps) as a 1:1.1 mixture of diastereomers about the propargylic position after flash chromatography on silica (4:1, *n*-Hexane/EtOAc). Obtained as a 1:1.1 mixture of diastereomers about the propargylic position. ^{1}H NMR (400 MHz, $CDCl_3$): δ 7.45-7.28 (m, 15H, both isomers), 6.48 (dd, J = 1.6 , 8.1 Hz, 1H, major isomer), 6.43 (dd, J = 0.8, 8.1 Hz, 1H, minor isomer), 5.03 (dd, J =1.4, 4.6 Hz, 1H, minor isomer), 4.98 (d, J = 14.8 Hz, 1H), 4.94 (dd, J = 2.9, 8.2 Hz, 1H, major isomer), 4.84 (d, J = 14.7 Hz, 1H, major isomer), 4.79 (d, J = 15.5 Hz, 1H, minor isomer), 4.71-4.56 (m, 2H, both isomers), 4.37-4.30 (m, 1H, both isomers), 4.15 (dd, J = 3.8, 13.2 Hz, 1H, major isomer), 4.10-4.05 (m, 1H, both isomers), 3.97 (dd, J = 9.2, 13.2 Hz, 1H, major isomer), 2.15 (s, 3H, major isomer), 2.13 (s, 3H, minor isomer); ^{13}C NMR (100 MHz, $CDCl_3$): δ 169.5, 169.5, 144.3, 144.0, 138.0, 138.0, 137.8, 137.5, 132.1, 131.9, 128.8, 128.8, 128.5, 128.4, 128.4, 128.3, 128.2, 128.0, 128.0, 127.9, 127.8, 127.8, 127.6, 121.9, 121.8, 100.5, 99.9, 87.4, 86.8, 83.7, 82.2, 77.4, 76.9, 76.7, 75.4, 74.5, 72.6, 72.2, 71.9, 70.9, 70.4, 64.1, 61.8, 20.9, 20.9; HRMS (ESI) calcd. for $[C_{30}H_{28}O_5Na]^+$, 491.1834; found 491.1830.

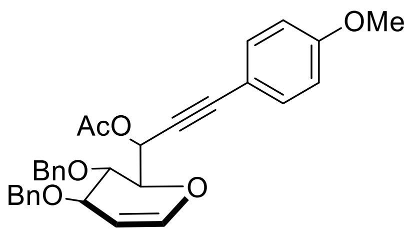

1,5-anhydro-3,4-bis-*O*-benzyl-2-deoxy-6-(1-*O*-acetyl-3-(*p*-methoxyphenyl)prop-2-yn-1-yl)-D-arabino-hex-1-enitol (1b): Compound was prepared following the general procedure **B**, acyl chloride (124.8 mg, 1.6 mmol), 4-ethynylanisole (458.2 mg, 3.45 mmol), **1b** was obtained (382.1 mg, 62%, 3 steps) as a 1.1.5: mixture of diastereomers about the propargylic position after flash chromatography on silica (8:1, *n*-Hexane/EtOAc). ^{1}H NMR (400 MHz, $CDCl_3$): δ 7.38-7.24 (m, 12H, both isomers), 6.83-6.79 (m, 2H, both isomers), 6.47 (d, *J* = 6.1 Hz, 1H, major isomer), 6.43, (d, *J* = 6.2 Hz, 1H, minor isomer), 6.21 (d, *J* = 6.2 Hz, 1H, minor isomer), 6.17(d, *J* = 2.8 Hz, 1H, major isomer), 5.02 (dd, *J* = 6.2, 3.7 Hz, 1H, minor isomer), 4.96 (d, *J* = 11.1 Hz, 1H, major isomer), 4.93 (dd, *J* = 8.2, 2.1 Hz, 1H, major isomer), 4.82 (d, *J* = 11.1 Hz, 1H, major isomer), 4.78 (d, *J* = 11.6 Hz, 1H, minor isomer), 4.68 (d, *J* = 11.6 Hz, 1H, major isomer), 4.65 (d, *J* = 11.6 Hz, minor isomer), 4.64 (d, *J* = 11.6 Hz, 1H, major isomer), 4.55 (s, 2H, minor isomer), 4.36 (dt, *J* = 1.7, 7.0 Hz, 1H, major isomer), 4.30 (dt, *J* = 1 Hz, 5.8 Hz, 1H, minor isomer), 4.14-3.94 (m, 2H, both isomers), 3.81 (s, 3H, both isomers), 2.14 (s,3H, major isomer), 2,12 (s, 3H, minor isomer); ^{13}C NMR (100 MHz, $CDCl_3$): δ 169.6, 169.5, 160.0, 159.9, 144.3, 144.0, 138.0, 138.0, 137.9, 137.5, 133.6, 133.4, 1283.5, 128.4, 128.3, 128.2, 128.0, 127.9, 127.8, 127.8, 127.7, 127.6, 127.6, 113.9, 113.9, 113.8, 100.5, 99.9, 87.4, 86.8, 82.4, 80.8, 77.5, 77.0, 76.8, 75.5, 74.5, 72.6, 72.2, 71.9, 70.9, 70.3, 64.3, 61.9, 55.2, 20.9, 20.9; HRMS (ESI) calcd. for

$[C_{30}H_{31}O_6Na]^+$, 521.1940; found 521.1946.

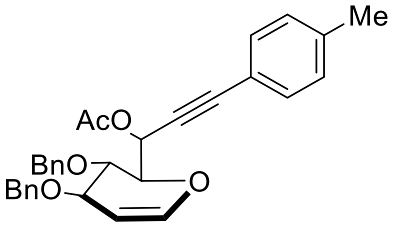

1,5-anhydro-3,4-bis-*O*-benzyl-2-deoxy-6-(1-*O*-acetyl-3-(*p*-methylphenyl)prop-2-yn-1-yl)-D-arabino-hex-1-enitol (1c): Compound was prepared following the general procedure **B**, acyl chloride (124.8 mg, 1.6 mmol), 4-ethynyltoluene (401.0 mg, 3.45 mmol), **1c** was obtained (371.6 mg, 60%, 3 steps) as a 1:1.2 mixture of diastereomers about the propargylic position after flash chromatography on silica (8:1, *n*-Hexane/EtOAc). ^{1}H NMR (400 MHz, $CDCl_3$): δ 7.37-7.19 (m, 12H, both isomer), 7.09-7.05 (m, 2H, both isomer), 6.46 (d, J = 5.3 Hz, 1H, major isomer), 6.42 (d, J = 6.3 Hz, minor isomer), 6.22 (d, J = 6.2 Hz, 1H, minor isomer), 6.18 (d, J = 2.8 Hz, major isomer), 5.00 (dd, J = 3.8, 6.0 Hz, minor isomer), 4.96 (d, J = 11 Hz, 1H, major isomer), 4.91 (dd, J = 2.1, 6.1 Hz, 1H, major isomer), 4.82 (d, J = 11.0 Hz, 1H, major isomer), 4.76 (d, 11.8 Hz, 1H, minor isomer), 4.67 (d, 11.6 Hz, 1H, minor isomer), 4.64 (d, 11.7 Hz, 1H, major isomer), 4.57 (d, 11.6 Hz, 1H, major isomer), 4.53 (s, 1H, minor isomer), 4.35-4.29 (m, 1H, both isomers), 4.14-3.94 (m, 2H, both isomers), 2.32 (s, 3H, both isomers), 2.12 (s, 3H, major isomer), 2.10 (s, 3H, minor isomer); ^{13}C NMR (100 MHz, $CDCl_3$): δ 169.5, 169.4, 144.26, 143.9, 138.9, 138.9, 138.0, 137.9, 137.8, 137.5, 132.0, 131.8, 128.9, 128.4, 128.4, 128.3, 128.2, 128.0, 127.9, 127.8, 121.8, 127.7, 127.6, 127.6, 118.7, 118.7, 100.5, 99.88, 87.5, 86.9, 83.0, 81.4, 77.4, 76.9, 75.4, 74.5, 72.5, 72.1, 71.8, 70.8, 70.2, 64.1, 61.8, 21.4, 20.9; HRMS (ESI)

calcd. for $[C_{31}H_{30}O_5Na]^+$, 505.1991; found 505.1991.

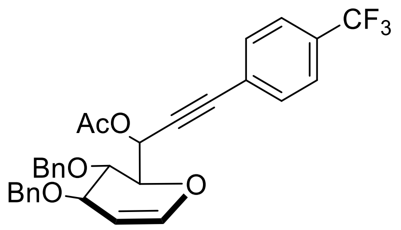

1,5-anhydro-3,4-bis-*O*-benzy-2-deoxyl-6-(1-*O*-acetyl-3-(4-trifluoromethyl-phenyl) prop-2-yn-1-yl)-D-arabino-hex-1-enitol (1d): Compound was prepared following the general procedure **B**, acyl chloride (124.8 mg, 1.6 mmol), 4-ethynyl-α,α,α-trifluorotouene (586.5 mg, 3.45 mmol), **1d** was obtained (382.2 mg, 58%, 3 steps) as a 1:1.2 mixture of diastereomers about the propargylic position after flash chromatography on silica (8:1, *n*-Hexane/EtOAc). ^{1}H NMR (400 MHz, $CDCl_3$): δ 7.56-7.49 (m, 3H, both isomers), 7.39-7.27 (m, 11H, both isomers), 6.47 (dd, J = 1.0, 6.1 Hz, 1H, major isomer), 6.45 (dd, J = 0.6, 6.2 Hz, 1H, minor isomer), 6.21 (d, J = 5.8 Hz, 1H, minor isomer), 6.17 (d, J = 2.9 Hz, 1H, major isomer), 5.04 (dd, J = 3.6, 6.2 Hz, 1H, minor isomer), 4.99-4.95 (m, 1H, both isomers), 4.83 (d, J = 5.4 Hz, 1H, major isomer), 4.80 (d, J = 5.9 Hz, 1H, minor isomer), 4.70 (d, J = 11.5 Hz, 1H, major isomer), 4.64 (d, J = 11.6 Hz, 1H, minor isomer), 4.61 (d, J = 6.2 Hz, 1H, minor isomer), 4.58 (d, J = 6.1 Hz, 1H, major isomer), 4.54 (d, J = 11.7 Hz, 1H, minor isomer), 4.36 (dt, J = 1.8, 6.8 Hz, 1H, major isomer), 4.30 (td, J = 0.64, 5.9 Hz, 1H, minor isomer), 4.16 (dd, J = 2.9, 9.8 Hz, 1H, major isomer), 4.12-4.09 (m, 1H, minor isomer), 4.04-4.01 (m, 1H, minor isomer), 3.94 (dd, J = 6.4, 9.8 Hz, 1H, major isomer), 2.16 (s, 3H, major isomer), 2.13 (s, 3H, minor isomer); ^{13}C NMR (100 MHz, $CDCl_3$): δ 169.6, 169.5, 144.3, 144.0, 138.0, 137.0, 137.7, 137.5, 132.4, 132.2, 130.6

(q, $J_{(C\text{-}F)}$ = 29.9 Hz), 128.6, 128.5, 128.5, 128.5, 128.3, 128.1, 127.9, 127.9, 127.8, 127.6, 125.7 (q, $J_{(C\text{-}F)}$ = 1.8 Hz), 125.2 (q, $J_{(C\text{-}F)}$ = 3.6 Hz), 121.1 (q, $J_{(C\text{-}F)}$ = 271.2 Hz), 100.6, 100.1, 86.3, 85.8, 85.3, 84.8, 76.8, 75.0, 74.4, 72.7, 72.6, 71.9, 70.9, 70.4, 64.0, 61.6, 20.9, 20.9; HRMS (ESI) calcd. for $[C_{31}H_{28}F_3O_5]^+$, 537.1889; found 537.1899.

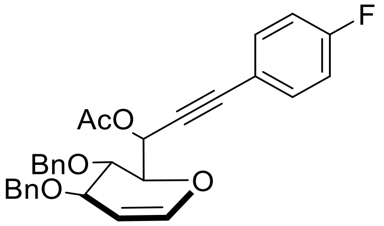

1,5-anhydro-3,4-bis-*O*-benzyl-2-deoxy-6-(1-*O*-acetyl-3-(4-fluorophenyl)prop-2-yn-1-yl)-D-arabino-hex-1-enitol (1e): Compound was prepared following the general procedure **B**, acyl chloride (124.8 mg, 1.6 mmol), 1-ethynyl-4-fluorobenzene (414.0 mg, 3.45 mmol), (**1e** was obtained (341.0 mg, 57%, 3 steps) as a 1:1.1 mixture of diastereomers about the propargylic position after flash chromatography on silica (8:1, *n*-Hexane/EtOAc). ^{1}H NMR (400 MHz, $CDCl_3$): δ 7.41-7.22 (m, 12H, both isomers), 7.01-6.95 (m, 2H, both isomers), 6.48 (d, J = 6.0 Hz, 1H, major isomer), 6.45 (d, J = 6.2 Hz, 1H, minor isomer), 6.20 (d, J = 5.9 Hz, 1H, major isomer), 6.17 (d, J = 2.8 Hz, 1H, major isomer), 5.04 (dd, J = 3.6, 6.1 Hz, 1H, minor isomer), 4.99-4.94 (m, 1H, both isomers), 4.84-4.53 (m, 3H, both isomers), 4.36 (dt, J = 1.6, 6.9 Hz, 1H, major isomer), 4.30 (dt, J = 0.6, 5.8 Hz, 1H, minor isomer), 4.16 (dd, J = 2.9, 9.9 Hz, 1H, major isomer), 4.10-4.08 (m, 1H, minor isomer), 4.06-4.04 (m, 1H, minor isomer), 3.96 (dd, J = 6.9, 9.9 Hz, 1H, major isomer), 2.15 (s, 1H, major isomer), 2.13 (s, 1H, minor isomer); ^{13}C NMR (100 MHz, $CDCl_3$): δ 169.5, 169.4, 164.0, 164.0, 161.5, 161.5, 144.3, 144.0, 138.0, 137.9, 137.8, 137.5, 134.1, 134.0, 133.89, 133.8, 128.5,

128.4, 128.4, 128.4, 128.2, 128.0, 127.9, 127.9, 127.6, 127.6, 117.9, 117.9, 115.6, 115.4, 100.5, 100.0, 86.3, 85.7, 83.5, 82.0, 77.4, 76.9, 76.7, 75.2, 74.4, 72.6, 72.4, 72.4, 72.0, 70.9, 70.3, 64.1, 61.7, 20.9; HRMS (ESI) calcd. for $[C_{30}H_{28}FO_5]^+$, 487.1921; found 487.1921.

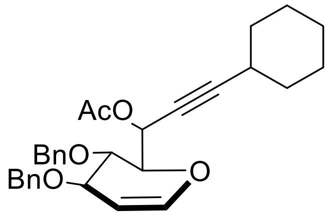

1,5-anhydro-3,4-bis-*O*-benzyl-2-deoxy-6-(1-*O*-acetyl-3-cyclohexylprop-2-yn-1-yl)-D-arabino-hex-1-enitol (1f): Compound was prepared following the general procedure **B**, acyl chloride (124.8 mg, 1.6 mmol), cyclohexylacetylene (373.2 mg, 3.45 mmol), **1f** was obtained (396.4 mg, 65%, 3 steps) as a 1:3 mixture of diastereomers about the propargylic position after flash chromatography on silica (8:1, *n*-Hexane/EtOAc). ^{1}H NMR (400 MHz, $CDCl_3$): δ 7.37-7.28 (m, 10H, both isomers), 6.45 (dd, J = 1.7, 6.0 Hz, 1H, major isomer), 6.41 (d, J = 6.2 Hz, 1H, minor isomer), 6.02, (dd, J = 1.8, 6.3 Hz, 1H, minor isomer), 5.99 (t, J = 2.4 Hz, 4.7 Hz, 1H, major isomer), 5.00-4.98 (m, 1H, minor isomer), 4.95 (d, J = 11.0 Hz, 1H, major isomer), 4.90 (dd, J = 2.1, 6.1 Hz, major isomer), 4.80 (d, J = 11.0 Hz, 1H, major isomer), 4.77 (d, J = 11.8 Hz, minor isomer), 4.69 (d, J = 11.6 Hz, 1H, major), 4.65 (d, J = 11.8 Hz, 1H, minor isomer), 4.60 (d, J = 11.6 Hz, 1H, major isomer), 4.54 (s, 2H, minor isomer), 4.34 (dt, J = 1.7, 7.1 Hz, 1H, major isomer), 4.22-4.18 (m, 1H, minor isomer), 4.06 (d, J = 2.8 Hz, 1H, minor isomer), 4.03 (d, J = 2.6 Hz, 1H, major isomer), 3.91 (d, J = 7.1 Hz, 1H, major isomer), 3.89 (d, J = 7.0 Hz, 1H, minor isomer), 2.45-2.41

(m, 1H, major isomer), 2.35-2.31 (m, 1H, minor isomer), 2.12 (s, 3H, major isomer), 2.09 (s, 3H, minor isomer), 1.81-1.64 (m, 4H, both isomers), 1.53-1.42 (m, 2H, both isomers) 1.33-1.24 (m, 4H, both isomers); ^{13}C NMR (100 MHz, $CDCl_3$): *δ* 169.5, 169.5, 144.3, 144.0, 138.1, 138.0, 137.9, 137.6, 128.4, 128.4, 128.4, 128.3, 128.1, 127.9, 127.8, 127.8, 127.7, 127.6, 127.5, 100.4, 99.7, 92.7, 91.9, 77.4, 76.8, 75.8, 74.7, 73.1, 72.4, 71.9, 70.8, 70.2, 64.1, 61.6, 32.3, 32.3, 32.2, 32.2, 29.00, 28.9, 25.7, 25.7, 24.7, 21.0; HRMS (ESI) calcd. for $[C_{30}H_{34}O_5Na]^+$ 497.2307; found 497.2307.

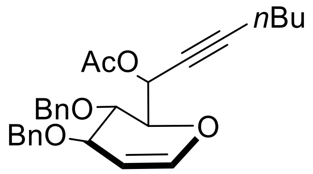

1,5-anhydro-3,4-bis-*O*-benzyl-2-deoxy-6-(1-*O*-acetylhept-2-yn-1-yl)-D-arabino-hex-1-enitol (1g): Compound was prepared following the general procedure **B**, acyl chloride (124.8 mg, 1.6 mmol), 1-hexyne (283.2 mg, 3.45 mmol), **1g** was obtained (352.6 mg, 61%, 3 steps) as a 1:2.1 mixture of diastereomers about the propargylic position after flash chromatography on silica (8:1, *n*-Hexane/EtOAc). ^{1}H NMR (400 MHz, $CDCl_3$): *δ* 7.36-7.29 (m, 10H, both isomers), 6.44 (dd, *J* = 0.84, 6.9 Hz, 1H, major isomer), 6.41 (d, *J* = 6.2 Hz, 1H, minor isomer), 5.97-5.93 (m, 1H, both isomers), 4.97 (dd, *J* = 3.5, 6.2 Hz, 1H, minor isomer), 4.94 (d, *J* = 11.0 Hz, 1H, major isomer), 4.90 (dd, *J* = 2.1 Hz, 6.1 Hz, 1H, major isomer), 4.79 (d, *J* = 11.0 Hz, major isomer), 4.82 (d, *J* = 11.4 Hz, 1H, minor isomer), 4.68 (d, *J* = 11.5 Hz, major isomer), 4.62 (d, *J* = 11.6 Hz, 1H, minor isomer), 4.69 (d, *J* = 11.6 Hz, major isomer), 4.56 (s, 2H, minor isomer), 4.32 (dt, *J* = 1.7, 7.0 Hz, 1H, major isomer), 4.15 (t, *J* =

5.8 Hz, 1H, minor isomer), 4.07 (t, J = 8.0 Hz, 1H, minor isomer), 4.03 (dd, J = 2.8, 10.0 Hz, 1H, major isomer), 3.99-3.96 (m, 1H, minor isomer), 3.88 (dd, J = 7.0, 10.0 Hz, 1H, major isomer), 2.23 (dt, J = 2.0, 7.0 Hz, 2H, major isomer), 2.15 (dt, J = 1.8, 6.9 Hz, 2H, minor isomer), 2.11 (s, 3H, major isomer), 2.08 (s, 3H, minor isomer), 1.53-1.31 (m, 4H, both isomers), 0.91-0.87 (s, 3H, major isomer), 0.88-0.85 (s, 3H, minor isomer); ^{13}C NMR (100 MHz, $CDCl_3$): δ 169.6, 169.5, 144.3, 144.1, 138.1, 138.1, 137.9, 137.7, 128.4, 128.4, 128.2, 128.0, 127.9, 127.8, 127.8, 127.8, 127.6, 127.6, 100.4, 99.8, 88.8, 87.8, 77.5, 75.5, 74.8, 74.5, 73.1, 72.8, 72.8, 72.3, 70.8, 70.3, 64.1, 61.7, 30.4, 30.4, 21.9, 21.9, 21.0, 18.5, 18.4, 13.5; HRMS (ESI) calcd. for $[C_{28}H_{32}O_5Na]^+$, 471.2147; found 471.2158.

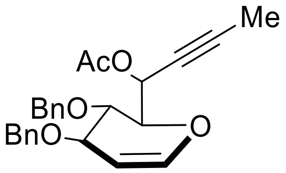

1,5-anhydro-3,4-bis-*O*-benzyl-2-deoxy-6-(1-*O*-acetylbut-2-yn-1-yl)-D-arabino-Hex-1-enitol (1h): Compound was prepared following the general procedure **B**, acyl chloride (124.8 mg, 1.6 mmol), 1-propynylmagnesium bromide solution (0.5 M in THF, 6.9 mL, 3.45 mmol), **1h** was obtained (159.6 mg, 52%, 3 steps) as a 1:3 mixture of diastereomers about the propargylic position after flash chromatography on silica (8:1, *n*-Hexane/EtOAc). ^{1}H NMR (400 MHz, $CDCl_3$): δ 7.38-7.29 (m, 10H, both isomers), 6.45 (dd, J = 1.0, 6.1 Hz, 1H, major isomer), 6.43 (dd, J = 0.8, 6.2 Hz, 1H, minor isomer), 5.93-5.89 (m, 1H, both isomers), 4.99-4.91 (m, 2H, both isomers), 4.81-4.77 (m, 1H, both isomers), 4.70-4.53 (m, 2H, both isomers), 4.33 (dt, J = 1.8,

6.9 Hz, 1H, major isomer), 4.15-4.10 (m, 1H, both isomers), 4.04 (dd, *J* = 2.8, 10 Hz, 1H major isomer), 3.95 (dd, *J* = 1.6, 5.0 Hz, 1H, minor isomer) 3.90 (dd, *J* = 7.0 , 10 Hz, 1H, major isomer), 2.11 (s, 3H, major isomer), 2.08 (s, 3H, minor isomer), 1.84 (d, *J* = 2.2 Hz, 3H, major isomer), 1.81 (d, *J* = 2.2 Hz, 3H, minor isomer); ^{13}C NMR (100 MHz, CDCl3): δ 169.6, 169.5, 144.3, 144.0, 138.0, 138.0, 137.8, 137.5, 128.4, 128.3, 128.3, 128.0, 127.9, 127.8, 127.7, 127.6, 100.3, 100.0, 84.2, 83.4, 77.4, 77.0, 75.1, 74.3, 74.0, 73.4, 72.9, 72.3, 72.2, 70.8, 70.4, 64.0, 61.7, 20.9, 20.9, 3.8, 3.6; HRMS (ESI) calcd. for $[C_{25}H_{27}O_5]^+$, 407.1858; found 407.1851.

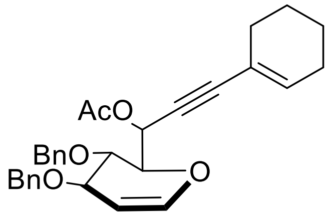

1,5-anhydro-3,4-bis-*O*-benzyl-2-deoxy-6-(1-*O*-acetyl-3-(cyclohex-1-enyl)prop-2-yn-1-yl)-D-arabino-Hex-1-enitol (1i): Compound was prepared following the general procedure **B**, acyl chloride (124.8 mg, 1.6 mmol), 1-ethynycyclohexene (345.0 mg, 3.45 mmol), **1i** was obtained (360.0 mg, 62%, 3 steps) as a 1:3.5 mixture of diastereomers about the propargylic position after flash chromatography on silica (8:1, *n*-Hexane/EtOAc). ^{1}H NMR (300 MHz, $CDCl_3$): δ 7.38-7.29 (m, 10H, both isomers), 6.45 (d, *J* = 6.0 Hz, 1H, major isomer), 6.41 (d, *J* = 6.2 Hz, 1H, minor isomer), 6.16-6.14 (m, 1H, major isomer), 6.11 (d, *J* = 6.1 Hz, 1H, minor isomer), 6.08 (s, 1H, major isomer), 6.05-6.03 (m, 1H, minor isomer), 4.99 (dd, *J* = 4.2, 6.3 Hz, 1H, minor isomer), 4.95 (d, *J* = 11.0 Hz, 1H, major isomer), 4.91 (d, *J* = 2.0, 6.1 Hz, 1H, major isomer), 4.80 (d, *J* = 11.0 Hz, 1H, major isomer), 4.75 (d, *J* = 11.7 Hz, 1H, minor

isomer), 4.68 (d, J = 11.5 Hz, 1H, major isomer), 4.64 (d, J = 11.6 Hz, 1H, minor isomer), 4.69 (d, J = 11.5 Hz, 2H, major isomer), 4.54 (s, 2H, minor isomer), 4.33 (dt, J = 1.6, 7.0 Hz, 1H, major isomer), 4.22 (td, J = 1.5, 5.6 Hz, 1H, major isomer), 4.07 (dd, J = 2.8, 10.0 Hz, 1H, major isomer), 4.03-4.01 (m, 2H, minor isomer), 3.90 (dd, J = 7.1, 10.0 Hz, 1H, major isomer), 2.12 (s, 3H, major isomer), 2.11-2.00 (m, 4H, both isomers), 1.63-1.55 (m, 4H, both isomers); ^{13}C NMR (100 MHz, $CDCl_3$): δ 169.5, 144.3, 144.0, 138.1, 138.0, 137.9, 136.6, 136.4, 128.4, 128.4, 128.3, 128.2, 128.0, 127.9, 127.9, 127.8, 127.6, 127.6, 119.6, 100.4, 99.8, 89.4, 79.3, 77.4, 77.2, 76.8, 75.7, 74.6, 72.5, 72.2, 71.9, 70.9, 70.3, 64.3, 61.9, 28.9, 28.8, 25.6, 22.1, 21.3, 20.9; HRMS (ESI) calcd. for $[C_{30}H_{33}O_5]^+$, 473.2328; found 473.2330.

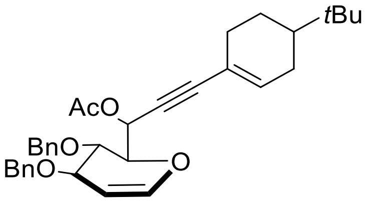

1,5-anhydro-3,4-bis-*O*-benzyl-2-deoxy-6-(1-*O*-acetyl-3-(4-*t*Bu-cyclohex-1-enyl)prop-2-yn-1-yl)-D-arabino-Hex-1-enitol (1j): Compound was prepared following the general procedure **B**, acyl chloride (124.8 mg, 1.6 mmol), 4-(1,1-dimethylethyl)-1-ethynyl-cyclohexene (559.0mg, 3.45 mmol), **1j** was obtained (404.2 mg, 64%, 3 steps) as a 1:1.3 mixture of diastereomers about the propargylic position after flash chromatography on silica (8:1, *n*-Hexane/EtOAc). ^{1}H NMR (400 MHz, $CDCl_3$): δ 7.39-7.29 (m, 10H, both isomers), 6.45 (d, J = 6.1 Hz, 1H, major isomer), 6.42 (d, J = 6.2 Hz, 1H, minor isomer), 6.17 (br, 1H, major isomer), 6.13 (d, J = 6.9 Hz, 1H, minor isomer), 6.10 (d, J = 2.7 Hz, 1H, major isomer), 6.06 (br, 1H,

minor isomer), 4.99 (dd, *J* = 3.4, 6.2 Hz, 1H, minor isomer), 4.98 (d, *J* = 11.0 Hz, 1H, major isomer), 4.92 (d, *J* = 2.0, 6.1 Hz, 1H, major isomer), 4.81 (d, *J* = 11.0 Hz, 1H, major isomer), 4.77 (d, *J* = 11.6 Hz, 1H, minor isomer), 4.69 (d, *J* = 11.6 Hz, 1H, major isomer), 4.65 (d, *J* = 11.7 Hz, 1H, minor isomer), 4.59 (d, *J* = 11.5 Hz, 1H, major isomer), 4.55 (s, 2H, minor isomer), 4.33 (dt, *J* = 1.7, 7.0 Hz, 1H, major isomer), 4.24 (td, *J* = 1.6, 6.3 Hz, 1H, minor isomer), 4.08 (dd, *J* = 2.9, 9.9 Hz, 1H, major isomer), 4.04-4.02 (m, 2H, minor isomer), 3.91 (dd, *J* = 7.0, 9.9 Hz, 1H, major isomer), 2.30-2.27 (m, 1H, both isomers), 2.21-2.21 (m, 3H, both isomers), 2.12 (s, 3H, major isomer), 2.10 (s, 3H, minor isomer), 1.58-1.40 (m, 8H, both isomers); ^{13}C NMR (100 MHz, $CDCl_3$): δ 169.5, 169.4, 144.3, 144.0, 138.0, 138.0, 137.9, 137.6, 137.0, 136.9, 136.8, 136.7, 128.4, 128.4, 128.4, 128.3, 128.2, 128.0, 127.9, 127.9, 127.8, 127.7, 127.6, 127.6, 119.3, 100.4, 99.8, 89.1, 88.4, 81.1, 79.5, 77.4, 77.0, 76.7, 75.7, 75.6, 74.6, 72.5, 72.2, 71.9, 70.9, 70.3, 64.2, 61.8, 43.0, 32.1, 30.4, 30.3, 27.3, 27.0, 23.6, 20.9; HRMS (ESI) calcd. for $[C_{34}H_{41}O_5]^+$, 529.2954; found 529.2952.

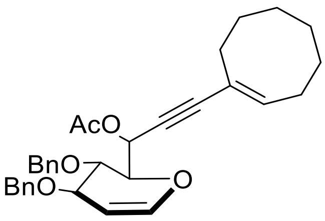

1,5-anhydro-3,4-bis-*O*-benzyl-2-deoxy-6-(1-*O*-acetyl-3-(cycloocten-1-enyl)prop-2-yn-1-yl)-D-arabino-Hex-1-enitol (1k): Compound was prepared following the general procedure **B**, acyl chloride (124.8 mg, 1.6 mmol), 1-ethynylcyclooctene (462.3 mg, 3.45 mmol), **1k** was obtained (387.6 mg, 66%, 3 steps) as a 1:1.1 mixture of diastereomers about the propargylic position after flash chromatography on silica

(8:1, *n*-Hexane/EtOAc). ^{1}H NMR (400 MHz, $CDCl_3$): δ 7.36-7.28 (m, 10H, both isomers), 6.45 (d, *J* = 6.2 Hz, 1H, major isomer), 6.41 (d, *J* = 6.2 Hz, 1H, minor isomer), 6.27-5.99 (m, 2H, both isomers), 4.99 (dd, *J* = 3.9, 5.4 Hz, 1H, minor isomer), 4.97 (d, *J* = 11.0 Hz, 1H, major isomer), 4.90 (d, *J* = 2.1, 6.1 Hz, 1H, major isomer), 4.79 (d, *J* = 11.0 Hz, 1H, major isomer), 4.74 (d, *J* = 11.8 Hz, 1H, minor isomer), 4.68-4.63 (m, 1H, both isomers), 4.60-4.55 (m, 1H, both isomers), 4.52 (d, *J* = 11.8 Hz, 1H, minor isomer), 4.33 (dt, *J* = 1.7, 7.1 Hz, 1H, major isomer), 4.24 (td, *J* = 1.0, 6.0 Hz, 1H, minor isomer), 4.07 (dd, *J* = 2.8, 10.0 Hz, 1H, major isomer), 4.04-4.00 (m, 2H, major), 3.90 (dd, *J* = 7.1, 9.9 Hz, 1H, major isomer), 2.17-2.05 (m, 3H, both isomers), 2.12 (s, 3H, major isomer), 2.10 (s, 3H, minor isomer), 1.88-1.80 (m, 2H, both isomers), 1.28-1.11 (m, 2H, both isomers), 0.87 (s, 9H, both isomers); ^{13}C NMR (100 MHz, $CDCl_3$): δ 169.6, 169.5, 144.4, 144.0, 139.5, 139.3, 138.1, 138.0, 137.9, 137.6, 128.5, 128.4, 128.4, 128.4, 128.1, 128.0, 127.8, 127.8, 127.7, 127.6, 127.5, 122.6, 100.5, 99.8, 90.1, 89.4, 80.4, 78.8, 77.5, 76.7, 72.4, 71.9, 71.7, 70.8, 70.2, 64.3, 61.9, 29.8, 29.6, 29.5, 29.5, 28.4, 28.3, 26.9, 26.9, 26.3, 26.2, 25.7, 25.7, 21.0; HRMS (ESI) calcd. for $[C_{32}H_{37}O_5]^+$, 501.2641; found 501.2644.

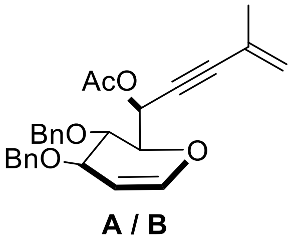

A / B

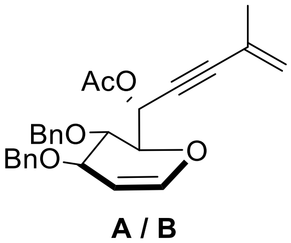

A / B

1,5-anhydro-3,4-bis-*O*-benzyl-2-deoxy-6-(1-*O*-acetyl-4-methylbut-4-en-2-yn-1-yl)-D-arabino-Hex-1-enitol (1l-A): Compound was prepared following the general

procedure **B**, acyl chloride (124.8 mg, 1.6 mmol), isopropenylacetylene (228.0 mg, 3.45 mmol), **1l-A** was obtained (165.4 mg, 31%, 3 steps) after flash chromatography on silica (8:1, *n*-Hexane/EtOAc). Rf 0.44 (3:1 Hexane/EtOAc); $[\alpha]_D^{22}$ = 38.2; (*c* = 1.0, $CHCl_3$); ^{1}H NMR (400 MHz, $CDCl_3$): δ 7.38-7.28 (m, 10H), 6.46-6.44 (dd, *J* = 1.1, 6.0 Hz, 1H), 6.08-6.08 (d, *J* = 2.7 Hz, 1H), 5.35 (br, 1H), 5.28-5.27 (t, *J* = 1.6 Hz, 1H), 4.97-4.94 (d, *J* = 11.1 Hz, 1H), 4.93-4.91 (dd, *J* = 2.2, 6.1 Hz, 1H), 4.81-4.78 (d, *J* = 11.1 Hz, 1H), 4.69-4.67 (d, *J* = 11.5 Hz, 1H), 4.60-4.57 (d, *J* = 11.5 Hz, 1H), 4.34-4.32 (dt, *J* = 1.8, 7.0 Hz, 1H), 4.10-4.07 (dd, *J* = 2.9, 10.0 Hz, 1H), 3.91-3.87 (dd, *J* = 7.0, 10.0 Hz, 1H), 2.13 (s, 3H), 1.88 (s, 3H); ^{13}C NMR (100 MHz, CDCl3): δ 169.4, 144.3, 138.0, 137.8, 128.4, 128.4, 128.0, 127.9, 127.8, 127.8, 125.7, 123.5, 100.5, 88.6, 81.1, 77.3, 76.9, 75.4, 74.5, 70.9, 64.0, 23.2, 20.9; HRMS (ESI) calcd. for $[C_{27}H_{29}O_5]^+$, 433.2015; found 433.2010.

1,5-anhydro-3,4-bis-*O*-benzyl-2-deoxy-6-(1-*O*-acetyl-4-methylbut-4-en-2-yn-1-yl)-D-arabino-Hex-1-enitol (1l-B): Compound was prepared following the general procedure **B**, acyl chloride (124.8 mg, 1.6 mmol), isopropenylacetylene (228.0 mg, 3.45 mmol), **1l-B** was obtained (166.9 mg, 32%, 3 steps) after flash chromatography on silica (8:1, *n*-Hexane/EtOAc). Rf 0.41 (3:1 Hexane/EtOAc); $[\alpha]_D^{22}$ = 1.4; (*c* = 1.0, $CHCl_3$); ^{1}H NMR (400 MHz, $CDCl_3$): δ 7.36-7.28 (m, 10H), 6.43-6.40 (dd, *J* = 0.8, 6.2 Hz, 1H), 6.13-6.11 (d, *J* = 6.0 Hz, 1H), 5.25-5.23 (m, 2H), 5.01-4.98 (dd, *J* = 3.6, 6.2 Hz, 1H), 4.79-4.75 (d, *J* = 11.6 Hz, 1H), 4.65-4.61 (d, *J* = 11.6 Hz, 1H), 4.55 (s, 2H), 4.25-4.20 (dt, *J* = 1.1, 5.9 Hz, 1H), 4.06-3.98 (m, 2H), 2.10 (s, 3H), 1.80 (s, 3H); ^{13}C NMR (100 MHz, $CDCl_3$): δ 169.5, 144.0, 138.0, 137.6, 128.5, 128.4, 128.2, 128.0,

127.6, 127.6, 125.7, 123.3, 99.9, 87.9, 82.7, 77.2, 72.6, 72.3, 71.9, 70.3, 61.3, 23.0, 20.9; HRMS (ESI) calcd. for $[C_{27}H_{29}O_5]^+$, 433.2015; found 433.2018.

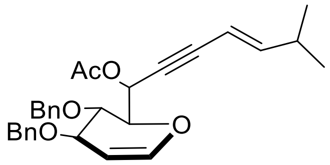

1,5-anhydro-3,4-bis-*O*-benzyl-2-deoxy-6-(1-*O*-acetyl-6-methyl-4-hepten-2-yn-1-yl)-D-arabino-Hex-1-enitol (1m): Compound was prepared following the general procedure **B**, acyl chloride (124.8 mg, 1.6 mmol), (3*E*)-5-methyl-3-hexen-1-yne (324.3 mg, 3.45 mmol), **1m** was obtained (362.3 mg, 64%, 3 steps) as a 1:3.5 mixture of diastereomers about the propargylic position after flash chromatography on silica (8:1, *n*-Hexane/EtOAc). ^{1}H NMR (300 MHz, $CDCl_3$): δ 7.36-7.27 (m, 10H, both isomers), 6.45 (d, J = 6.0 Hz, 1H, major isomer), 6.41 (d, J = 5.7 Hz, 1H, minor isomer), 6.16 (dd, J = 6.8, 16.0 Hz, 1H, major isomer), 6.10-6.06 (m, 1H, minor isomer), 6.07-6.04 (m, 1H, both isomers), 5.43-5.36 (m, 1H, both isomers), 4.98 (dd, J = 3.6, 6.1 Hz, 1H, minor isomer), 4.94 (d, J = 11.3 Hz, 1H, major isomer), 4.91 (d, J = 2.1, 6.2 Hz, 1H, major isomer), 4.78 (d, J = 11.1 Hz, 1H, major isomer), 4.76 (d, J = 11.4 Hz, 1H, minor isomer), 4.68 (d, J = 11.5 Hz, 1H, major isomer), 4.63 (d, J = 11.4 Hz, 1H, minor isomer), 4.58 (d, J = 11.4 Hz, 1H, major isomer), 4.54 (s, 2H, minor isomer), 4.31 (dt, J = 1.7, 7.0 Hz, 1H, major isomer), 4.24 (t, J = 11.6 Hz, 1H, minor isomer), 4.08-4.04 (m, 1H, both isomers), 4.00-3.97 (m, 1H, minor isomer), 3.89 (dd, J = 7.0, 9.9 Hz, 1H, major isomer), 2.33 (sep, J = 6.7 Hz, 1H, both isomers) 2.11 (s, 3H, major isomer), 2.10 (s, 3H, minor isomer), 1.00 (d, J = 6.7 Hz, 6H, major isomer),

0.97 (d, J = 6.7 Hz, 6H, minor isomer); ^{13}C NMR (100 MHz, $CDCl_3$): δ 169.6, 169.5, 153.2, 153.1, 144.3, 144.1, 138.1, 138.0, 137.9, 137.6, 128.5, 128.4, 128.4, 128.3, 128.1, 128.0, 127.9, 127.8, 127.7, 127.6, 105.9, 105.8, 100.4, 99.9, 86.5, 85.8, 82.2, 80.6, 77.5, 77.2, 76.8, 75.3, 74.5, 72.9, 72.8, 72.1, 71.0, 70.4, 64.3, 61.9, 31.7, 21.6, 20.9; HRMS (ESI) calcd. for $[C_{29}H_{33}O_5]^+$, 461.2328; found 461.2324.

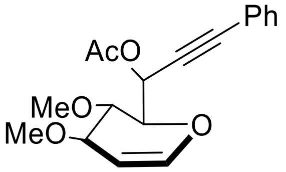

1,5-anhydro-2-deoxy-3,4-bis-*O*-methyl-6-(1-*O*-acetyl-3-phenylprop-2-yn-1-yl)-D-arabino-Hex-1-enitol (1n): Compound was prepared following the general procedure **B**, **8b** (212.4 mg, 1.2 mmol), acyl chloride (124.8 mg, 1.6 mmol), **1n** was obtained (254.9 mg, 65%, 3 steps) as a 1:1.1 mixture of diastereomers about the propargylic position after flash chromatography on silica (8:1, *n*-Hexane/EtOAc). ^{1}H NMR (300 MHz, $CDCl_3$): δ 7.48-7.44 (m, 2H, both isomers), 7.36-7.27 (m, 3H, both isomers), 6.46-6.44 (dd, J = 1.1, 6.1 Hz, 1H, minor isomer), 6.38 (d, J = 6.2 Hz, 1H, major isomer), 4.99-4.96 (dd, J = 3.7, 6.2 Hz, 1H, major isomer), 4.88 (dd, J = 2.3, 6.1 Hz, 1H, minor isomer), 4.26 (dt, J = 1.1, 5.9 Hz, 1H, major isomer), 4.05 (dd, J = 3.1, 9.8 Hz, 1H, minor isomer), 4.00 (dt, J = 3.7, 6.9 Hz, 1H, major isomer), 3.80-3.72 (m, 1H, both isomers), 3.64 (s, 3H, minor isomer), 3,61 (dd, J = 6.9, 9.8 Hz, 1H, major isomer), 3.51 (s, 3H, major isomer), 3.42 (s, 3H, both isomers), 2.16 (s, 3H, minor isomer), 2.15 (s, 3H, major isomer); ^{13}C NMR (75 MHz, $CDCl_3$): δ 169.5, 169.5, 144.3, 14308, 132.0, 131.9, 128.8, 128.3, 128.2, 121.9, 121.9, 100.1, 99.7, 87.2, 86.7, 83.8, 82.3, 78.1, 77.2, 76.9, 76.3, 74.5, 73.8, 64.0, 61.6, 60.1, 58.6, 56.0, 55.9, 20.9.

20.9; HRMS (ESI) calcd. for $[C_{18}H_{21}O_5]^+$, 317.1389; found 317.1392.

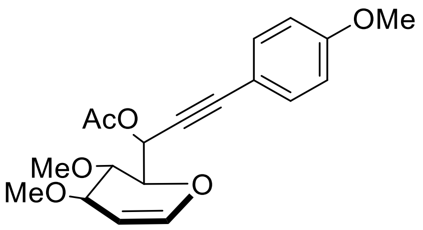

1,5-anhydro-2-deoxy-3,4-bis-*O*-methyl-6-(1-*O*-acetyl-3-(*p*-methoxyphenyl)prop-2-yn-1-yl)-D-arabino-Hex-1-enitol (1o): Compound was prepared following the general procedure **B**, **8b** (212.4 mg, 1.2 mmol), acyl chloride (124.8 mg, 1.6 mmol), 4-ethynylanisole (458.2 mg, 3.45 mmol), **1o** was obtained (274.2 mg, 61%, 3 steps) as a 1:3 mixture of diastereomers about the propargylic position after flash chromatography on silica (8:1, *n*-Hexane/EtOAc). ^{1}H NMR (300 MHz, $CDCl_3$): δ 7.42-7.38 (m, 2H, both isomers), 6.84-6.81 (m, 2H, both isomers), 6.45 (d, J = 6.0 Hz, 1H, major isomer), 6.38 (d, J = 6.2 Hz, minor isomer), 6.12 (d, J = 6.2 Hz, 1H, minor isomer), 6.08 (d, J = 3.0 Hz, 1H, major isomer), 4.97 (dd, J = 3.8, 6.1 Hz, 1H, minor isomer), 4.87 (dd, J = 2.2, 6.1 Hz, 1H, major isomer), 4.24 (t, J = 5.9 Hz, minor isomer), 4.03 (dd, J = 3.0, 9.8 Hz, 1H, major isomer), 4.00 (dt, J = 1.6, 6.9 Hz, 1H, major isomer), 3.80 (s, 3H, both isomers), 3.79 (t, J = 4.0 Hz, 1H, minor isomer), 3.73 (t, J = 5.1 Hz, 1H, minor isomer), 3.63 (s, 3H, major isomer), 3.59 (dd, J = 6.9, 9.8 Hz, 1H, both isomers), 3.51 (s, 3H, minor isomer), 3.42 (s, 3H, both isomers); ^{13}C NMR (100 MHz, CDCl3): δ 169.6, 169.6, 160.0, 144.3, 143.8, 133.6, 133.5, 114.0, 114.0, 113.9, 113.9, 100.1, 99.7, 87.4, 86.8. 82.4, 80.9, 78.2, 77.3, 76.9, 76.4, 74.5, 73.9, 64.2, 61.8, 60.2, 58.6, 56.1, 56.0, 55.3, 21.0, 21.0; HRMS (ESI) calcd. for $[C_{19}H_{22}O_6Na]^+$, 369.1314; found 369.1310.

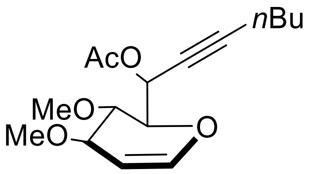

1,5-anhydro-2-deoxy-6-(1-*O*-acetylhept-2-yn-1-yl)-3,4-bis-*O*-methyl-D-arabino-Hex-1-enitol (1p): Compound was prepared following the general procedure **B**, **8b** (212.4 mg, 1.2 mmol), acyl chloride (124.8 mg, 1.6 mmol), 1-hexyne (283.2 mg, 3.45 mmol), **1p** was obtained (244.9 mg, 63%, 3 steps) as a 1:1.3 mixture of diastereomers about the propargylic position after flash chromatography on silica (8:1, *n*-Hexane/EtOAc). ^{1}H NMR (400 MHz, $CDCl_3$): δ 6.36 (d, J = 6.0 Hz, 1H, major isomer), 6.32 (d, J = 6.2 Hz, 1H, minor isomer), 5.84 (dt, J = 1.9, 6.1 Hz, 1H, minor isomer), 5.80 (d, J = 3.4 Hz, 1H, major isomer), 4.91 (dd, J = 3.7, 9.9 Hz, 1H, minor isomer), 4.82 (dd, J = 2.2, 8.2 Hz, 1H, major isomer), 4.04 (t, J = 6.0 Hz, 1H, minor isomer), 3,94 (d, J = 6.9 Hz, 1H. major isomer), 3.89 (dd, J = 2.9, 9.9 Hz, 1H, major isomer), 3.73 (t, J = 4.0 Hz, 1H, minor isomer), 3.66-3.61 (m, 1H, both isomers), 3.57 (s, 3H, major isomer), 3.49-3.32 (m, 1H, both isomers), 3.45 (s, 3H, minor isomer), 3.38 (s, 3H, major isomer), 3.37 (s, 3H, minor isomer), 2.23-2.19 (m, 2H, both isomers), 2.09 (s, 3H, major isomer), 2.08 (s, 3H, minor isomer), 1.51-1.44 (m, 2H, both isomers), 1.42-1.34 (m, 2H, both isomers), 0.89-0.85 (m, 3H, both isomers) ; ^{13}C NMR (100 MHz, $CDCl_3$): δ 1695, 144.2, 143.8, 99.9, 99.4, 88.5, 87.9, 78.2, 77.2, 76.7, 76.4, 74.8, 74.5, 73.9, 73.1, 63.9, 61.4, 60.0, 58.5, 55.9, 55.8, 30.3, 21.8, 20.9, 20.8, 18.4, 13.4; HRMS (ESI) calcd. for $[C_{16}H_{24}O_5Na]^+$, 319.1521; found 319.1522.

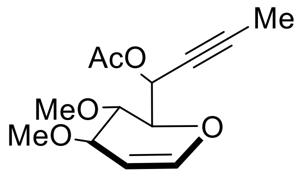

1,5-anhydro-6-(1-*O*-acetylbut-2-yn-1-yl)-2-deoxy-3,4-bis-*O*-methyl-D-arabino-Hex-1-enitol (1q): Compound was prepared following the general procedure **B**, **8b** (212.4 mg, 1.2 mmol), acyl chloride (124.8 mg, 1.6 mmol), 1-propynylmagnesium bromide solution (0.5 M in THF, 6.9 mL, 3.45 mmol), **1q** was obtained (178.3 mg, 58%, 3 steps) as a 1:1.7 mixture of diastereomers about the propargylic position after flash chromatography on silica (8:1, *n*-Hexane/EtOAc). ^{1}H NMR (400 MHz, $CDCl_3$): δ 6.41 (d, J = 4.8 Hz, 1H, major isomer), 6.35 (d, J = 6.2 Hz, 1H, minor isomer), 5.82 (dd, J = 2.3, 5.6 Hz, 1H, minor isomer), 5.79 (t, J = 2.4 Hz, 1H, major isomer), 4.92 (dd, J = 3.6, 6.2 Hz, 1H, minor isomer), 4.85 (dd, J = 2.2, 6.1 Hz, 1H, major isomer), 4.06 (t, J = 6.0 Hz, 1H, minor isomer), 3.95 (dt, J = 1.6, 6.8 Hz, 1H, major isomer), 3.91 (dd, J = 3.0, 9.9 Hz, 1H, major isomer), 3.78 (t, J = 3.9 Hz, 1H, minor isomer, 3.60 (t, J = 6.0 Hz, 1H, minor isomer), 3.58 (s, 3H, major isomer), 3.50 (dd, J = 6.8, 9.8 Hz, 1H, major isomer), 3.47 (s, 3H, minor isomer), 3.40 (s, 3H, major isomer), 3.39 (s, 3H, minor isomer), 2.11 (s, 3H, major isomer), 2.11 (s, 3H, minor isomer), 1.87 (s, 3H, minor isomer), 1.87 (s, 3H, major isomer); ^{13}C NMR (100 MHz, $CDCl_3$): δ 169.6, 169.6, 144.2, 143.9, 99.9, 99.5, 84.0, 83.4, 78.1, 77.1, 76.7, 76.6, 74.6, 74.5, 73.9, 72.4, 63.9, 61.5, 60.0, 58.7, 60.0, 55.9, 20.9, 20.9, 3.7; HRMS (ESI) calcd. for $[C_{13}H_{18}O_5Na]^+$, 277.1052; found 277.1056.

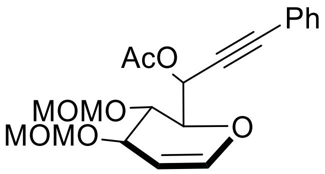

1,5-anhydro-2-deoxy-3,4-bis-*O*-methoxymethyl-6-(1-*O*-acetyl-3-phenylprop-2-yn-1-yl)-D-arabino-Hex-1-enitol (1r): Compound was prepared following the general procedure **B**, **8c** (280.8 mg, 1.2 mmol), acyl chloride (124.8 mg, 1.6 mmol), **1r** was obtained (301.9 mg, 63%, 3 steps) as a 1:2.4 mixture of diastereomers about the propargylic position after flash chromatography on silica (8:1, *n*-Hexane/EtOAc). ^{1}H NMR (400 MHz, $CDCl_3$): δ 7.48-7,43 (m, 2H, both isomers), 7.35-7.27 (m, 3H, both isomers), 6.44 (dd, J = 1.3, 7.4 Hz, 1H, minor isomer), 6.40 (dd, J = 0.8, 7.0 Hz, 1H, major isomer), 6.12 (d, J = 2.9 Hz, major isomer), 6.10 (s, 1H, minor isomer), 5.01-4.96 (m, 1H, both isomers), 4.91 (dd, J = 2.6, 6.1 Hz, 1H, minor isomer), 4.83-4.69 (m, 4H, both isomers), 4.34 (dd, J = 1.3, 7.4 Hz, 1H, major isomer), 4.29-4.24 (m, 1H, both isomers), 4.16 (dd, J = 3.8, 8.9 Hz, 1H, minor isomer), 4.10 (t, J = 4.0 Hz, 1H, major isomer), 4.01 (dd, J = 6.4, 9.0 Hz, 1H, minor isomer), 3.47 (s, 3H, minor isomer), 3.40 (s, 3H, major isomer), 3.38 (s, 3H, both isomers), 2.16 (s, 3H, minor isomer), 2.15 (3H, major isomer); ^{13}C NMR (100 MHz, $CDCl_3$): δ 169.6, 169.4, 144.0, 143.7, 132.0, 131.8, 128.9, 128.7, 128.3, 128.2, 121.9, 121.8, 101.2, 100.1, 97.1, 96.3, 95.8, 94.8, 87.1, 86.9, 83.6, 82.4, 77.2, 76.9, 74.2, 72.8, 71.2, 68.3, 63.7, 61.6, 56.3, 56.0, 55.6, 55.5, 20.9, 20.9; HRMS (ESI) calcd. for $[C_{20}H_{24}O_7Na]^+$, 399.1420; found 399.1425.

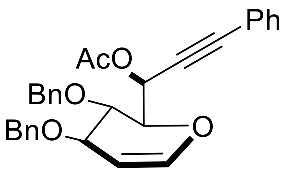

A / B

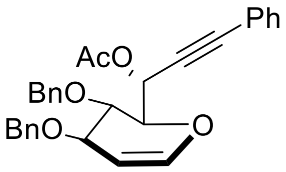

A / B

1,5-anhydro-3,4-bis-*O*-benzyl-2-deoxy-6-(1-*O*-acetyl-3-phenylprop-2-yn-1-yl)-D-arabino-hex-5-enitol (*epi*-1a-A): Compound was prepared following the general procedure **B**, **8d** (400.0 mg, 1.2 mmol), acyl chloride (124.8 mg, 1.6 mmol), ***epi*-1a-A** was obtained (195.9 mg, 32%, 3 steps) after flash chromatography on silica (8:1, *n*-Hexane/EtOAc). Rf 0.45 (4:1 Hexane/EtOAc); $[\alpha]_D^{22}$ = -77.9; (*c* = 1.0, $CHCl_3$); ^1H NMR (400 MHz, $CDCl_3$): δ 7.45-7.27 (m, 15H), 6.46 (dd, *J* = 1.3, 7.5 Hz, 1H), 5.81 (d, *J* = 8.6 Hz, 1H), 4.98 (d, *J* = 11.4 Hz, 1H), 4.94 (d, *J* = 6.3 Hz, 1H), 4.75-4.70 (m, 2H), 4.64 (d, *J* = 12.0 Hz), 4.37 (br, 1H), 4.11 (d, *J* = 8.6 Hz, 1H), 4.03 (br, 1H), 1.97 (s, 3H); ^{13}C NMR (100 MHz, CDCl3): δ 168.8, 144.1, 138.2, 138.2, 132.1, 128.7, 128.5, 128.4, 128.2, 127.7, 127.5, 122.2, 100.6, 86.0, 84.6, 76.8, 74.0, 73.0, 71.0, 68.3, 62.6, 20.9; HRMS (ESI) calcd. for $[C_{30}H_{28}O_5Na]^+$, 491.1834; found 491.1833.

1,5-anhydro-3,4-bis-*O*-benzyl-2-deoxy-6-(1-*O*-acetyl-3-phenylprop-2-yn-1-yl)-D-arabino-hex-5-enitol (*epi*-1a-B): Compound was prepared following the general procedure **B**, **8d** (400.0 mg, 1.2 mmol), acyl chloride (124.8 mg, 1.6 mmol), ***epi*-1a-B** was obtained (193.2 mg, 30%, 3 steps) after flash chromatography on silica (8:1, *n*-Hexane/EtOAc). Rf 0.40 (4:1 Hexane/EtOAc); $[\alpha]_D^{22}$ = -30.6; (*c* = 1.0, $CHCl_3$); ^1H NMR (400 MHz, $CDCl_3$): δ 7.46-7.28 (m, 15H), 6.42 (dd, *J* = 1.3, 6.2 Hz, 1H), 6.08 (d, *J* = 9.2 Hz, 1H), 5.09 (d, *J* = 11.4 Hz, 1H), 4.95 (d, *J* = 6.3 Hz, 1H), 4.82 (d, *J* = 11.3 Hz, 1H), 4.74 (d, *J* = 12.1 Hz, 1H), 4.67 (d, *J* = 12.1 Hz, 1H), 4.42 (br, 1H), 4.36

(br, 1H), 4.18 (d, J = 9.2 Hz, 1H), 2.17 (s, 3H); ^{13}C NMR (100 MHz, $CDCl_3$): δ 169.6, 143.9, 138.3, 138.2, 131.9, 129.0, 128.4, 128.3, 128.3, 128.0, 127.6, 127.6, 127.4, 121.7, 100.4, 87.4, 83.1, 77.5, 74.7, 73.0, 71.0, 70.8, 64.7, 21.0; HRMS (ESI) calcd. for $[C_{30}H_{28}O_5Na]^+$, 491.1834; found 491.1830.

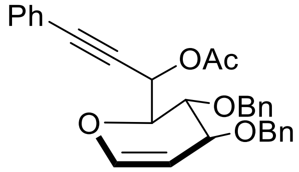

1-((1S,2R,5S)-2(benzyloxyl-8-oxabicyclo[3.2.1]oct-3,6-dien-6-yl)-1-phenyl-methanone (*ent*-1a): Compound was prepared following the general procedure **B**, **8e** (400.0 mg, 1.2 mmol), acyl chloride (124.8 mg, 1.6 mmol), ***ent*-1a** was obtained (382.1 mg, 64%, 3 steps) as a 1:1.1 mixture of diastereomers about the propargylic position after flash chromatography on silica (8:1, *n*-Hexane/EtOAc). ^{1}H NMR (400 MHz, $CDCl_3$): δ 7.47-7.27 (m, 15H, both isomers), 6.50 (dd, J = 1.6, 6.1 Hz, 1H, minor isomer), 6.46 (d, J = 6.4 Hz, 1H, major isomer), 6.25 (d, J = 6.2 Hz, 1H, minor isomer), 6.22 (d, J = 2.8 Hz, 1H, minor isomer), 5.05 (ddd, J = 0.6, 3.6, 6.2 Hz, 1H, major isomer), 5.01 (d, J = 11.1 Hz, 1H, minor isomer), 4.96 (dd, J = 2.1, 6.1 Hz, 1H, minor isomer), 4.86 (d, J = 11.0 Hz, 1H, minor isomer) 4.81 (d, J = 11.6 Hz, 1H, major isomer), 4.71 (d, J = 11.6 Hz, 1H, major isomer), 4.67 (d, J = 11.6 Hz, 1H, major isomer), 4.61 (d, J = 11.6 Hz, 1H, major isomer), 4.58 (s, 2H, minor isomer), 4.38 (dt, J = 1.7, 7.0 Hz, 1H, minor isomer), 4.35 (dt, J = 1.2, 5.8 Hz, 1H, major isomer), 4.17 (dd, J = 2.9, 9,9 Hz, 1H, minor isomer), 4.11-4.07 (m, 1H, both isomers), 4.00 (dd, J = 7.0, 10.0 Hz, 1H, minor isomer), 2.16 (s, 3H, minor isomer), 2.14 (s, 3H, major isomer); ^{13}C NMR (100

MHz, $CDCl_3$): δ 169.5, 169.4, 144.2, 144.0, 138.0, 137.9, 137.8, 137.5, 132.0, 131.9, 128.8, 128.7, 128.5, 128.5, 128.4, 128.3, 128.2, 128.2, 128.1, 128.0, 127.9, 127.8, 127.8, 127.7, 127.6, 121.8, 121.8, 100.5, 99.9, 87.3, 86.7, 83.7, 82.1, 77.4, 76.9, 75.3, 74.5, 72.5, 72.1, 71.8, 70.8, 70.3, 66.2, 64.1, 61.7, 20.9; HRMS (ESI) calcd. for $[C_{30}H_{28}O_5Na]^+$, 491.1834; found 491.1830.

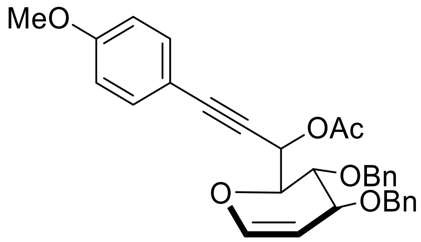

1-((1S,2R,5S)-2-benzyloxyl-8-oxabicyclo[3.2.1]oct-3,6-dien-6-yl)-1-(4-methoxyphenyl)-methanone (*ent*-1b): Compound was prepared following the general procedure **B**, **8e** (400.0 mg, 1.2 mmol), acyl chloride (124.8 mg, 1.6 mmol), 4-ethynylanisole (458.2 mg, 3.45 mmol), ***ent*-1b** was obtained (422.1 mg, 66%, 3 steps) as a 1:1.3 mixture of diastereomers about the propargylic position after flash chromatography on silica (8:1, *n*-Hexane/EtOAc). ^1H NMR (500 MHz, $CDCl_3$): δ 7.38-7.24 (m, 12H, both isomers), 6.82-6.79 (m, 2H, both isomers), 6.47 (dd, J = 1.1, 4.8 Hz, 1H, major isomer), 6.43 (d, J = 6.2 Hz, 1H, minor isomer), 6.21 (d, J = 6.2 Hz, 1H, minor isomer), 6.17 (d, J = 2.9 Hz, 1H, major isomer), 5.02 (ddd, J = 0.5, 4.2, 6.2 Hz, 1H, minor isomer), 4.97 (d, J = 11.1 Hz, major isomer), 4.93 (dd, J = 2.2, 6.1 Hz, 1H, major isomer), 4.83 (d, J = 11.1 Hz, 1H, major isomer), 4.78 (d, J = 11.6 Hz, 1H, minor isomer), 4.68 (d, J = 11.6 Hz, 1H, minor isomer), 4.65 (d, J = 11.6 Hz, minor isomer), 4.59 (d, J = 11.6 Hz, 1H, major isomer), 4.55 (s, 2H, minor isomer); 4.35 (dt,

J = 1.8, 7.0 Hz, 1H, major isomer), 4.31 (td, J = 1.1 Hz, 5.8 Hz, minor isomer), 4.14 (dd, J = 2.9, 10.0 Hz, 1H, major isomer), 4.09-4.05 (m, 2H, minor isomer), 3.97 (dd, J = 7.0, 9.9 Hz, 1H, major isomer), 3.81 (s, 3H, minor isomer), 3.81 (s, 3H, major isomer), 2.15 (s, 3H, major isomer), 2.12 (s, 3H, minor isomer); ^{13}C NMR (100 MHz, $CDCl_3$): δ 169.6, 169.5, 160.0, 159.9. 144.3, 144.0, 138.0, 138.0, 137.8, 137.5, 133.6, 133.4, 128.5, 128.4, 128.4, 128.4, 128.2, 128.0, 128.0, 127.8, 127.8, 127.8, 128.6, 128.6, 113.9, 113.9, 113.8, 100.5, 99.9, 87.4, 86.8, 82.4, 80.8, 77.5, 77.0, 76.8, 75.5, 74.6, 72.6, 72.2, 71.9, 70.9, 70.3, 64.3, 61.9, 55.2, 21.0, 20.9; HRMS (ESI) calcd. for $[C_{31}H_{20}O_6Na]^+$, 521.1940; found 521.1940.

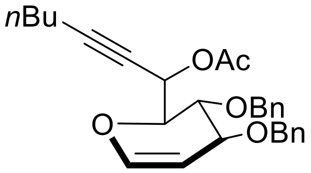

1,5-anhydro-3,4-bis-*O*-benzyl-2-deoxy-6-(1-*O*-acetylhept-2-yn-1-yl)-L-arabino-hex-1-enitol (*ent*-1g): Compound was prepared following the general procedure **B**, **8e** (400.0 mg, 1.2 mmol), acyl chloride (124.8 mg, 1.6 mmol), 1-hexyne (283.2 mg, 3.45 mmol), ***ent*-1g** was obtained (352.5 mg, 61%, 3 steps) as a 1:1.7 mixture of diastereomers about the propargylic position after flash chromatography on silica (8:1, *n*-Hexane/EtOAc).^{1}H NMR (500 MHz, $CDCl_3$): δ 7.37-7.29 (m, 10H, both isomers), 6.45 (dd, J = 0.84, 6.1 Hz, 1H, major isomer), 6.42 (d, J = 6.3 Hz, 1H, minor isomer), 5.98 (dt, J = 2.0, 5.6 Hz, 1H, minor isomer), 5.96-5.54 (m, 1H, major isomer), 4.98 (dd, J = 3.6, 6.2 Hz, 1H, minor isomer), 4.67 (d, J = 11.1 Hz, 1H, major isomer), 4.91 (dd, J = 2.1, 6.1 Hz, 1H, major isomer), 4.80 (d, J = 11.0 Hz, 1H, major isomer), 4.78

(d, *J* = 11.4 Hz, 1H, minor isomer), 4.69 (d, *J* = 11.6 Hz, 1H, major isomer), 4.63 (d, *J* = 11.5 Hz, 1H, minor isomer), 4.59 (d, *J* = 11.6 Hz, 1H, major isomer), 4.56 (s, 2H, minor isomer), 4.33 (dt, *J* = 1.7, 7.0 Hz, 1H, major isomer), 4.17 (t, *J* = 5.8 Hz,1H, minor isomer), 4.17 (t, *J* = 5.8 Hz, 1H, minor isomer), 4.09-4.07 (m, 1H, minor isomer), 4.04 (dd, *J* = 2.8, 10.0 Hz, 1H, major isomer), 4.00-3.97 (m, 1H, minor isomer), 3.90 (dd, *J* = 7.0, 10.0 Hz, 1H, major isomer), 2.22 (td, *J* = 2.0, 7.0 Hz, 2H, major isomer), 2.18-2.14 (td, *J* = 1.9, 7.1 Hz, 1H, minor isomer), 2.11 (s, 3H, major isomer), 2.09 (s, 3H, major isomer), 1.53-1.27 (m, 4H, both isomers), 0.92-0.86 (m, 3H, both isomers); ^{13}C NMR (100 MHz, $CDCl_3$): δ 169.6, 169.5, 144.3, 144.0, 138.0, 138.0, 137.9, 137.6, 128.4, 128.4, 128.4, 128.4, 128.2, 128.0, 127.9, 127.8, 127.8, 127.7, 127.7, 127.6, 127.6, 100.4, 99.8, 88.7, 87.9, 77.4, 77.0, 75.4, 74.7, 74.5, 73.1, 72.8, 72.7, 72.2, 70.8, 70.3, 64.1, 61.3, 30.4, 30.3, 21.9, 21.9, 20.9, 20.9, 18.5, 18.4, 13.5; HRMS (ESI) calcd. for $[C_{28}H_{32}O_5Na]^+$, 471.2147; found 471.2144.

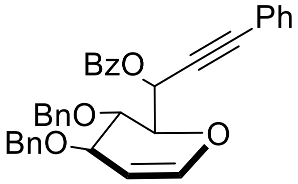

1,5-anhydro-3,4-bis-*O*-benzyl-2-deoxy-6-(1-*O*-benzoyl-3-phenylprop-2-yn-1-yl)-D-arabino-hex-1-enitol (1s): Compound was prepared following the general procedure **B**, benzoyl chloride (224.9 mg, 1.6 mmol), **1s** was obtained (413.9 mg, 61%, 3 steps) as a 1:1.1 mixture of diastereomers about the propargylic position after flash chromatography on silica (8:1, *n*-Hexane/EtOAc). ^{1}H NMR (500 MHz, $CDCl_3$): δ 8.13-8.10 (m, 2H, both isomers), 7.61-7.57 (m, 1H, both isomers), 7.48-7.42 (m, 4H,

both isomers), 7.38-7.27 (m, 13H, both isomers), 6.53 (dd, *J* = 1.1, 6.1 Hz, 1H, minor isomer), 6.50-6.48 (m, 1H, major isomer), 6.48 (d, *J* = 6.1 Hz, 1H, major isomer); 6.44 (d, *J* = 3.4 Hz, 1H, minor isomer), 5.06 (dd, *J* = 3.5, 6.2 Hz, 1H, major isomer), 5.03 (d, *J* = 11.2 Hz, 1H, minor isomer), 4.98 (dd, *J* = 2.2, 6.1 Hz, 1H, minor isomer), 4.90 (d, *J* = 11.2 Hz, 1H, minor isomer), 4.81 (d, *J* = 11.5 Hz, 1H, major isomer), 4.70-4.57 (m, 3H, both isomers), 4.47 (td, *J* = 0.9, 5.9 Hz, 1H, major isomer), 4.36 (dt, *J* = 1.8, 4.8 Hz, 1H, minor isomer), 4.34 (dd, *J* = 3.4, 9.4 Hz, 1H, minor isomer), 4.19-4.17 (m, 1H, major isomer), 4.15-4.13 (m, 1H, major isomer), 4.08 (dd, *J* = 6.6, 9.4 Hz, 1H, minor isomer) ; ^{13}C NMR (100 MHz, $CDCl_3$): 165.3, 165.2, 144.4, 144.2, 138.1, 138.0, 137.9, 137.6, 133.3, 133.3, 132.2, 132.0, 130.0, 130.0, 129.7, 129.6, 128.8, 128.5, 128.5, 128.5, 128.4, 128.3, 128.2, 128.2, 128.1, 128.0, 127.9, 127.8, 127.7, 122.0, 121.9, 100.5, 100.1, 87.4, 87.0, 83.9, 82.6, 77.5, 76.5, 75.1, 74.4, 72.9, 72.8, 72.4, 70.9, 70.6, 64.6, 62.5; HRMS (ESI) calcd. for $[C_{35}H_{30}O_5Na]^+$, 553.1991; found 553.1984.

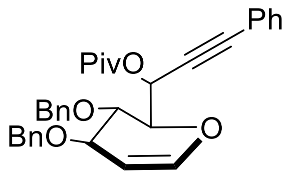

1,5-anhydro-3,4-bis-*O*-benzyl-2-deoxy-6-(1-*O*-pivaloyl-3-phenylprop-2-yn-1-yl)-D-arabino-hex-1-enitol (1t): Compound was prepared following the general procedure **B**, pivaloyl chloride (193.0 mg, 1.6 mmol), **1t** was obtained (418.5 mg, 64%, 3 steps) as a 1:1.2 mixture of diastereomers about the propargylic position after flash chromatography on silica (8:1, *n*-Hexane/EtOAc). ^{1}H NMR (500 MHz, $CDCl_3$): δ

7.42-7.27 (m, 15H, both isomers), 6.48 (d, *J* = 6.1 Hz, 1H, minor isomer), 6.42 (d, *J* = 6.2 Hz, 1H, major isomer), 6.26 (d, *J* = 6.6 Hz, 1H, major isomer), 6.16 (d, *J* = 3.3 Hz, 1H, minor isomer), 5.02-5.00 (m, 1H, major isomer), 4.96-4.92 (m, 1H, both isomers), 4.84 (d, *J* = 11.2 Hz, 1H, minor isomer), 4.77 (d, *J* = 11.6 Hz, 1H, major isomer), 4.68 (d, *J* = 11.6 Hz, 1H, major isomer), 4.63 (d, *J* = 11.6 Hz, 1H, major isomer), 4.58 (d, *J* = 11.7 Hz, 1H, major isomer), 4.58 (s, 2H, minor isomer), 4.35 (t, *J* = 5.8 Hz, 1H, major isomer), 4.31 (d, *J* = 6.5 Hz, 1H, minor isomer), 4.16 (dd, *J* = 3.4, 9.3 Hz, 1H, minor isomer), 4.09 (t, *J* = 4.3 Hz, 1H, major isomer), 4.04-4.03 (m, 1H, major isomer), 3.97 (dd, *J* = 6.7, 9.1 Hz, 1H, minor isomer), 1.26 (s, 9H, major isomer), 1.23 (s, 9H, minor isomer); ^{13}C NMR (100 MHz, CDCl3): δ 177.0, 176.9, 144.4, 144.0, 138.1, 138.0, 137.8, 137.6, 132.1, 131.9, 128.7, 128.5, 128.5, 128.4, 128.4, 128.2, 128.1, 128.1, 128.0, 127.9, 127.8, 127.8, 127.6, 122.1, 122.0, 100.2, 99.8, 86.9, 86.6, 84.0, 82.8, 76.8, 76.2, 75.1, 74.3, 72.6, 72.2 ,71.7, 70.8, 70.3, 63.7, 61.4, 38.9, 38.8, 27.1, 27.0; HRMS (ESI) calcd. for $[C_{33}H_{34}O_5Na]^+$, 533.2304; found 533.2303.

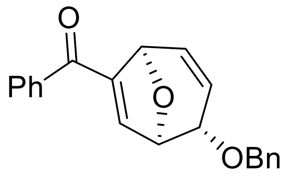

1-((1R,2S,5R)-2-benzyloxyl-8-oxabicyclo[3.2.1]oct-3,6-dien-6-yl)-1-phenyl-methanone (2a): Compound was prepared following the general procedure **C**, **1a** (46.9 mg, 0.1 mmol), **2a** was obtained (25.7 mg, 81%) as colorless oil after flash chromatography on silica (4:1, *n*-Hexane/EtOAc). $[\alpha]_D^{22}$ = 6.9; (*c* = 1.0, $CHCl_3$); ^{1}H NMR (300 MHz, $CDCl_3$): δ 7.81-7.78 (m, 7.81-78 2H), 7.61-7.55 (m 4H), 7.39-7.28

(m, 4H), 6.70 (ddd, J = 1.3, 4.3, 9.6 Hz, 1H), 6.70 (dd, J = 2.2, 9.6 Hz, 1H), 5.58 (ddd, J = 2.1, 3.6, 9.8 Hz, 1H), 5.22 (t, J = 1.9 Hz, 1H), 5.15 (d, J = 4.2 Hz, 1H), 4.71 (s, 2H), 3.60 (d, J = 3.5 Hz, 1H); ^{13}C NMR (100 MHz, $CDCl_3$): δ 190.3, 155.8, 139.1, 138.2, 137.6, 137.2, 133.0, 128.8, 128.6, 128.5, 127.8, 122.9, 84.2, 77.5, 70.0, 68.7; HRMS (ESI) calcd. for $[C_{21}H_{18}O_3Na]^+$ 341.1154; found 341.1147.

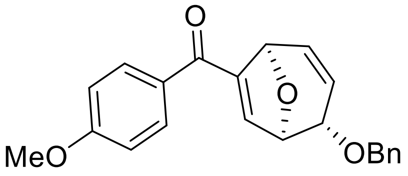

1-((1R,2S,5R)-2-benzyloxyl-8-oxabicyclo[3.2.1]oct-3,6-dien-6-yl)-1-(4-methoxyphenyl)-methanone (2b): Compound was prepared following the general procedure **C**, **1b** (49.8 mg, 0.1 mmol), **2b** was obtained (31.0 mg, 89 %) as a white solid after flash chromatography on silica (4:1, *n*-Hexane/EtOAc). m. p. 101-102 °C; $[\alpha]_D^{22}$ = -64.7; (c = 1.0, $CHCl_3$); ^{1}H NMR (400 MHz, $CDCl_3$): δ 7.84-7.81 (m, 2H), 7.40-7.28 (m, 5H), 6.96-6.93 (m, 2H), 6.71 (ddd, J = 1.3, 4.3, 9.8 Hz, 1H), 6.63 (d, J = 2.2 Hz), 5.58 (ddd, J = 2.2, 3.6, 9.8 Hz, 1H), 5.21 (t, J = 4.0 Hz, 1H), 5.11 (d, J = 9.7 Hz, 1H), 4.71 (s, 2H), 3.88 (s, 3H), 3.61 (dd, J = 0.7, 3.5 Hz, 1H); ^{13}C NMR (100 MHz, $CDCl_3$): δ 188.9, 163.6, 155.8, 138.2, 137.8, 137.5, 131.1, 129.9, 128.5, 128.4, 127.7, 127.0, 122.7, 113.8, 84.1, 77.7, 70.0, 68.9, 55.5; HRMS (ESI) calcd. for $[C_{22}H_{20}O_4Na]^+$ 371.1259; found 371.1257.

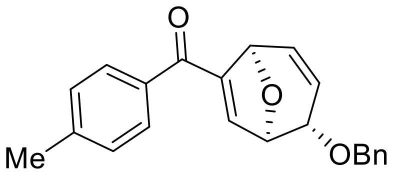

1-((1R,2S,5R)-2-benzyloxyl-8-oxabicyclo[3.2.1]oct-3,6-dien-6-yl)-1-(*p*-tolyl)-methanone (2c): Compound was prepared following the general procedure **C**, **1c** (48.2 mg, 0.1 mmol), **2c** was obtained (27.3 mg, 82%) as colorless oil after flash chromatography on silica (4:1, *n*-Hexane/EtOAc). $[\alpha]_D^{22}$ = -58.3.; (*c* = 1.0, $CHCl_3$); ^{1}H NMR (300 MHz, $CDCl_3$): δ 7.72 (d, *J* = 7.9 Hz, 2H), 7.40-7.30 (m, 5H), 7.26 (d, *J* = 7.9 Hz, 2H), 6.70 (dd, *J* = 1.3, 4.3 Hz, 1H), 6.66 (d, *J* = 2.2 Hz, 1H), 5.58 (ddd, *J* = 2.2, 4.8, 13.0 Hz, 1H), 5.21 (t, *J* = 2.0 Hz, 1H), 5.13 (d, *J* = 4.3 Hz, 1H), 4.71 (s, 2H), 3.60 (dd, *J* = 0.7, 3.2 Hz, 1H); ^{13}C NMR (100 MHz, $CDCl_3$): δ 189.0, 155.8, 143.9, 138.4, 138.2, 137.7, 134.6, 129.2, 128.9, 128.4, 127.7, 127.7, 122.8, 84.1, 77.5, 70.0, 68.8, 21.6; HRMS (ESI) calcd. for $[C_{22}H_{20}O_3Na]^+$ 355.1310; found 355.1309.

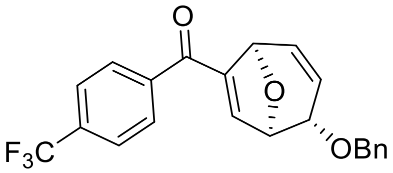

1-((1R,2S,5R)-2-benzyloxyl-8-oxabicyclo[3.2.1]oct-3,6-dien-6-yl)-1-(4-trifluoromethyl-phenyl)-methanone (2d): Compound was prepared following the general procedure **C**, **1d** (53.6 mg, 0.1 mmol), **2d** was obtained (24.3 mg, 63%) as a white solid after flash chromatography on silica (4:1, *n*-Hexane/EtOAc). m. p. 88-90 °C; $[\alpha]_D^{22}$ = -12.1; (*c* = 1.0, $CHCl_3$); ^{1}H NMR (400 MHz, $CDCl_3$): δ 7.89 (d, *J* = 8.1 Hz, 2H), 7.74 (d, *J* = 8.2 Hz, 2H), 7.39-7.27 (m, 5H), 6.73 (d, *J* = 2.2 Hz, 1H), 6.68 (ddd, *J* = 1.1, 4.2, 9.8 Hz, 1H), 5.60 (ddd, *J* = 2.2, 3.5, 9.8 Hz), 5.23 (t, *J* = 2.0 Hz, 1H), 5.17 (d, *J* = 4.2 Hz, 1H), 4.71 (s, 2H), 3.60 (d, *J* = 3.4 Hz); ^{13}C NMR (100 MHz, $CDCl_3$): δ 189.2, 155.6, 140.4, 140.1, 138.0, 137.2, 134.3 (q, $J_{(C-F)}$ = 32.6 Hz), 129.1,

128.5, 127.8, 127.8, 125.7 (q, $J_{(C-F)}$ = 3.7 Hz), 124.5 (q, $J_{(C-F)}$ = 271.6 Hz), 123.2, 84.2, 77.3, 70.1, 68.6; HRMS (ESI) calcd. for $[C_{22}H_{17}O_3F_3Na]^+$ 409.1027; found 409.1027.

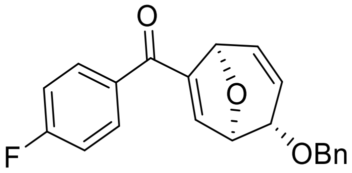

1-((1R,2S,5R)-2-benzyloxyl-8-oxabicyclo[3.2.1]oct-3,6-dien-6-yl)-1-(4-fluorophenyl)-methanone (2e): Compound was prepared following the general procedure **C**, **1e** (48.6 mg, 0.1 mmol), **2e** was obtained (22.5 mg, 67%) as colorless oil after flash chromatography on silica (4:1, *n*-Hexane/EtOAc). $[\alpha]_D^{22}$ = -74.1; (*c* = 1.0, $CHCl_3$); ^{1}H NMR (300 MHz, $CDCl_3$): δ 7.87-7.81 (m, 2H), 7.40-7.28 (m, 5H), 7.17-7.11 (m, 2H), 6.71-6.67 (m, 2H), 5.58 (ddd, *J* = 2.2, 3.6, 9.8 Hz, 1H), 5.21 (t, *J* = 4.2 Hz, 1H), 5.13 (d, *J* = 4.3 Hz, 1H), 4.71 (s, 2H), 3.60 (d, *J* = 3.3 Hz, 1H); ^{13}C NMR (100 MHz, $CDCl_3$): 188.8, 167.0, 164.4, 155.6, 138.9, 138.1, 137.5, 133.5, 133.4, 131.4, 131.3, 128.5, 127.8, 122.9, 115.9, 115.6, 84.2, 77.5, 70.0, 68.7; HRMS (ESI) calcd. for $[C_{21}H_{17}FO_5Na]^+$, 359.1059; found 359.1069.

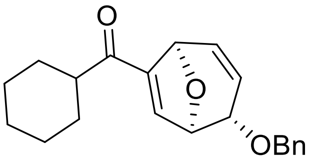

1-((1R,2S,5R)-2-benzyloxyl-8-oxabicyclo[3.2.1]oct-3,6-dien-6-yl)-1-cyclohexyl-methanone (2f): Compound was prepared following the general procedure **C**, **1f** (47.4 mg, 0.1 mmol), **2f** was obtained (25.3 mg, 78%) as a white solid after flash chromatography on silica (4:1, *n*-Hexane/EtOAc). m. p. 84-86 ^{o}C; $[\alpha]_D^{22}$ = 52.1 (*c* = 1.0, $CHCl_3$); ^{1}H NMR (400 MHz, $CDCl_3$): δ 7.39-7.27 (m, 5H), 6.81 (d, *J* = 2.3 Hz,

1H), 6.54 (dd, J = 1.3, 9.8 Hz, 1H), 5.53 (ddd, J = 2.1, 4.3, 9.8 Hz), 5.13 (t, J = 2.0 Hz, 1H), 5.01 (d, J = 4.3 Hz, 1H), 4.70 (s, 2H), 3.61 (dd, J = 0.7, 4.2 Hz, 1H), 2.82-2.76 (m, 2H), 1.82-1.68 (m, 5H), 1.48-1.17 (m, 6H); ^{13}C NMR (100 MHz, $CDCl_3$): δ 199.9, 156.1, 138.2, 137.3, 136.0. 128.5, 127.7. 123.0, 83.7, 76.3, 69.9, 69.0, 47.4, 29.7, 28.6, 25.8, 25.7, 25.5; HRMS (ESI) calcd. for $[C_{21}H_{24}O_3Na]^+$ 347.1623; found 347.1622.

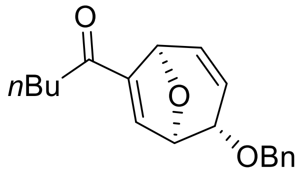

1-((1R,2S,5R)-2-benzyloxyl-8-oxabicyclo[3.2.1]oct-3,6-dien-6-yl)pentan-1-one (2g): Compound was prepared following the general procedure **C**, **1g** (44.8 mg, 0.1 mmol), **2g** was obtained (21.5 mg, 72%) as colorless oil after flash chromatography on silica (4:1, *n*-Hexane/EtOAc). $[\alpha]_D^{22}$ = 65.7; (c = 1.0, $CHCl_3$); ^{1}H NMR (300 MHz, $CDCl_3$): δ 7.40-7.27 (m, 5H), 6.81 (d, J = 2.3 Hz, 1H), 6.54 (ddd, J = 1.4, 4.3, 9.8 Hz, 1H), 5.54 (ddd, J = 2.1, 3.6, 9.8 Hz, 1H), 5.12 (t, J = 2.0 Hz, 1H), 5.02 (d, J = 4.3 Hz, 1H), 4.70 (s, 2H), 3.61 (dd, J = 0.87, 3.5 Hz, 1H), 2.64 (td, J = 0.8, 7.1 Hz, 2H), 1.65-1.55 (m, 2H), 1.39-1.26 (m, 2H), 0.91 (t, J = 9.3 Hz, 3H); ^{13}C NMR (100 MHz, $CDCl_3$): δ 196.6, 157.1, 138.2, 137.2, 136.5, 128.4, 127.7, 123.1, 83.7, 76.2, 69.9, 69.0, 39.0, 26.3, 22.3, 13.8; HRMS (ESI) calcd. for $[C_{19}H_{22}O_3Na]^+$ 321.1467; found 321.1470.

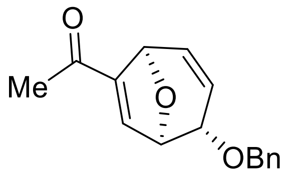

1-((1R,2S,5R)-2-benzyloxyl-8-oxabicyclo[3.2.1]octa-3,6-dien-6-yl)ethan-1-one (2h): Compound was prepared following the general procedure **C**, **1h** (40.6 mg, 0.1 mmol), **2h** was obtained (17.8 mg, 69%) as colorless oil after flash chromatography on silica (4:1, *n*-Hexane/EtOAc). $[\alpha]_D^{22} = 59.5$; (c = 6.8, $CHCl_3$); ^1H NMR (400 MHz, $CDCl_3$): δ 7.39-7.28 (m, 5H), 6.84 (d, J = 2.3 Hz, 1H), 6.54 (ddd, J = 1.4, 4.3, 9.8 Hz, 1H), 5.55 (ddd, J = 2.1, 3.6, 9.8 Hz, 1H), 5.13 (t, J = 4.1 Hz, 1H), 5.03 (d, J = 4.2 Hz, 1H), 4.70 (s, 2H), 3.62 (dd, J = 0.8, 3,6 Hz, 1H), 2.33 (s, 3H); ^{13}C NMR (100 MHz, $CDCl_3$): δ 193.8, 157.4, 138.2, 137.8, 137.1, 128.5, 127.8, 127.8, 123.2, 83.7, 76.0, 70.0, 69.0, 26.8; HRMS (ESI) calcd. for $[C_{16}H_{16}O_3Na]^+$, 279.0997; found 279.0995.

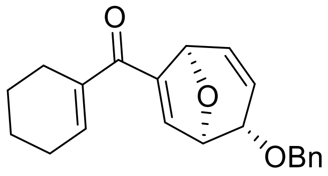

1-((1R,2S,5R)-2-benzyloxyl-8-oxabicyclo[3.2.1]oct-3,6-dien-6-yl)-1-(cyclohex-1-en -1-yl)-methanone (2i): Compound was prepared following the general procedure **C**, **1i** (47.2 mg, 0.1 mmol), **2i** was obtained (20.9 mg, 65%) as a white solid after flash chromatography on silica (4:1, *n*-Hexane/EtOAc). m. p. 113-115 °C; $[\alpha]_D^{22} = -15.7$ (c = 1.0, $CHCl_3$); ^1H NMR (300 MHz, $CDCl_3$): δ 7.39-7.28 (m, 10H), 6.82-6.80 (m, 1H), 6.64 (ddd, J = 1.0, 4.2, 9.8 Hz, 1H), 6.50 (d, J = 2.1 Hz, 1H), 5.54 (ddd, J = 2.2, 3.5, 9.8 Hz, 1H), 5.13 (t, J = 2.0 Hz, 1H), 4.95 (d, J = 4.2 Hz, 1H), 4.70 (s, 2H), 3.59 (d, J = 3.3 Hz, 1H), 2.48-2.43 (m, 1H), 2.27-2.24 (m 2H), 2.11-2.06 (m, 1H), 1.70-1.62 (m, 4H); ^{13}C NMR (100 MHz, $CDCl_3$): 191.6, 155.3, 142.2, 139.0, 138.3, 138.1, 135.6, 128.4, 127.8, 127.7, 122.5, 83.9, 77.7, 69.9, 68.9, 26.1, 23.3, 21.8, 21.6; HRMS (ESI)

calcd. for $[C_{21}H_{23}O_3]^+$, 323.1647; found 323.1642.

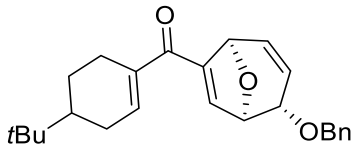

1-((1R,2S,5R)-2-benzyloxyl-8-oxabicyclo[3.2.1]oct-3,6-dien-6-yl)-1-(4-*t*Bu-cyclohex-1-en-1-yl)-methanone (2j): Compound was prepared following the general procedure **C**, **1j** (52.8 mg, 0.1 mmol), **2i** was obtained (22.5 mg, 62%) as a white solid after flash chromatography on silica (4:1, *n*-Hexane/EtOAc). ^{1}H NMR (300 MHz, $CDCl_3$): δ 7.38–7.28 (m, 5H), 6.82 (s, 1H), 6.68–6.60 (dd, J = 4.0, 9.7 Hz,1H), 6.50 (d, J = 2.3 Hz, 1H), 5.54 (dt, J = 10.0, 2.7 Hz, 1H), 5.13 (d, J = 2.3 Hz, 1H), 4.95 (d, J = 4.2 Hz, 1H), 4.70 (s, 2H), 3.59 (d, J = 3.7 Hz, 1H), 2.37-2.27 (m, 2H), 2.11-1.93 (m, 2H), 1.33-1.25 (m, 2H), 1.19-1.09 (m, 1H), 0.89 (s, 9H); ^{13}C NMR (100 MHz, $CDCl_3$): 191.6, 155.2, 142.5, 139.0, 138.3, 138.0, 135.5, 128.5, 127.8, 127.7, 122.5, 83.9, 77.7, 69.9, 68.9, 43.4, 32.2, 27.8, 27.1, 25.4, 23.3; HRMS (ESI) calcd. for $[C_{25}H_{31}O_3]^+$, 379.2273; found 379.2270.

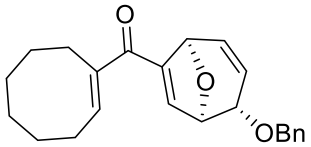

1-((1R,2S,5R)-2-benzyloxyl-8-oxabicyclo[3.2.1]oct-3,6-dien-6-yl)-1-(cycloocten-1-en-1-yl)-methanone (2k): Compound was prepared following the general procedure **C**, **1k** (50.0 mg, 0.1 mmol), **2k** was obtained (24.5 mg, 58%) as a white solid after flash chromatography on silica (4:1, *n*-Hexane/EtOAc). ^{1}H NMR (400 MHz, $CDCl_3$): δ 7.46–7.26 (m, 5H), 6.76 (t, J = 8.3 Hz, 1H), 6.65 (ddd, J = 9.8, 4.3, 1.4 Hz, 1H),

6.45 (d, J = 2.2 Hz, 1H), 5.55 (ddd, J = 9.7, 3.6, 2.1 Hz, 1H), 5.12 (t, J = 2.2 Hz, 1H), 4.95 (d, J = 4.2 Hz, 1H), 4.70 (s, 2H), 3.59 (d, J = 3.7 Hz, 1H), 2.53–2.31 (m, 4H), 1.61-1.60 (m, 4H), 1.51-1.45 (m, 2H); ^{13}C NMR (100 MHz, $CDCl_3$): 192.0, 155.7, 145.1, 142.3, 138.3, 138.0, 135.6, 128.4, 127.7, 127.7, 122.5, 83.9, 77.8, 69.9, 69.0, 29.4, 29.0, 27.4, 26.5, 26.2, 24.7; HRMS (ESI) calcd. for $[C_{23}H_{27}O_3]^+$, 351.1960; found 351.1964.

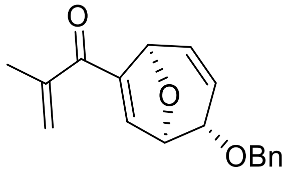

1-((1R,2S,5R)-2-benzyloxyl-8-oxabicyclo[3.2.1]oct-3,6-dien-6-yl)-2-methylprop-2-en-1-one (2l): Compound was prepared following the general procedure **C**, **1l** (43.2 mg, 0.1 mmol), **2l** was obtained (19.7 mg, 70%) as a white solid after flash chromatography on silica (4:1, *n*-Hexane/EtOAc). m. p. 93-95 ^{o}C; $[\alpha]_D^{22}$ = -21.7; (c = 1.0, $CHCl_3$); ^{1}H NMR (500 MHz, $CDCl_3$): δ 7.38-7.27 (m, 5H), 6.63 (dd, J = 1.0, 9.8 Hz, 1H), 6.62 (d, J = 2.2 Hz, 1H), 5.84 (s, 1H), 5.80 (s, 1H), 5.56 (ddd, J = 2.2, 3.5, 9.8 Hz, 1H), 5.14 (t, J = 4.1 Hz, 1H), 4.99 (d, J = 4.3 Hz, 1H), 4.70 (s, 2H), 3.60-3.59 (d, J = 3.4 Hz, 1H), 1.94 (s, 3H); ^{13}C NMR (100 MHz, $CDCl_3$): δ 192.0, 155.3, 144.0, 138.2, 137.7, 137.4, 128.5, 127.8, 125.9, 122.8, 83.9, 77.2, 70.0. 68.8, 17.8; HRMS (ESI) calcd. for $[C_{18}H_{19}O_3]^+$, 283.1334; found 283.1329.

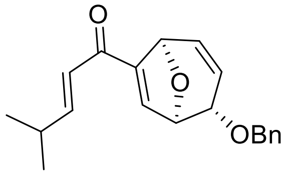

1-((1R,2S,5R)-2-benzyloxyl-8-oxabicyclo[3.2.1]oct-3,6-dien-6-yl)-4-methylpenten-

2-en-1-one (2m): Compound was prepared following the general procedure C, **1m** (46.0 mg, 0.1 mmol), **2m** was obtained (21.7 mg, 70%) as a white solid after flash chromatography on silica (4:1, *n*-Hexane/EtOAc)**.** ^{1}H NMR (400 MHz, $CDCl_3$): δ 7.30–7.27 (m, 5H), 6.93 (dd, J = 15.6, 6.8 Hz, 1H), 6.82 (d, J = 2.3 Hz, 1H), 6.59 (ddd, J = 9.8, 4.3, 1.4 Hz, 1H), 6.42 (dd, J = 15.6, 1.4 Hz, 1H), 5.54 (ddd, J = 9.8, 3.7, 2.1 Hz, 1H), 5.15 (t, J = 2.2 Hz, 1H), 5.06 (d, J = 4.3 Hz, 1H), 4.70 (s, 2H), 3.61 (dd, J = 3.7, 1.4 Hz, 1H), 2.49 (dqd, J = 13.5, 6.8, 1.4 Hz, 1H), 1.08 (d, J = 6.7 Hz, 6H); ^{13}C NMR (100 MHz, $CDCl_3$): 185.9, 157.5, 155.0, 138.2, 137.3, 136.5, 128.4, 127.7, 127.7, 123.0, 123.0, 83.8, 76.5, 69.9, 69.0, 31.3, 21.3, 21.2; HRMS (ESI) calcd. for $[C_{20}H_{23}O_3]^+$, 311.1647; found 311.1643.

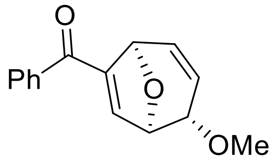

1-((1R,2S,5R)-2-methoxyl-8-oxabicyclo[3.2.1]oct-3,6-dien-6-yl)-1-phenyl-methanone (2n): Compound was prepared following the general procedure **C**, **1n** (31.6 mg, 0.1 mmol), **2n** was obtained (18.9 mg, 78%) as colorless oil after flash chromatography on silica (4:1, *n*-Hexane/EtOAc). $[\alpha]_D^{22}$ = -9.4; (c = 1.0, $CHCl_3$); ^{1}H NMR (300 MHz, $CDCl_3$): δ 7.81-7.78 (m, 2H), 7.60-7.54 (m, 1H), 7.48-7.43 (m, 2H), 6.71 (d, J = 2.2 Hz, 1H), 6.67 (ddd, J = 1.3, 4.3, 9.8 Hz, 1H), 5.54 (ddd, J = 2.2, 3.6, 9.8 Hz, 1H), 5.17 (t, J = 2.0 Hz, 1H), 5.11 (d, J = 4.3 Hz, 1H), 3.45 (s, 3H), 3.39 (dd, J = 0.6, 3.6 Hz, 1H); ^{13}C NMR (75 MHz, $CDCl_3$): δ 190.3, 155.7, 139.2, 137.6, 137.2, 133.0, 128.8, 128.6, 122.6, 83.6, 77.4, 70.6, 55.8; HRMS (ESI) calcd. for $[C_{15}H_{10}O_3Na]^+$, 265.0841; found 265.0842.

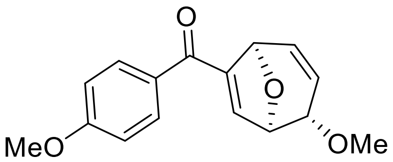

1-((1R,2S,5R)-2-methoxyl-8-oxabicyclo[3.2.1]oct-3,6-dien-6-yl)-1-(4-methoxyphenyl)-methanone (2o): Compound was prepared following the general procedure **C**, **1o** (34.6 mg, 0.1 mmol), **2o** was obtained (21.8 mg, 80%) as a white solid after flash chromatography on silica (4:1, *n*-Hexane/EtOAc). m. p. 80-81 ^{o}C; $[\alpha]_D^{22}$ = -70.9; (*c* = 1.0, $CHCl_3$); ^{1}H NMR (300 MHz, $CDCl_3$): δ 7.84 (d, *J* = 8.8 Hz, 2H), 6.95 (d, *J* = 8.8 Hz, 2H), 6.70 (dd, *J* = 4.2 Hz, 9.8 Hz, 1H), 6.65 (d, *J* = 2.1 Hz, 1H), 5.56 (dt, *J* = 2.5 Hz, 9.8 Hz, 1H), 5.17 (s, 1H), 5.09 (d, *J* = 4.2 Hz, 1H), 3.88 (s, 3H), 3.47 (s, 3H), 3.41 (d, *J* = 3.5 Hz, 1H); ^{13}C NMR (100 MHz, $CDCl_3$): δ 189.0, 163.7, 155.8, 137.9, 137.5, 131.2, 130.0, 122.3, 113.8, 83.6, 77.7, 70.7, 55.7, 55.5; HRMS (ESI) calcd. for $[C_{16}H_{16}O_4Na]^+$, 295.0946; found 295.2952.

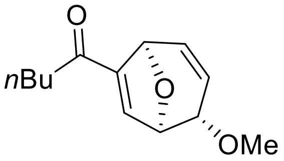

1-((1R,2S,5R)-2-methoxyl-8-oxabicyclo[3.2.1]oct-3,6-dien-6-yl)pentan-1-one (2p): Compound was prepared following the general procedure **C**, **1p** (29.6 mg, 0.1 mmol), **2p** was obtained (16.9 mg, 73%) as colorless oil after flash chromatography on silica (4:1, *n*-Hexane/EtOAc). $[\alpha]_D^{22}$ = 46.7; (*c* = 1.0, $CHCl_3$); ^{1}H NMR (400 MHz, $CDCl_3$): δ 6.83 (d, *J* = 2.9 Hz, 1H), 6.53 (ddd, *J* = 1.3, 4.3, 9.8 Hz, 1H), 5.52 (ddd, *J* = 2.2, 3.6, 9.8 Hz, 1H), 5.08 (t, *J* = 2.1 Hz, 1H), 5.00 (d, *J* = 4.2 Hz, 1H), 3.46 (s, 3H), 3.41 (dd, *J* = 0.6, 3.5 Hz, 2.66-2.63 (m, 2H), 1.64-1.56 (m, 2H), 1.33 (d, *J* = 7.6 Hz, 2H), 0.91 (t, *J* = 7.4 Hz, 3H); ^{13}C NMR (100 MHz, $CDCl_3$): δ 196.6, 157.1, 137.3, 136.6, 121.7,

83.1, 76.2, 70.8, 55.7, 39.1, 26.3, 22.3, 13.8; HRMS (ESI) calcd. for $[C_{13}H_{18}O_3Na]^+$, 254.1154; found 245.1161.

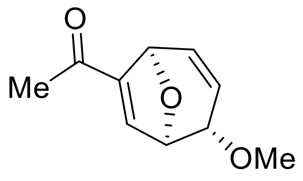

1-((1R,2S,5R)-2-methoxyl-8-oxabicyclo[3.2.1]oct-3,6-dien-6-yl)ethan-1-one (2q): Compound was prepared following the general procedure **C**, **1q** (25.4 mg, 0.1 mmol), **2q** was obtained (12.1 mg, 67%) as colorless oil after flash chromatography on silica (4:1, *n*-Hexane/EtOAc). $[\alpha]_D^{22}$ = 6.8; (c = 1.0, $CHCl_3$); ^1H NMR (400 MHz, $CDCl_3$): δ 6.86 (d, J = 2.3 Hz, 1H), 6.53 (ddd, J = 1.0, 4.2, 9.8 Hz, 1H), 5.53 (ddd, J = 2.2, 3.4, 9.8 Hz), 5.09 (t, J = 2.0 Hz, 1H), 5.01 (d, J = 4.3 Hz, 1H), 3.46 (s, 3H), 3.42 (d, J = 3.2 Hz, 1H), 2.34 (s, 3H); ^{13}C NMR (100 MHz, $CDCl_3$): δ 193.7, 157.4, 137.8, 137.1, 122.8, 83.1, 76.0, 70.7, 55.7, 26.8; HRMS (ESI) calcd. for $[C_{10}H_{12}O_3Na]^+$, 203.0684; found 203.0683.

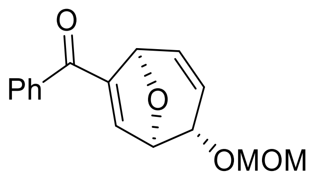

1-((1R,2S,5R)-2-methoxymethoxyl-8-oxabicyclo[3.2.1]oct-3,6-dien-6-yl)-1-phenyl -methanone (2r): Compound was prepared following the general procedure **C**, **1r** (37.6 mg, 0.1 mmol), **2r** was obtained (21.2 mg, 78%) as colorless oil after flash chromatography on silica (4:1, *n*-Hexane/EtOAc). $[\alpha]_D^{22}$ = -31.8; (c = 1.0, $CHCl_3$); ^1H NMR (300 MHz, $CDCl_3$): δ 7.82-7.79 (m, 2H), 7.61-7.56 (m, 1H), 7.42-7.44 (m, 2H), 6.72 (d, J = 2.2 Hz, 1H), 6.67 (dd, J = 1.6 Hz, 4.3 Hz, 9.8 Hz, 1H), 5.54 (ddd, J = 2.1,

3.7, 9.8 Hz, 1H), 5.18 (t, *J* = 2.0 Hz, 1H), 5.13 (d, *J* = 4.3 Hz, 1H), 4.80 (s, 2H), 3.70 (d, *J* = 3.7 Hz, 1H), 3.43 (s, 3H); ^{13}C NMR (100 MHz, $CDCl_3$): δ 190.3, 156.1, 138.8, 137.3, 137.1, 133.0, 128.8, 128.6, 123.0, 95.5. 84.7, 77.4, 67.9, 55.6; HRMS (ESI) calcd. for $[C_{16}H_{16}O_4Na]^+$, 295.0946; found 295.0949.

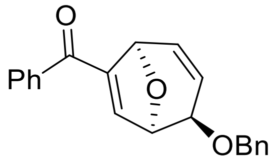

1-((1R,2R,5R)-2-benzyloxyl-8-oxabicyclo[3.2.1]octa-3,6-dien-6-yl)-1-phenyl-methanone (*epi*-2a): Compound was prepared following the general procedure **C**, ***epi*-1a**, (46.8 mg, 0.1 mmol), ***epi*-2a** was obtained (16.9 mg, 53%) as colorless oil after flash chromatography on silica (4:1, *n*-Hexane/EtOAc). $[\alpha]_D^{22}$ = -57.3; (*c* = 1.0, $CHCl_3$); ^{1}H NMR (400 MHz, $CDCl_3$): δ 7.81 (d, *J* = 7.7 Hz, 2H), 7.59-7.55 (m, 1H), 7.48-7.44 (m, 2H), 7.35-7.28 (m, 5H), 6.61 (d, *J* = 1.9 Hz, 1H), 6.58 (dd, *J* = 4.0, 10.4 Hz, 1H), 5.52 (d, *J* = 9.8 Hz, 1H), 5.36 (d, *J* = 6.2 Hz, 5.00 (d, J = 3.8 Hz, 1H), 4.63 (d, *J* = 11.8 Hz, 1H), 4.54 (d, *J* = 11.9 Hz, 1H), 4.41 (d, *J* = 6.1 Hz, 1H); ^{13}C NMR (100 MHz, $CDCl_3$): δ 190.3, 155.0, 140.3, 137.8, 137.3, 135.3, 132.8, 128.9, 128.5, 128.4, 128.0, 127.7, 124.1, 82.3, 77.9, 72.0, 69.6; HRMS (ESI) calcd. for $[C_{21}H_{18}O_3Na]^+$, 341.1154; found 341.1169.

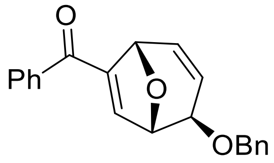

1-((1S,2R,5S)-2(benzyloxyl-8-oxabicyclo[3.2.1]oct-3,6-dien-6-yl)-1-phenyl-methanone (*ent* -2a): Compound was prepared following the general procedure **C**, ***ent***

-1a(46.8 mg, 0.1 mmol), ***ent* -2a** was obtained (25.4 mg, 80%) as colorless oil after flash chromatography on silica (4:1, *n*-Hexane/EtOAc). $[\alpha]_D^{22}$ = 66.5; (*c* = 1.0, $CHCl_3$); ^{1}H NMR (500 MHz, $CDCl_3$): δ 7.81-7.79 (m, 2H), 7.60-7.56 (m, 1H), 7.48-7.45 (m, 2H), 7.39-7.27 (m, 5H), 6.70 (ddd, *J* = 1.2, 4.3, 9.8 Hz, 1H), 6.70 (d, *J* = 2.1 Hz, 1H), 5.63 (ddd, *J* = 2.2, 3.6, 9.8 Hz, 1H), 5.22 (d, *J* = 2.0 Hz, 1H), 5.15 d, *J* = 4.3 Hz, 1H), 4.71 (s, 2H), 3.60-3.59 (dd, *J* = 0.4, 3.5 Hz, 1H); ^{13}C NMR (100 MHz, $CDCl_3$): δ 190.3, 155.7, 139.1, 138.1, 137.6, 137.2, 133.0, 128.8, 128.6, 128.4, 127.7, 122.9, 84.2, 77.4, 70.0, 68.8; HRMS (ESI) calcd. for $[C_{21}H_{18}O_3Na]^+$, 341.1154; found 341.1152.

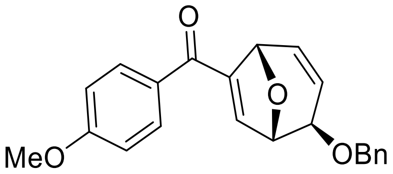

1-((1S,2R,5S)-2-benzyloxyl-8-oxabicyclo[3.2.1]oct-3,6-dien-6-yl)-1-(4-methoxyphe nyl)-methanone (*ent* -2b): Compound was prepared following the general procedure **C**, ***ent* -1b** (49.8 mg, 0.1 mmol), ***ent*-2b** was obtained (28.9 mg, 83%) as a white solid after flash chromatography on silica (4:1, *n*-Hexane/EtOAc). m. p. 97-99 °C; $[\alpha]_D^{22}$ = 52.0; (*c* = 1.0, $CHCl_3$); ^{1}H NMR (300 MHz, $CDCl_3$): δ 7.84-7.80 (m, 2H), 7.40-7.28 (m, 5H), 6.97-6.92 (m, 2H), 6.71 (ddd, *J* = 1.3, 4.3, 9.8 Hz, 1H), 6.63 (d, *J* = 2.2 Hz, 1H), 5.57 (ddd, *J* = 2.2, 3.6, 9.8 Hz, 1H), 5.21(t, *J* = 4.1 Hz, 1H), 5.11(d, *J* = 4.3 Hz, 1H), 4.71 (s, 2H), 3.87 (s, 3H), 3.61 (dd, *J* = 0.66, 3.5 Hz, 1H); ^{13}C NMR (100 MHz, $CDCl_3$): δ 189.0, 163.6, 155.8, 138.2, 137.8, 137.5, 131.1, 129.4, 128.4, 127.8, 127.7, 122.7, 113.8, 84.2, 77.7, 70.0, 68.9, 55.5; HRMS (ESI) calcd. for $[C_{22}H_{20}O_4Na]^+$,

371.1259; found 371.1253.

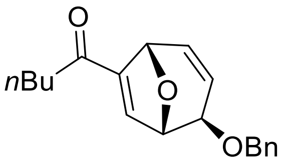

1-((1S,2R,5S)-2-benzyloxy-8-oxabicyclo[3.2.1]oct-3,6-dien-6-yl)pentan-1-one (*ent* -2g): Compound was prepared following the general procedure **C**, ***ent* -1g** (44.8 mg, 0.1 mmol), ***ent* -2g** was obtained (21.5 mg, 72%) as colorless oil after flash chromatography on silica (4:1, *n*-Hexane/EtOAc). $[\alpha]_D^{22}$ = -48.8; (*c* = 1.0, $CHCl_3$); ^1H NMR (500 MHz, $CDCl_3$): δ 7.39-7.27 (m, 5H), 6.81-6.81 (d, *J* = 2.3 Hz, 1H), 6.54 dd, *J* = 1.3, 4.3 Hz, 9.8 Hz, 1H), 5.54(dd, *J* = 2.2, 3.6, 9.8 Hz, 1H), 5.12(t, *J* = 2.1 Hz, 1H), 5.2(d, *J* = 4.3 Hz, 1H), 4.70 (s, 2H), 3.61 (dd, *J* = 0.8, 3.5 Hz, 1H), 2.64 (td, *J* = 2.5, 7.3 Hz, 1H), 1.63-1.56 (m, 2H), 1.36-1.29 (m, 2H), 0.92-0.89 (t, *J* = 7.4 Hz, 1H); ^{13}C NMR (100 MHz, $CDCl_3$): 196.6, 157.1, 138.2, 137.2, 136.5, 128.4, 127.7, 123.1, 83.7, 76.2, 70.0, 69.0, 39.0, 26.3, 22.3, 13.8; HRMS (ESI) calcd. for $[C_{19}H_{22}O_3Na]^+$, 321.1467; found 321.1468.

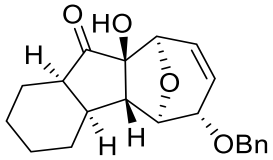

(4aR,4bS,5R,6S,9R,9aR,10aR)-6-(benzyloxy)-9a-hydroxy-decahydro-5,9-epoxybenzo[a]azulen-10(2H)-one (3i): Compound was prepared following the general procedure D, **2i** (17.0 mg, 0.05 mmol), **3i** was obtained (13.3 mg, 78%) as a colorless oil after flash chromatography on silica (2:1, *n*-Hexane/EtOAc)**.** ^1H NMR (400 MHz, $CDCl_3$): δ 7.39–7.27 (m, 5H), 6.30 (ddd, *J* = 9.8, 4.6, 1.1 Hz, 1H), 5.97 (ddd, *J* = 9.8, 4.1, 1.9 Hz, 1H), 4.70 (d, *J* = 12.1 Hz, 1H), 4.67 (d, *J* = 12.1 Hz, 1H), 4.44 (s, 1H),

4.20 (d, J = 4.6 Hz, 1H), 3.67 (d, J = 4.1 Hz, 1H), 3.01 (t, J = 6.5 Hz, 1H), 2.68 (s, 1H), 2.31-2.24 (m, 1H), 2.16 (d, J = 13.6 Hz, 1H), 1.87 (s, 1H), 1.83 (d, J = 15.0 Hz, 1H), 1.65-1.62 (m, 1H), 1.49-1.41 (m, 1H), 1.17– 1.03 (m, 2H), 0.97–0.0.86 (m, 1H); ^{13}C NMR (100 MHz, $CDCl_3$):δ 218.1, 138.3, 133.8, 128.5, 127.7, 124.1, 89.5, 86.0, 73.7, 70.4, 51.1, 47.8, 41.1, 31.3, 24.7, 21.9, 21.7.

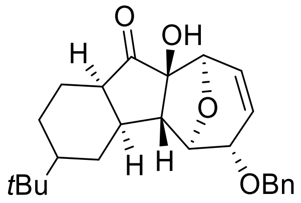

(4aR,4bS,5R,6S,9R,9aR,10aR)-6-(benzyloxy)-3-(tert-butyl)-9a-hydroxy-decahydro-5,9-epoxybenzo[a]azulen-10(2H)-one (3j): Compound was prepared following the general procedure D, **2j** (19.0 mg, 0.05 mmol), **3j** was obtained (11.8 mg, 61%) as a colorless oil after flash chromatography on silica (2:1, *n*-Hexane/EtOAc). ^{1}H NMR (500 MHz, $CDCl_3$): δ 7.39–7.29 (m, 5H), 6.30 (ddd, J = 10.0, 5.0, 4.0 Hz, 1H), 5.97 (ddd, J = 10.0, 4.5, 2.0 Hz, 1H), 4.70 (d, J = 12.0 Hz, 1H), 4.67 (d, J = 12.0 Hz, 1H), 4.45 (s, 1H), 4.20 (d, J = 4.5 Hz, 1H), 3.67 (d, J = 4.0 Hz, 1H), 2.98 (t, J = 6.5 Hz, 1H), 2.67 (s, 1H), 2.32–2.25 (m, 2H), 1.90-1.86 (m, 2H), 1.67–1.41 (m, 2H), 0.94–0.81 (m, 1H), 0.79 (s, 9H), 0.67–0.58 (m, 1H); ^{13}C NMR (100 MHz, $CDCl_3$): δ 218.2, 138.3, 133.8, 128.5, 127.7, 124.1, 89.5, 86.0, 73.7, 70.5, 51.3, 47.7, 46.7, 42.1, 32.8, 32.3, 27.3, 23.1, 22.4; HRMS (ESI) calcd. for $[C_{25}H_{33}O_4]^+$, 397.2379; found 397.2383.

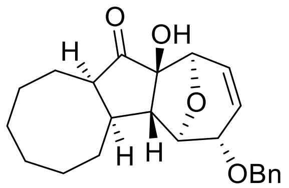

(1R,4S,5R,5aS,5bR,11aR,12aR)-4-(benzyloxy)-12a-hydroxy-dodecahydro-1,5-epoxycycloocta[a]azulen-12(1H)-one (3k): Compound was prepared following the general procedure D, **2k** (17.5 mg, 0.05 mmol), **3k** was obtained (13.2 mg, 72%) as a colorless oil after flash chromatography on silica (2:1, *n*-Hexane/EtOAc). ^{1}H NMR (400 MHz, $CDCl_3$): δ 7.40–7.28 (m, 5H), 6.30 (dd, *J* = 10.0, 4.6 Hz, 1H), 5.98 (ddd, *J* = 9.8, 4.2, 1.8 Hz, 1H), 4.72 (s, 2H), 4.43 (s, 1H), 4.22 (d, *J* = 4.6 Hz, 1H), 3.70 (d, *J* = 4.1 Hz, 1H), 2.83 (t, *J* = 0.8 Hz 1H), 2.52-2.50 (m, 2H), 1.99–1.93 (m, 2H), 1.78-1.72 (m, 3H), 1.68–1.59 (m, 2H), 1.51–1.46 (m, 2H), 1.42–1.36 (m, 3H), 1.31 – 1.31 (m, 2H); ^{13}C NMR (100 MHz, $CDCl_3$): δ 219.7, 138.3, 133.7, 128.5, 127.8, 124.2, 88.4, 87.2, 73.8, 70.5, 55.4, 52.6, 44.5, 33.2, 30.4, 26.1, 26.0, 24.7, 21.3; HRMS (ESI) calcd. for $[C_{23}H_{29}O_4]^+$, 369.2066; found 369.2069.

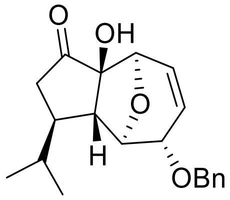

(3R,3aS,4R,5S,8R,8aR)-5-(benzyloxy)-8a-hydroxy-3-isopropyl-hexahydro-4,8-epoxyazulen-1(2H)-one (3m): Compound was prepared following the general procedure D, **2m** (15.5 mg, 0.05 mmol), **3m** was obtained (5.3 mg, 32%) as a colorless oil after flash chromatography on silica (2:1, *n*-Hexane/EtOAc). ^{1}H NMR (500 MHz, $CDCl_3$): δ 7.39–7.28 (m, 5H), 6.29 (dd, *J* = 10.0, 5.0 Hz, 1H), 6.00–5.98 (m, 1H), 4.68 (s, 2H), 4.38 (s, 1H), 4.32 (d, *J* = 4.5 Hz, 1H), 3.69 (d, *J* = 4.0 Hz, 1H),

2.78 (dd, J = 17.0, 8.5 Hz, 1H), 2.45–2.40 (m, 2H), 2.13 (s, 1H), 1.99 (t, J = 8.0 Hz, 1H), 1.65-1.60 (m, 1H), 0.90 (dd, J = 16.5, 6.5 Hz, 6H); ^{13}C NMR (100 MHz, $CDCl_3$): δ 218.2, 138.2, 133.5, 128.5, 127.8, 124.6, 89.6, 87.7, 76.9, 73.2, 70.4, 51.2, 46.7, 41.7, 32.8, 20.2, 19.7; HRMS (ESI) calcd. for $[C_{20}H_{25}O_4]^+$, 329.1753; found 329.1755.

Section D: Experimental Procedures and Characterization of Substrates and Products for Mechanistic Studies

Procedure for synthesis of substrate 1a' for ^{18}O labeling experiment:

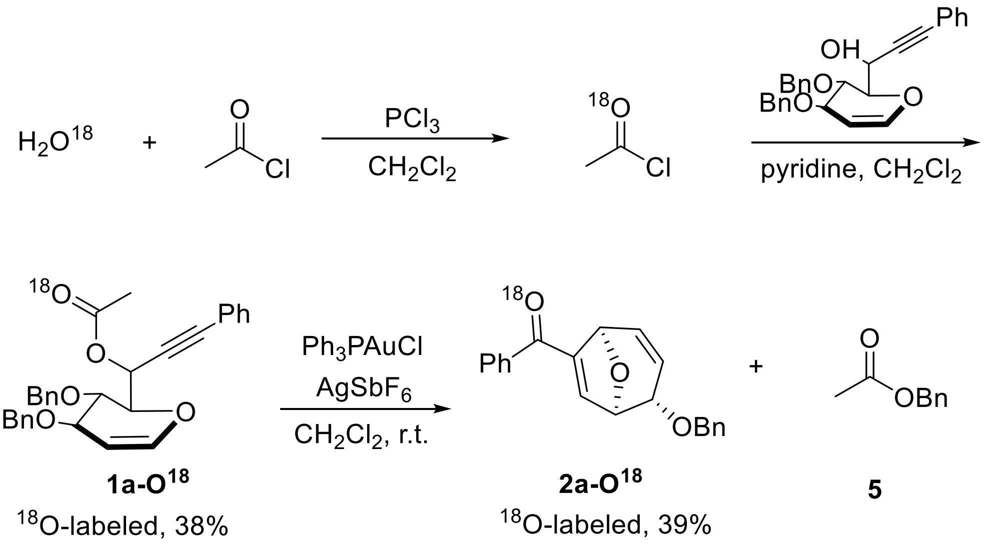

To a solution of acetyl chloride (471.0 mg, 0.34 mL, 6.0 mmol) in Schlenk tube, $H_2{}^{18}O$ was added dropwise *via* syringe under nitrogen at -20 ^{o}C. The reaction was stirred at room temperature for 30 min, then the tube was cooled to 0 ^{o}C and PCl_3 (268 mg, 2.0 mmol) was added. The reaction mixture was stirred at room temperature for 1h and then 2 mL anhydrous CH_2Cl_2 was added. The upper layer organic solvent was taken and added to solution of pyridine and propargylic alcohol (171 mg, 0.4 mmol) in anhydrous CH_2Cl_2. The reaction was stirred for 4 h. The mixture was diluted with CH_2Cl_2 (10 mL), washed with H_2O (5 mL), saturated $NaHCO_3$ solution (5 mL) and brine (5 mL). The organic layer was dried over Na_2SO_4 and filtered. Evaporation and flash chromatography on silica gel (8:1, *n*-Hexane/EtOAc) afforded the propargylic acetate **1a-^{18}O** (125 mg, 66%) as a 1:1.1 mixture of diastereomers

about the propargylic position. ^{1}H NMR (400 MHz, $CDCl_3$): δ 7.45-7.28 (m, 15H, both isomers), 6.49-6.47 (dd, J = 1.6, 8.1 Hz, 1H, major isomer), 6.45-6.43 (m, 1H, minor isomer), 6.24-6.22 (d, J = 6.1 Hz, 1H, minor isomer), 6.19-6.18 (d, J = 2.9 Hz, 1H, major isomer), 5.04-5.01 (dd, J =1.4, 4.6 H, 1H, minor isomer), 5.00-4.96 (d, J = 14.8 Hz, 1H, major isomer), 4.96-4.93 (dd, J = 2.9, 8.2 Hz, 1H, major isomer), 4.86-4.82 (d, J = 14.7 Hz, 1H, major isomer), 4.80-4.77 (d, J = 15.5 Hz, 1H, minor isomer), 4.71-4.56 (m, 2H, both isomers), 4.37-4.30 (m, 1H, both isomers), 4.17-4.13 (dd, J = 3.8, 13.2 Hz, 1H, major isomer), 4.10-4.05 (m, 1H, both isomers), 4.00-3.95 (dd, J = 9.2, 13.2 Hz, 1H, major isomer), 2.15 (s, 3H, major isomer), 2.13 (s, 3H, minor isomer); ^{13}C NMR (100 MHz, $CDCl_3$): δ 169.5, 169.5, 144.3, 144.0, 138.0, 138.0, 137.8, 137.5, 132.1, 131.9, 128.8, 128.8, 128.5, 128.5, 128.4, 128.4, 128.3, 128.2, 128.2, 128.0, 128.0, 127.8, 127.8, 127.7, 127.6, 121.8, 121.9, 100.5, 100.0, 87.4, 86.8, 83.8, 82.2, 77.4, 76.9, 75.4, 74.6, 72.6, 72.2, 71.8, 70.8, 70.3, 64.1, 61.7, 20.9; HRMS (ESI) calcd. for $[C_{35}H_{30}O_5{}^{18}ONa]^+$, 493.1877; found 493.1504. MS analysis of the products obtained indicated an isotopic composition about 38% (**Supplementary Figure 1**)

To solution of Ph_3PAuCl (2.5 mg, 5 mol %) and $AgSbF_6$ (3.4 mg, 10 mol %) in distilled CH_2Cl_2 (1 mL) was added the solution of proparglylic acetate **1a-^{18}O** (47.0 mg, 0.1 mmol) in distilled CH_2Cl_2 (1 mL). The reaction was stirred at room temperature until the starting material completely consumed. The mixture was filtered through a plug of silica and concentrated *in vacuo*. Purification of the residue by flash chromatography on silica gel (4:1, *n*-Hexane/EtOAc) afforded the product **2a-^{18}O**

(23.6 mg, 76% yield) as colorless oil. ^{1}H NMR (500 MHz, $CDCl_3$): δ 7.81-7.79 (m, 2H), 7.60-7.56 (m, 1H), 7.48-7.45 (m, 2H), 7.39-7.33 (m, 4H), 7.30-7.27 (m, 1H) 6.72-6.68 (ddd, J = 1.3, 4.3, 9.8 Hz, 1H), 6.70-6.69 (d, J = 2.1 Hz, 1H), 5.60-5.56 (ddd, J = 2.1, 3.6, 9.8 Hz, 1H); 5.22-5.21 (t, J = 2.0 Hz, 1H); 5.15-5.14 (d, J = 4.3 Hz, 1H), 4.71 (s, 2H), 3.60-3.59 (dd, J = 0.6, 3.4 Hz, 1H); ^{13}C NMR (100 MHz, $CDCl_3$): δ 190.3, 190.3, 155.8, 139.2, 138.1, 137.6, 137.2, 133.0, 128.8, 128.6, 128.5, 127.8, 122.9, 84.2, 70.0, 68.8; HRMS (ESI) calcd. for $[C_{21}H_{18}O_2{}^{18}ONa]^+$, 343.1196; found 343.0901. MS analysis of the products obtained indicated an isotopic composition about 39% (**Supplementary Figure 2**).

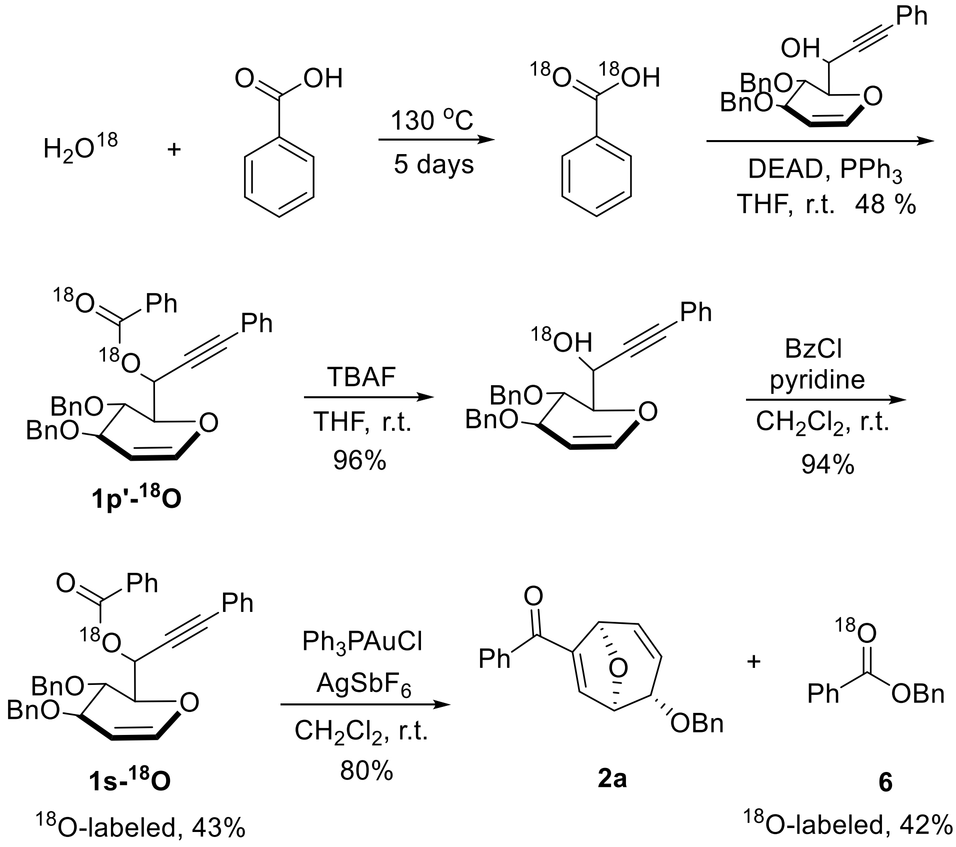

$H_2{}^{18}O$ (700 μL, 39.0 mmol) was added to benzoic acid (215 mg, 11.8 mmol) in Schlenk tube under nitrogen and the reaction mixture was stirred at 130 °C for 5 days.

After cooling to room temperature, the water was removed under vacuum by oil pump. The ^{18}O labeled benzoic acid was identical to the ^{1}H NMR spectrum and MS of the known compound matched that as reported.

A Schlenk tube was charged with propargylic alcohol (222 mg, 0.68 mmol), ^{18}O labeled benzoic acid (100 mg, 0.82 mmol), PPh_3 (231 mg, 0.88 mmol) and anhydrous THF (10 mL). The reaction mixture was treated with the diethyl azodicarboxylate (142 mg. 123 μL) *via* syringe at 0 °C and stirred at room temperature overnight. The solvent was removed *in vacuo* and the product was purified by flash chromatography (10:1, *n*-Hexane/EtOAc) providing 133.2 mg (48% yield) of the doubly ^{18}O labeled benzoate **1s'-^{18}O** as colourless oil. The fully protected propargylic benzoate derivative (133.2 mg, 0.24 mmol) was dissolved in 1M THF solution of TBAF (3.0 ml, 3 mmol) and stirred for 4 h. The mixture was concentrated *in vacuo* and the product was purified by flash chromatography (8:1, *n*-Hexane/EtOAc) providing 101.6 mg (96% yield) of the ^{18}O labeled propargylic alcohol as a colourless oil.

To a mixture of the ^{18}O labeled propargylic alcohol (101.6 mg, 0.23 mmol) in CH_2Cl_2 (4 mL), pyridine (158 mg, 2 mmol) and benzoate chloride (30.7 mg, 0.4 mmol) were added and the mixture was stirred for 4 h. The mixture was then diluted with CH_2Cl_2 (5 mL), washed with H_2O (3 mL), saturated $NaHCO_3$ solution (3 mL) and brine (3 mL). The organic layer was dried over Na_2SO_4 and filtered. Evaporation of solvent and flash chromatography on silica gel (8:1, *n*-Hexane/EtOAc) afforded the ^{18}O labeled propargylic benzoate **1s-^{18}O** (118.4 mg, 94% yield) as a light yellow oil. ^{1}H NMR (500 MHz, $CDCl_3$): δ 8.13-8.10 (m, 2H, both isomers), 7.61-7.57 (m, 1H,

both isomers), 7.48-7.42 (m, 4H, both isomers), 7.38-7.27 (m, 12H, both isomers), 6.53-6.52 (dd, J = 1.0, 6.1 Hz, 1H, major isomer), 6.50-6.48 (m, 1H, minor isomer), 6.48-6.47 (d, J = 6.1 Hz, 1H, minor isomer); 6.44-6.43 (d, J = 3.4 Hz, 1H, major isomer), 5.07-5.05 (dd, J = 3.5, 6.2 Hz, 1H, minor isomer), 5.04-5.01 (d, J = 11.2 Hz, 1H, major isomer), 4.99-4.97 (dd, J = 2.2, 6.1 Hz, 1H, major isomer), 4.91-4.88 (d, J = 11.2 Hz, 1H, major isomer), 4.82-4.79 (d, J = 11.5 Hz, 1H, minor isomer), 4.70-4.57 (m, 3H, both isomers), 4.49-4.45 (td, J = 0.9, 5.9 Hz, 1H, minor isomer), 4.37-4.35 (dt, J = 1.8, 4.8 Hz, 1H, major isomer), 4.35-4.32 (dd, J = 3.4, 9.4 Hz, 1H, major isomer), 4.19-4.17 (m, 1H, minor isomer), 4.15-4.13 (m, 1H, minor isomer), 4.10-4.06 (dd, J = 6.6, 9.4 Hz, 1H, major isomer); ^{13}C NMR (100 MHz, $CDCl_3$): 165.2, 165.2, 165.1, 165.1, 144.4, 144.2, 138.0, 138.0, 137.8, 137.5, 133.3, 133.2, 132.1, 132.0, 130.0, 130.0, 129.6, 129.5, 128.8, 128.5, 128.4, 128.4, 128.4, 128.4, 128.3, 128.1, 128.14, 127.9, 127.8, 127.7, 127.7, 121.9, 121.9, 100.4, 100.0, 87.4, 87.0, 83.8, 82.5, 77.4, 76.4, 75.1, 74.3, 72.9, 72.7, 70.8, 70.5, 64.5, 64.5, 62.4, 62.4; HRMS (ESI) calcd. for $[C_{35}H_{30}O_5{}^{18}ONa]^+$, 555.2033; found 555.2044. MS analysis of the products obtained indicated an isotopic composition about 43% (**Supplementary Figure 3a**).

To solution of Ph_3PAuCl (2.5 mg, 5 mol %) and $AgSbF_6$ (3.4 mg, 10 mol %) in distilled CH_2Cl_2 (1 mL) was added the solution of ^{18}O labeled propargylic benzoate **1p-^{18}O** (53.4 mg, 0.1 mmol) in distilled CH_2Cl_2 (1 mL). The reaction was stirred at room temperature until the starting material completely consumed. The mixture was filtered through a plug of silica and concentrated *in vacuo*. Purification of the residue by flash chromatography on silica gel (4:1, *n*-Hexane/EtOAc) afforded the product **2a**

(25.2 mg, 80% yield) and ^{18}O labeled benzyl benzoate **6-^{18}O** (16.8 mg, 78% yield). ^{1}H NMR (400 MHz, $CDCl_3$): δ 8.09-8.07 (m, 2H), 7.58-7.54 (m, 1H), 7.47-7.33 (m, 7H), 5.37 (s, 2H); ^{13}C NMR (100 MHz, CDCl3): 166.4, 166.4, 136.1, 133.0, 130.1, 129.7, 128.6, 128.4, 128.2, 128.2, 66.7; HRMS (ESI) calcd. for $[C_{14}H_{12}O^{18}ONa]^+$, 237.0777; found 237.0849. MS analysis of the products obtained indicated an isotopic composition about 42% (**Supplementary Figure 3b**).

Section E: Preparation of 2,4-dinitrophenyl hydrazone S2a for Stereochemistry Identification

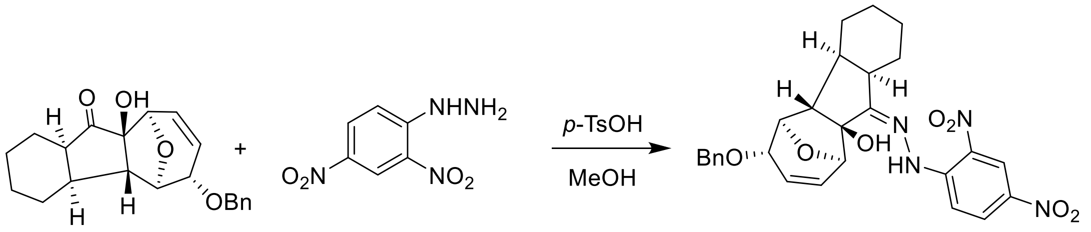

3i **9**

To a solution of ketone **3i** (15 mg, 0.045 mmol) in methanol (1 ml) was added *p*-TsOH (2 mg, 10 μmol) followed by the 2,4-dinitrophenyl hydrazine (15 mg, 0.074 mmol). The reaction was heated to reflux until the starting material completely consumed as indicated by TLC. The reaction was cooled to r.t. and the solvent was removed by rotary evaporation. Purification of the residue by flash chromatography on silica gel (4:1, *n*-Hexane/EtOAc) afforded the product **9** (18.8 mg, 82% yield) . A crystal of S2a was generated with EA and an X-ray structure of **9** was obtained; ^{1}H NMR (400 MHz, $CDCl_3$): δ 11.03 (s, 1H), 9.13 (s, 2H), 8.30 (d, J = 9.3 Hz, 1H), 7.99 (dd, J = 18.1, 9.7 Hz, 1H), 7.47–7.26 (m, 5H), 6.44 (dd, J = 10.0, 4.6 Hz, 1H), 6.23–

6.09 (m, 1H), 4.78–4.64 (m, 2H), 4.59 (d, J = 4.6 Hz, 1H), 4.34 (s, 1H), 3.59 (d, J = 4.1 Hz, 1H), 3.16 (s, 1H), 2.42-2.31 (m, 2H), 2.23-2.14 (m, 2H), 2.08-2.00 (m, 1H), 1.77-1.74 (m, 3H), 1.41–1.21 (m, 3H).

Section E: X-ray Structure and Data for Compound 2b and 9

Table 1: Crystal data and structure refinement for 2b.

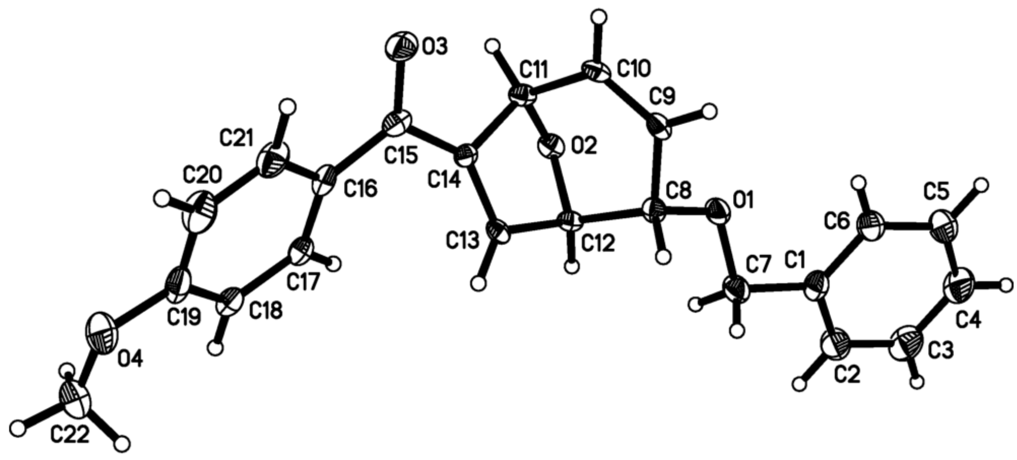

**Chemical formula**	$C_{22}H_{20}O_4$
**Formula weight**	348.38
**Temperature**	103(2) K
**Wavelength**	0.71073 Å
**Crystal size**	0.040 × 0.280 × 0.300 mm
**Crystal habit**	colorless plate
**Crystal system**	Orthorhombic
**Space group**	P 21 21 21
**Unit cell dimensions**	a = 5.8597(6) Å
	b = 8.4350(8) Å
	c = 35.607(4) Å
**Volume**	1759.9(3) $Å^3$
**Z**	4
**Density (calcdulated)**	1.315 g/cm^3
**Absorption coefficient**	0.090 mm^{-1}

**F(000)**	736
**Theta range for data collection**	2.29 to 25.35°
**Index ranges**	-6<=h<=6, -10<=k<=9, -42<=l<=42
**Reflections collected**	12377
**Independent reflections**	3111 [R(int) = 0.0420]
**Coverage of independent reflections**	97.60%
**Absorption correction**	multi-scan
**Max. and min. transmission**	0.9960 and 0.9740
**Structure solution technique**	direct methods
**Structure solution program**	SHELXS-97 (Sheldrick 2008)
**Refinement method**	Full-matrix least-squares on F^2
**Refinement program**	SHELXL-2013 (Sheldrick, 2013)
**Function minimized**	Σ w(F_o^2 - F_c^2)^2
**Data / restraints / parameters**	3111 / 0 / 236
**Goodness-of-fit on F^2**	1.089
**Final R indices**	2754 data; I>2σ(I) R1 = 0.0467, wR2 = 0.0946
	all data R1 = 0.0555, wR2 = 0.0978
**Weighting scheme**	$w=1/[\sigma^2(F_o^2)+(0.0376P)^2+0.7934P]$
	where $P=(F_o^2+2F_c^2)/3$
**Absolute structure parameter**	0.3(6)
**Largest diff. peak and**	0.194 and -0.212 eÅ^{-3}

**hole**	
**R.M.S. deviation from mean**	0.046 eÅ^{-3}

Table 2: Crystal data and structure refinement for 9.

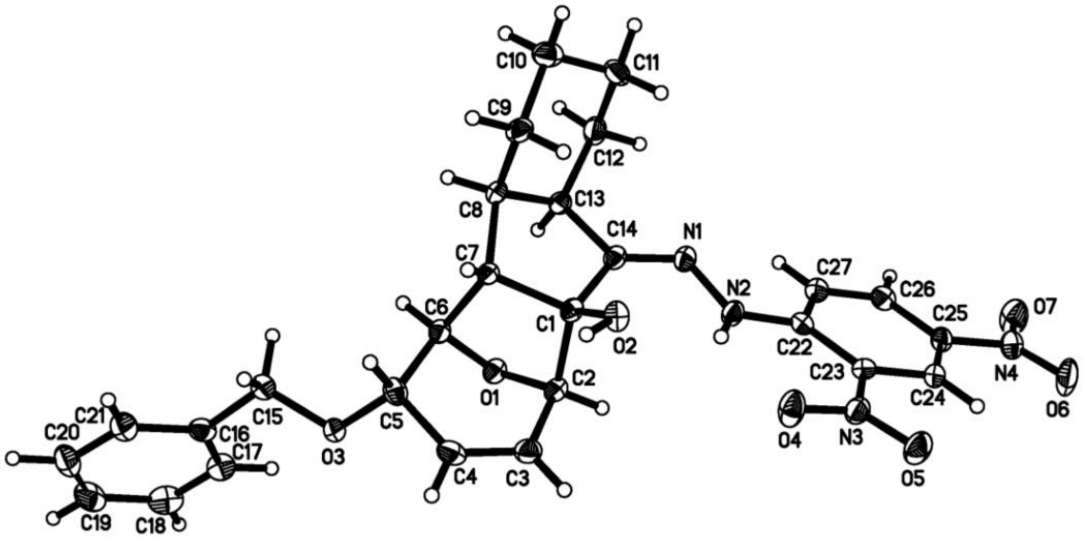

**Chemical formula**	$C_{27}H_{28}N_4O_7$	
**Formula weight**	520.53 g/mol	
**Temperature**	103(2) K	
**Wavelength**	1.54178 Å	
**Crystal size**	0.100 x 0.120 x 0.200 mm	
**Crystal habit**	yellow block	
**Crystal system**	orthorhombic	
**Space group**	P 21 21 21	
**Unit cell dimensions**	a = 5.5338(6) Å	α = 90°
	b = 16.6117(17) Å	β = 90°
	c = 26.533(3) Å	γ = 90°
**Volume**	2439.1(4) Å^3	
**Z**	4	
**Density (calculated)**	1.418 g/cm^3	
**Absorption coefficient**	0.864 mm^{-1}	
**F(000)**	1096	

**Theta range for data collection**	3.14 to 68.25°	
**Reflections collected**	4341	
**Coverage of independent reflections**	98.0%	
**Absorption correction**	Multi-Scan	
**Max. and min. transmission**	0.9190 and 0.8460	
**Structure solution technique**	direct methods	
**Structure solution program**	XT, VERSION 2014/5	
**Refinement method**	Full-matrix least-squares on F2	
**Refinement program**	SHELXL-2014/7 (Sheldrick, 2014)	
**Function minimized**	Σ w(Fo2 - Fc2)2	
**Data / restraints / parameters**	4341 / 0 / 348	
**Goodness-of-fit on F2**	1.088	
**Final R indices**	4188 data; $I>2\sigma(I)$	R1 = 0.0471, wR2 = 0.1278
	all data	R1 = 0.0520, wR2 = 0.1352
**Weighting scheme**	$w=1/[\sigma^2(F_o^2)+(0.0611P)^2+2.8738P]$ where $P=(F_o^2+2F_c^2)/3$	
**Absolute structure parameter**	-0.09(10)	
**Extinction coefficient**	0.0042(7)	
**Largest diff. peak and hole**	0.265 and -0.256 eÅ^{-3}	
**R.M.S. deviation from mean**	0.064 eÅ^{-}	

Section F: Supplementary Figures

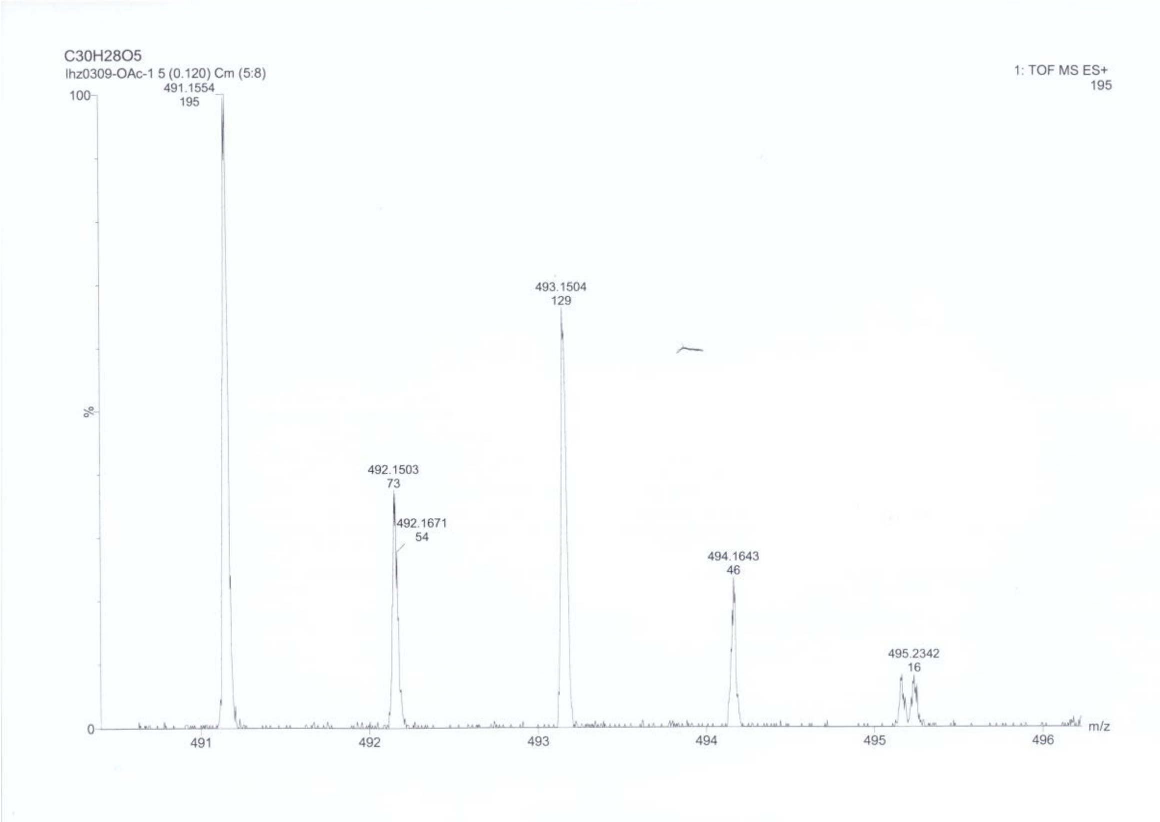

Supplementary Figure 1. Mass Spectrum of 1a-^{18}O.

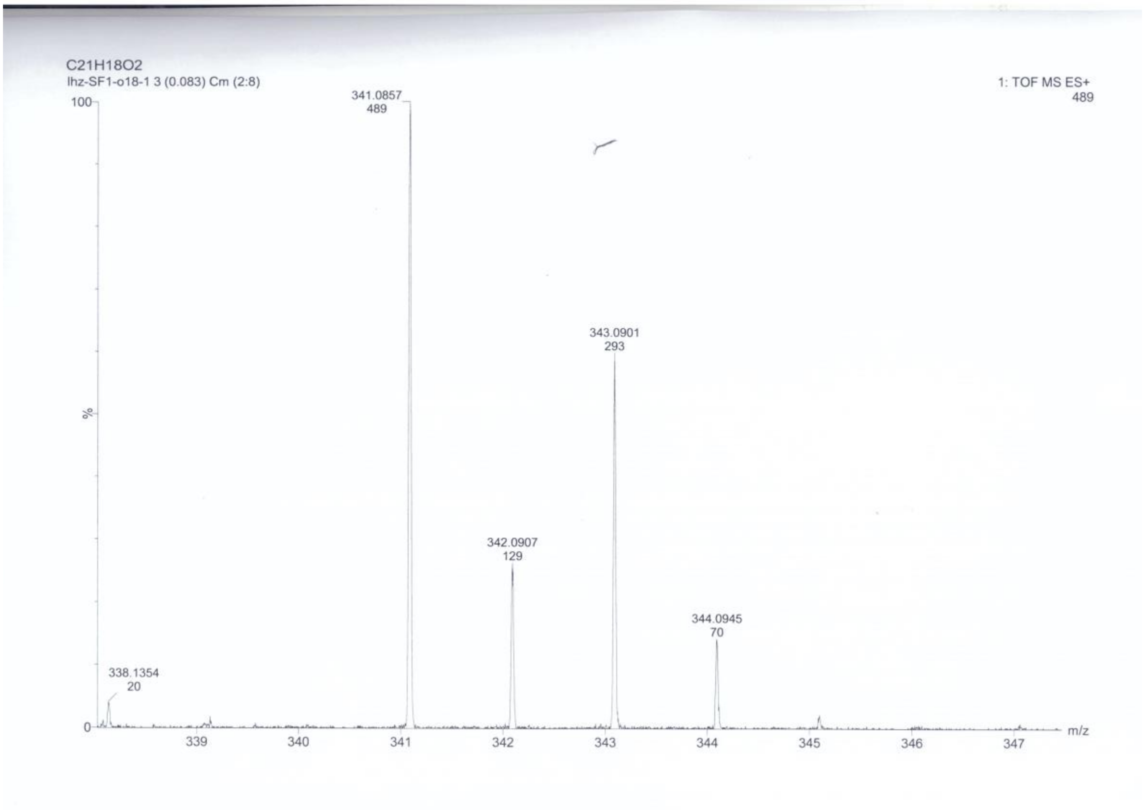

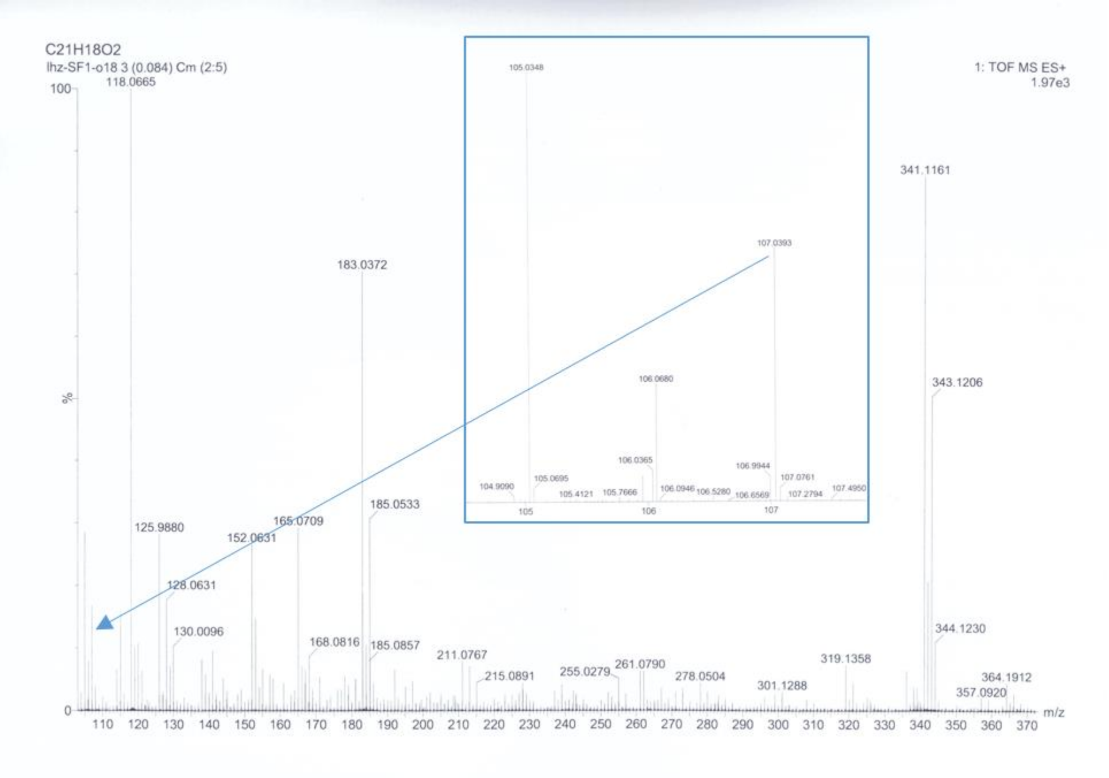

Supplementary Figure 2. Mass Spectrum of 2a-^{18}O.

a

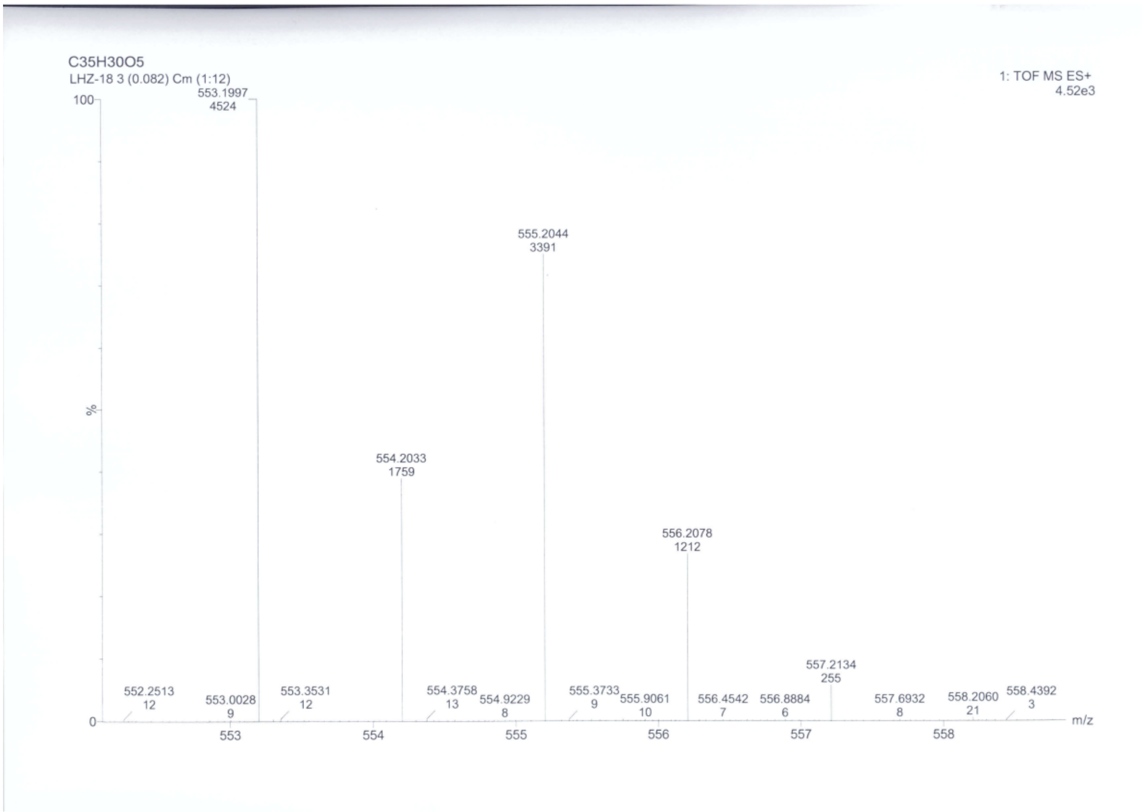

b

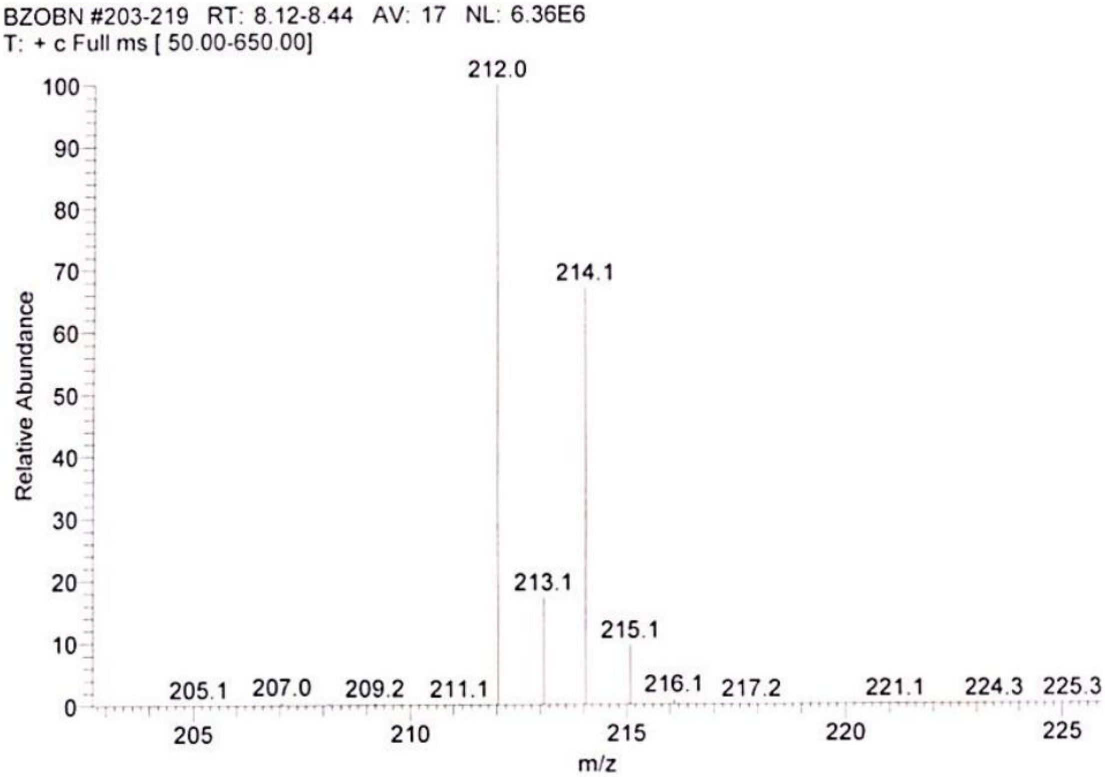

Supplementary Figure 3. Mass Spectrum of 1s-^{18}O (a) and 6-^{18}O (b).

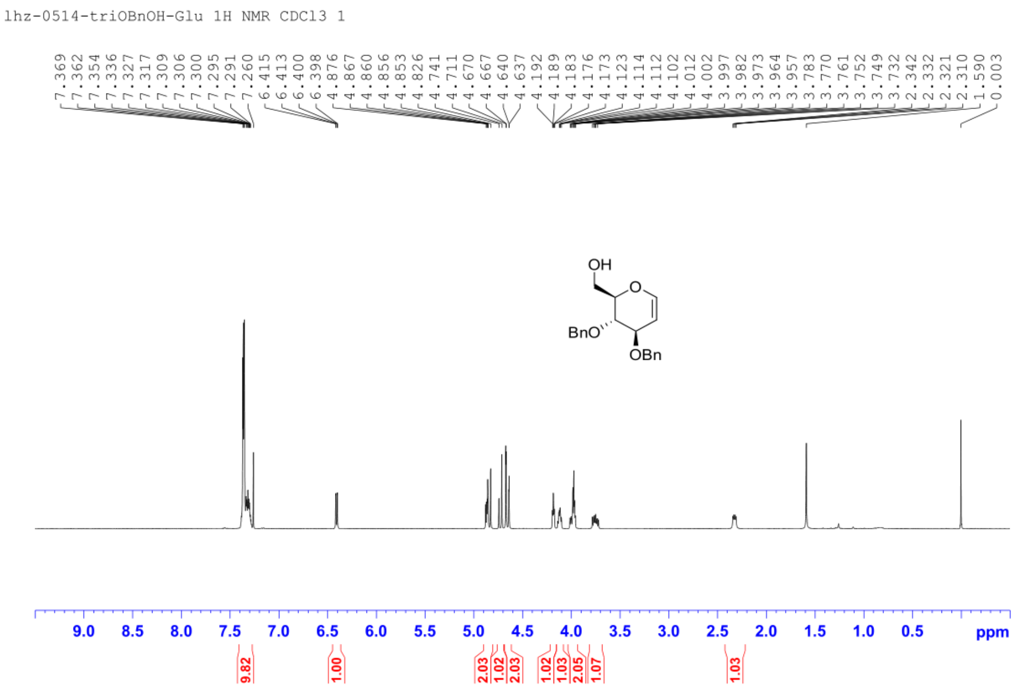

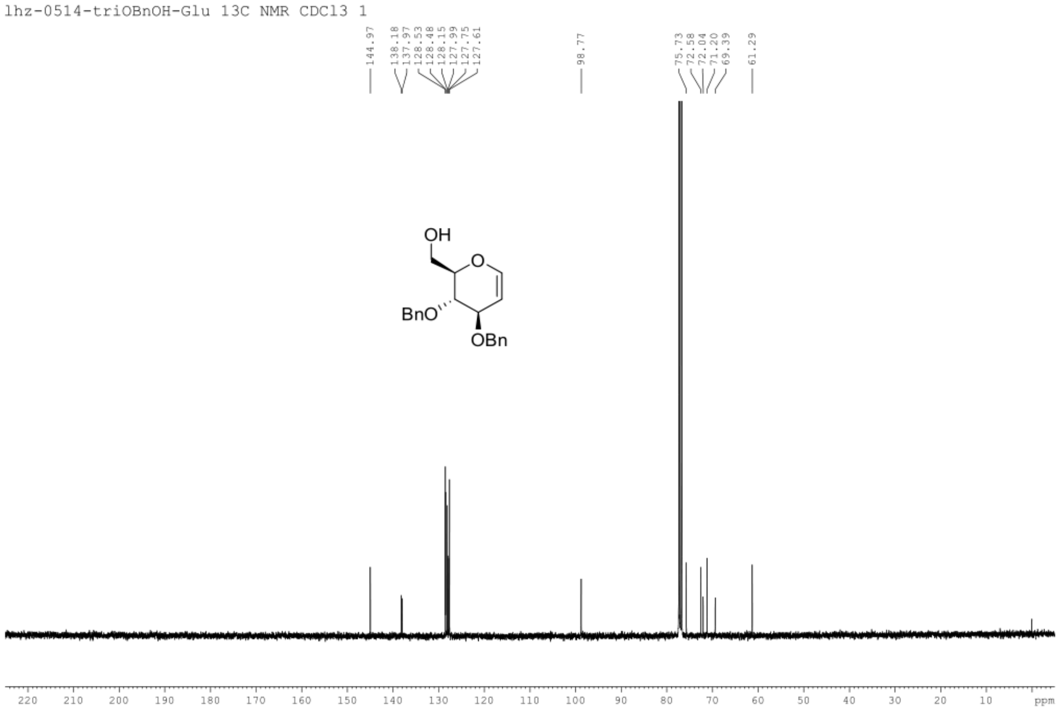

Supplementary Figure 4. ^{1}H and ^{13}C NMR spectrum for 8a.

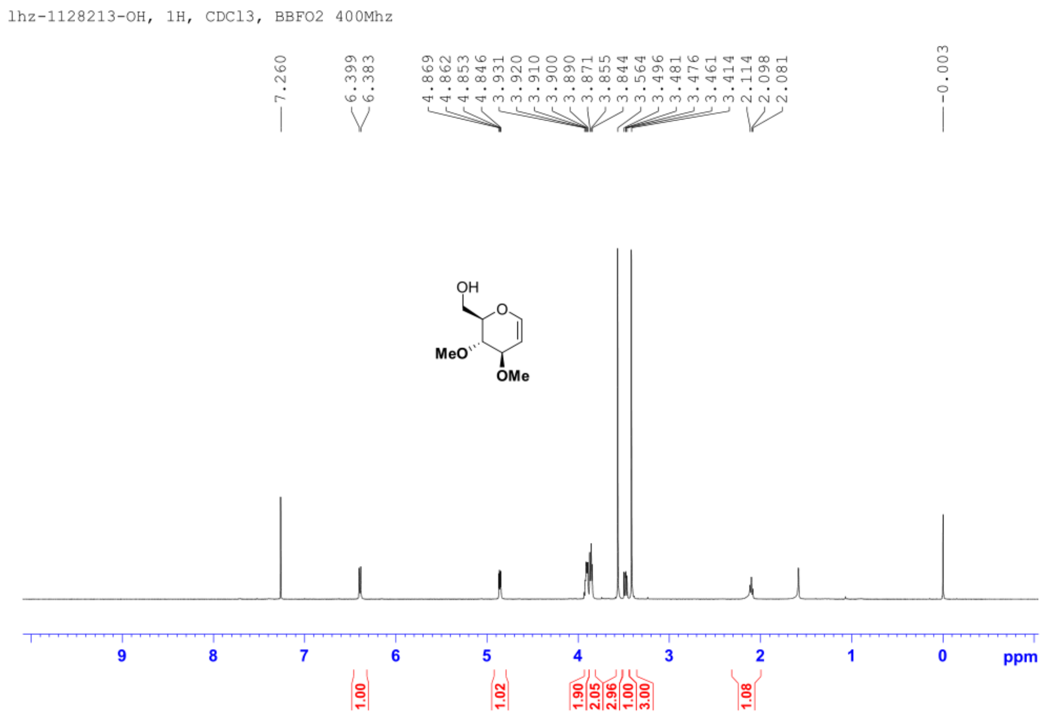

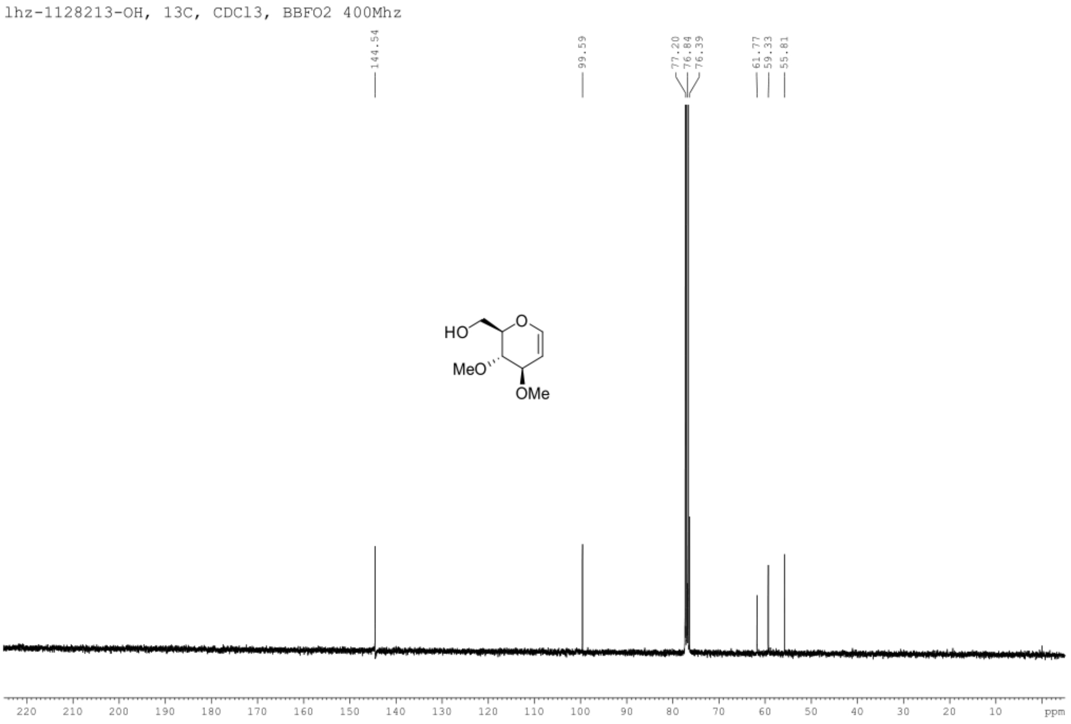

Supplementary Figure 5. ^{1}H and ^{13}C NMR spectrum for 8b.

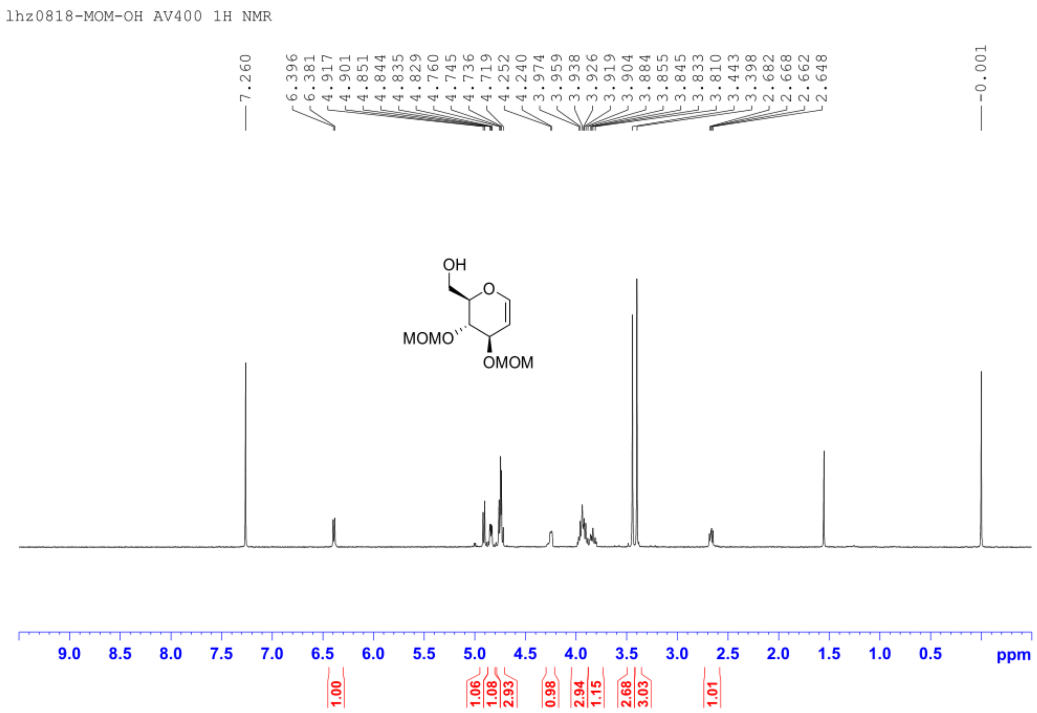

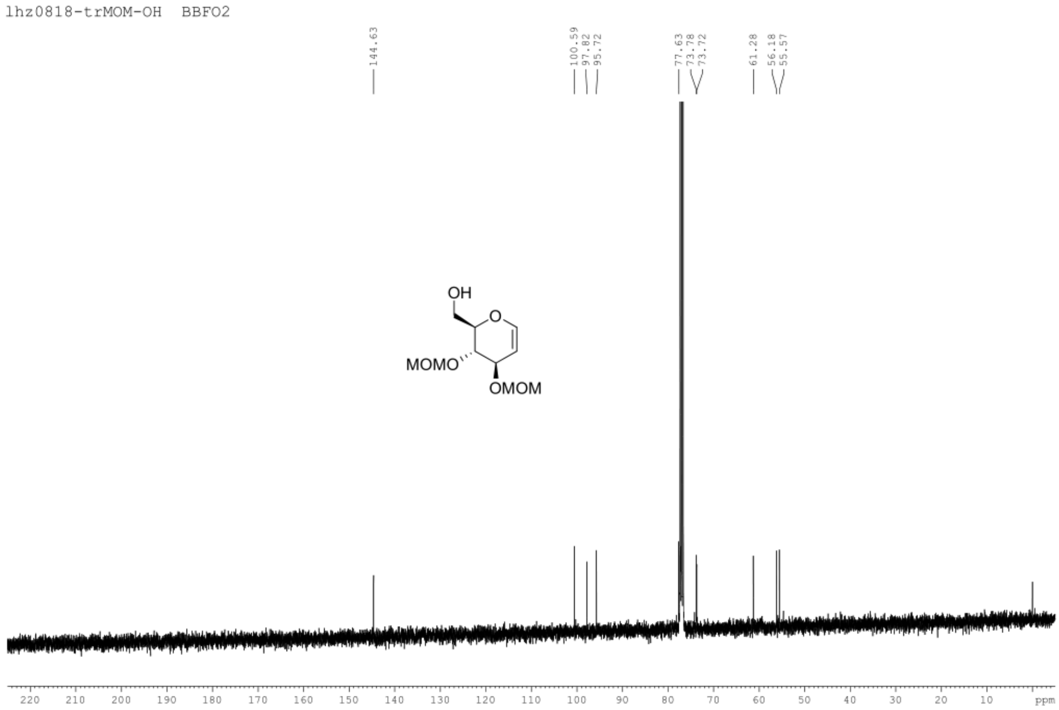

Supplementary Figure 6. ^{1}H and ^{13}C NMR spectrum for 8c.

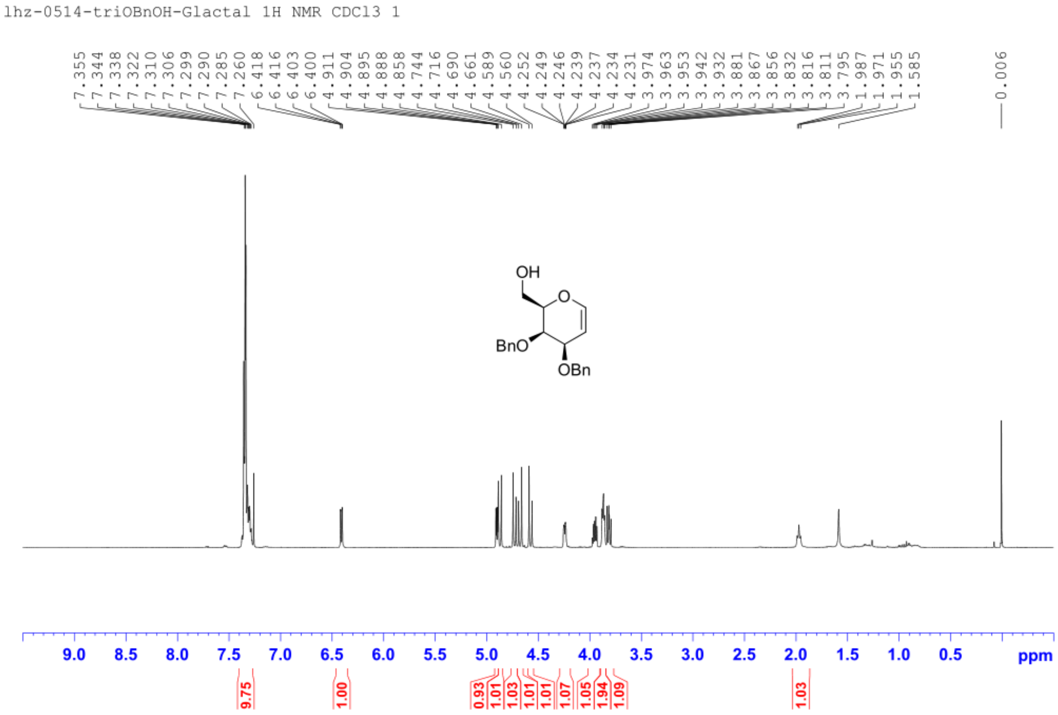

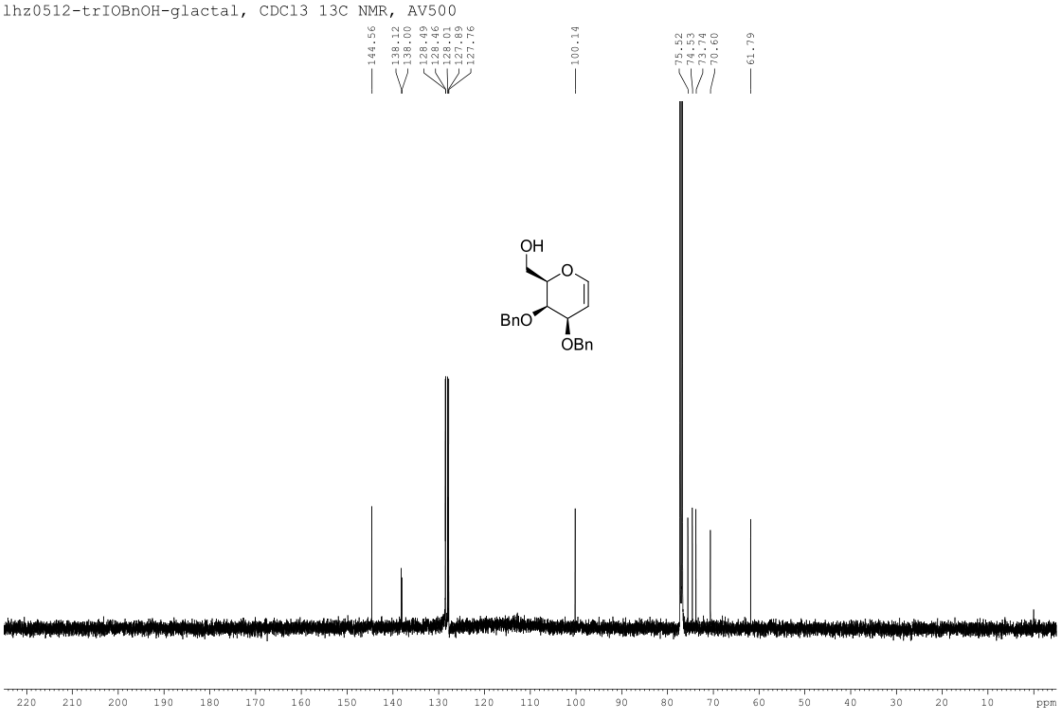

Supplementary Figure 7. ^{1}H and ^{13}C NMR spectrum for 8d.

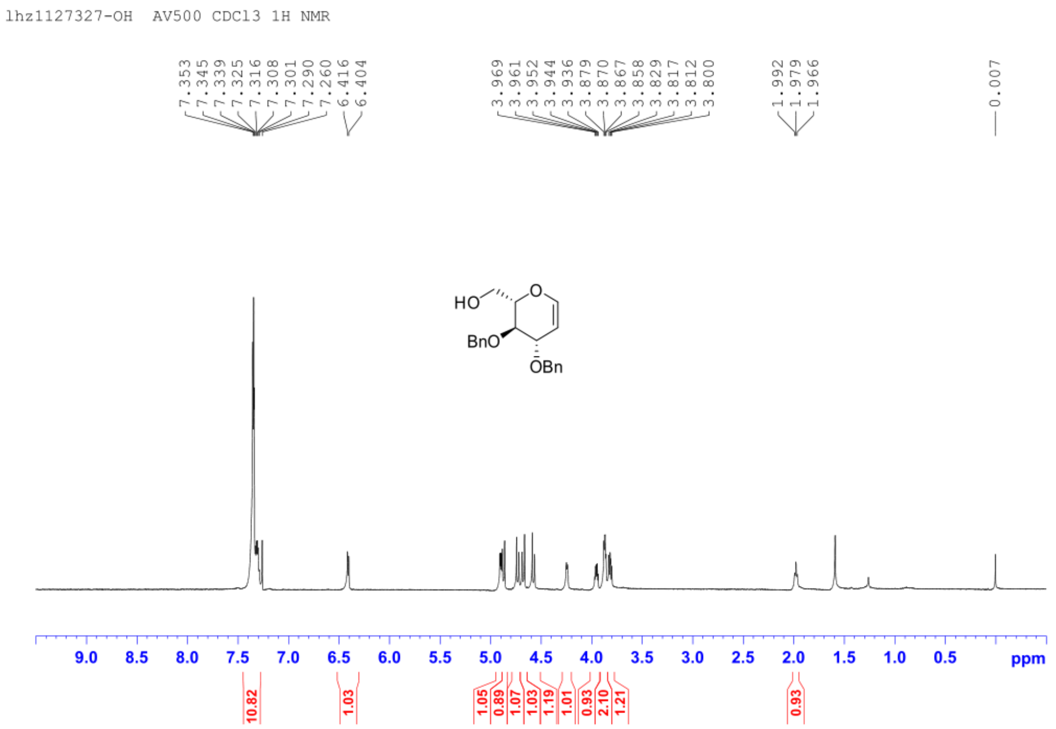

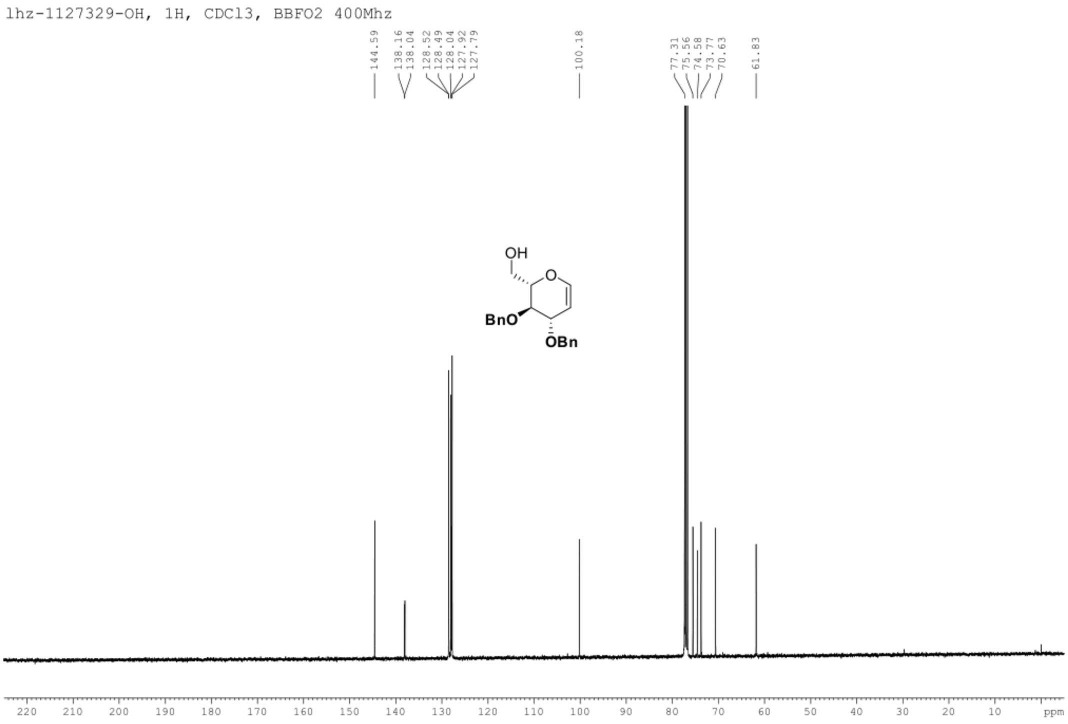

Supplementary Figure 8. ^{1}H and ^{13}C NMR spectrum for 8e.

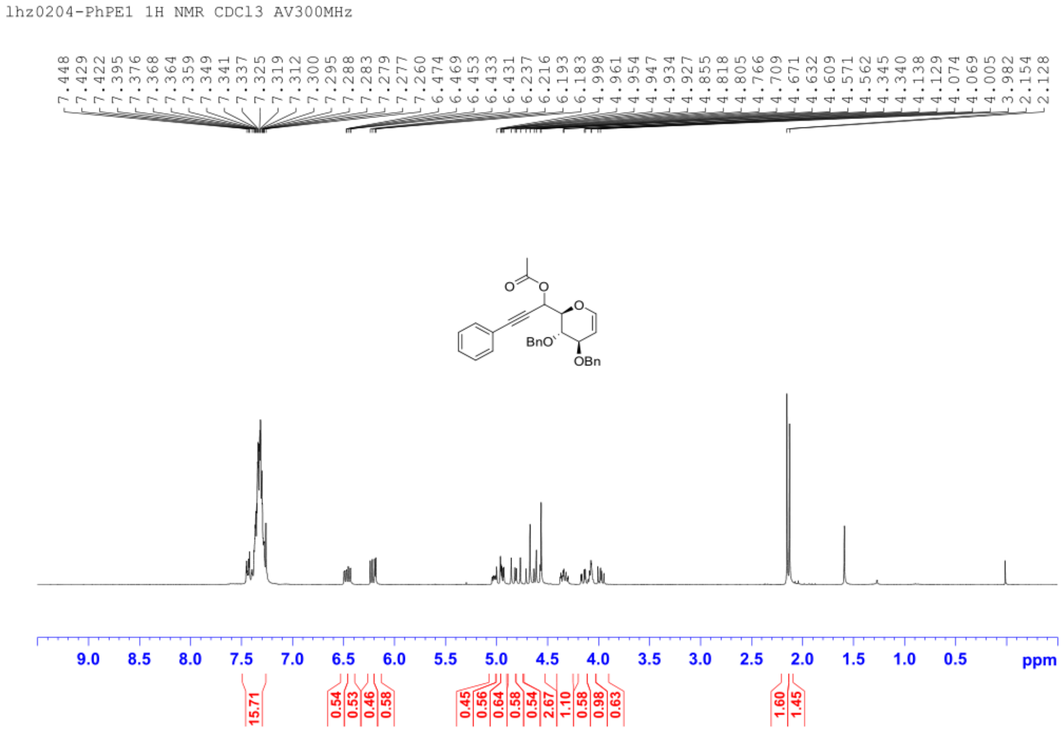

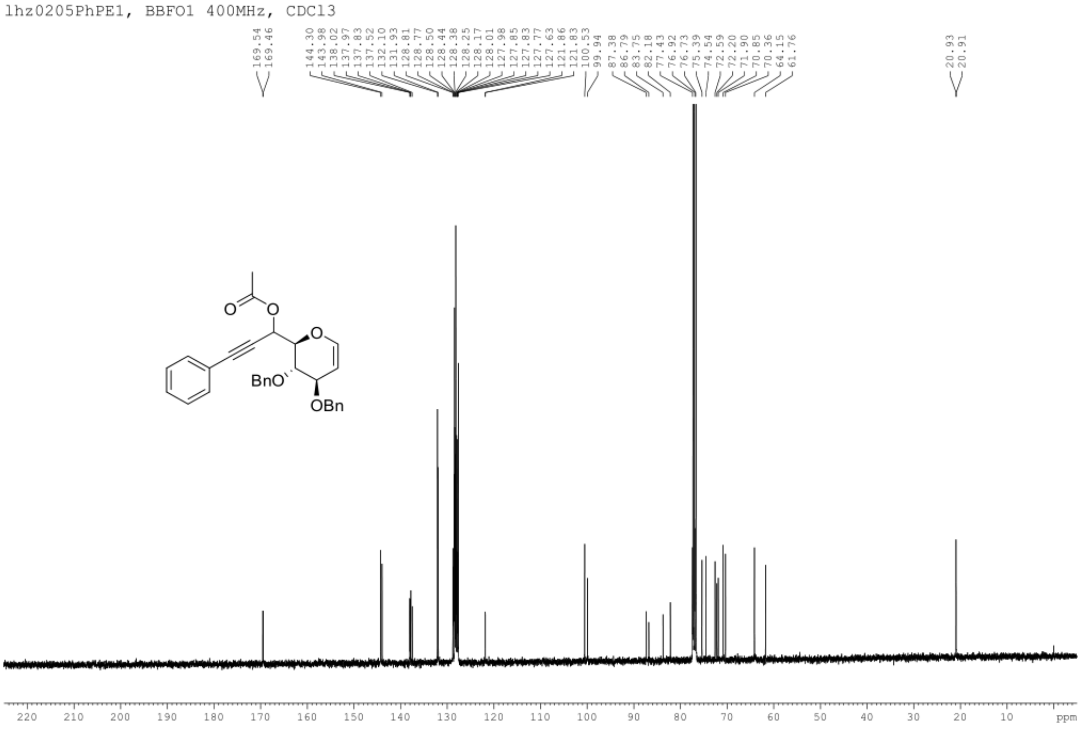

Supplementary Figure 9. ^{1}H and ^{13}C NMR spectrum for 1a.

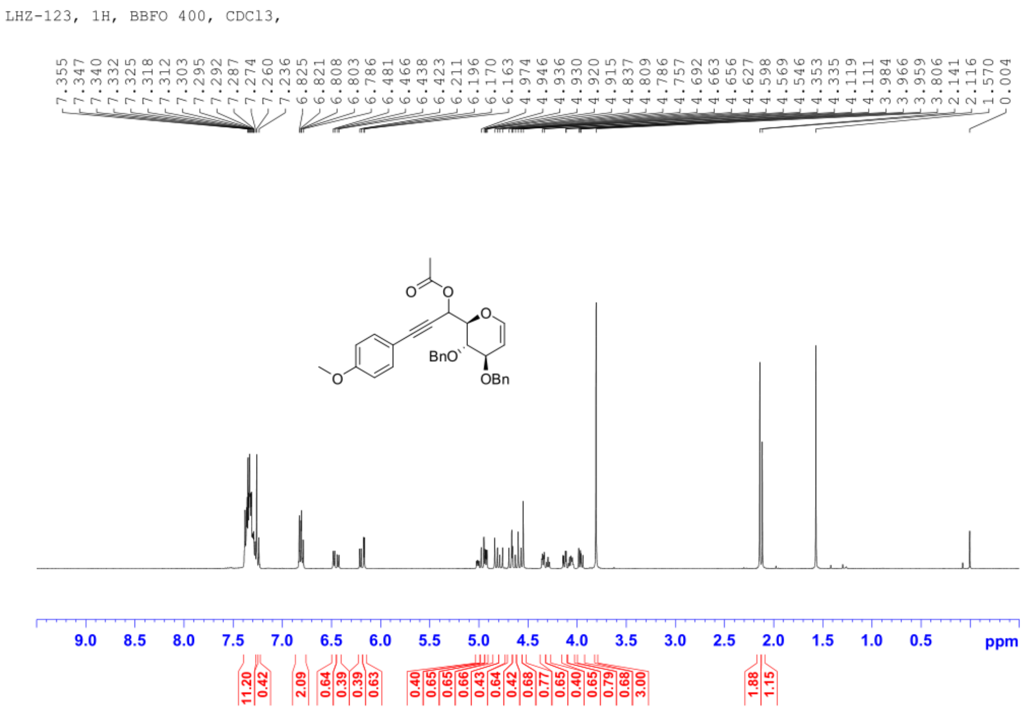

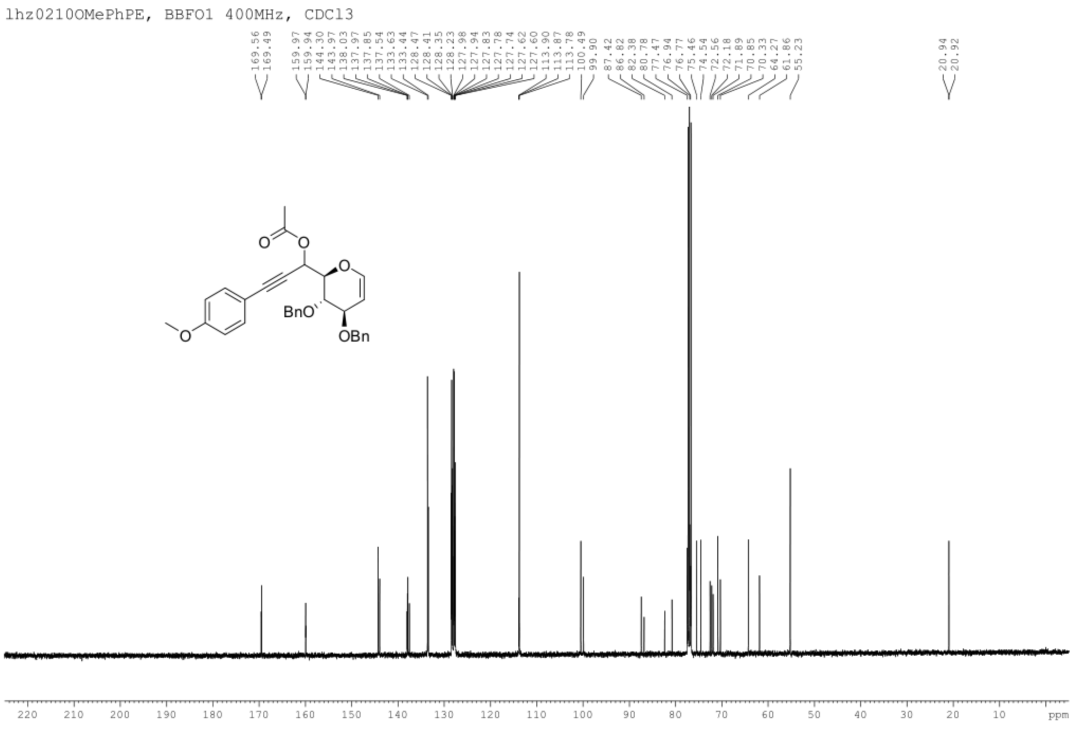

Supplementary Figure 10. ^{1}H and ^{13}C NMR spectrum for 1b.

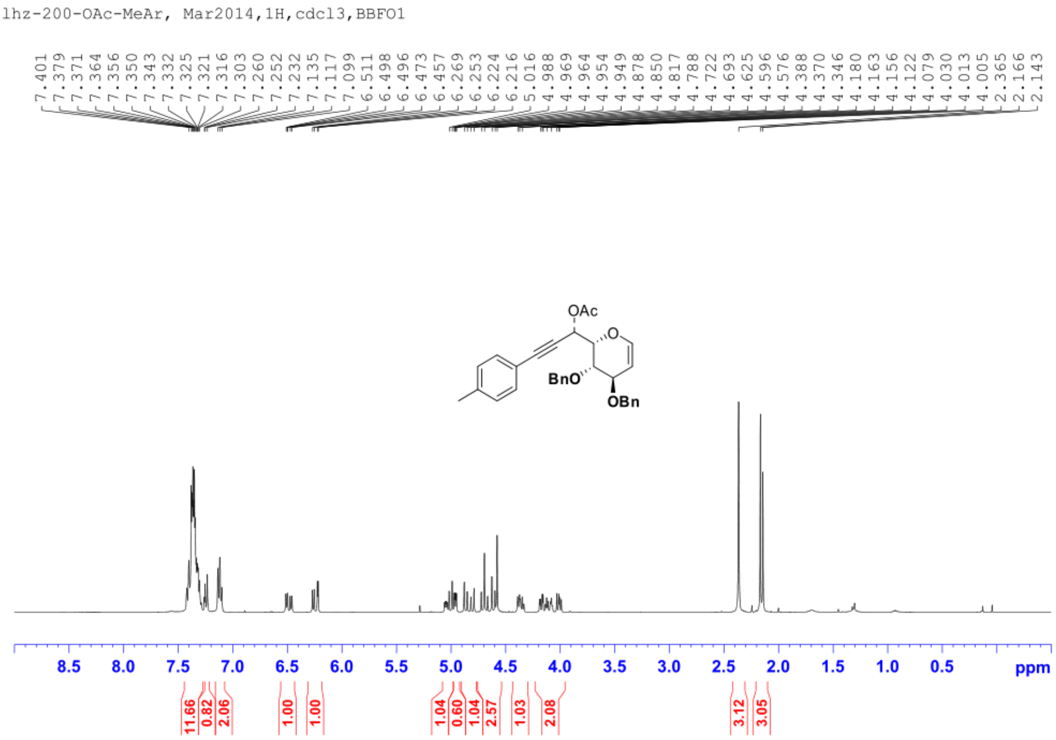

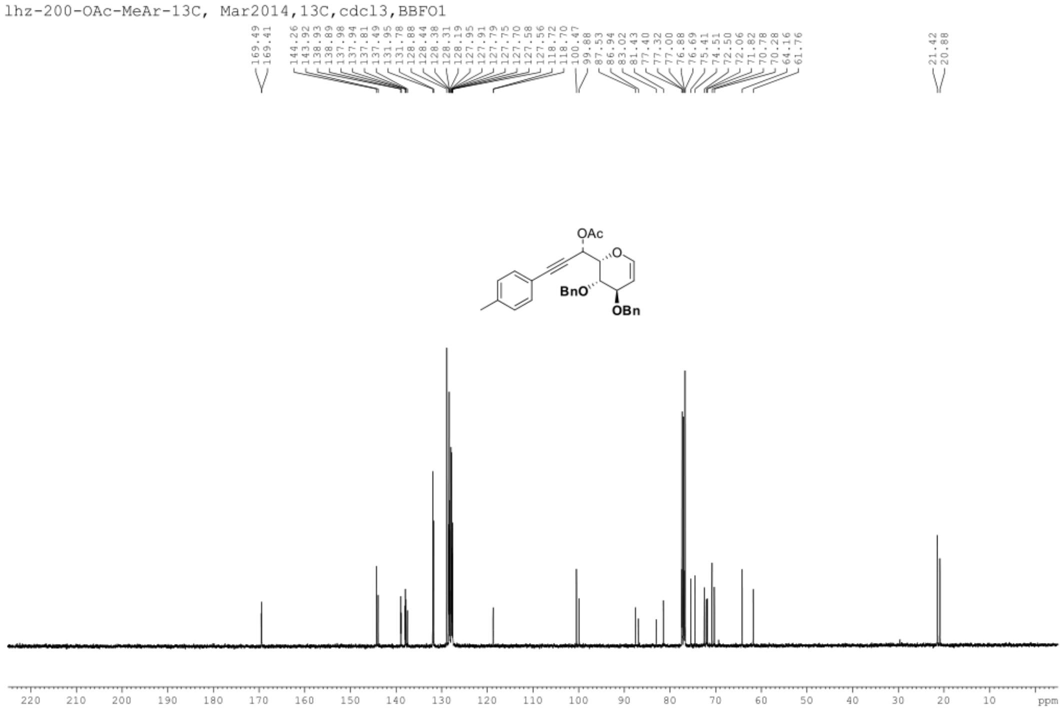

Supplementary Figure 11. ^{1}H and ^{13}C NMR spectrum for 1c.

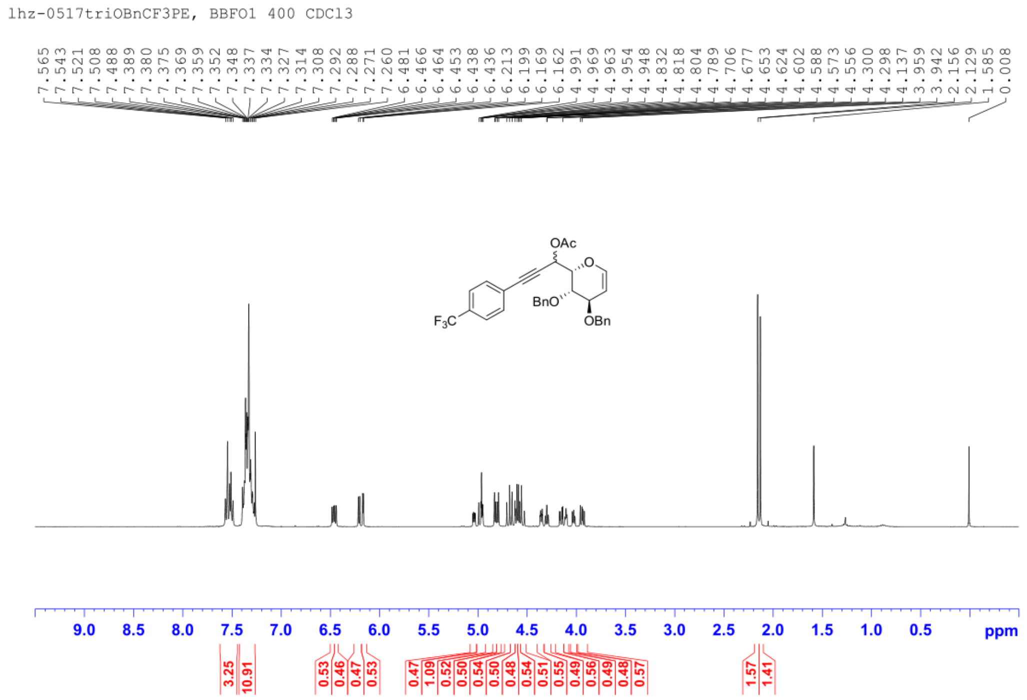

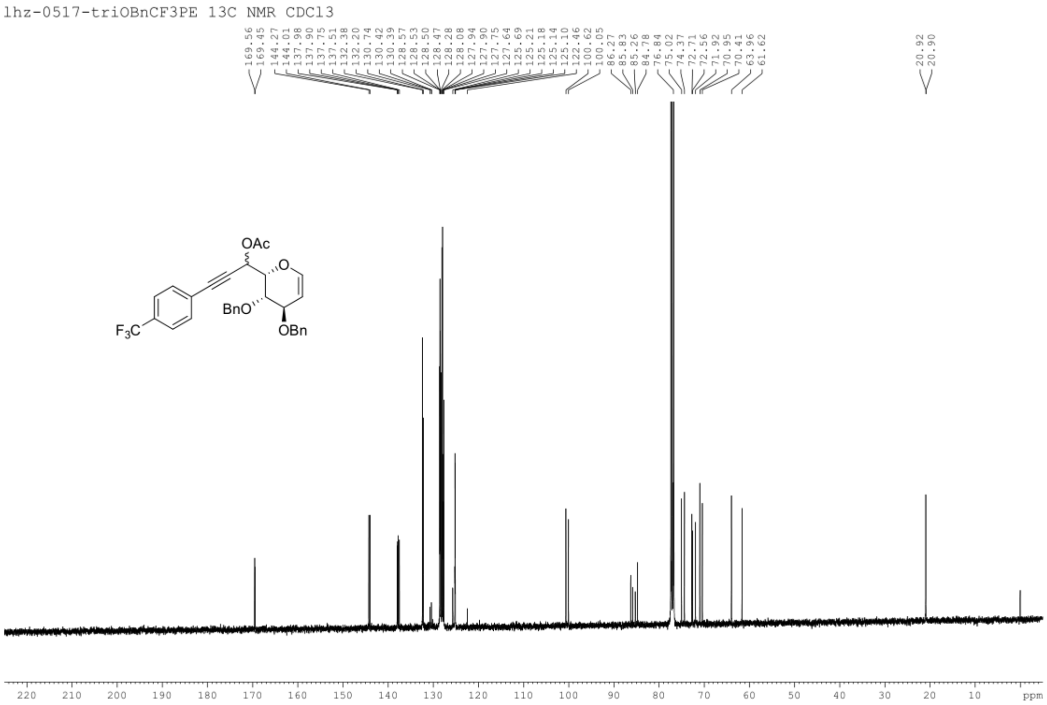

Supplementary Figure 12. ^{1}H and ^{13}C NMR spectrum for 1d.

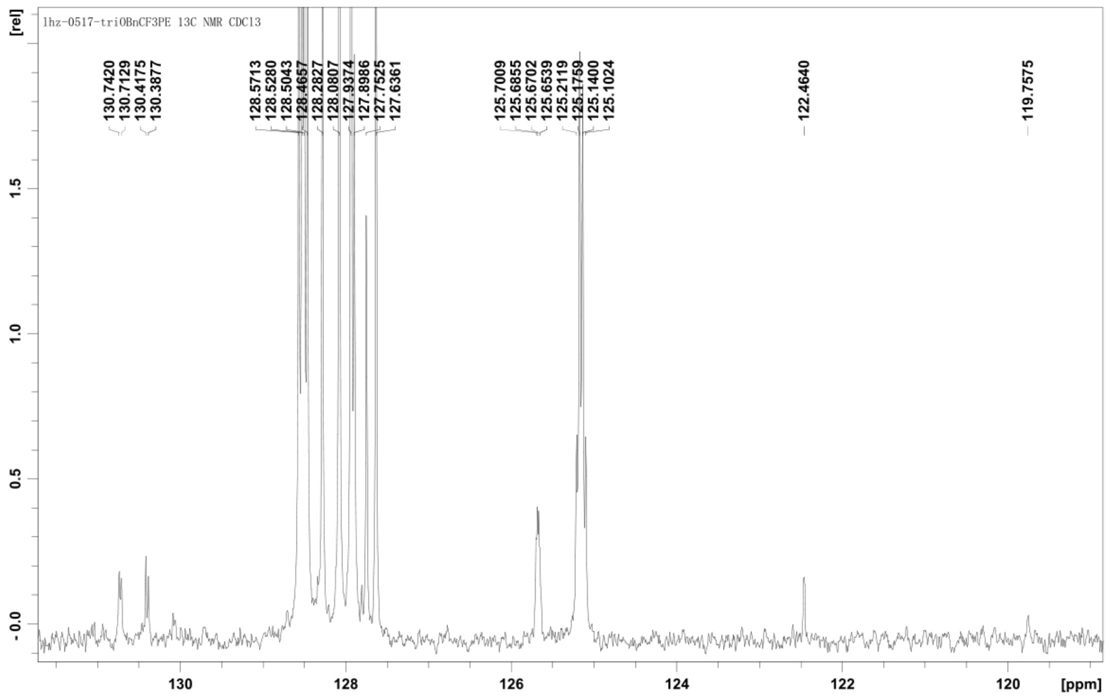

Supplementary Figure 13. ^{19}F NMR spectrum for 1d.

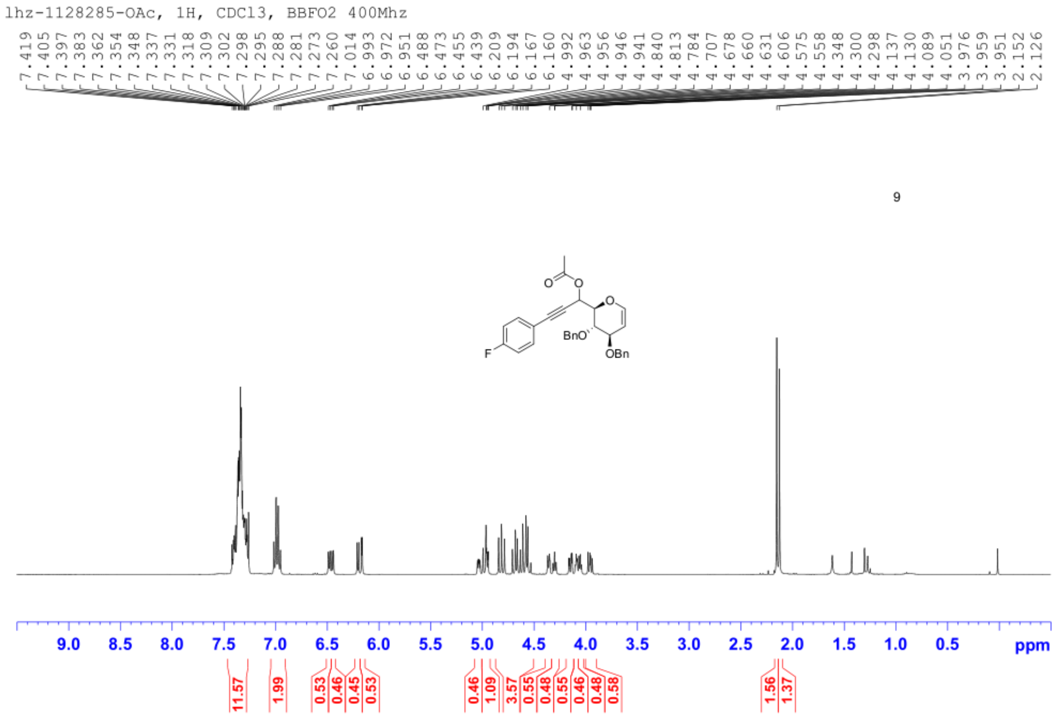

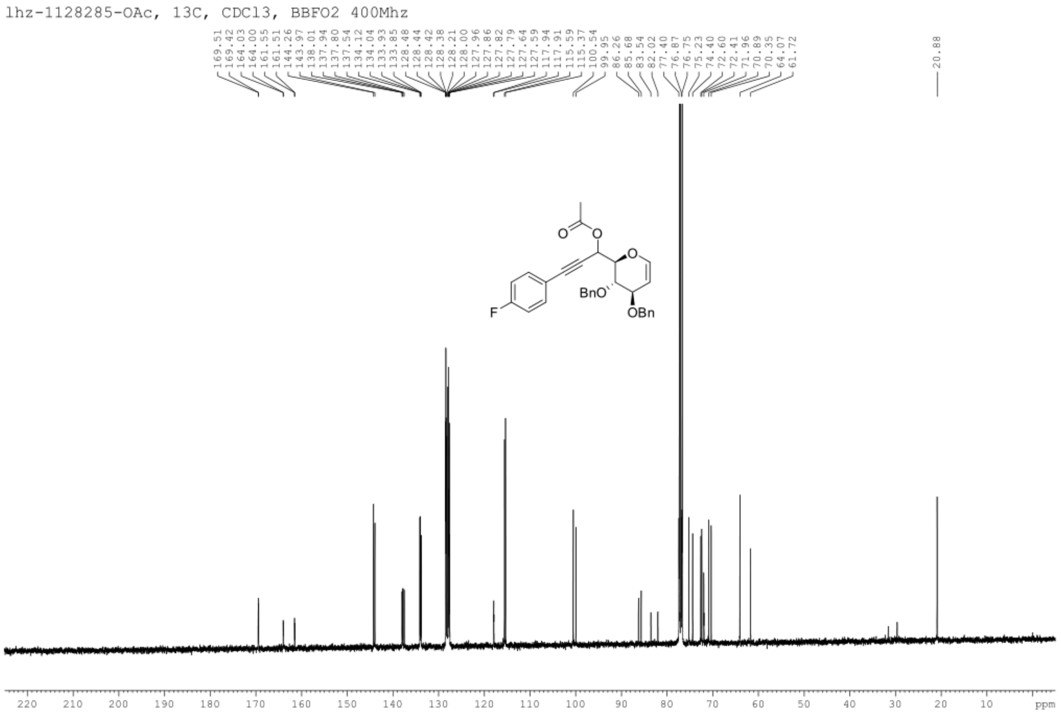

Supplementary Figure 14. ^{1}H and ^{13}C NMR spectrum for 1e.

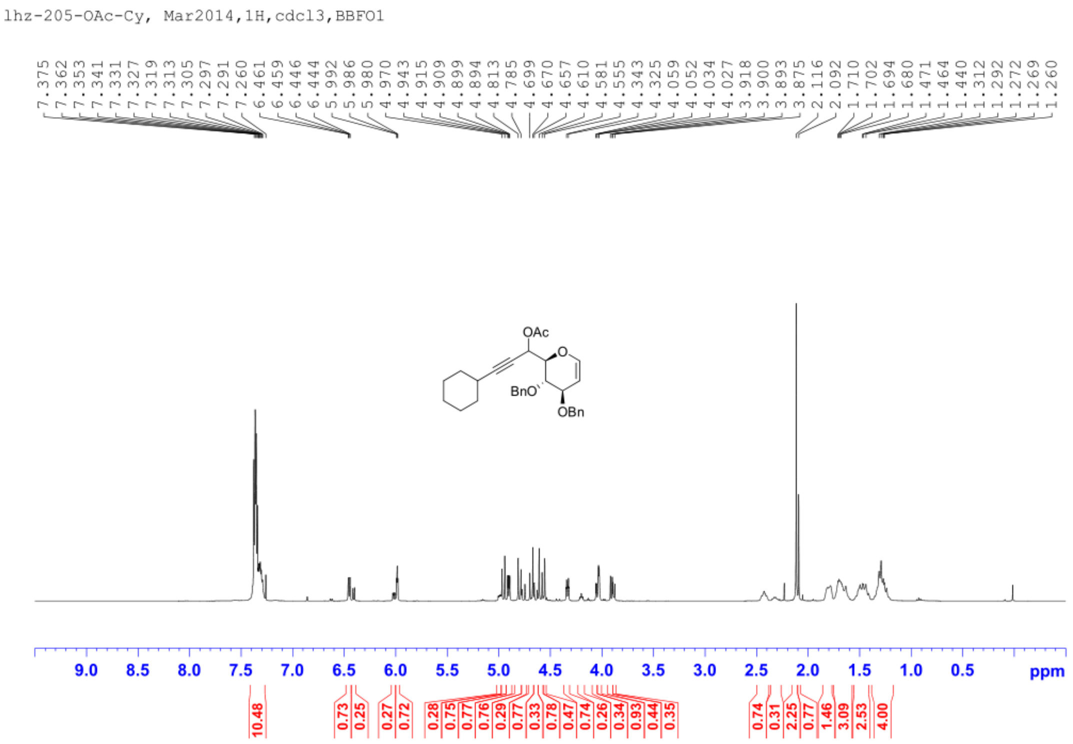

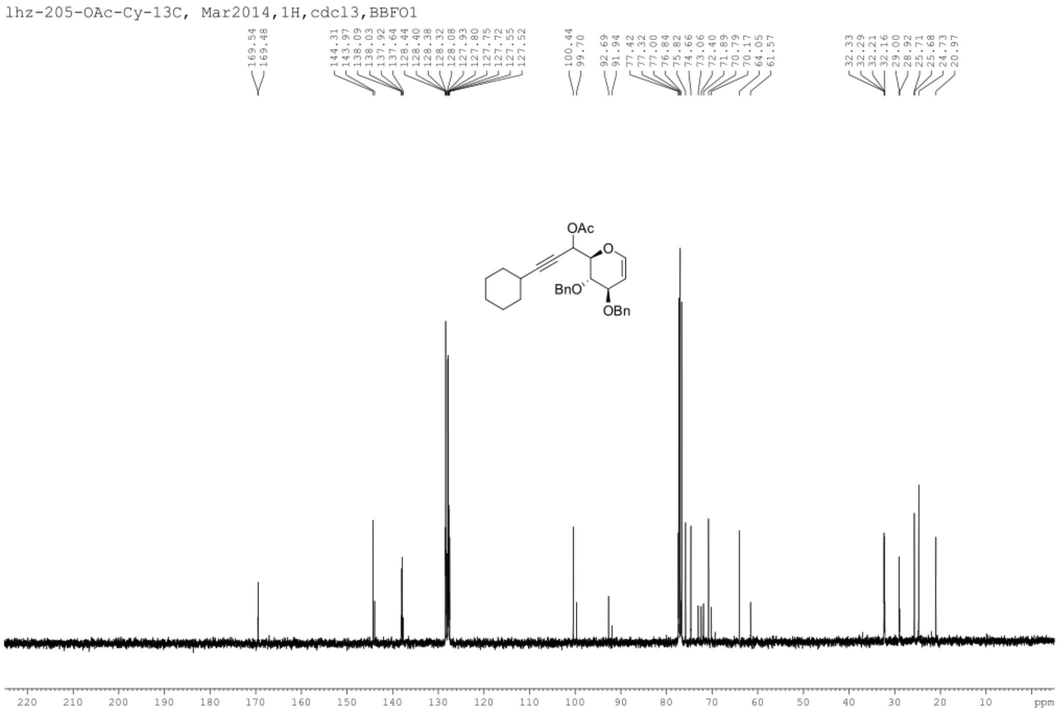

Supplementary Figure 15. ^{1}H and ^{13}C NMR spectrum for 1f.

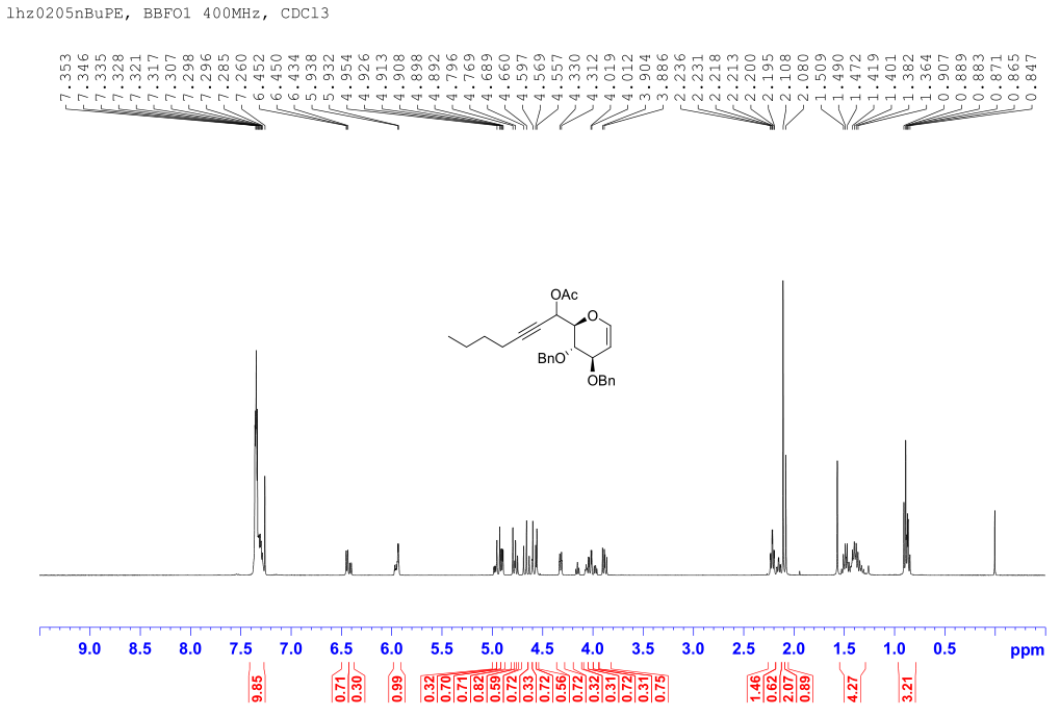

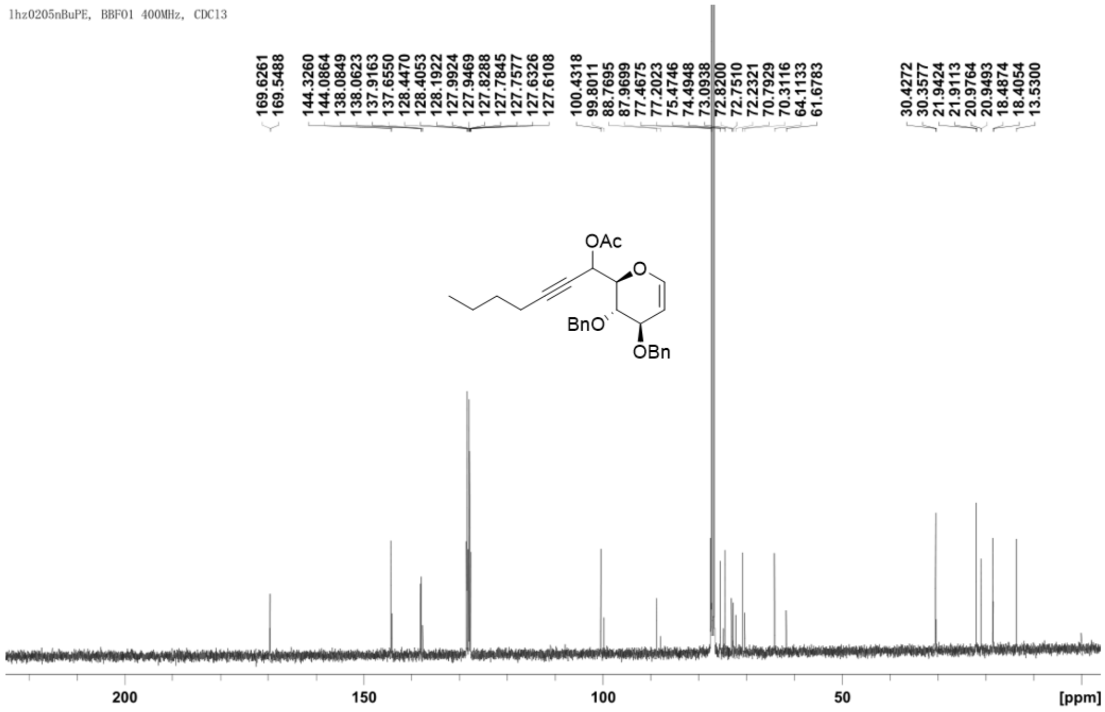

Supplementary Figure 16. ^{1}H and ^{13}C NMR spectrum for 1g.

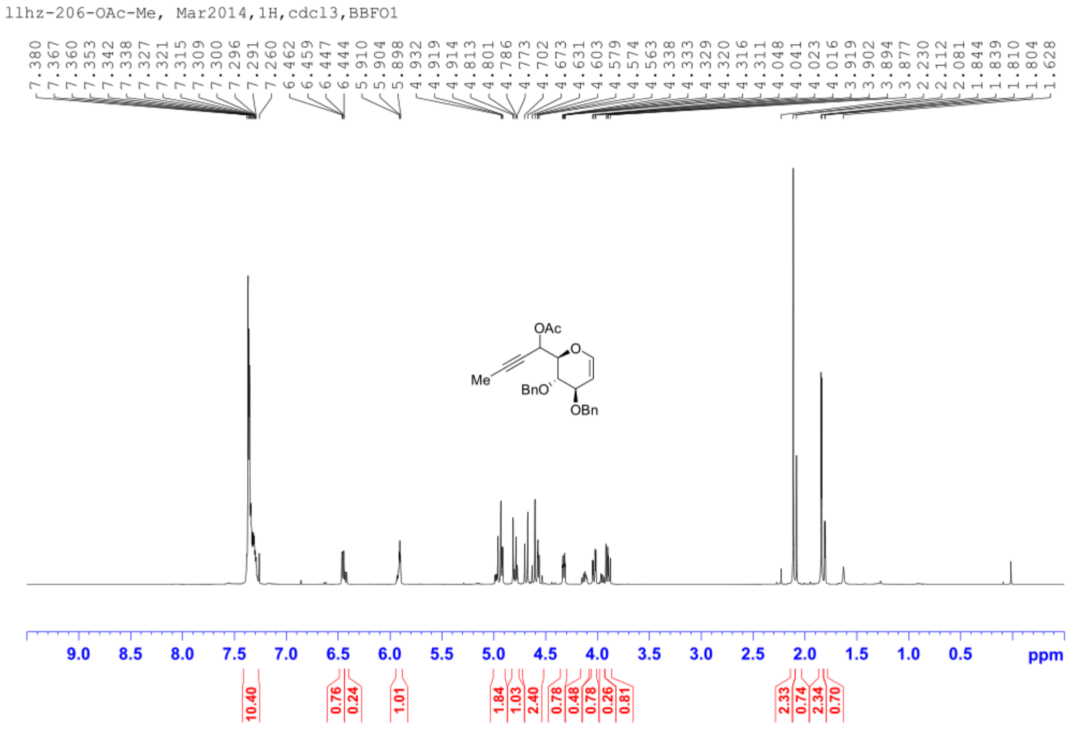

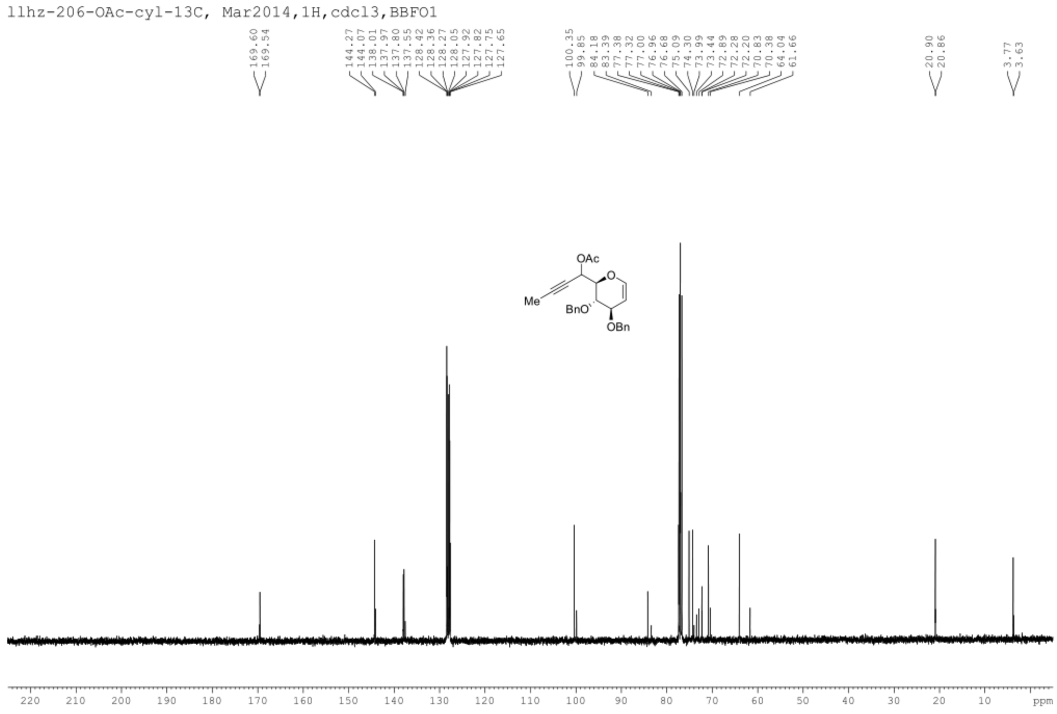

Supplementary Figure 17. ^{1}H and ^{13}C NMR spectrum for 1h.

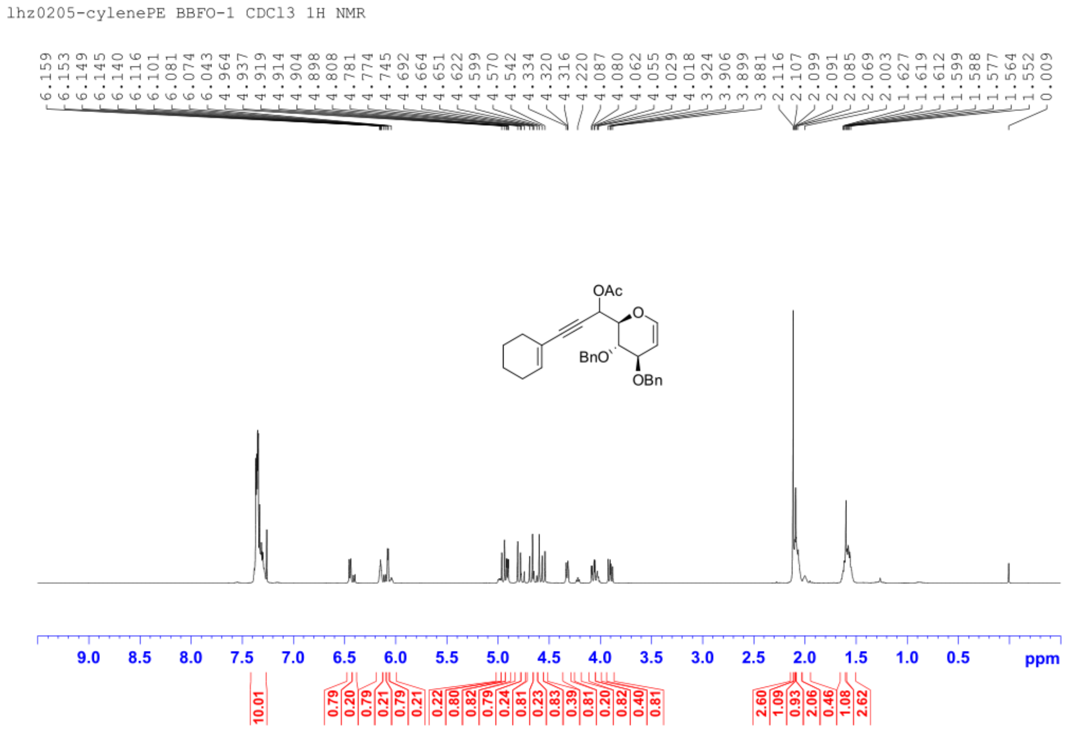

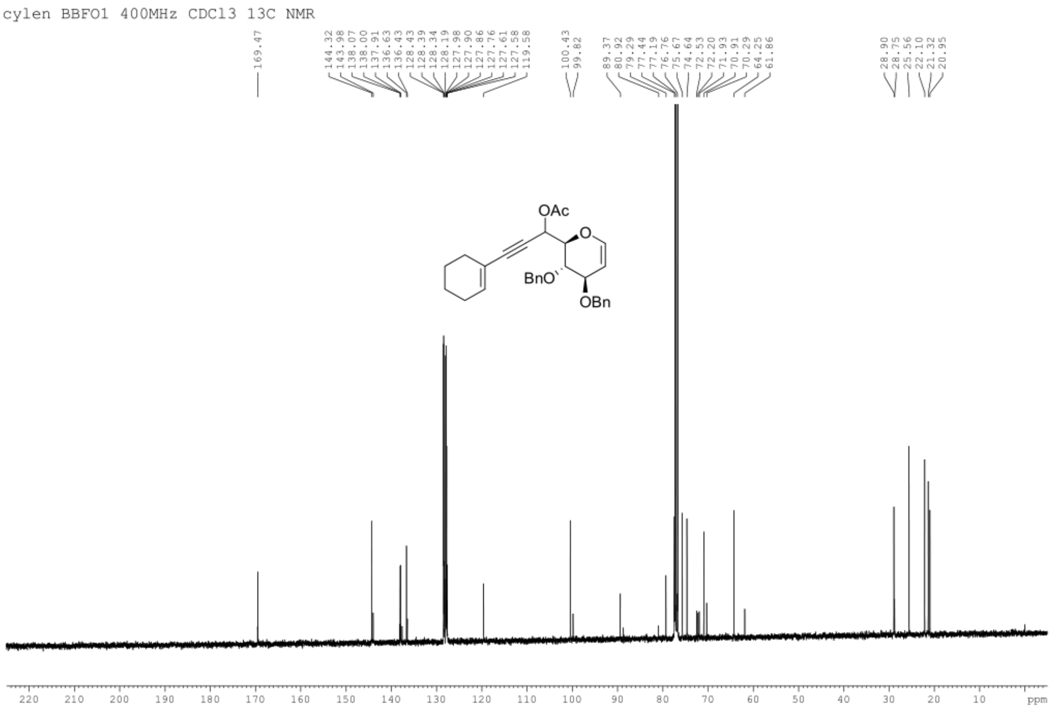

Supplementary Figure 18. ^{1}H and ^{13}C NMR spectrum for 1i.

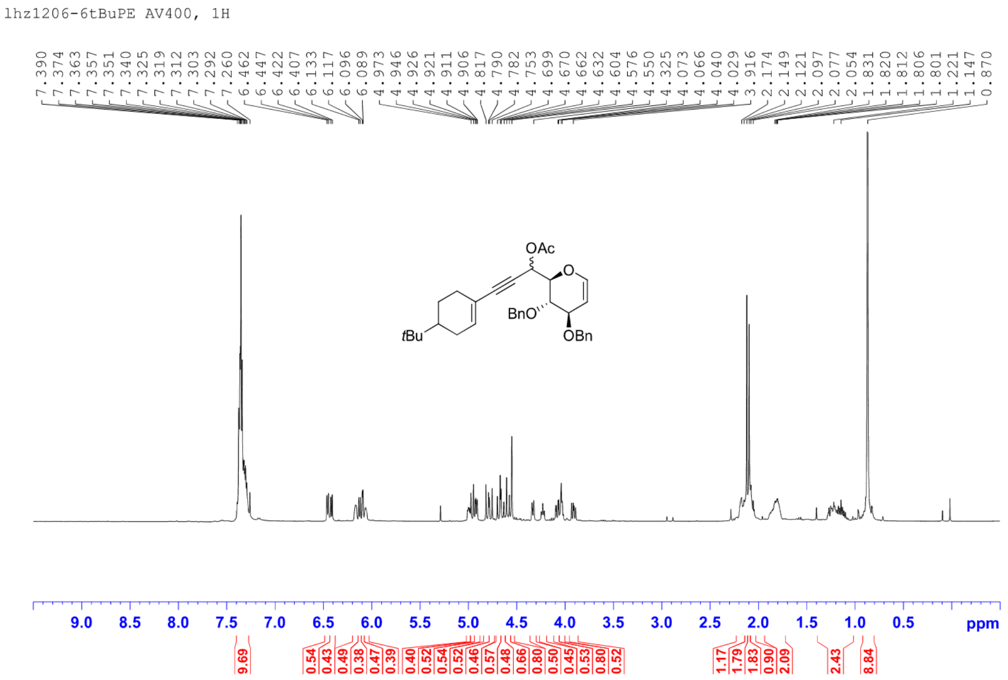

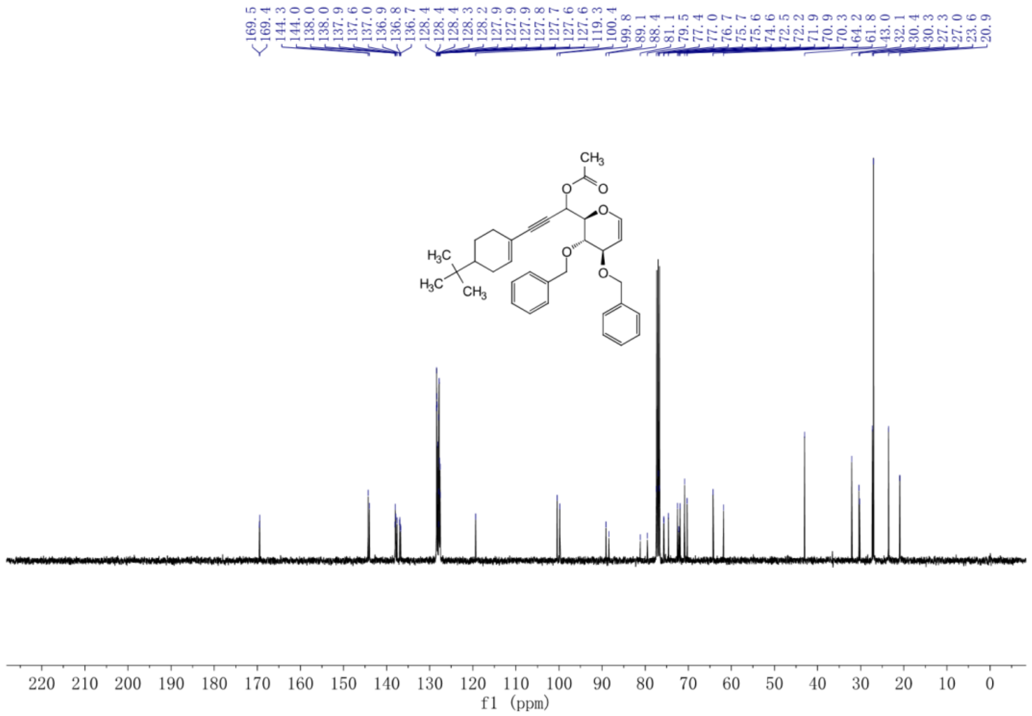

Supplementary Figure 19. ^{1}H and ^{13}C NMR spectrum for 1j.

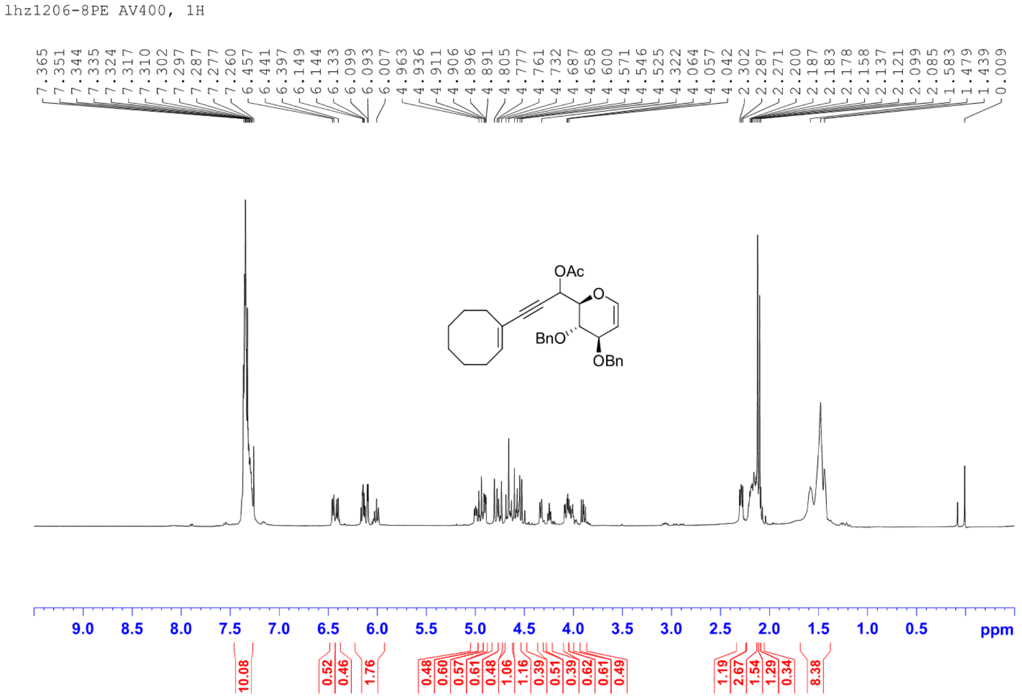

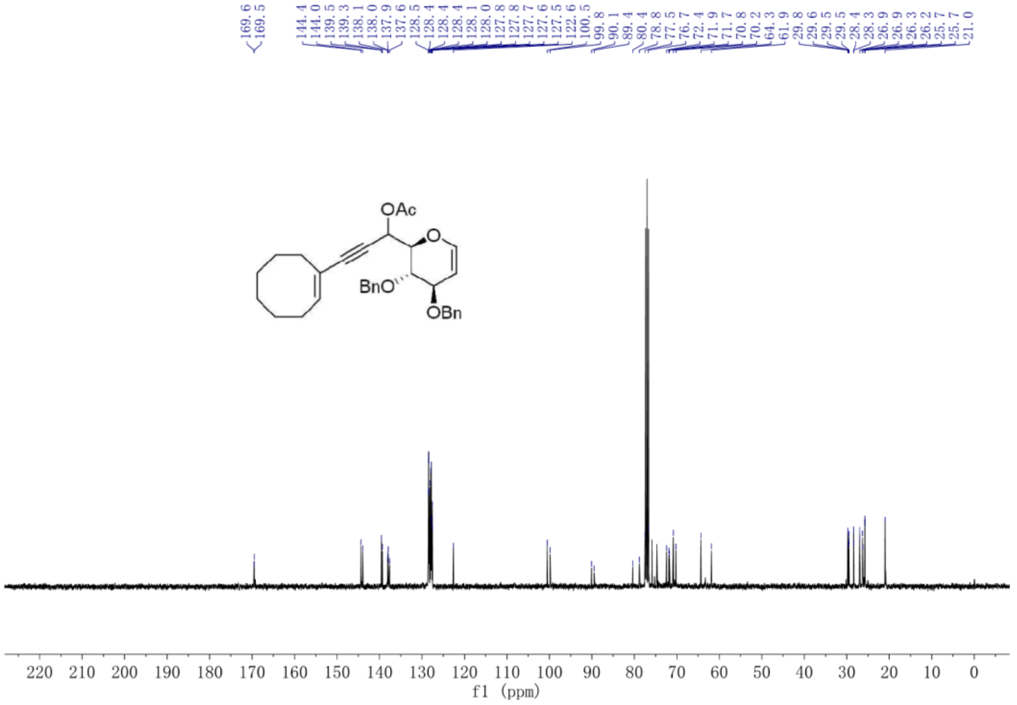

Supplementary Figure 20. ^{1}H and ^{13}C NMR spectrum for 1k.

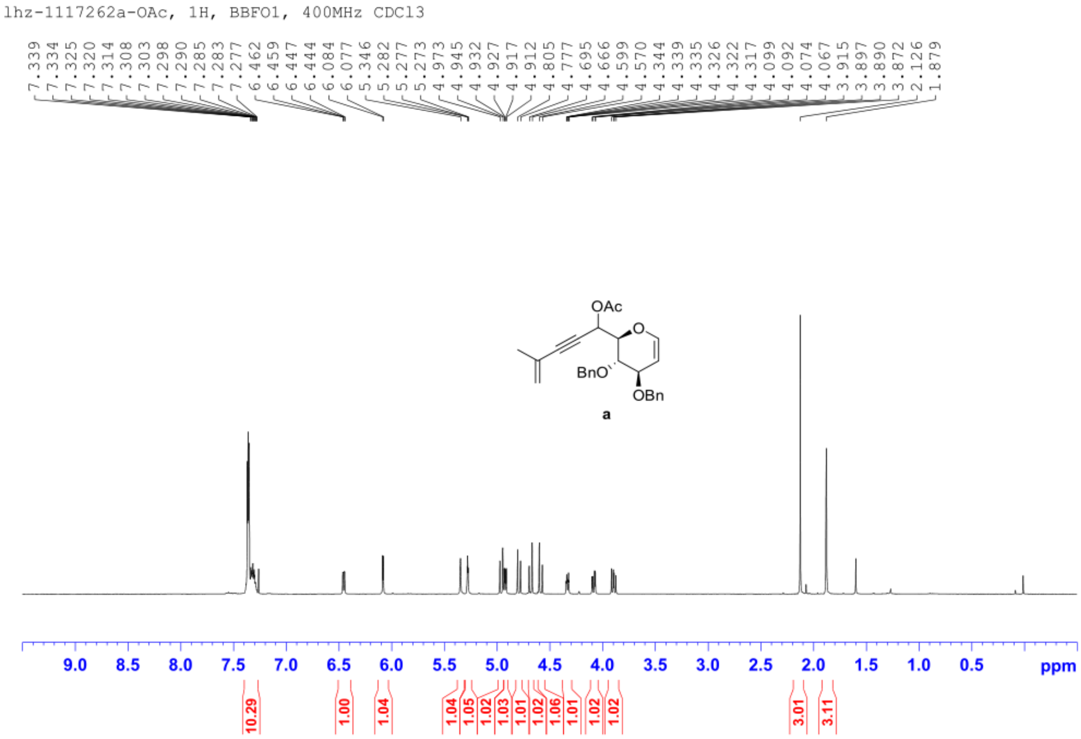

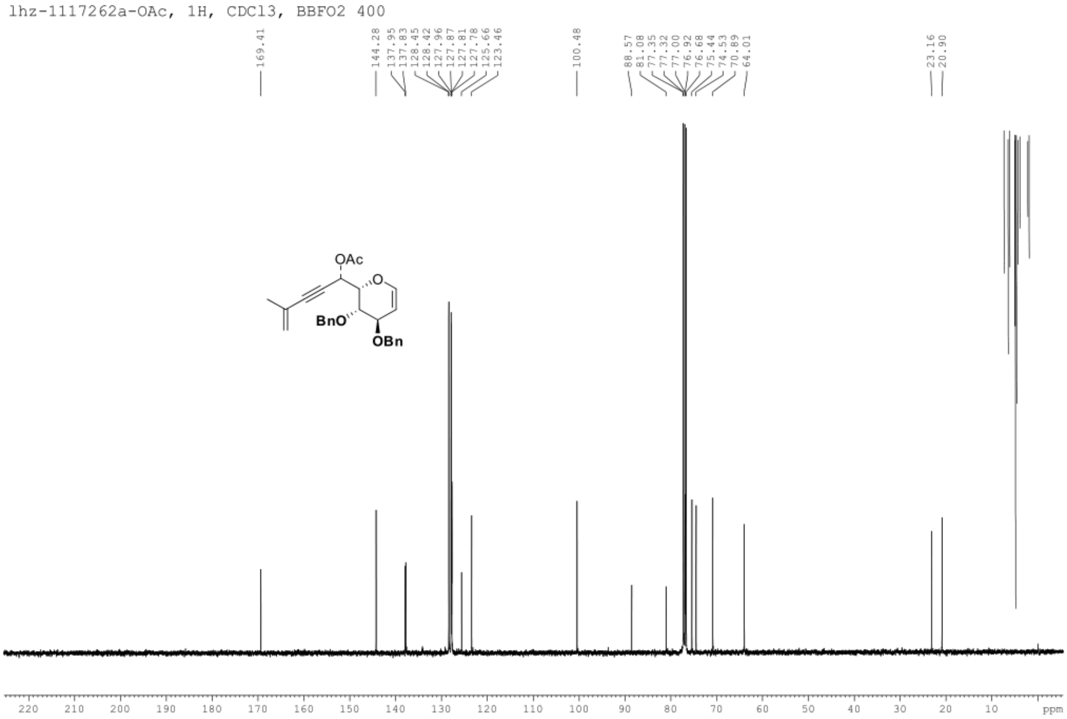

Supplementary Figure 21. ^{1}H and ^{13}C NMR spectrum for 1l-A.

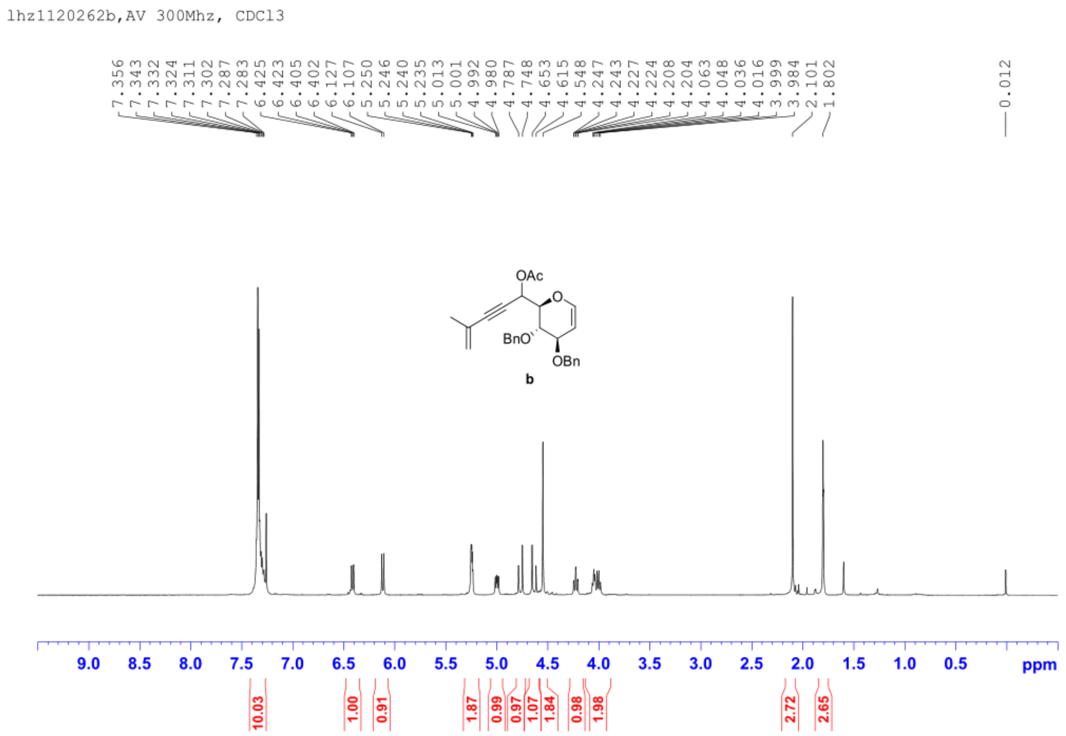

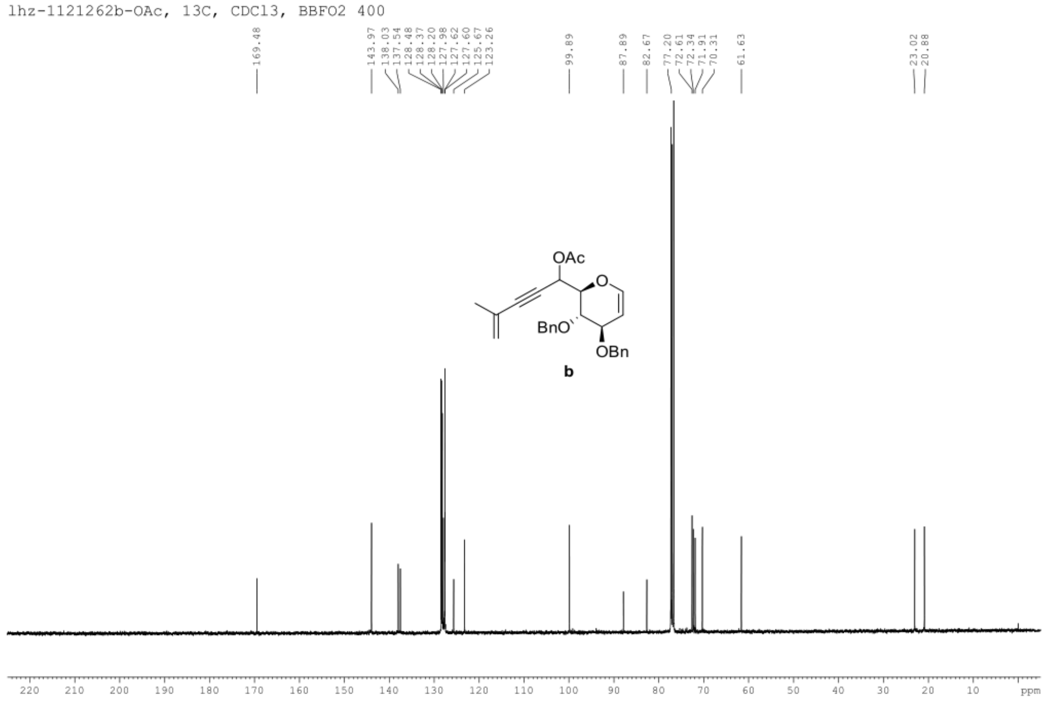

Supplementary Figure 22. ^{1}H and ^{13}C NMR spectrum for 1l-B.

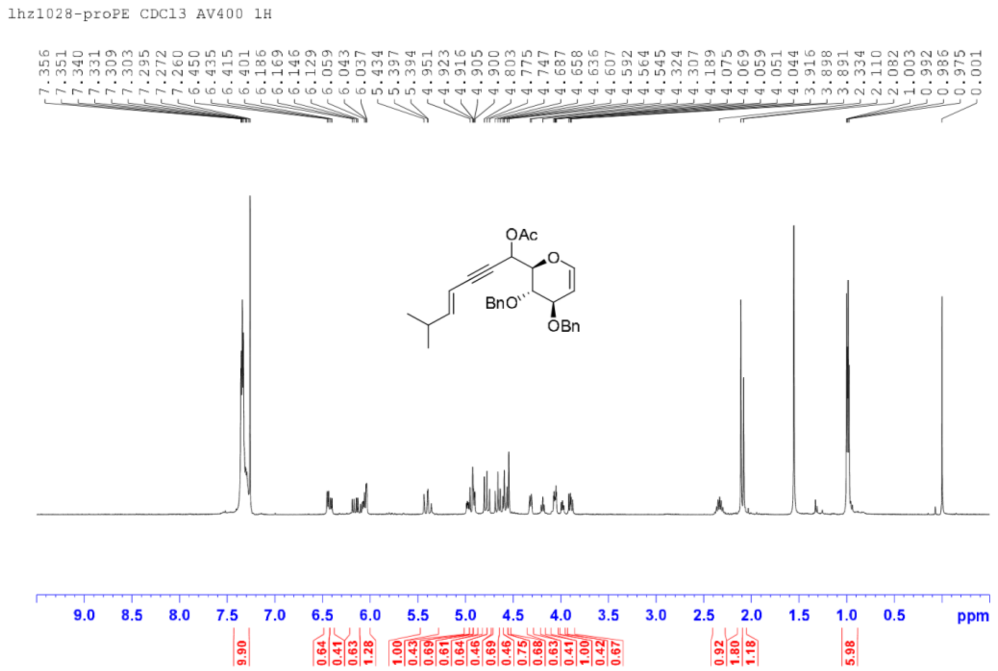

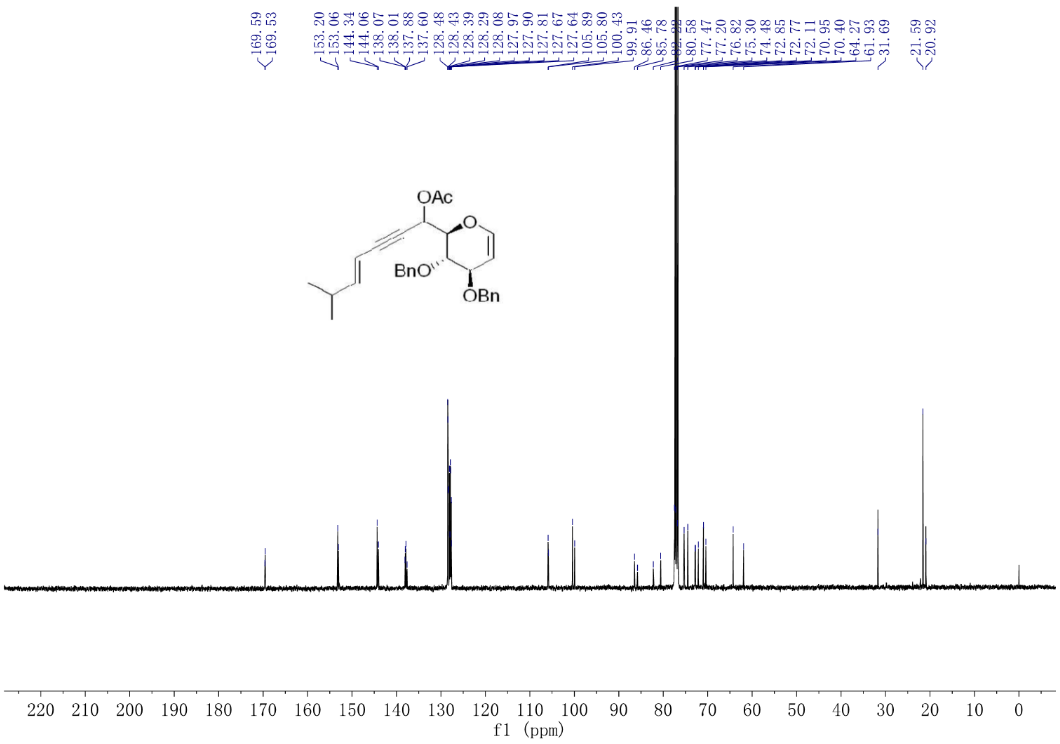

Supplementary Figure 23. ^{1}H and ^{13}C NMR spectrum for 1m.

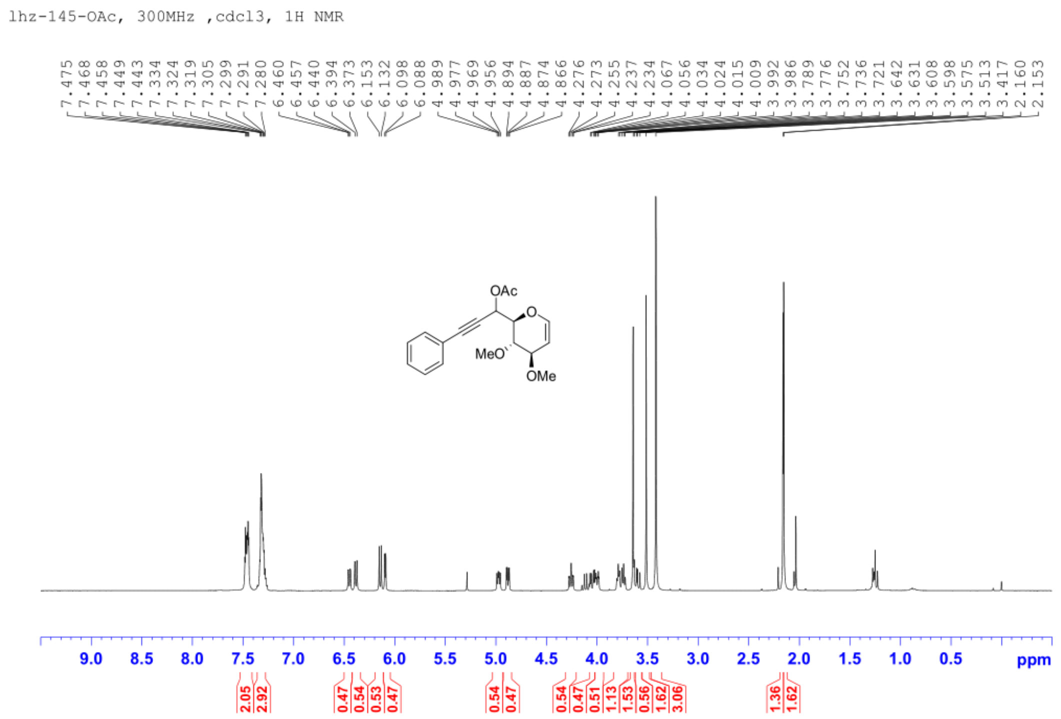

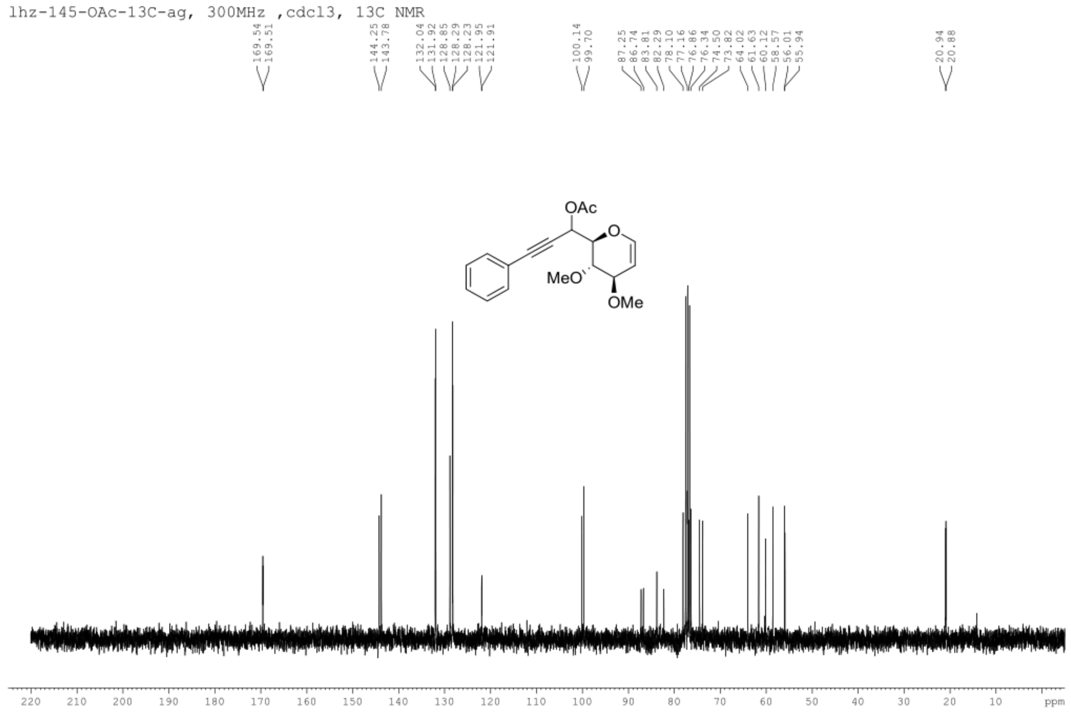

Supplementary Figure 24. ^{1}**H and** ^{13}**C NMR spectrum for 1n.**

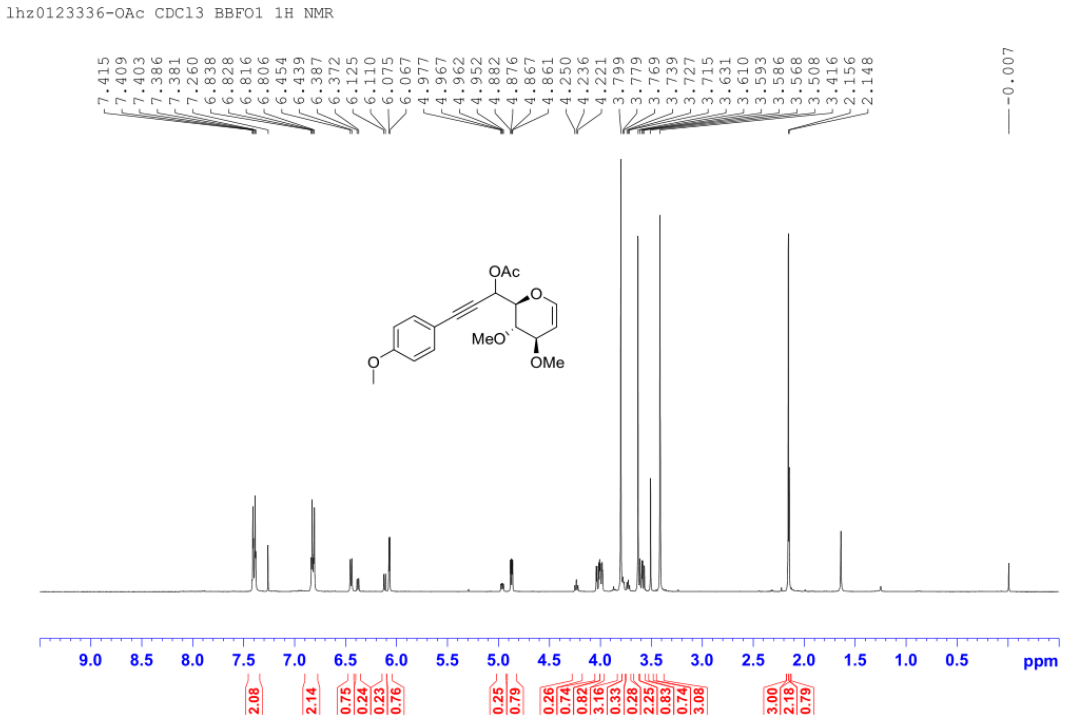

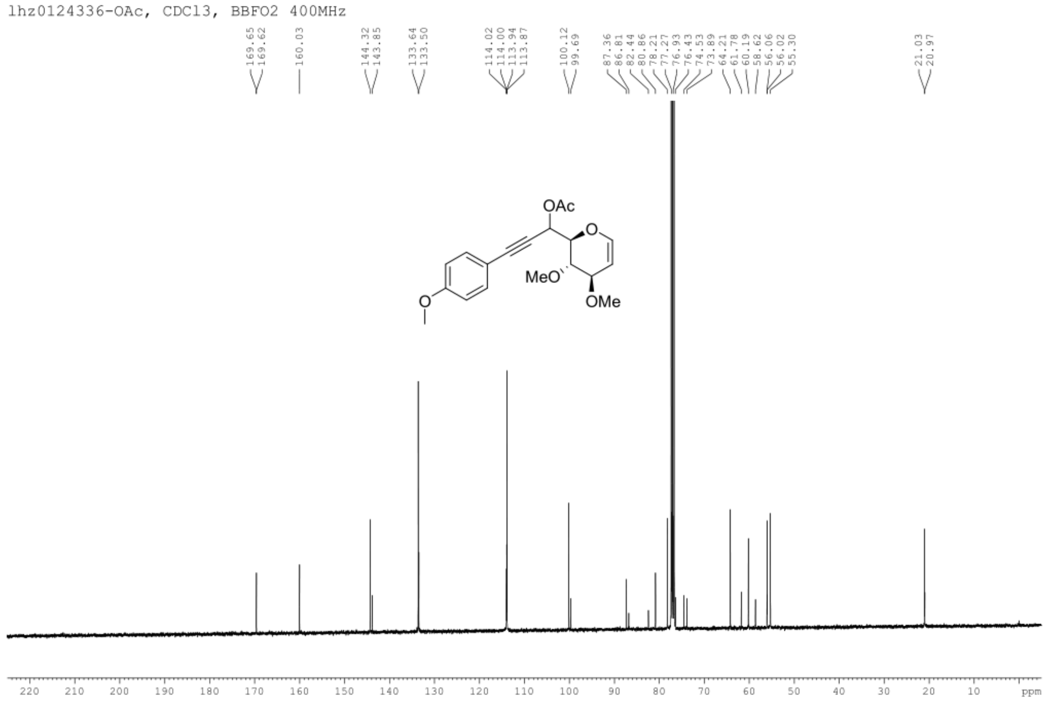

Supplementary Figure 25. ^{1}H and ^{13}C NMR spectrum for 1o.

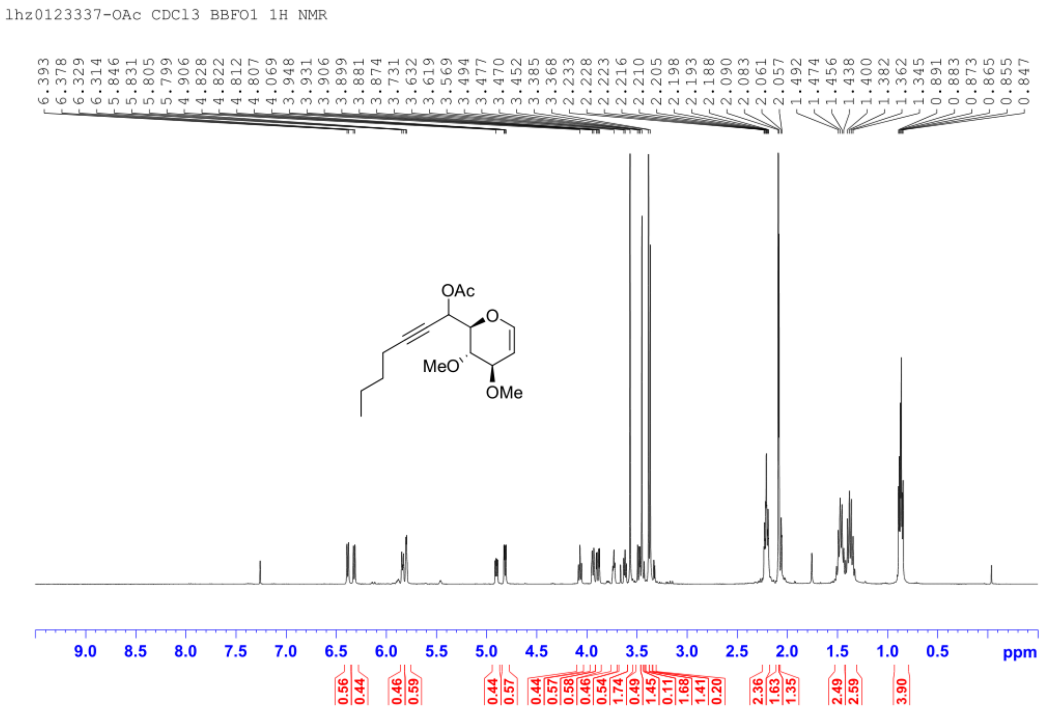

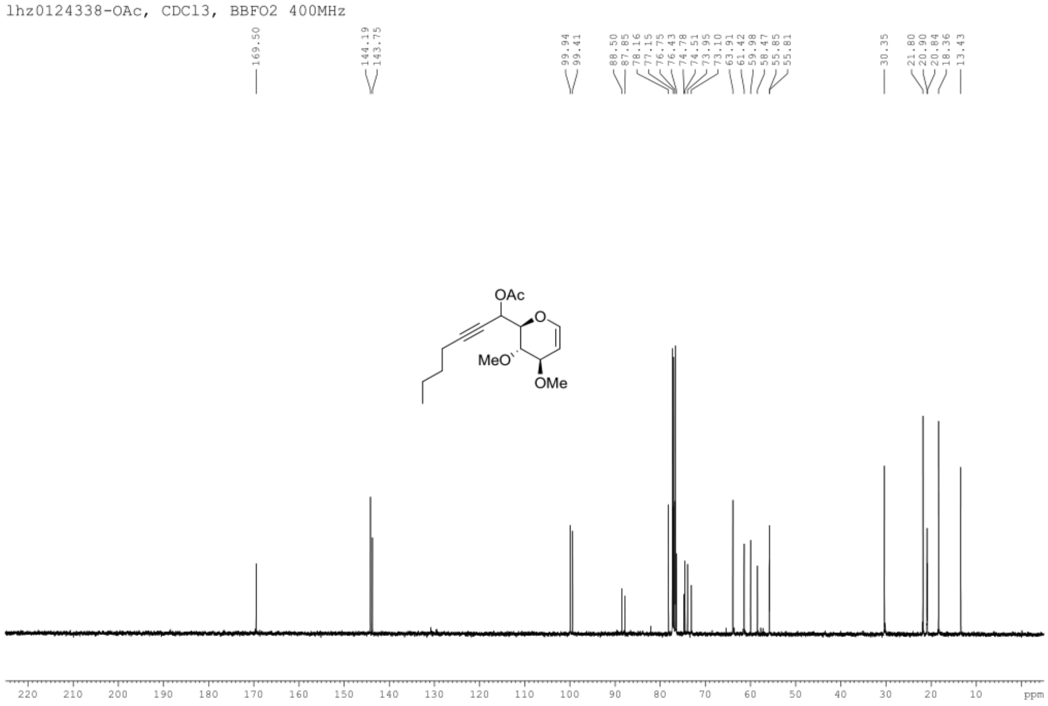

Supplementary Figure 26. ^{1}H and ^{13}C NMR spectrum for 1p.

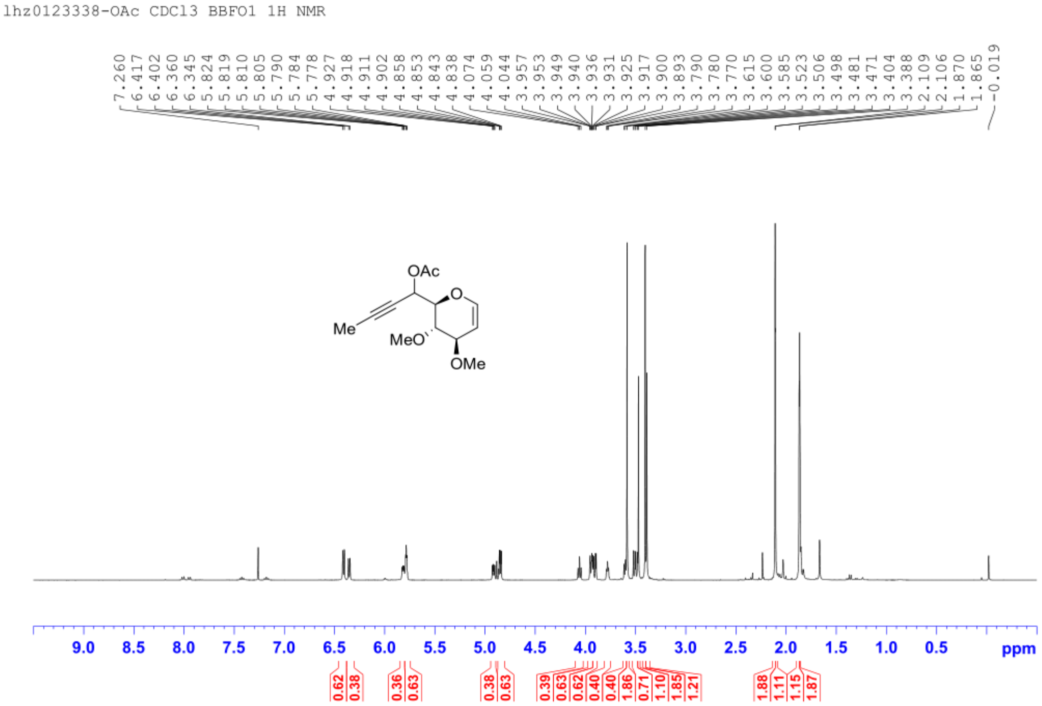

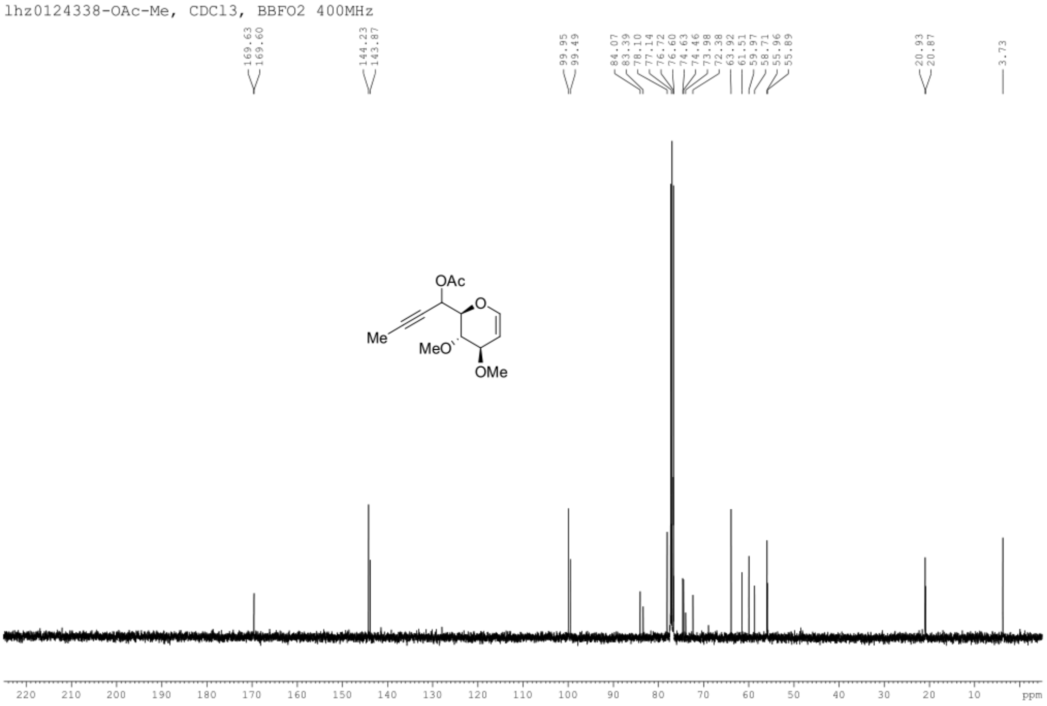

Supplementary Figure 27. ^{1}H and ^{13}C NMR spectrum for 1q.

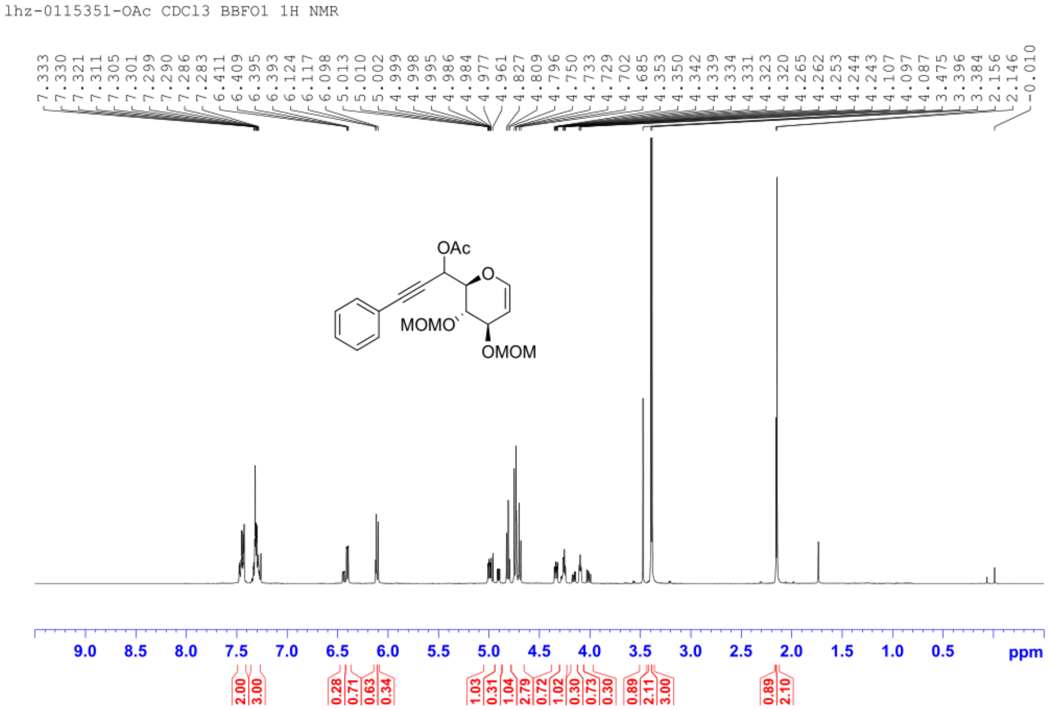

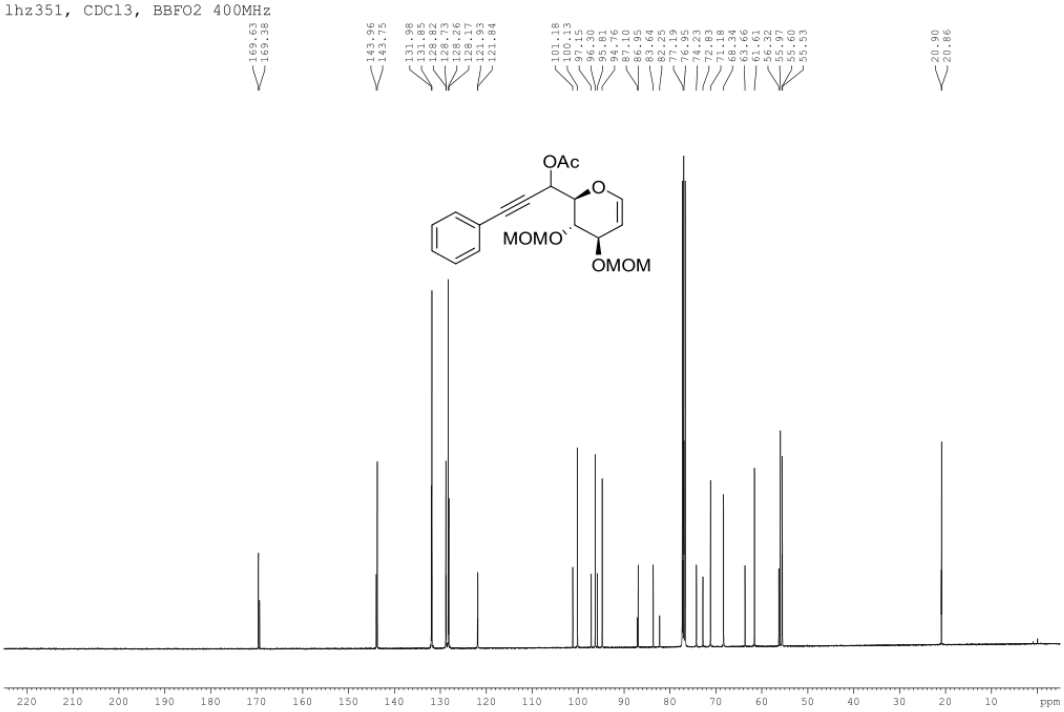

Supplementary Figure 28. ^{1}H and ^{13}C NMR spectrum for 1r.

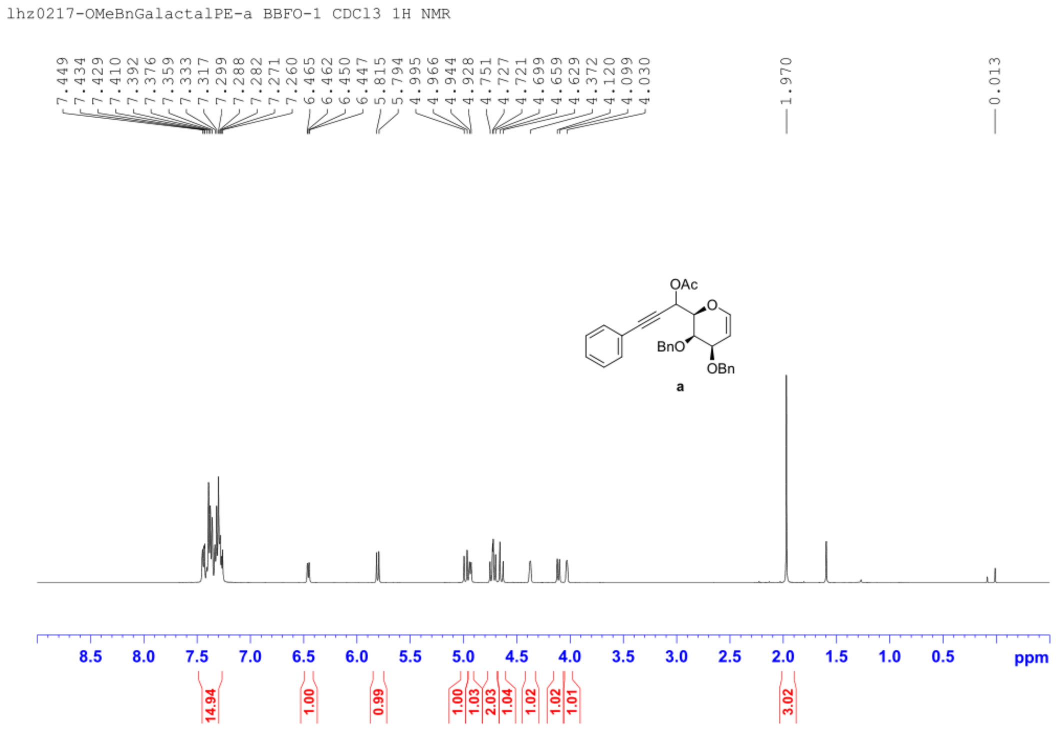

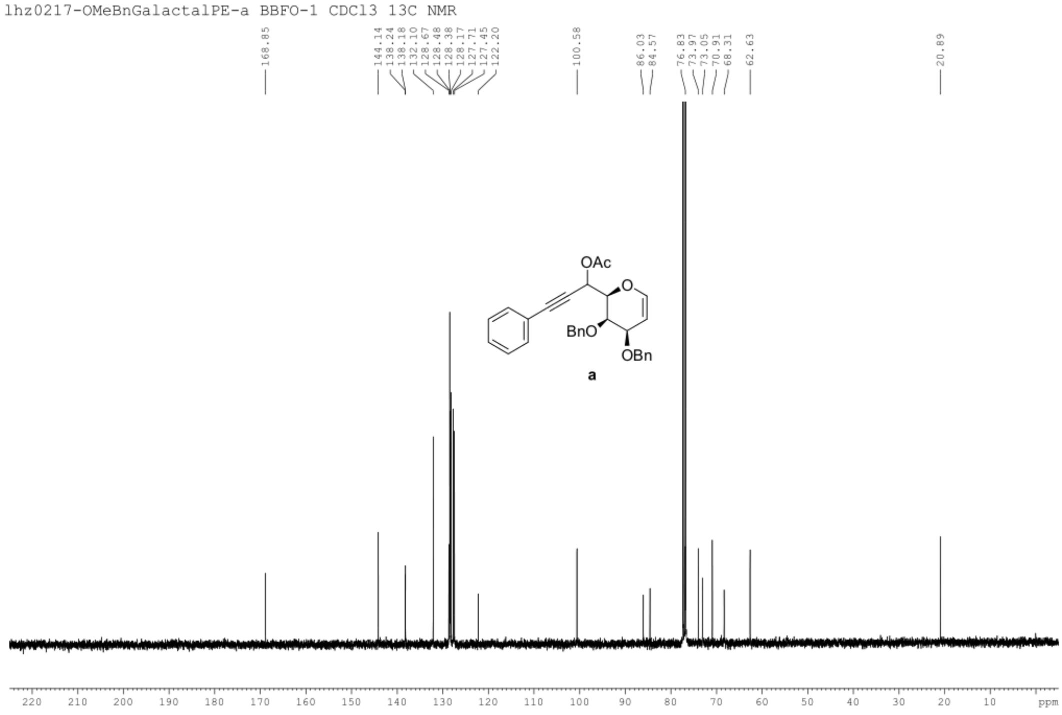

Supplementary Figure 29. ^{1}H and ^{13}C NMR spectrum for *epi*-1a-A.

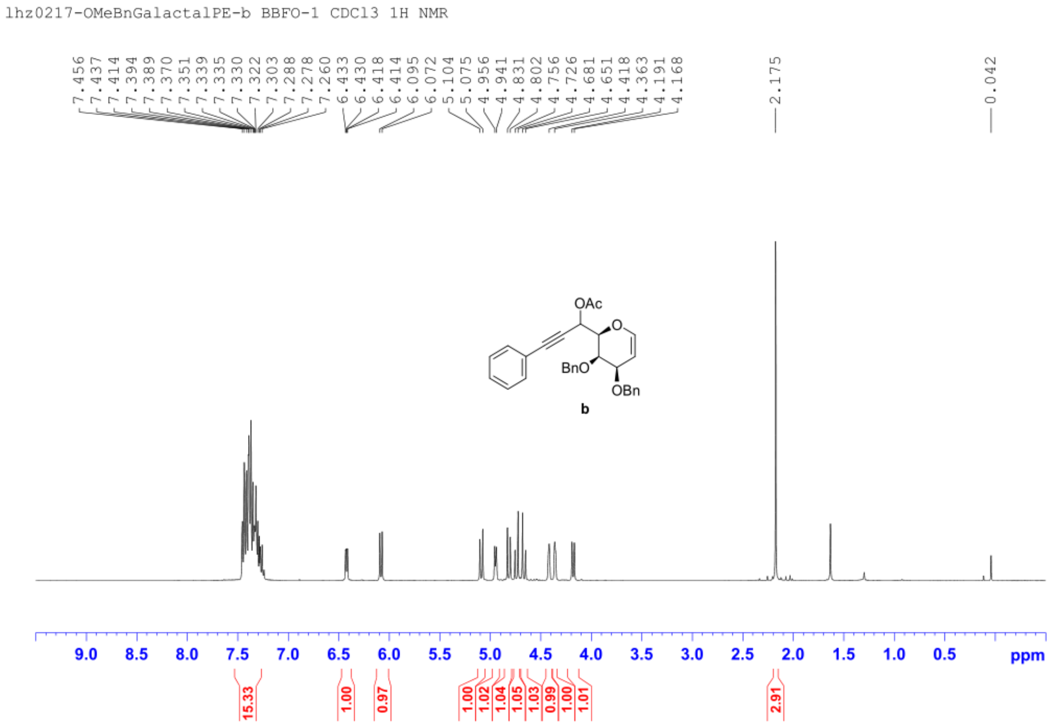

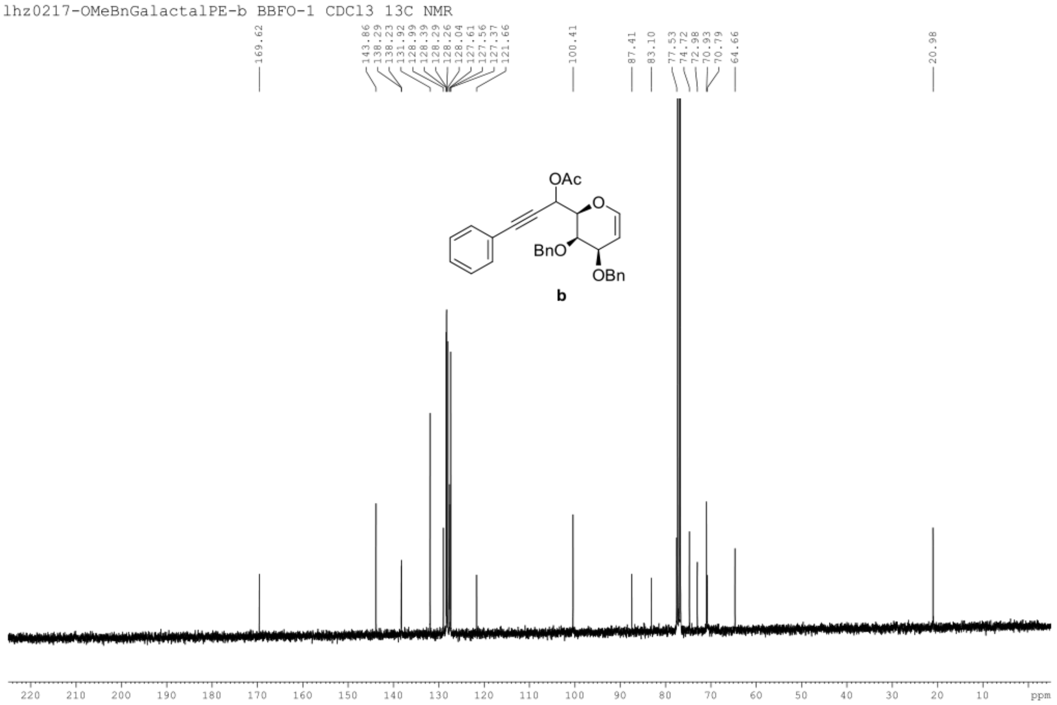

Supplementary Figure 30. ^{1}H and ^{13}C NMR spectrum for *epi*-1a-B.

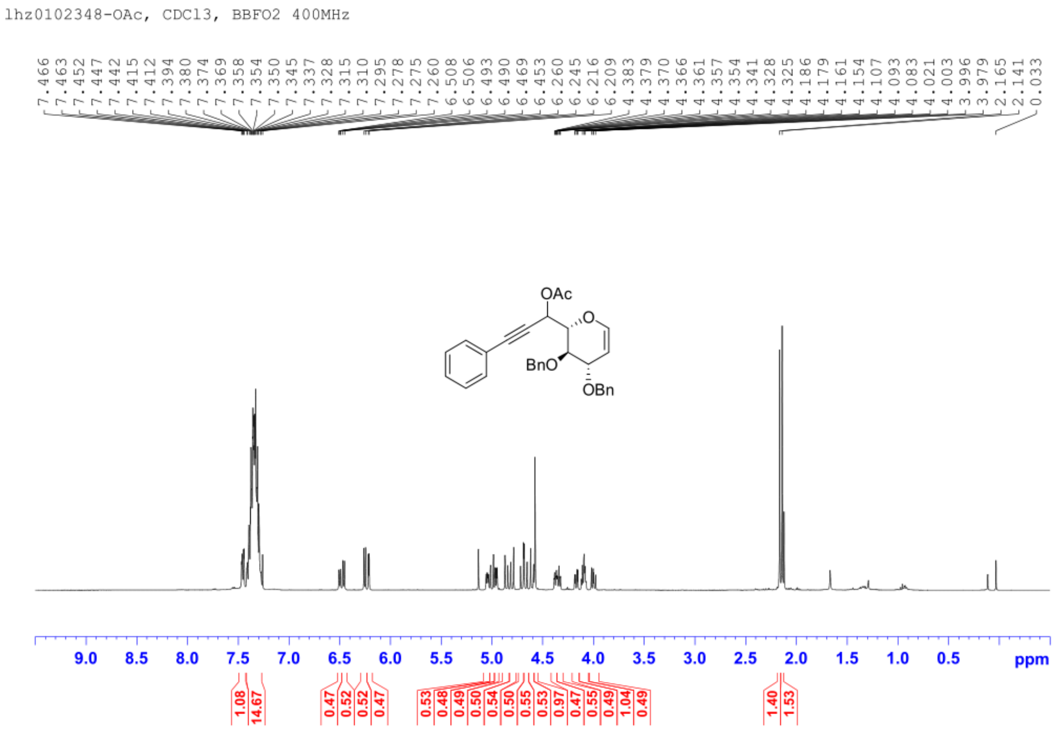

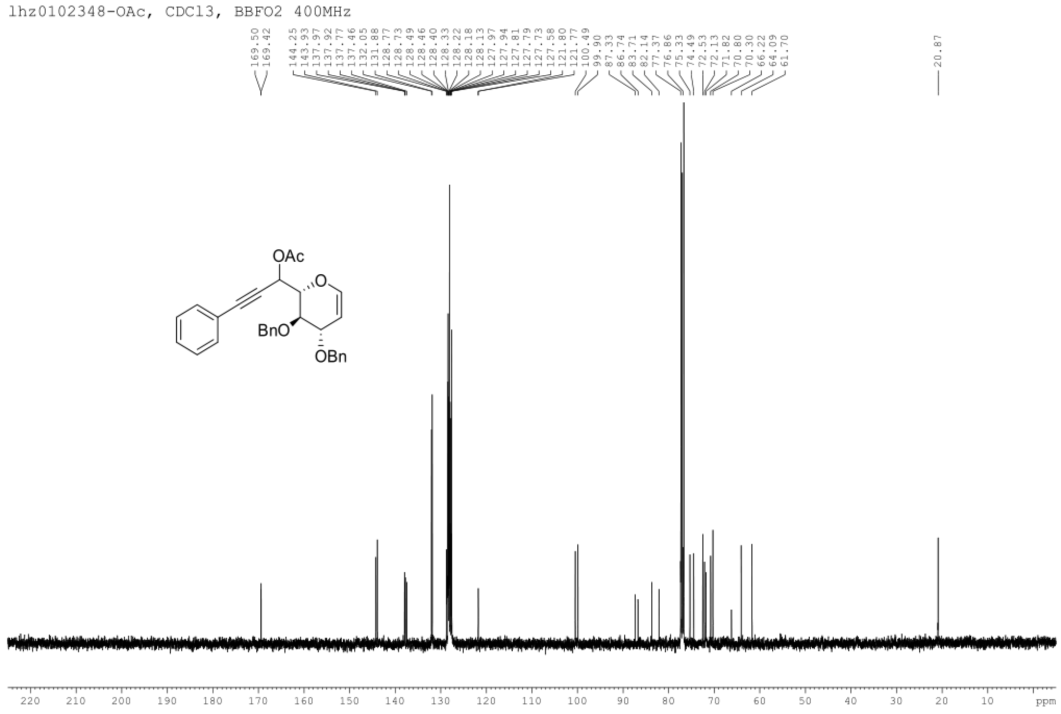

Supplementary Figure 31. ^{1}H and ^{13}C NMR spectrum for *ent*-1a.

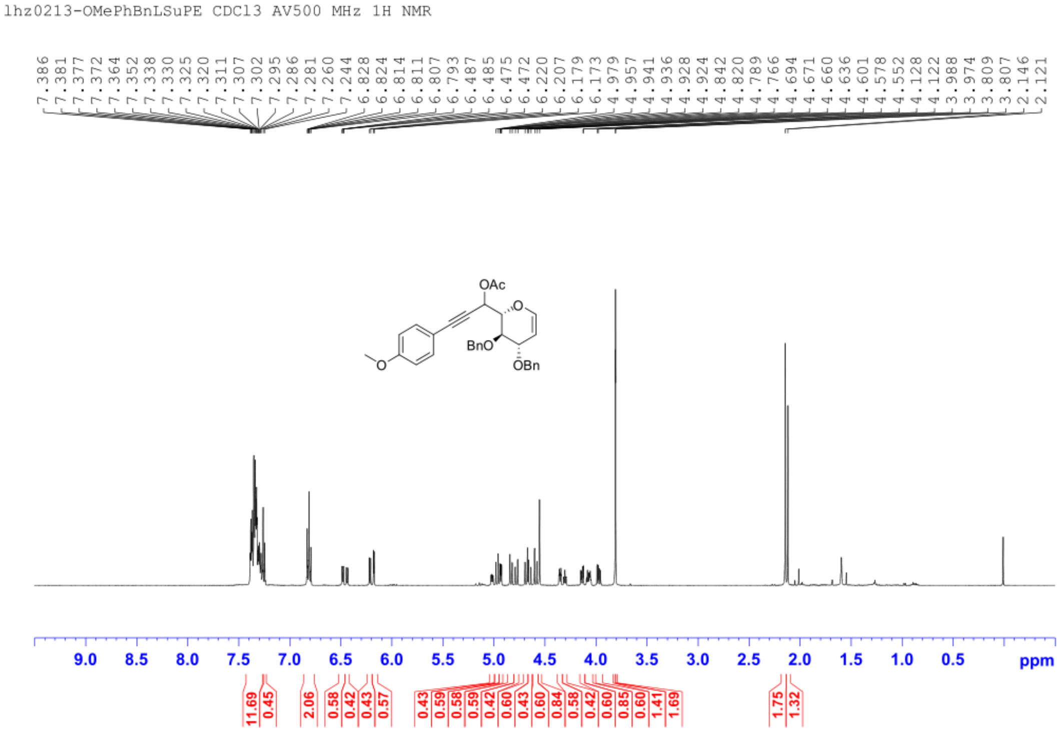

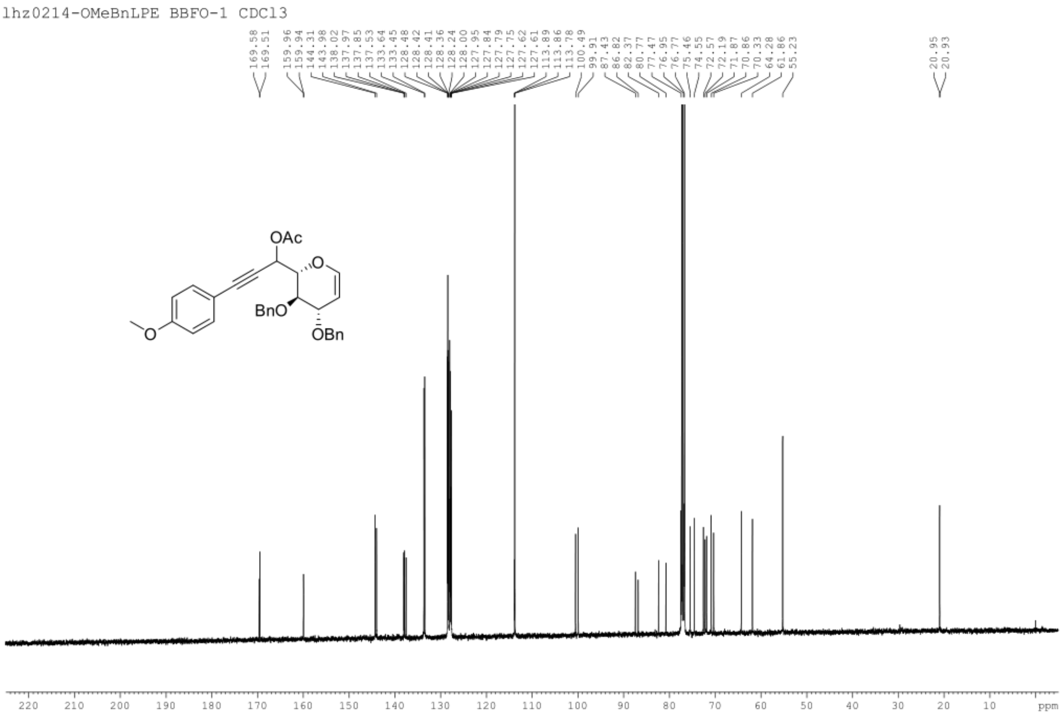

Supplementary Figure 32. ^{1}H and ^{13}C NMR spectrum for *ent*-1b.

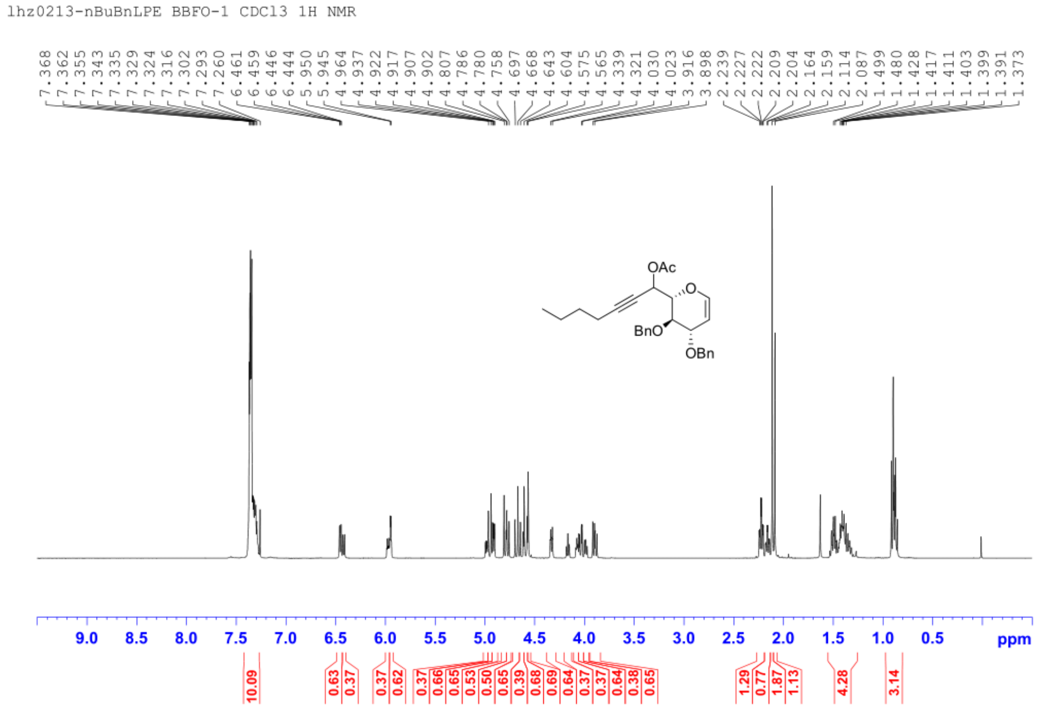

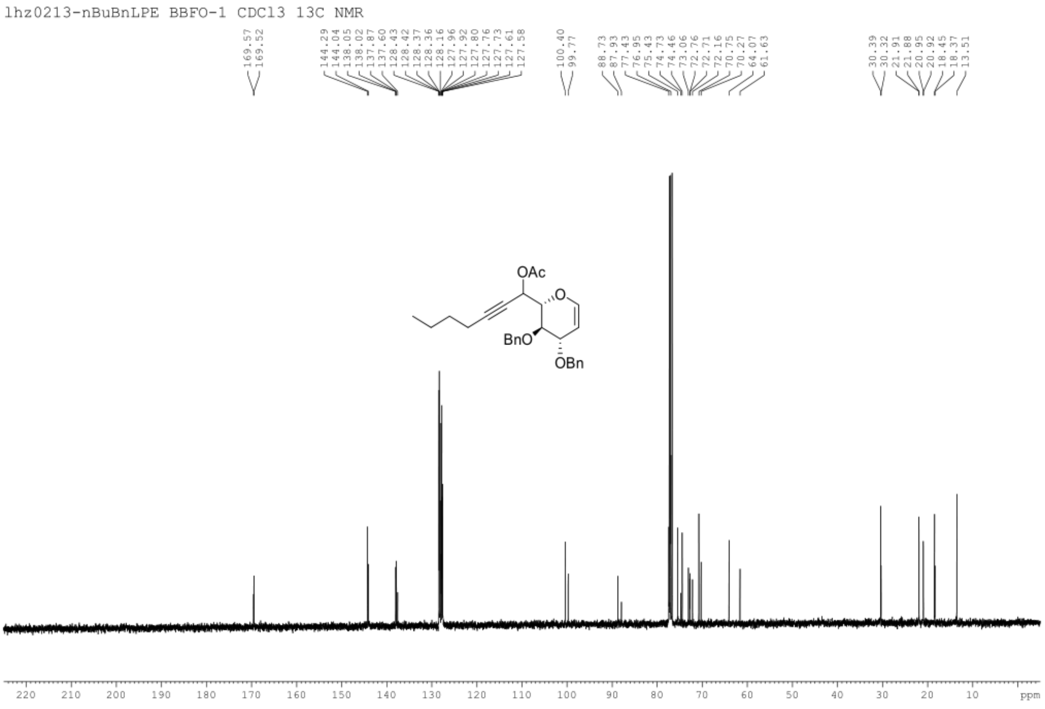

Supplementary Figure 33. ^{1}H and ^{13}C NMR spectrum for *ent*-1g.

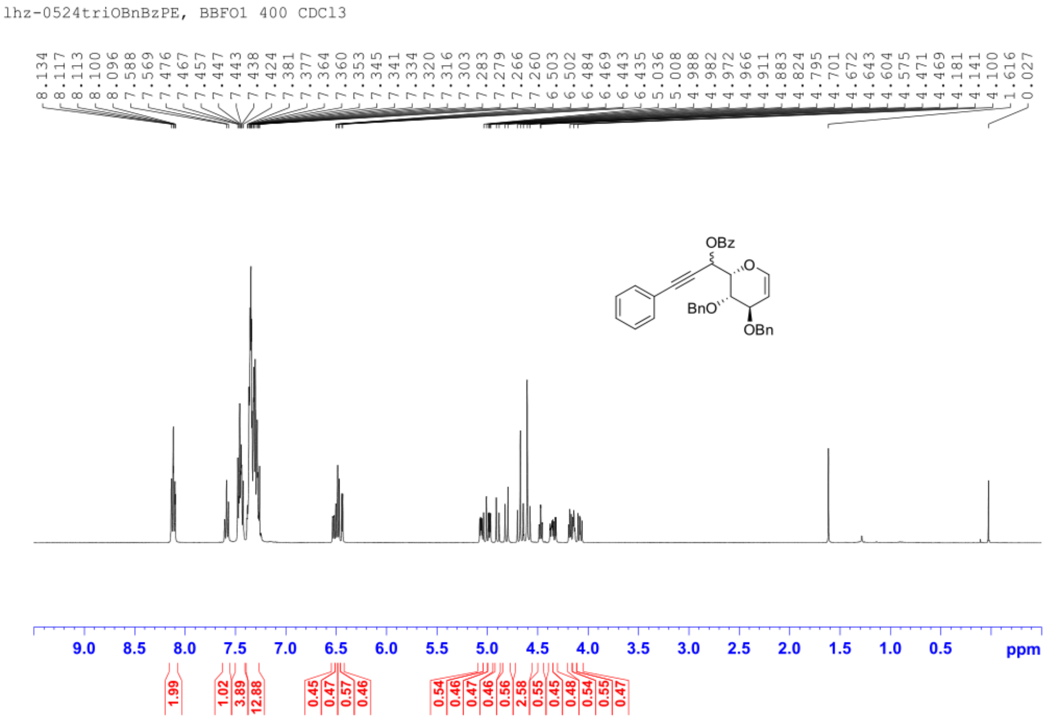

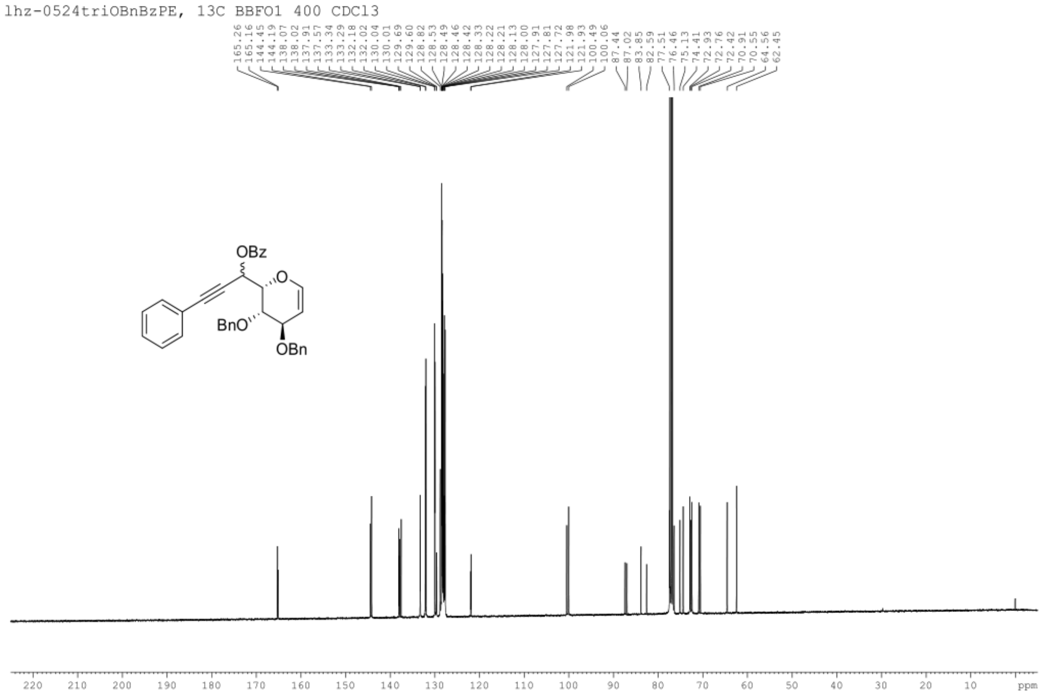

Supplementary Figure 34. ^{1}H and ^{13}C NMR spectrum for 1s.

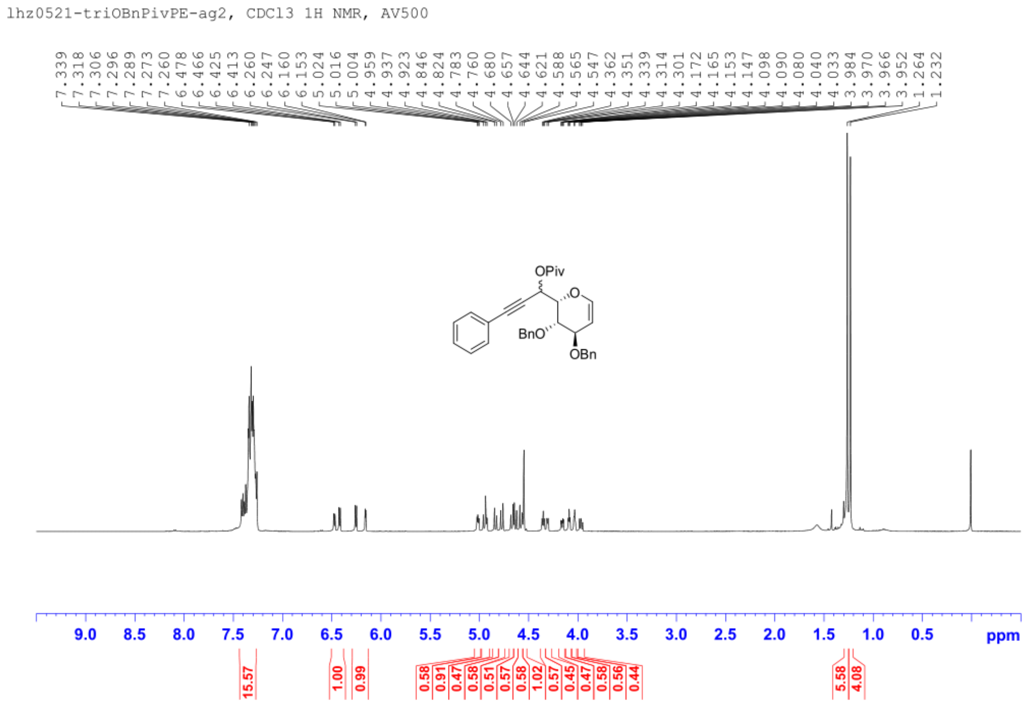

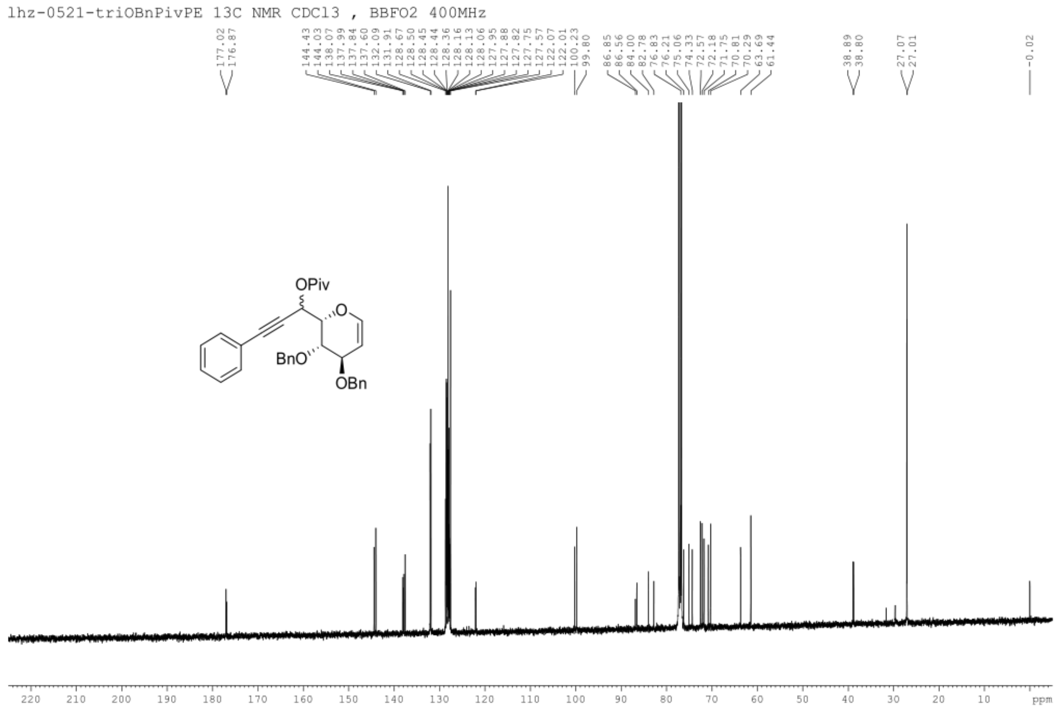

Supplementary Figure 35. ^{1}H and ^{13}C NMR spectrum for 1t.

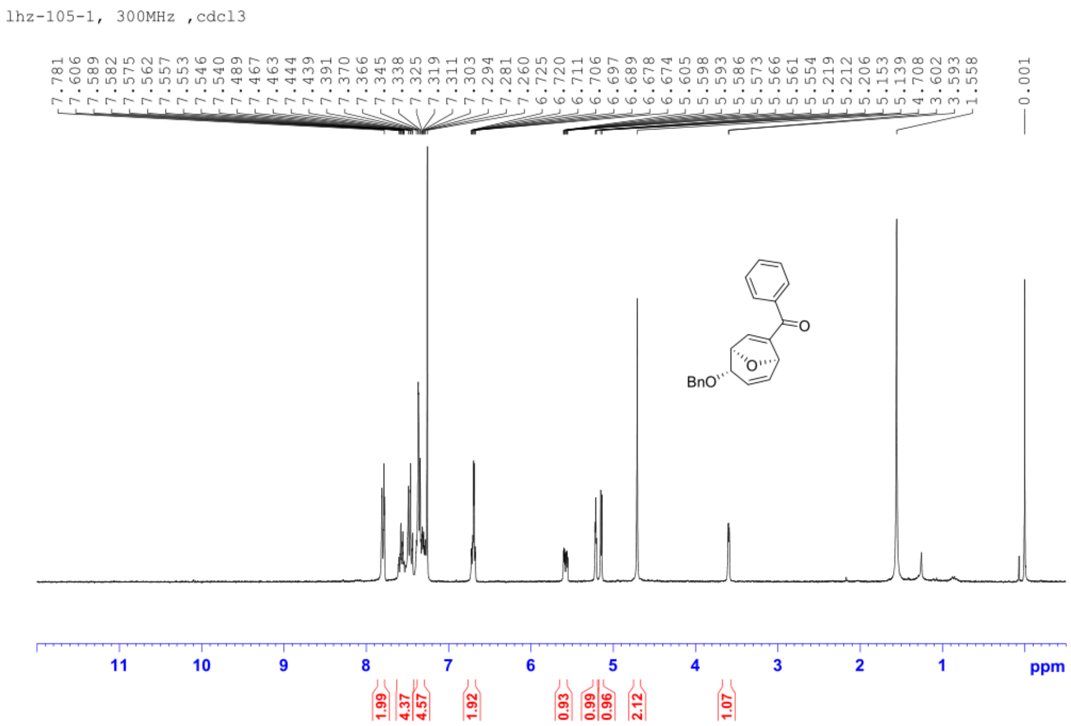

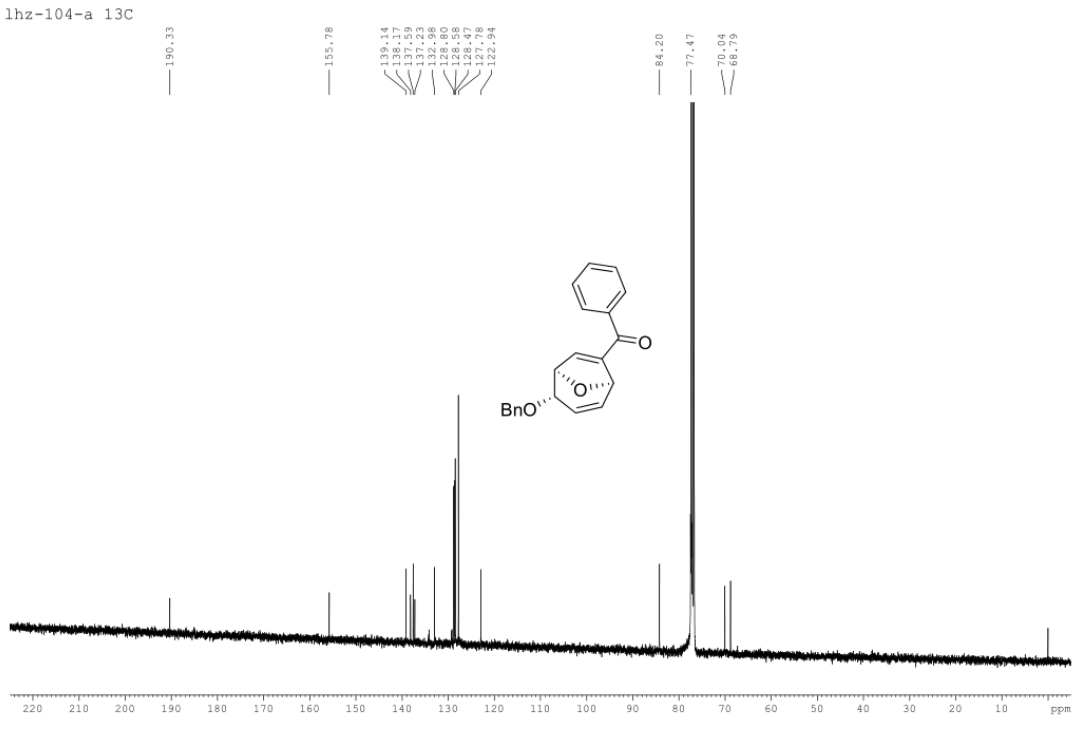

Supplementary Figure 36. ^{1}H and ^{13}C NMR spectrum for 2a.

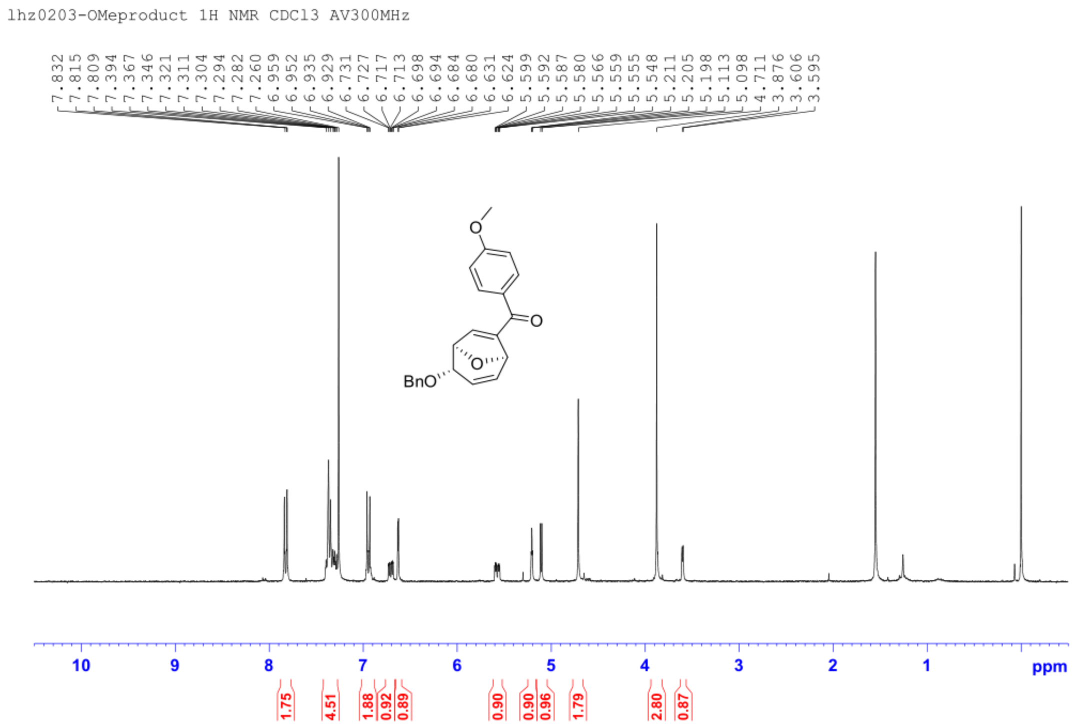

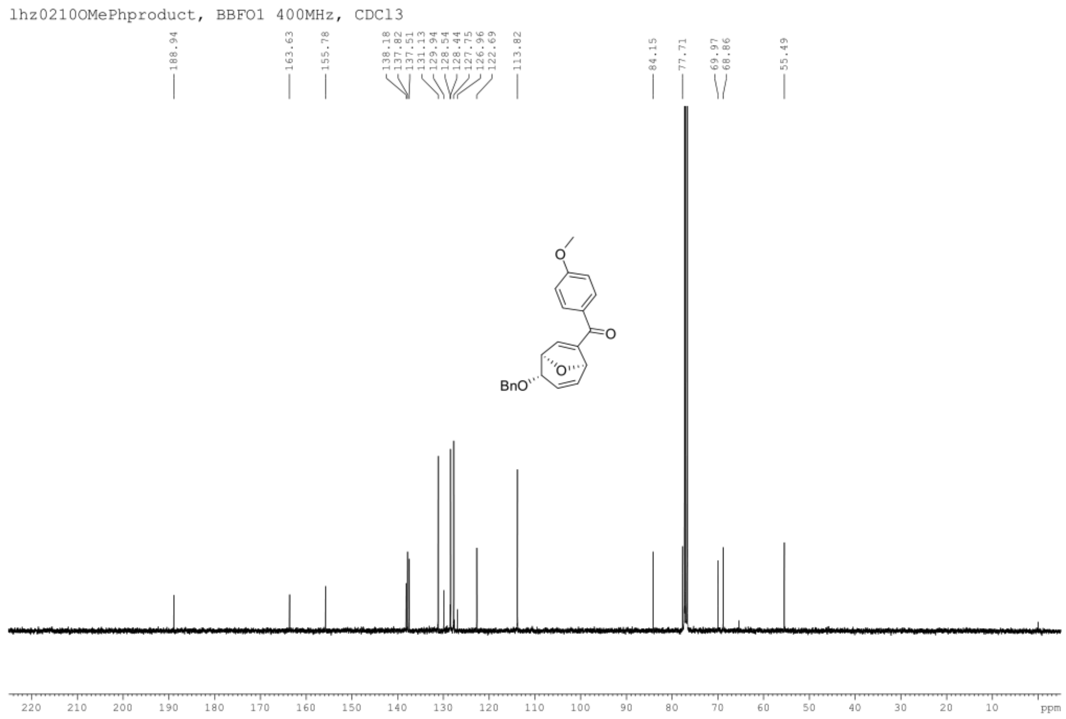

Supplementary Figure 37. ^{1}H and ^{13}C NMR spectrum for 2b.

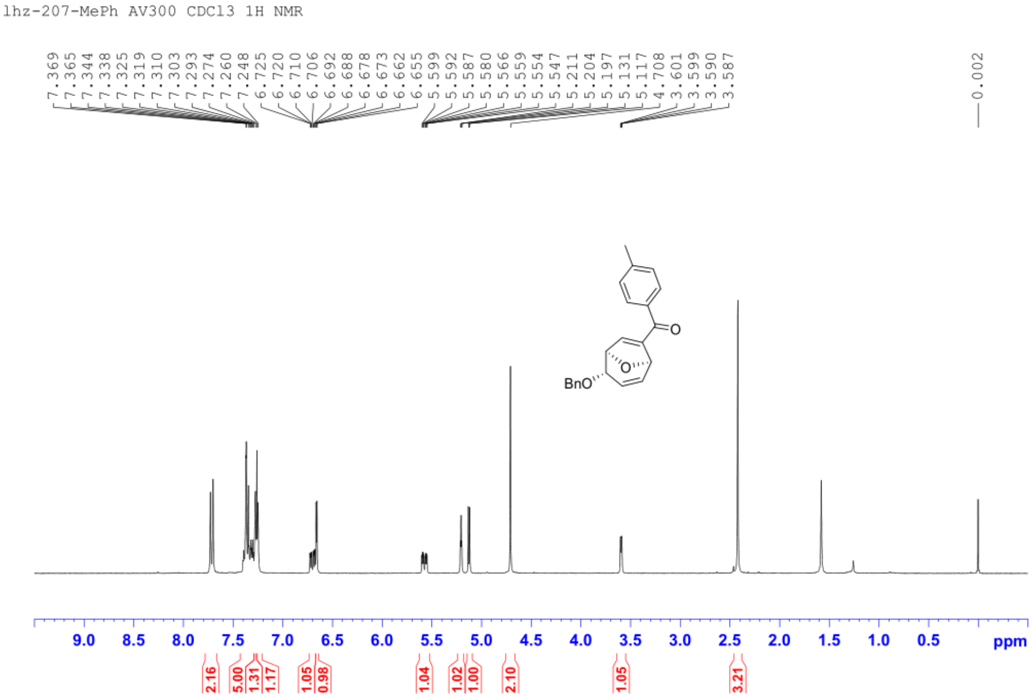

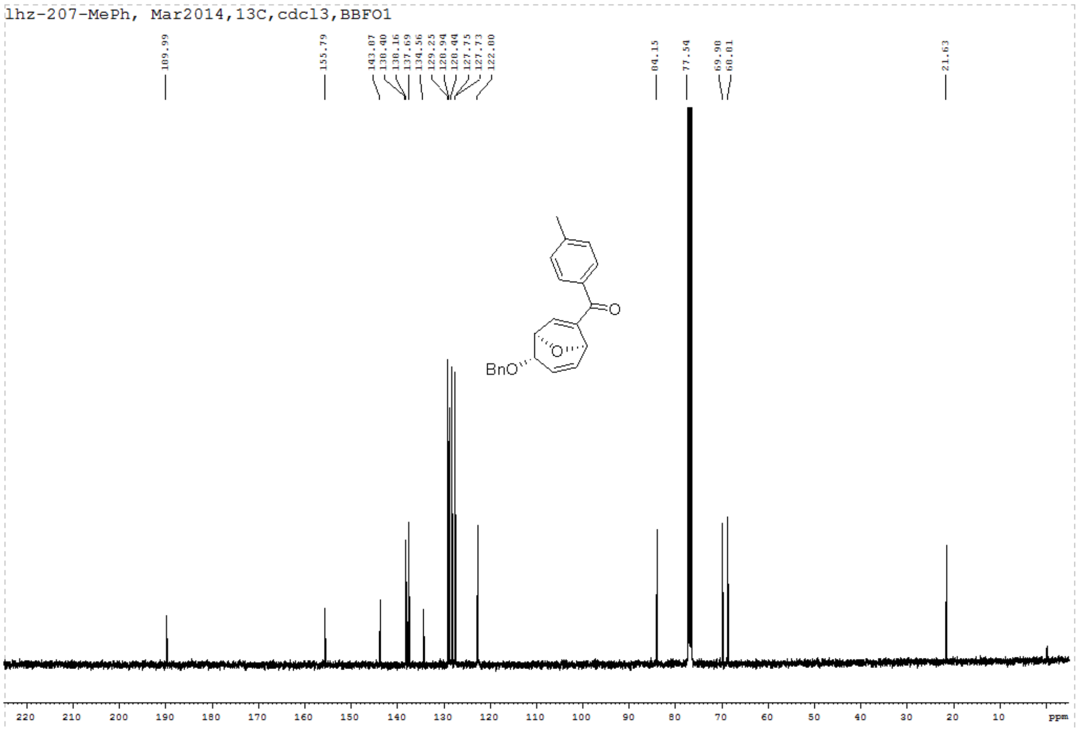

Supplementary Figure 38. **^{1}H and ^{13}C NMR spectrum for 2c.**

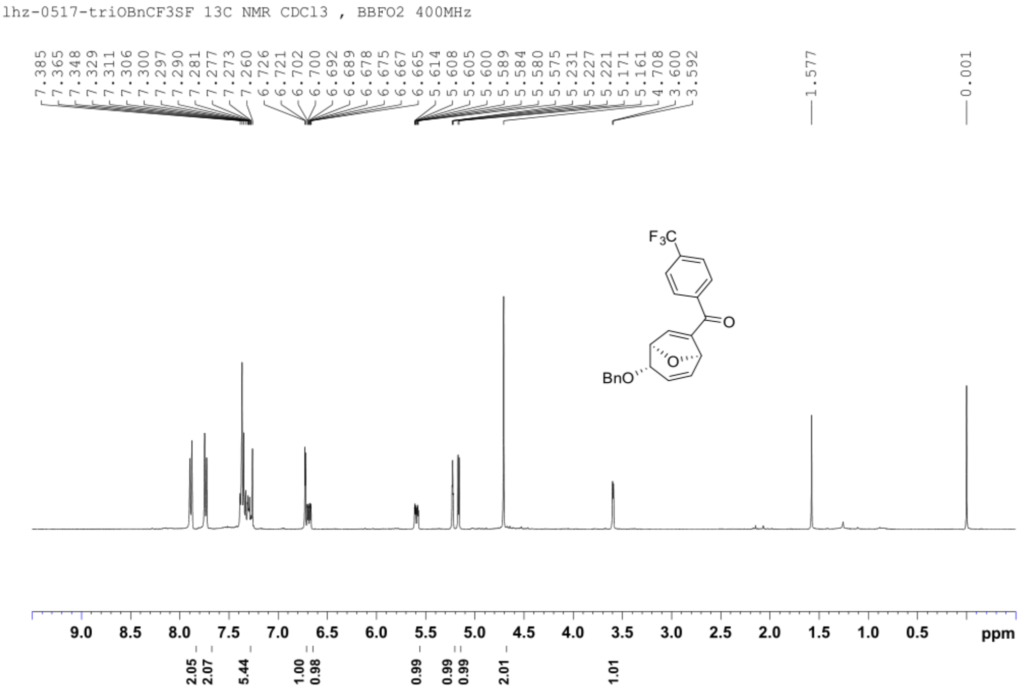

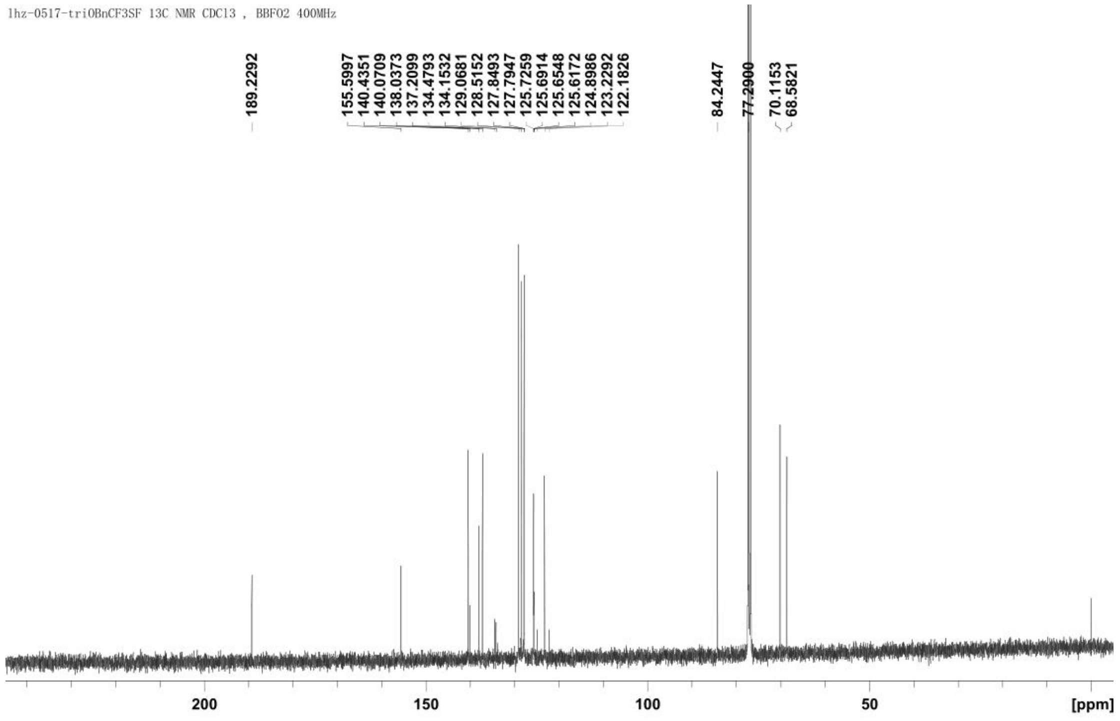

Supplementary Figure 39. ^{1}**H and** ^{13}**C NMR spectrum for 2d.**

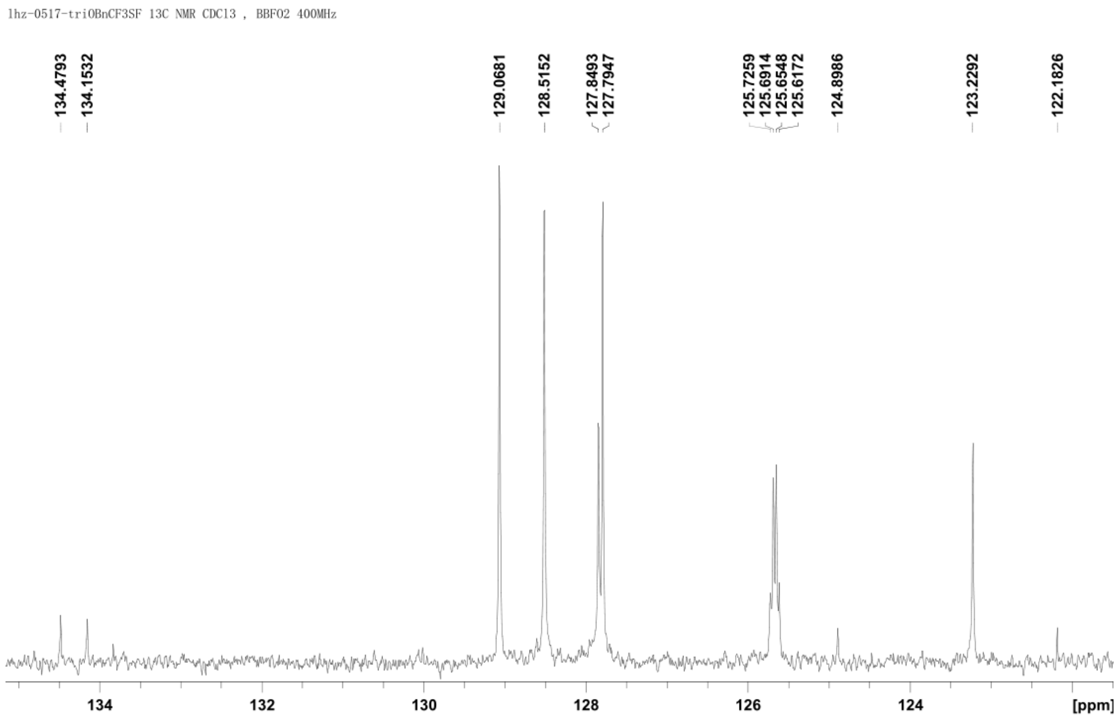

Supplementary Figure 40. ^{19}F NMR spectrum for 2d.

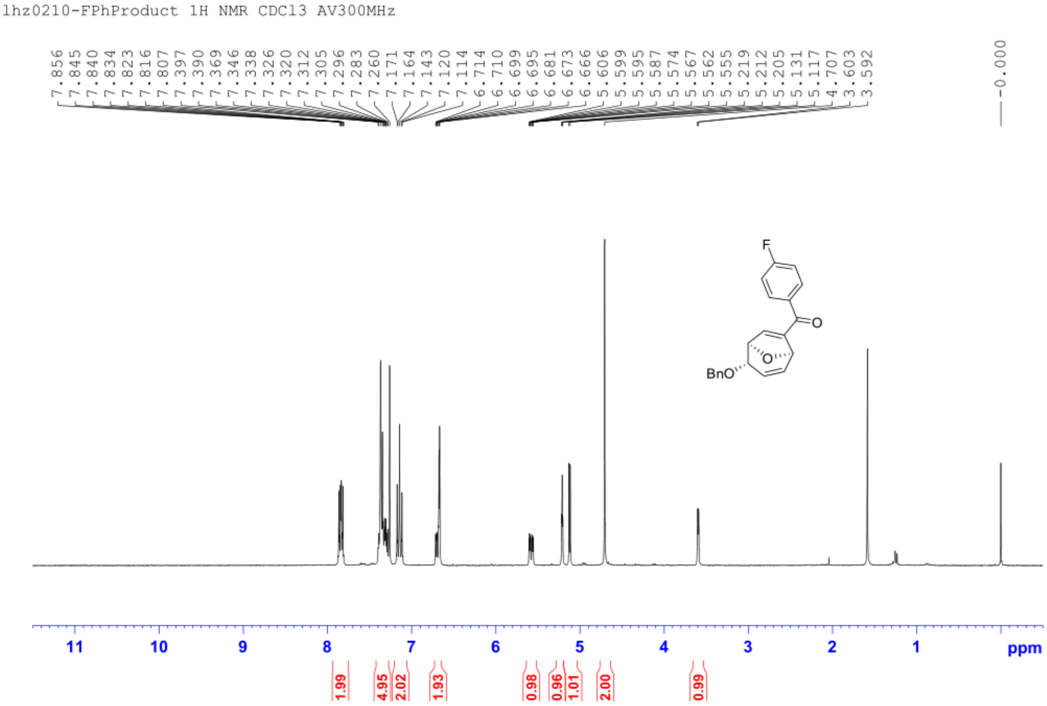

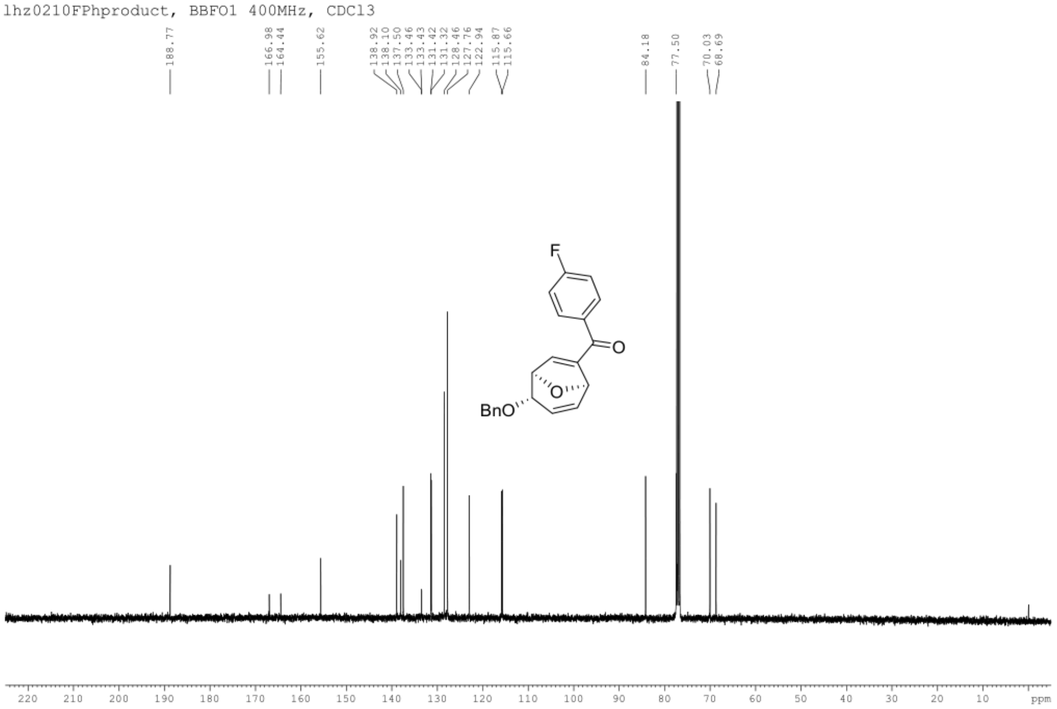

Supplementary Figure 41. ^{1}H and ^{13}C NMR spectrum for 2e.

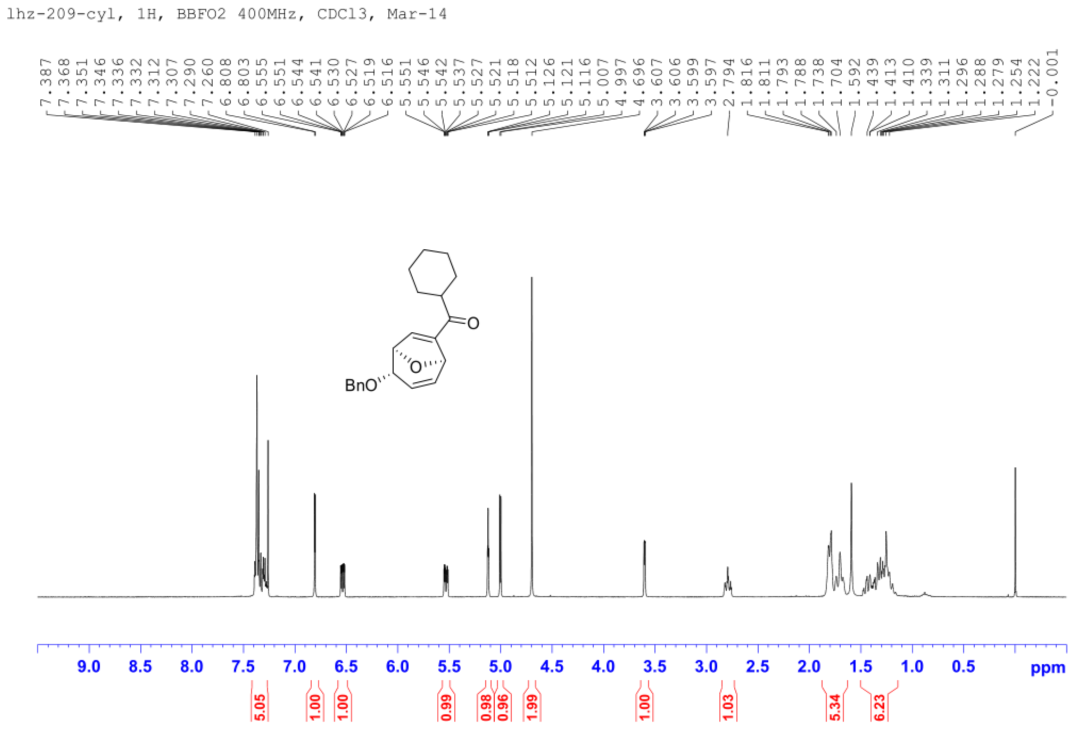

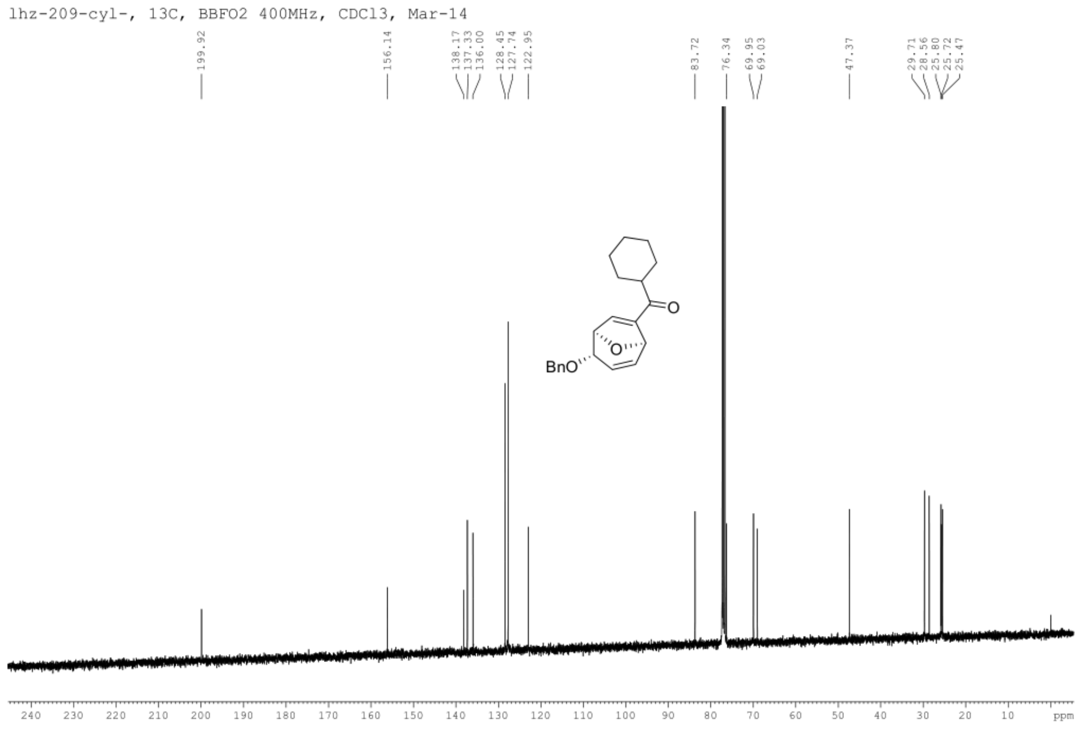

Supplementary Figure 42. ^{1}**H and** ^{13}**C NMR spectrum for 2f.**

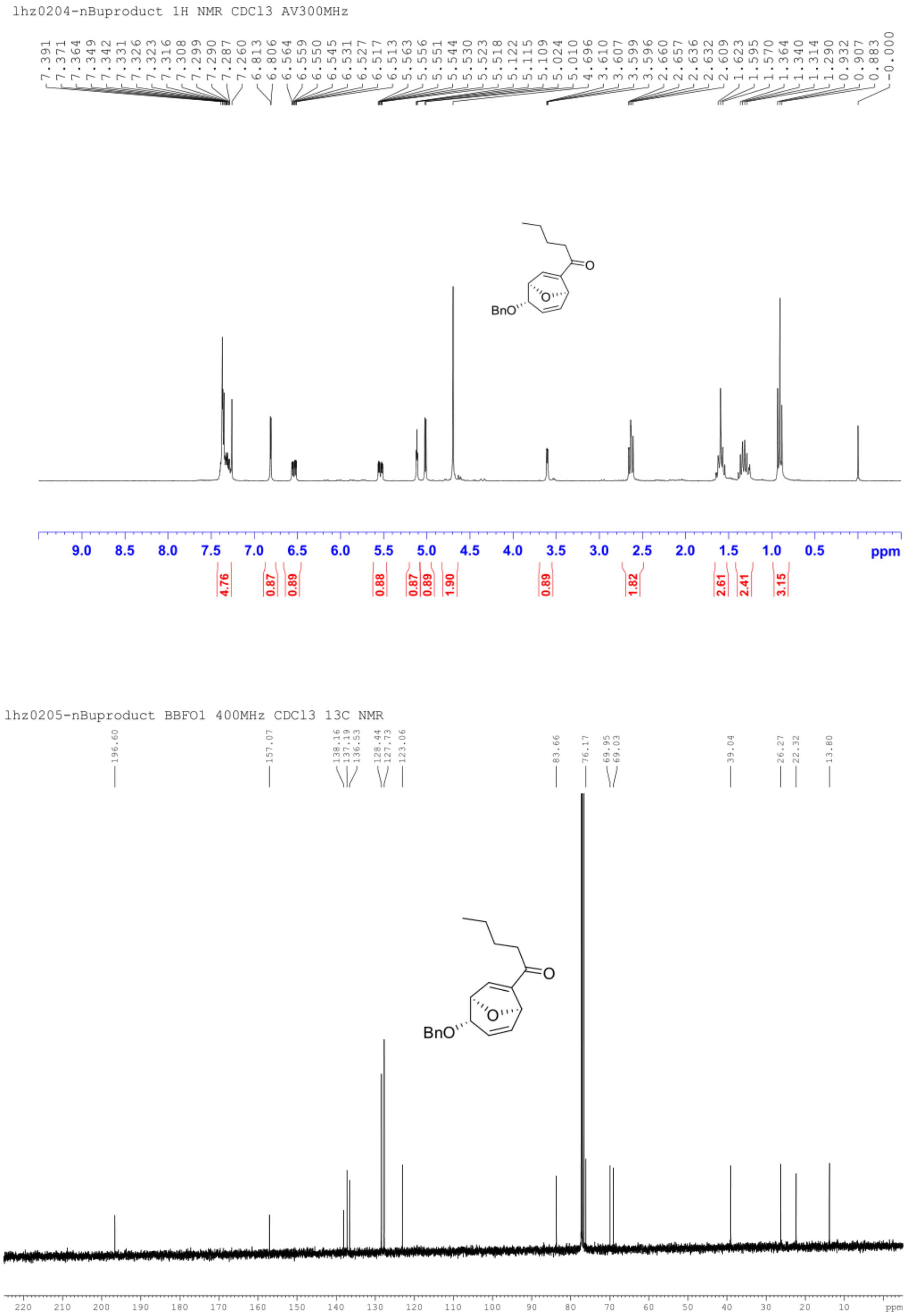

Supplementary Figure 43. ^{1}H and ^{13}C NMR spectrum for 2g.

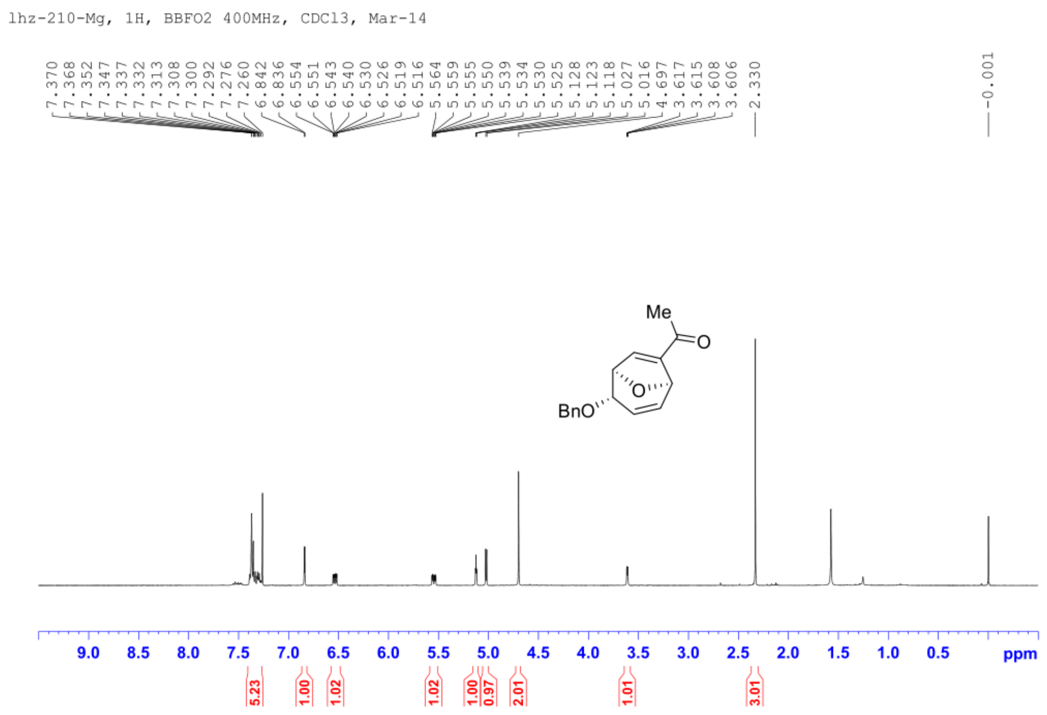

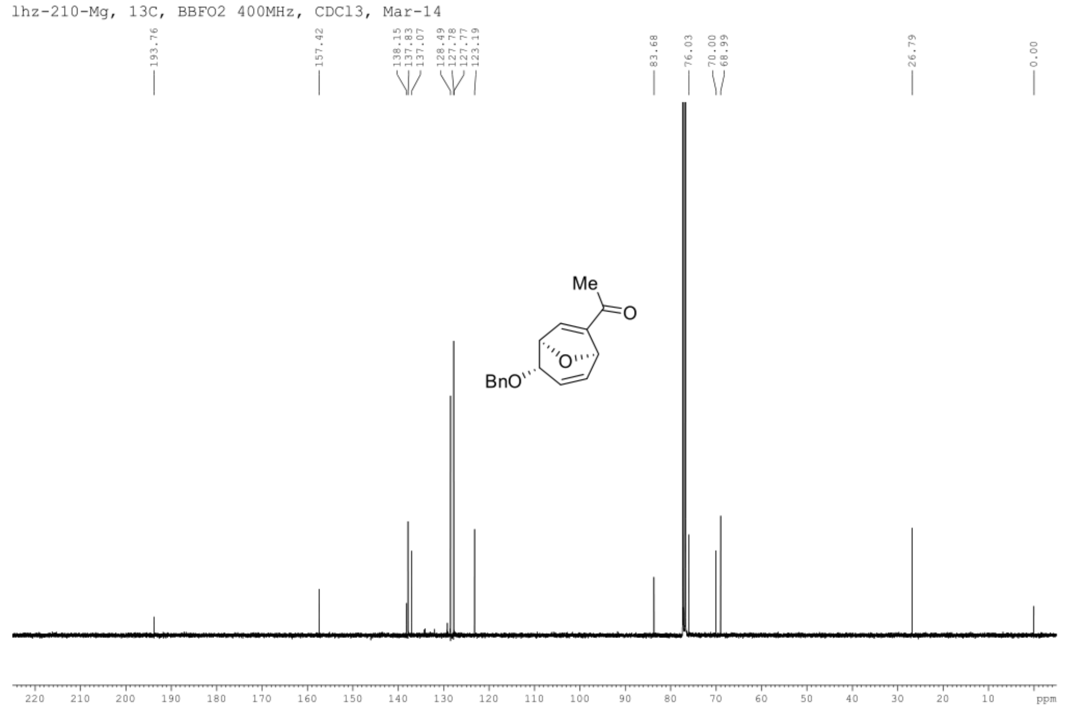

Supplementary Figure 44. ^{1}H and ^{13}C NMR spectrum for 2h.

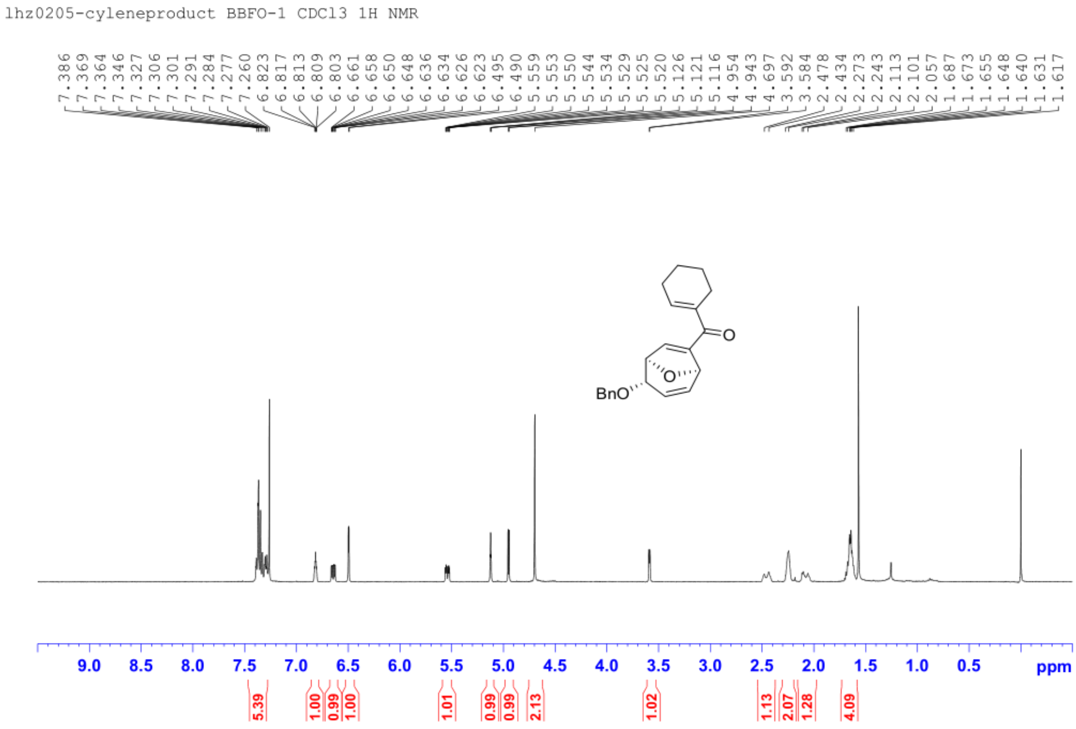

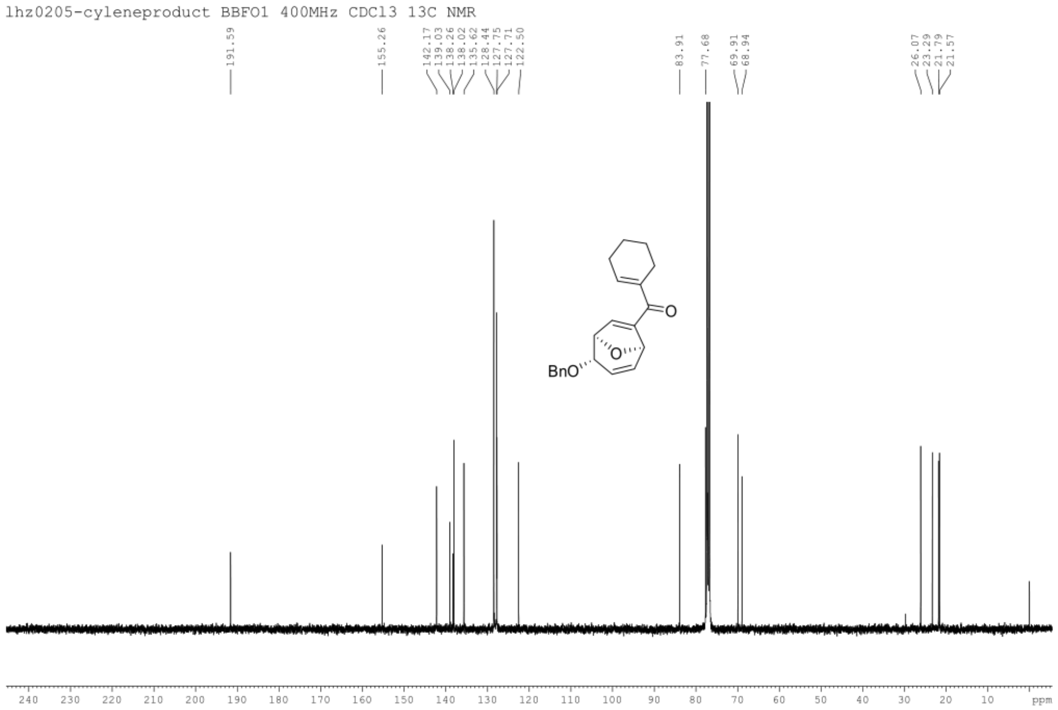

Supplementary Figure 45. ^{1}H and ^{13}C NMR spectrum for 2i.

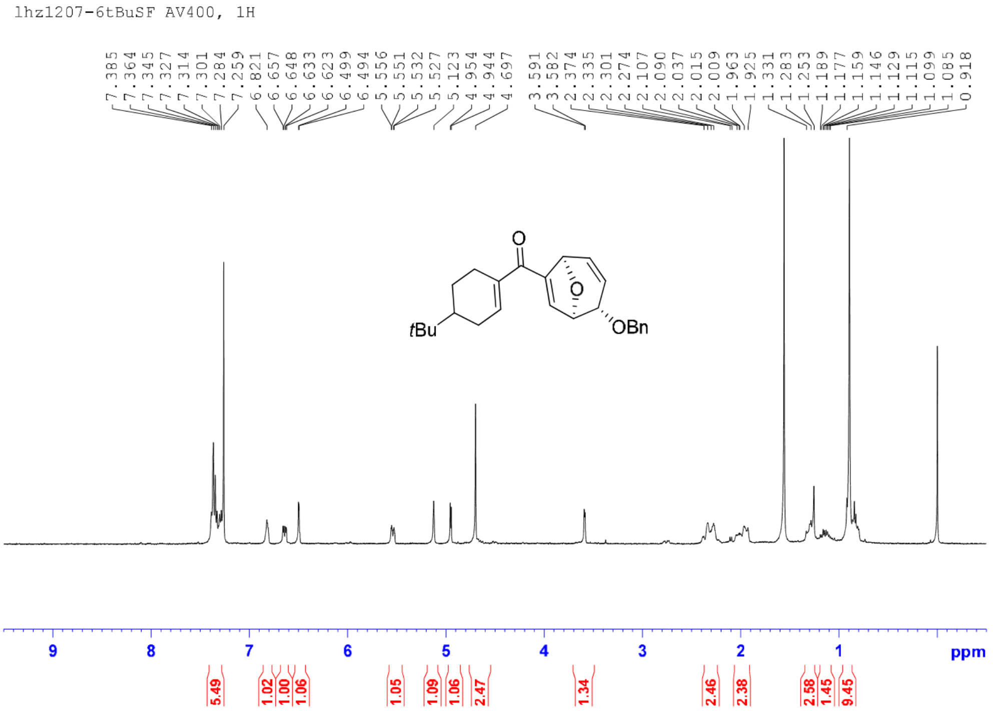

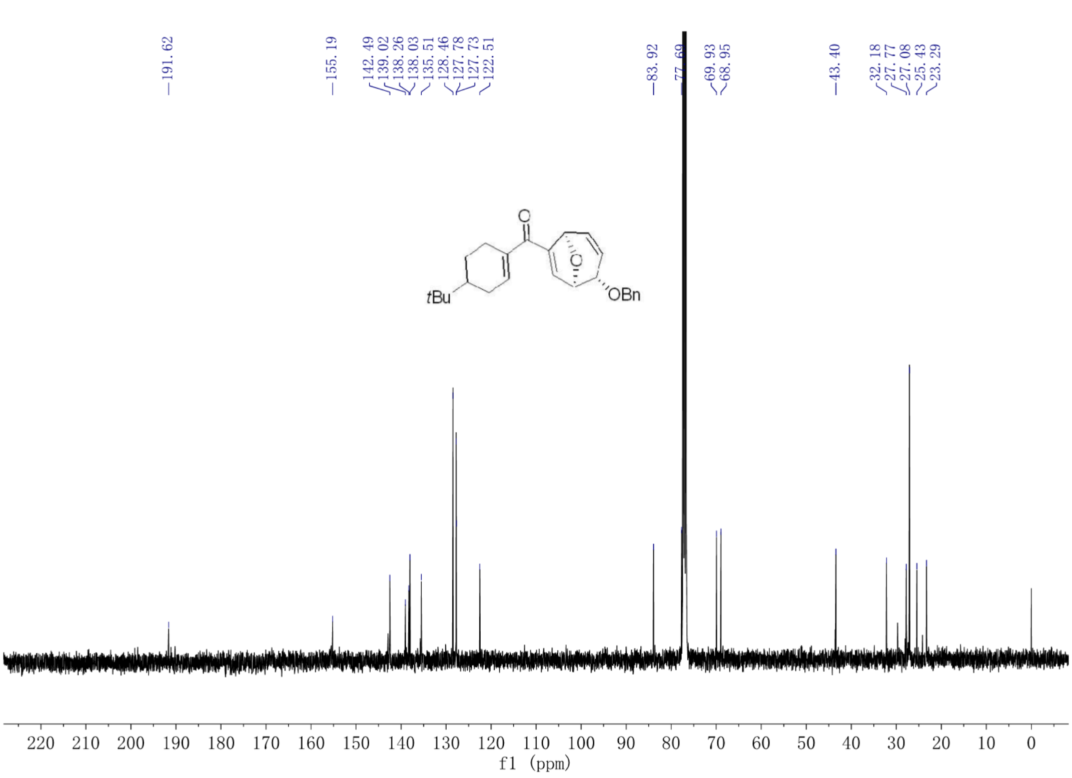

Supplementary Figure 46. ^{1}H and ^{13}C NMR spectrum for 2j.

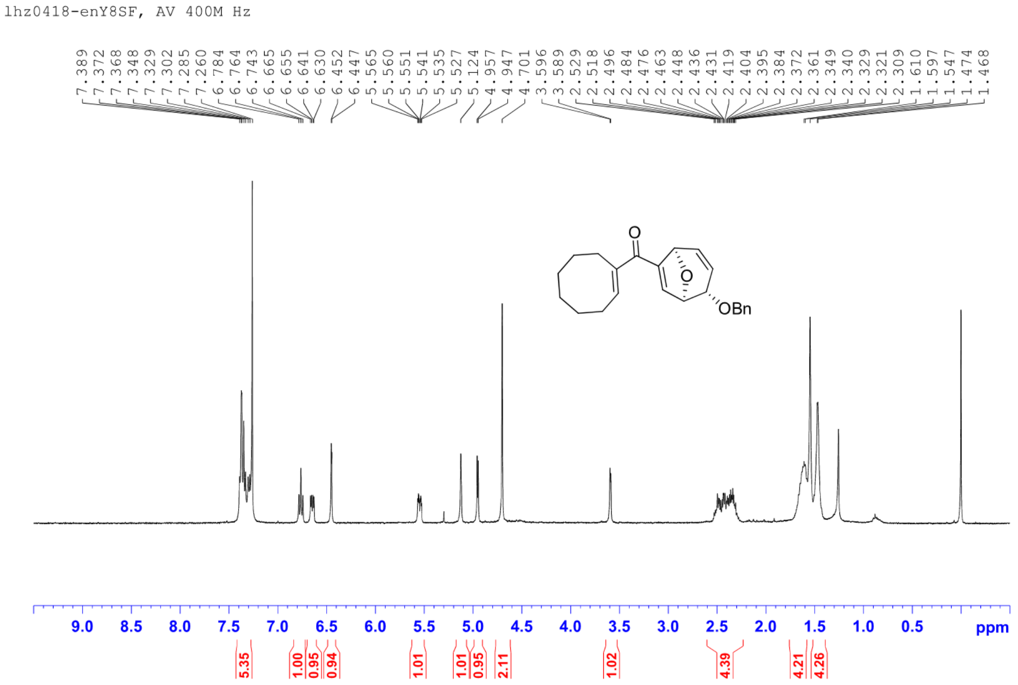

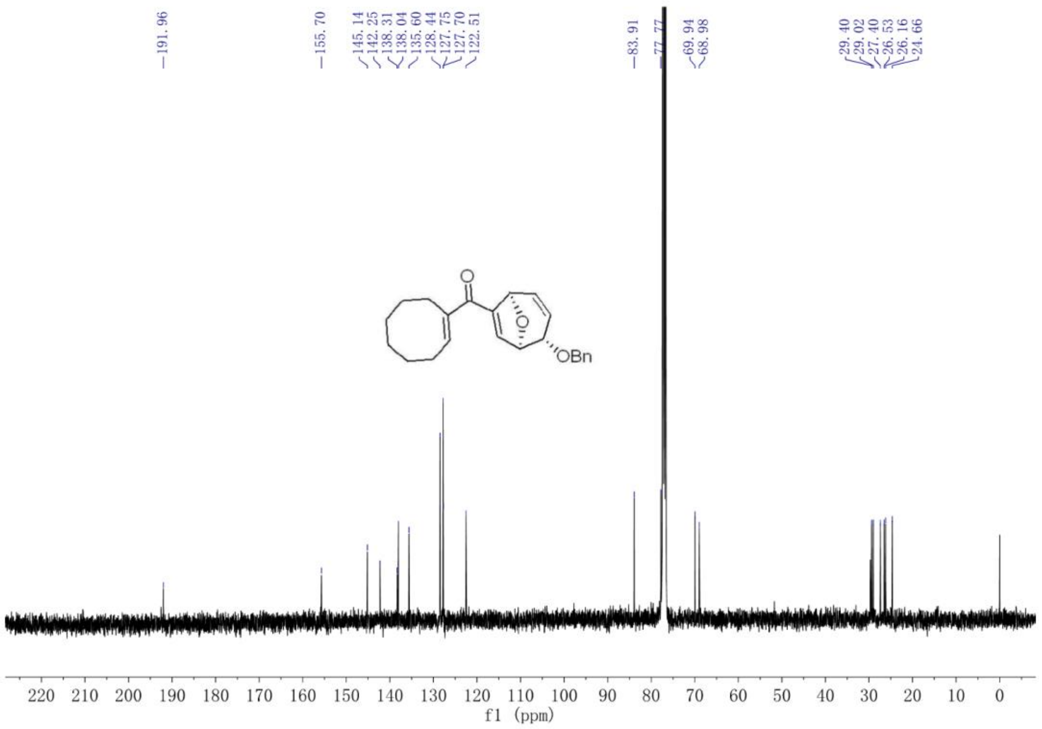

Supplementary Figure 47. ^{1}H and ^{13}C NMR spectrum for 2k.

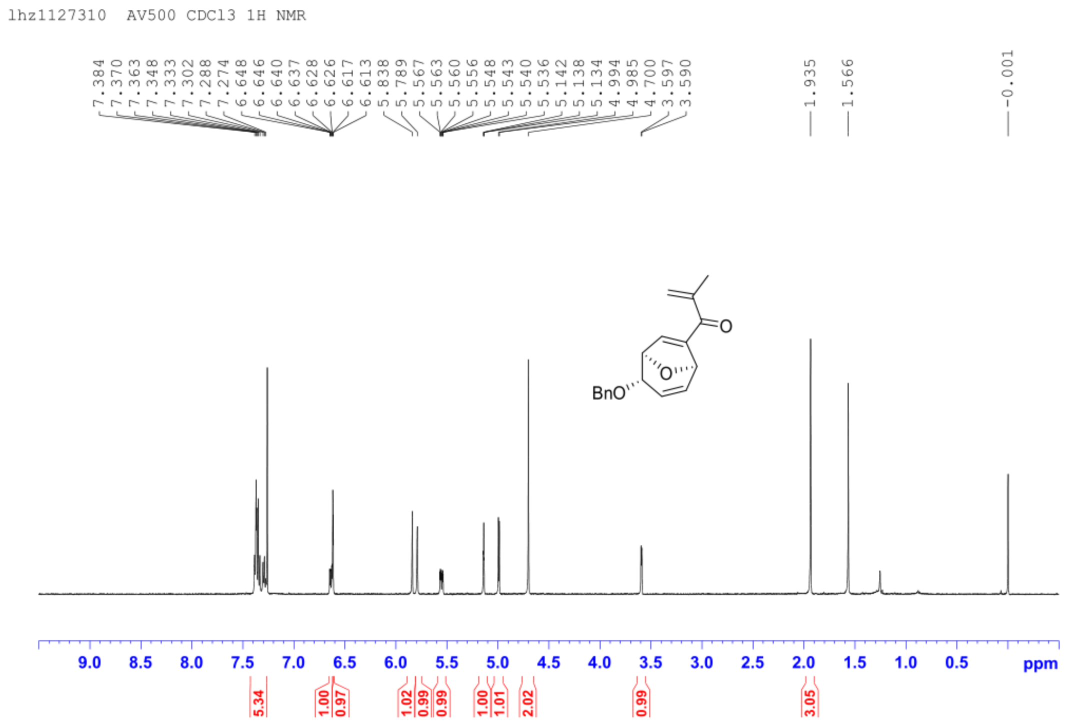

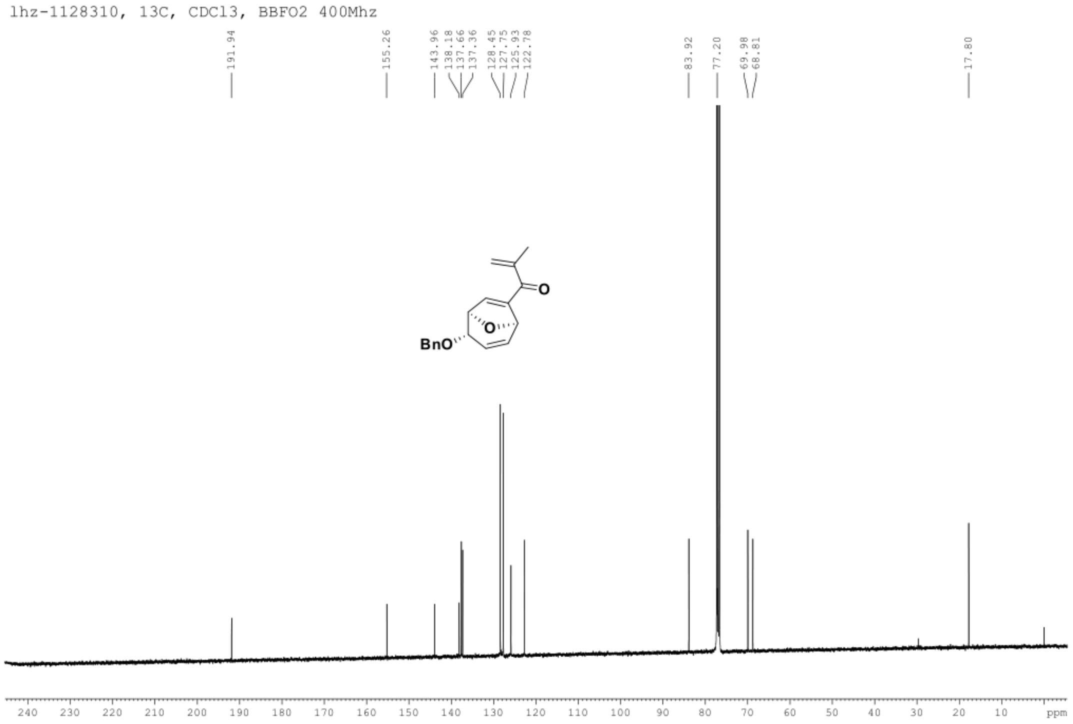

Supplementary Figure 48. ^{1}H and ^{13}C NMR spectrum for 2l.

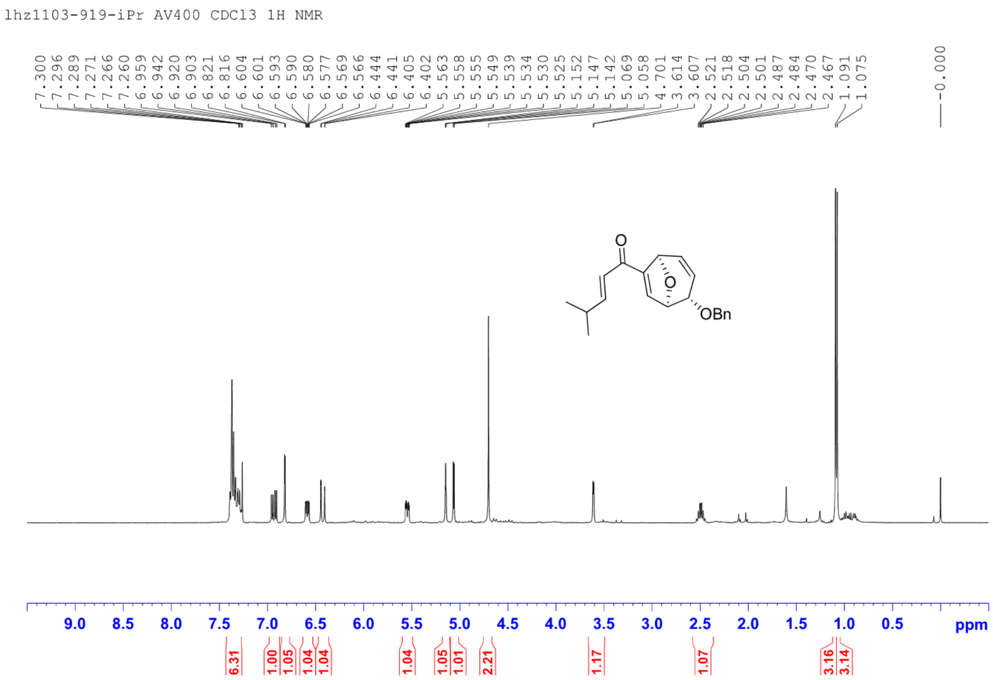

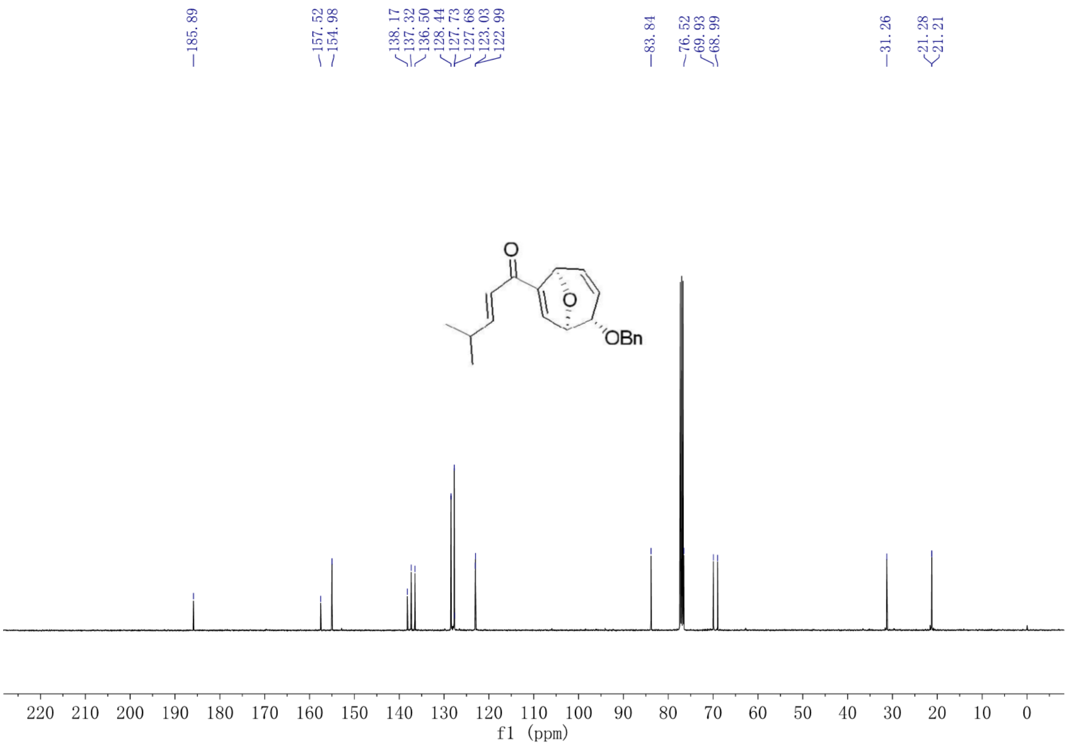

Supplementary Figure 49. ^{1}H and ^{13}C NMR spectrum for 2m.

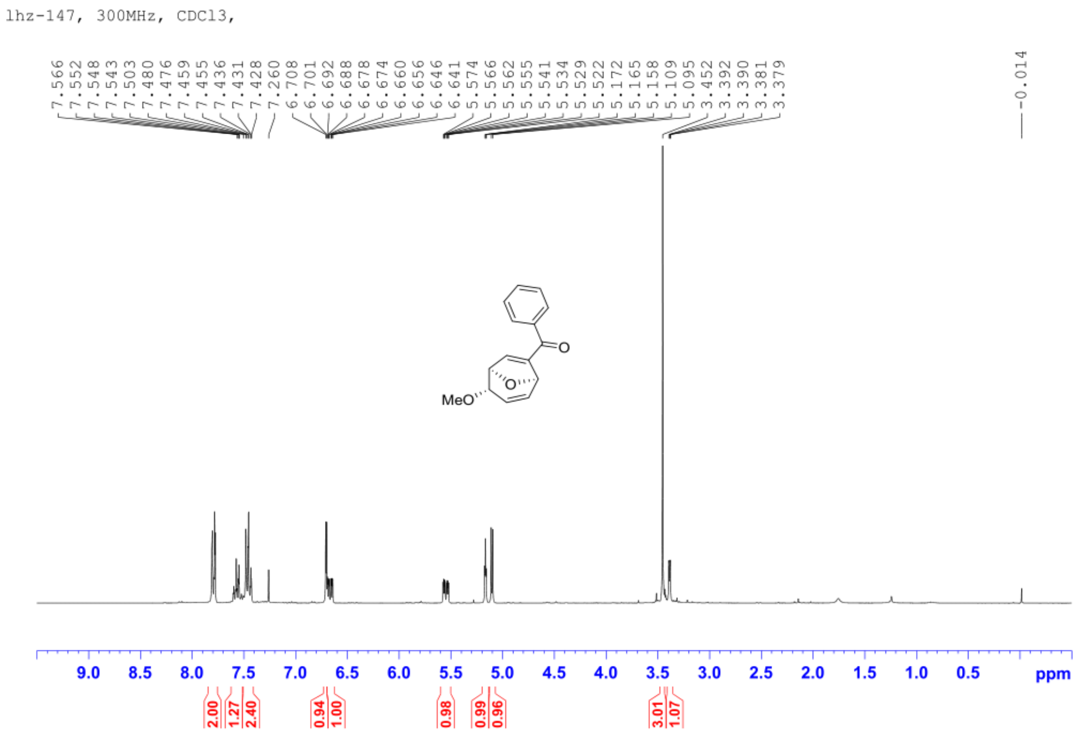

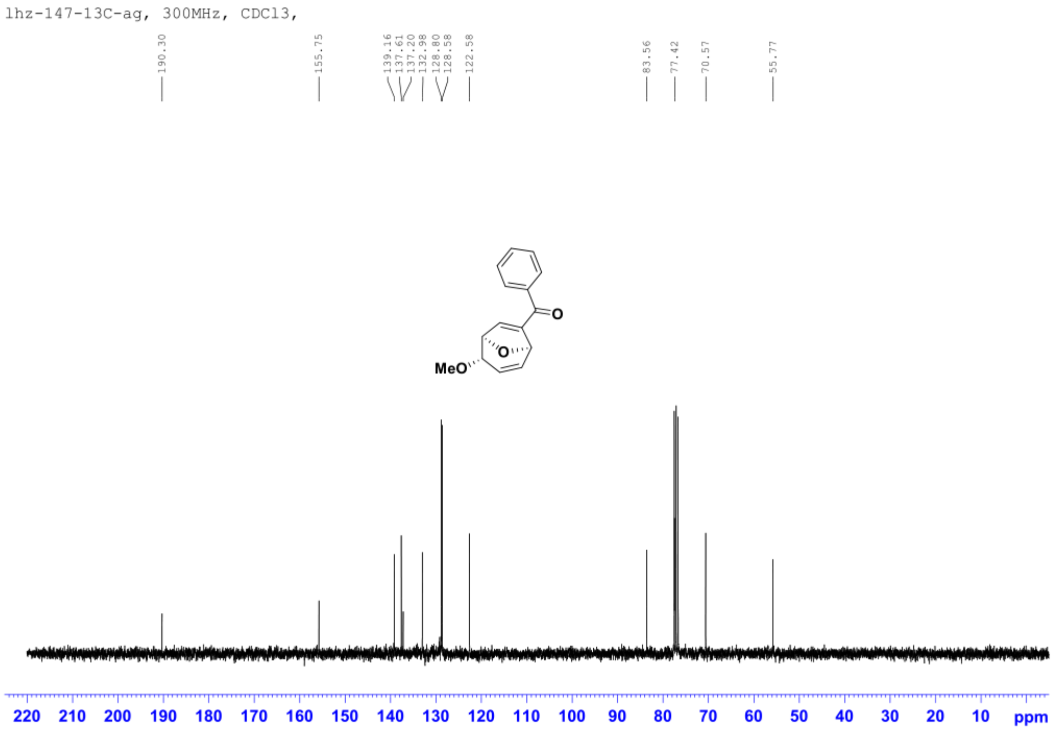

Supplementary Figure 50. ^{1}H and ^{13}C NMR spectrum for 2n.

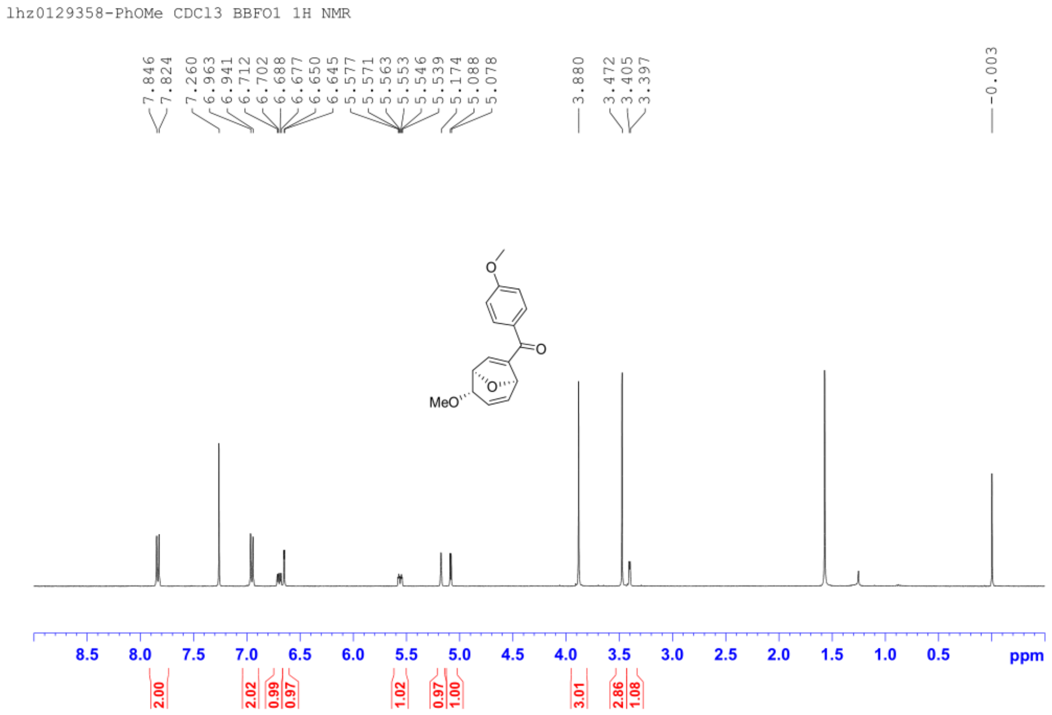

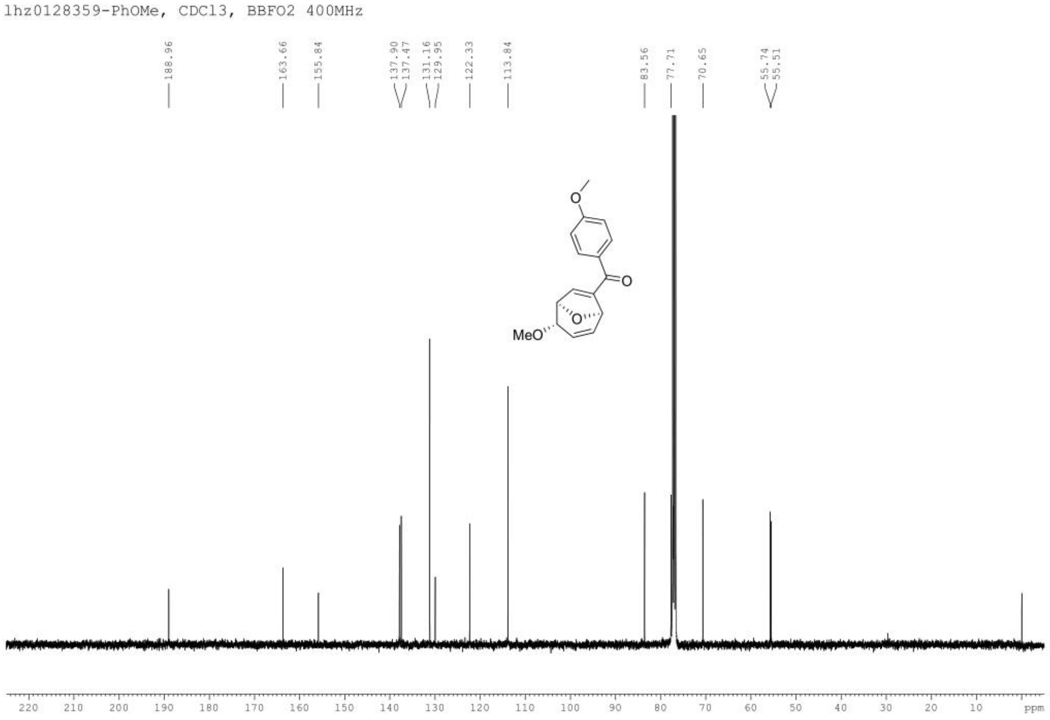

Supplementary Figure 51. ^{1}H and ^{13}C NMR spectrum for 2o.

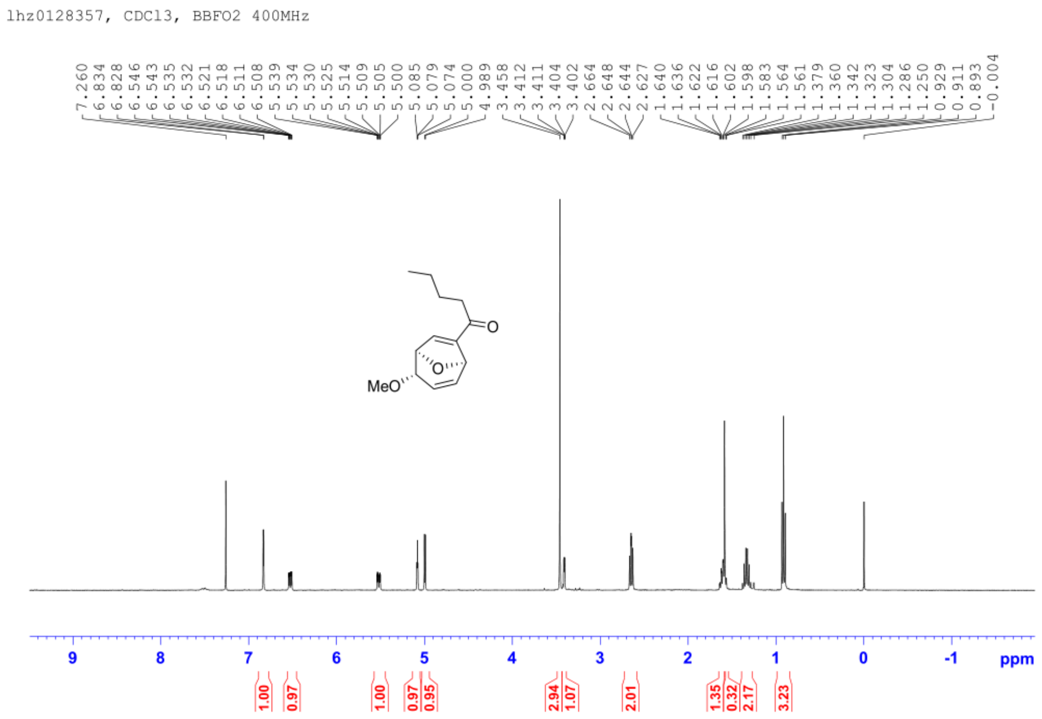

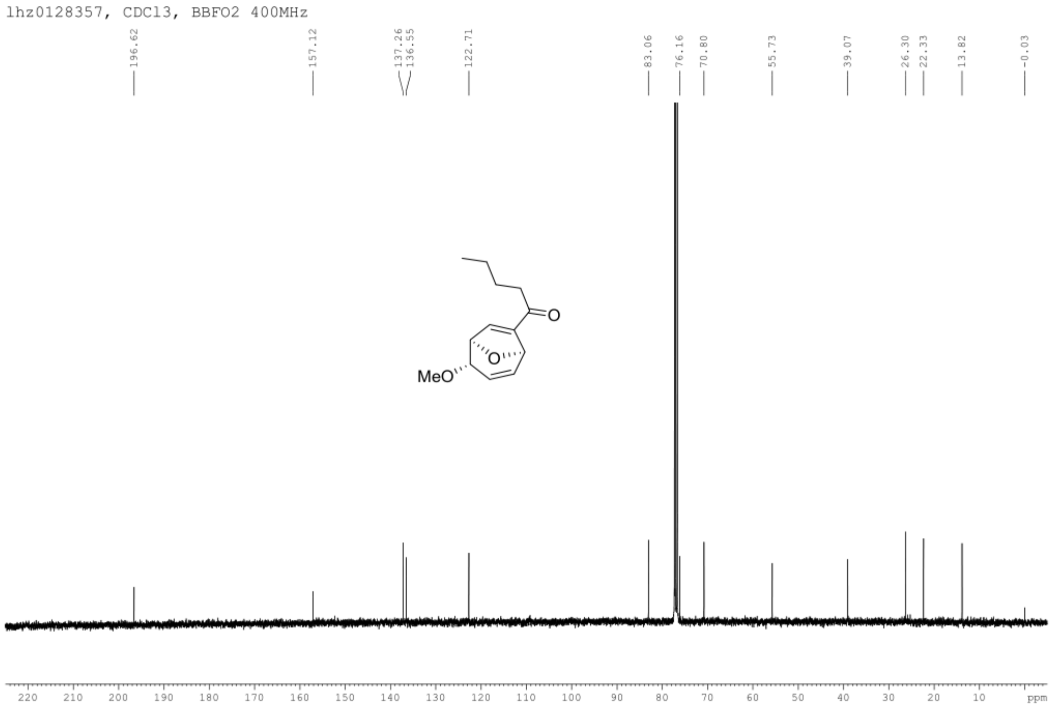

Supplementary Figure 52. ^{1}H and ^{13}C NMR spectrum for 2p.

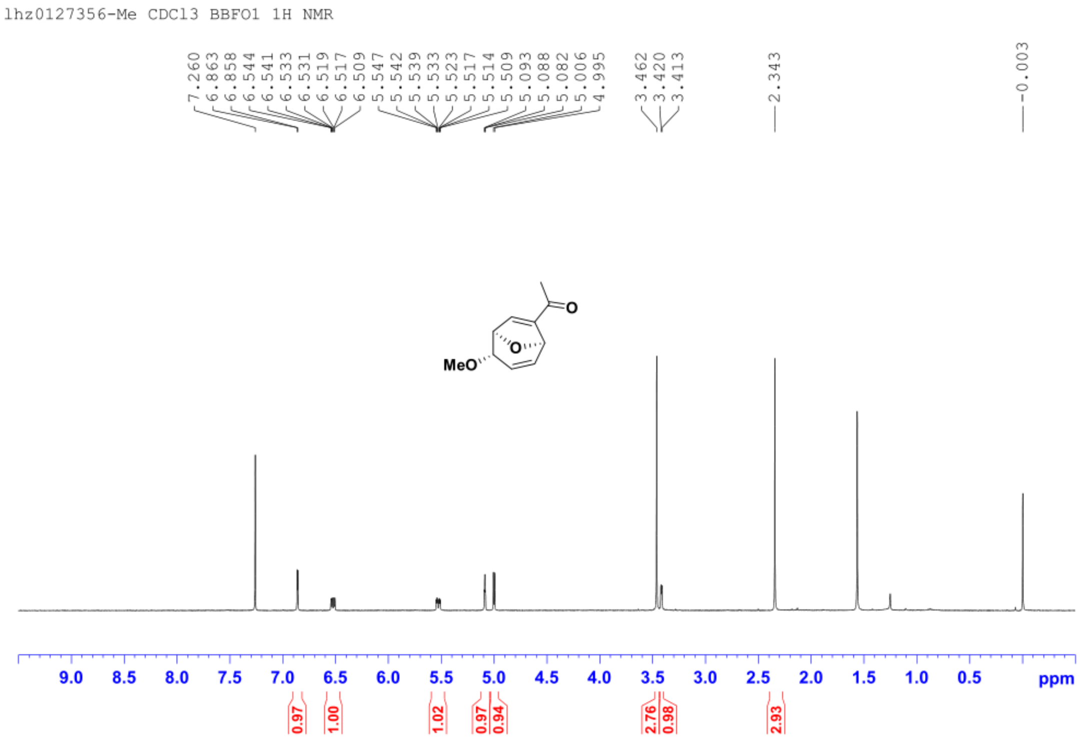

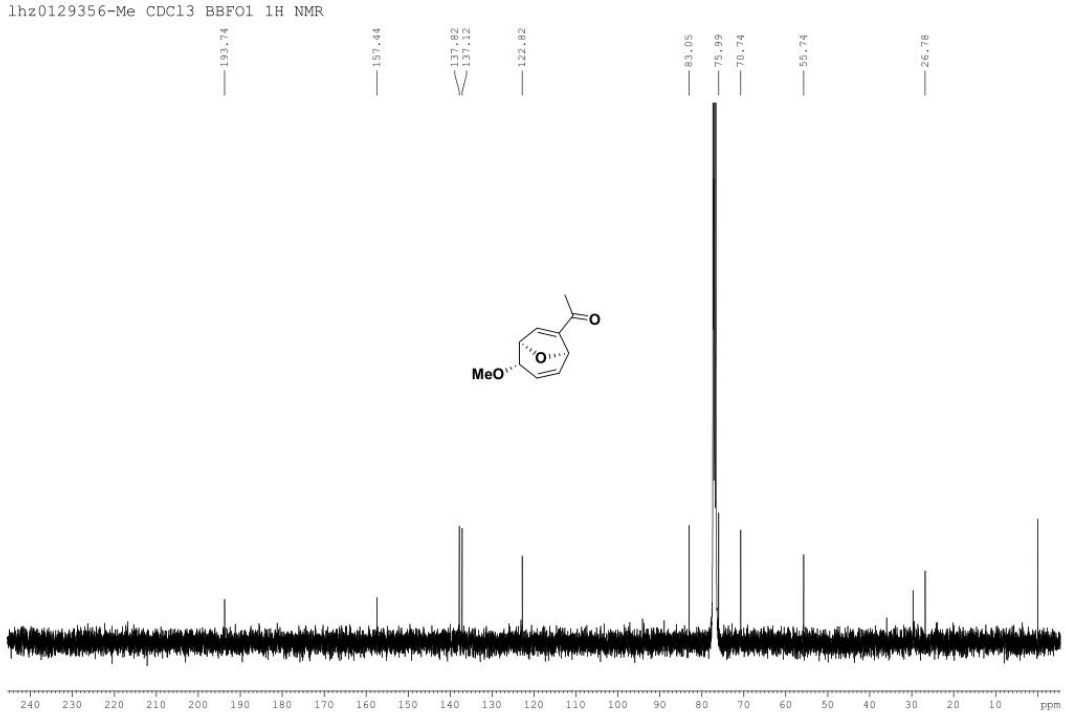

Supplementary Figure 53. ^{1}H and ^{13}C NMR spectrum for 2q.

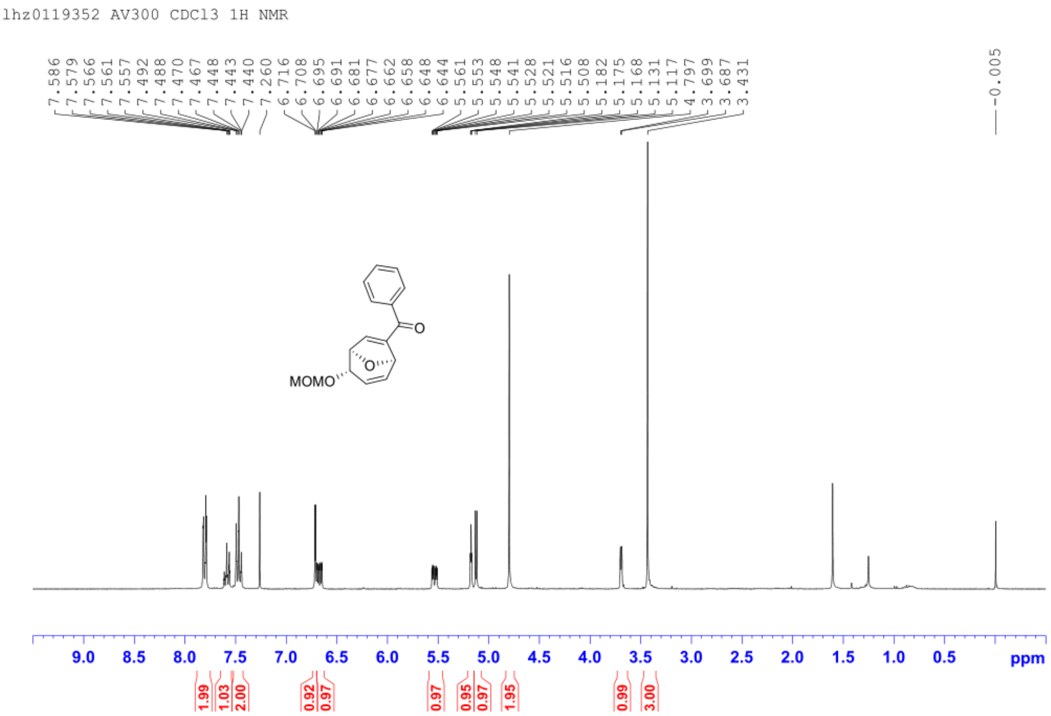

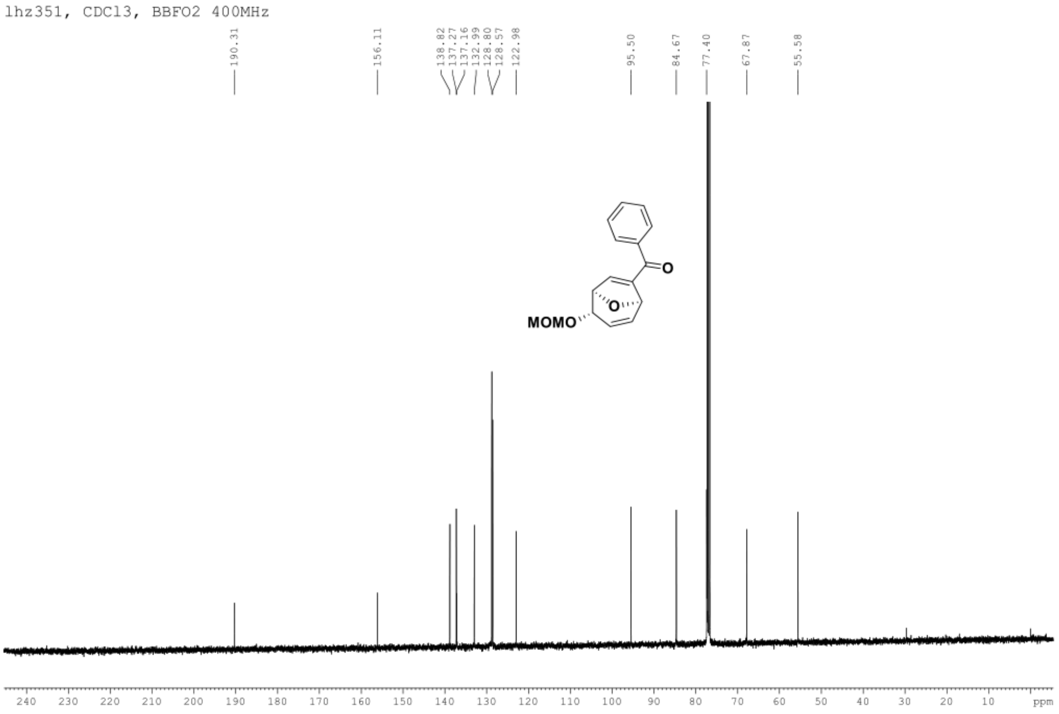

Supplementary Figure 54. ^{1}H and ^{13}C NMR spectrum for 2r.

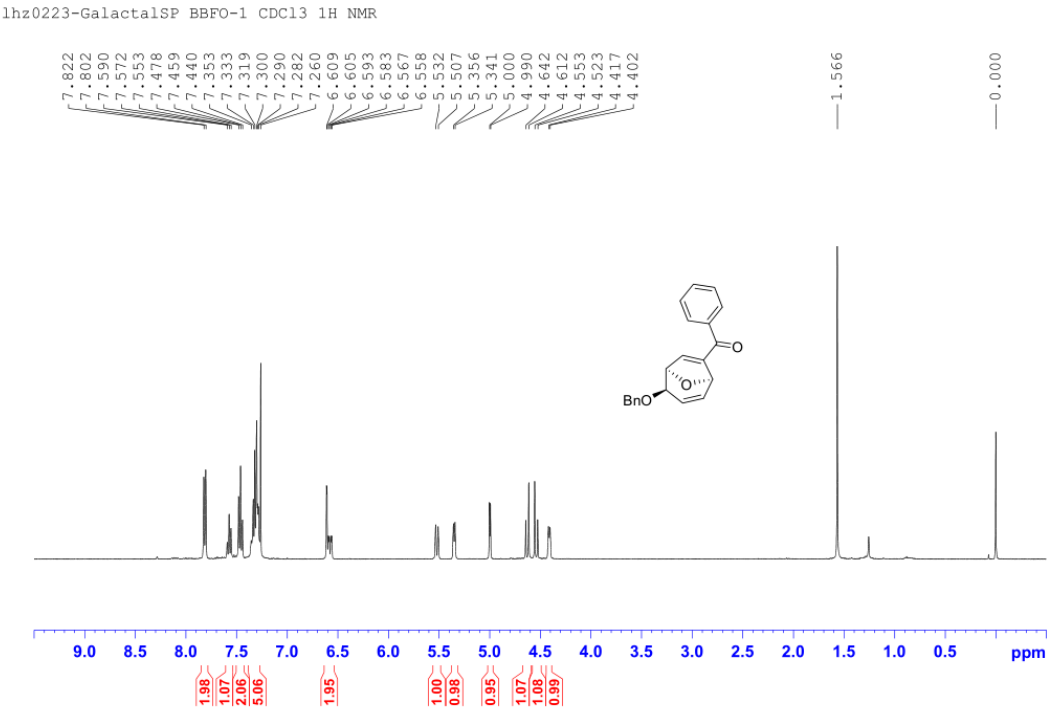

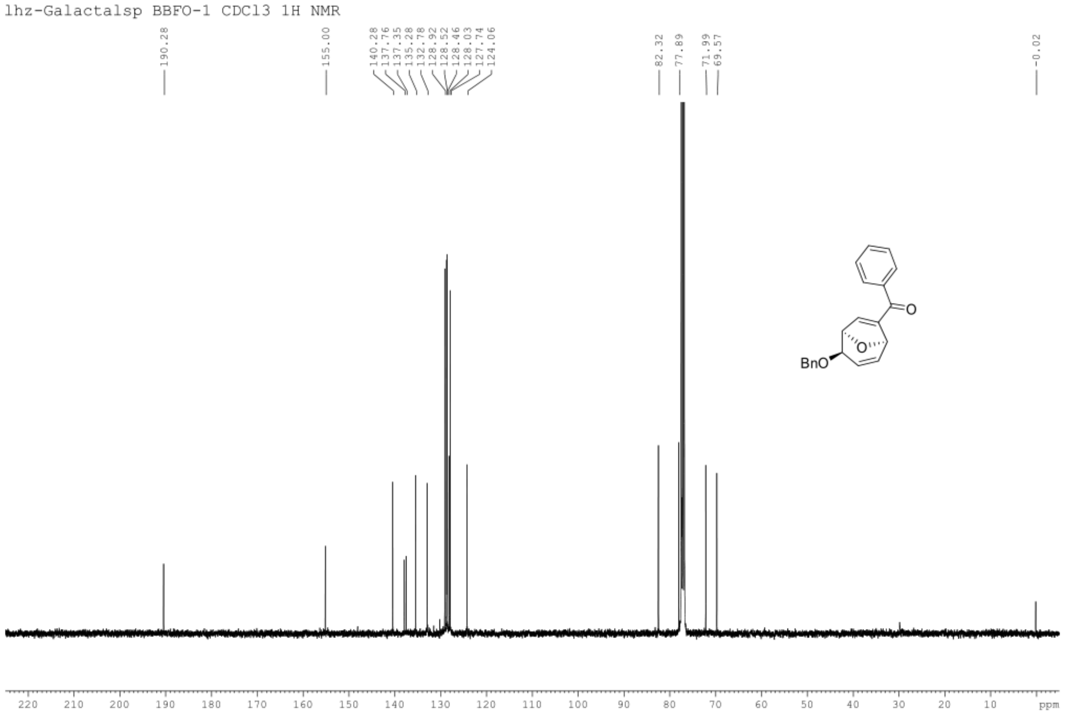

Supplementary Figure 55. ^{1}**H and** ^{13}**C NMR spectrum for** ***epi*****-2a.**

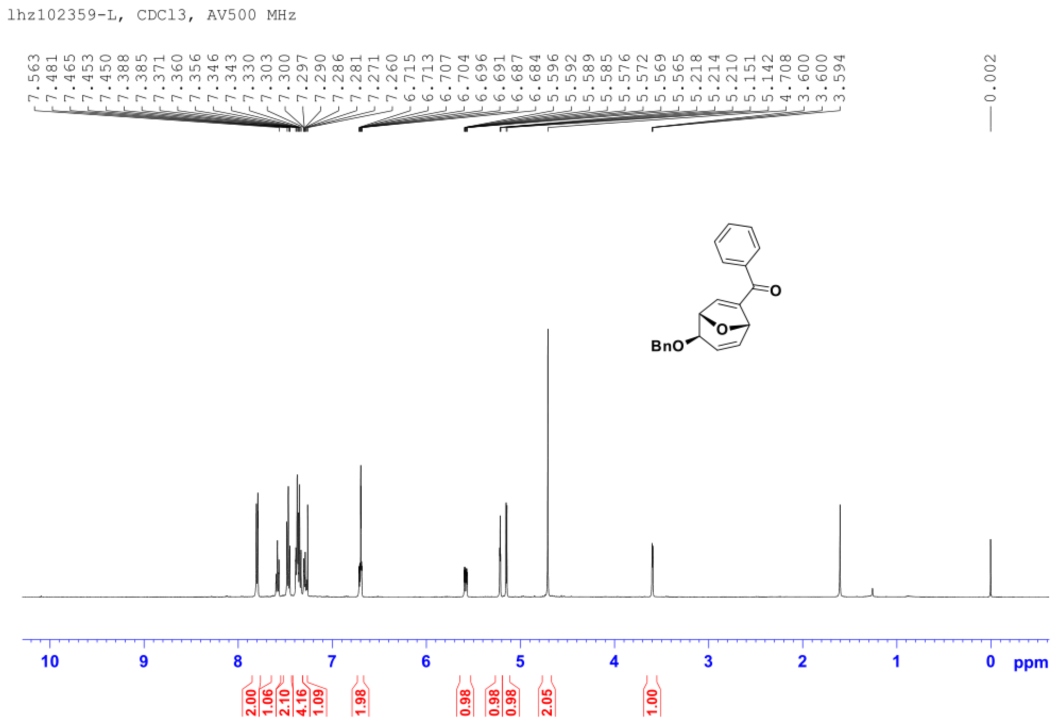

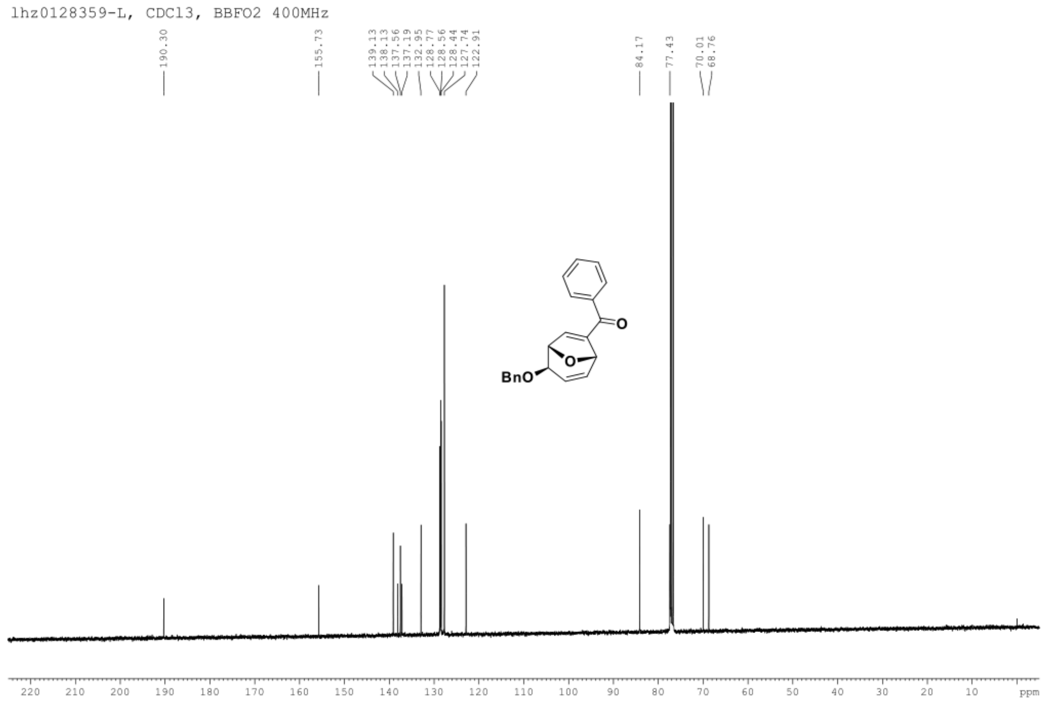

Supplementary Figure 56. ^{1}H and ^{13}C NMR spectrum for *ent*-2a.

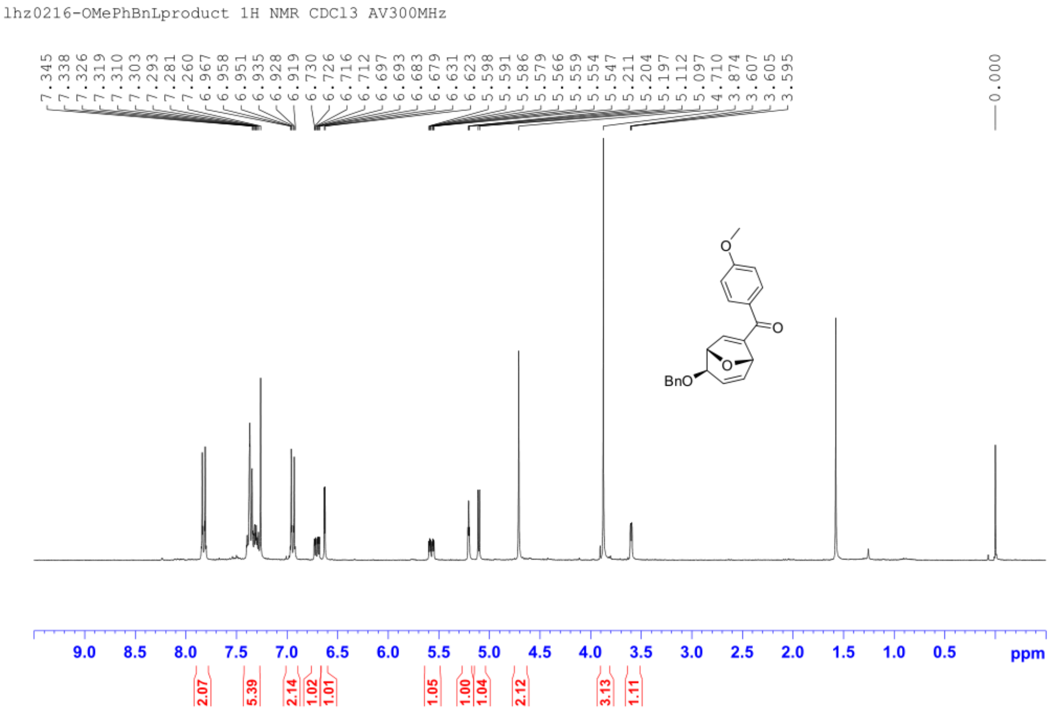

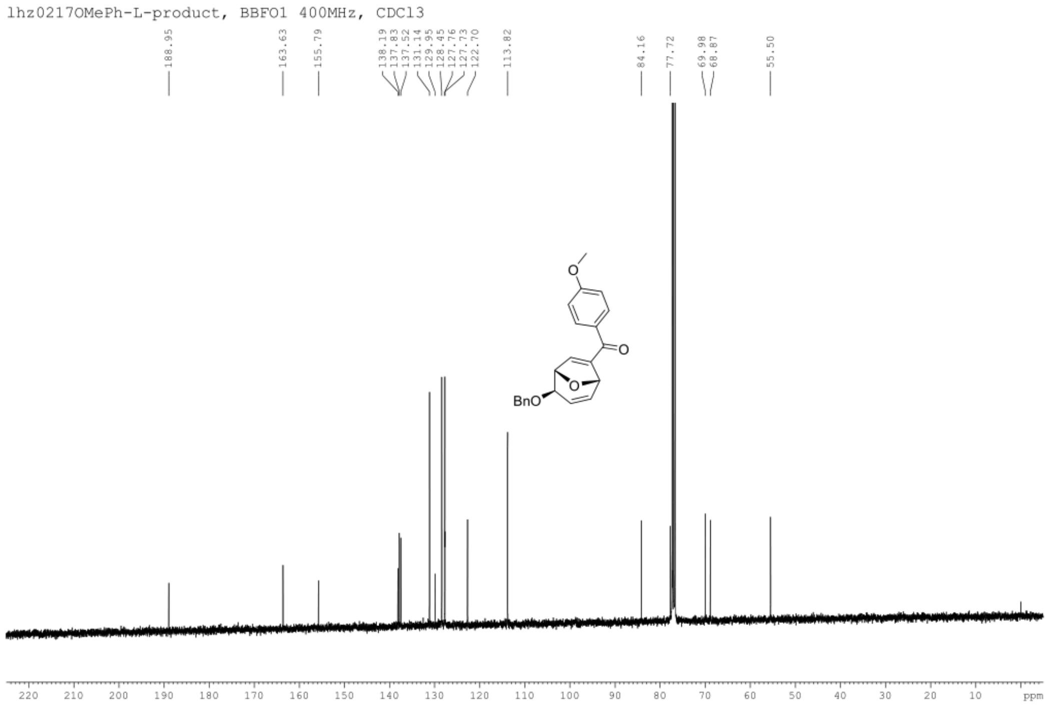

Supplementary Figure 57. ^{1}**H and** ^{13}**C NMR spectrum for *ent*-2b.**

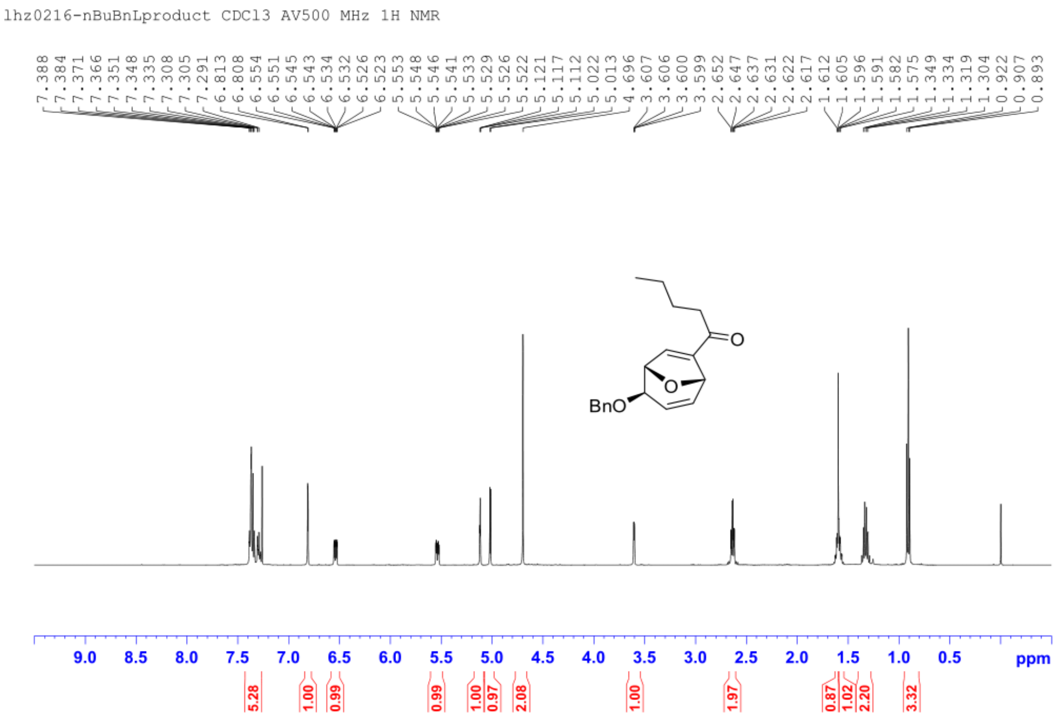

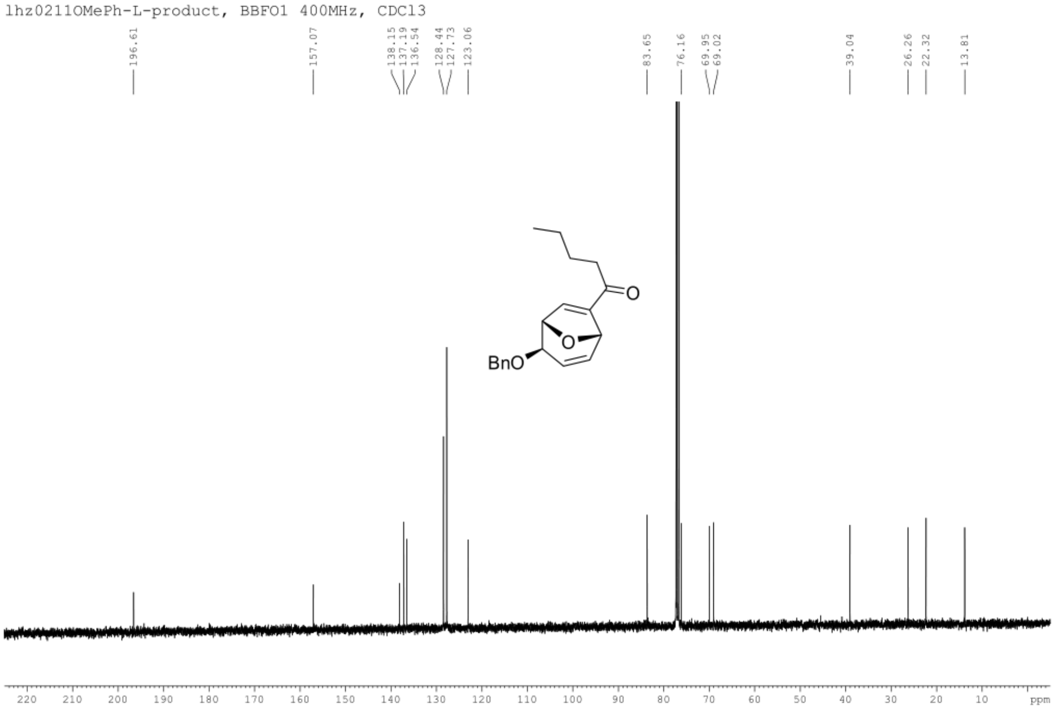

Supplementary Figure 58. ^{1}H and ^{13}C NMR spectrum for *ent*-2g.

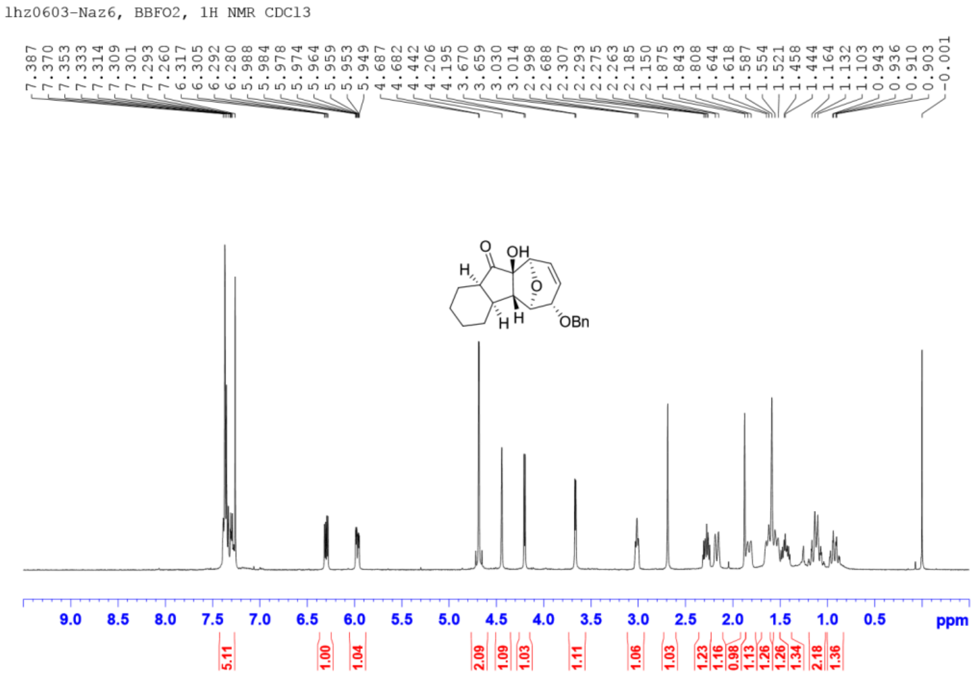

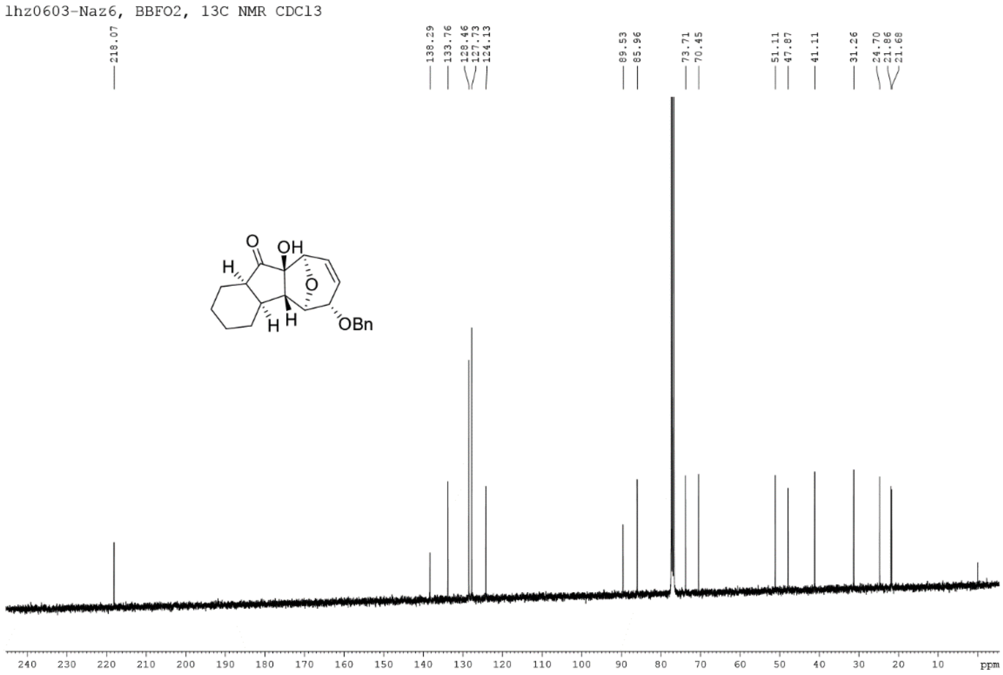

Supplementary Figure 59. ^{1}H and ^{13}C NMR spectrum for 3i.

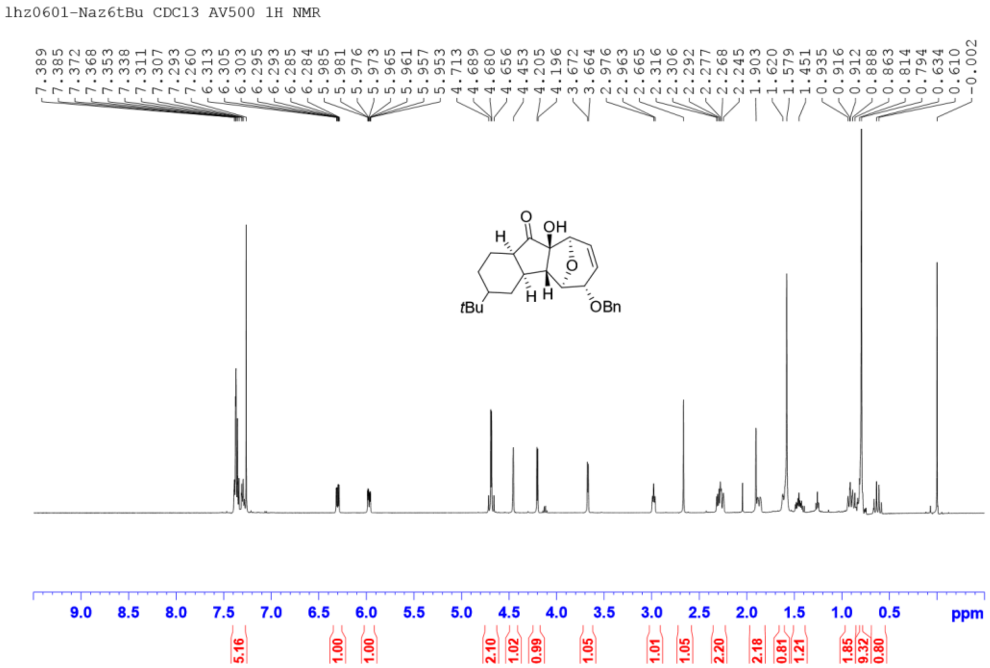

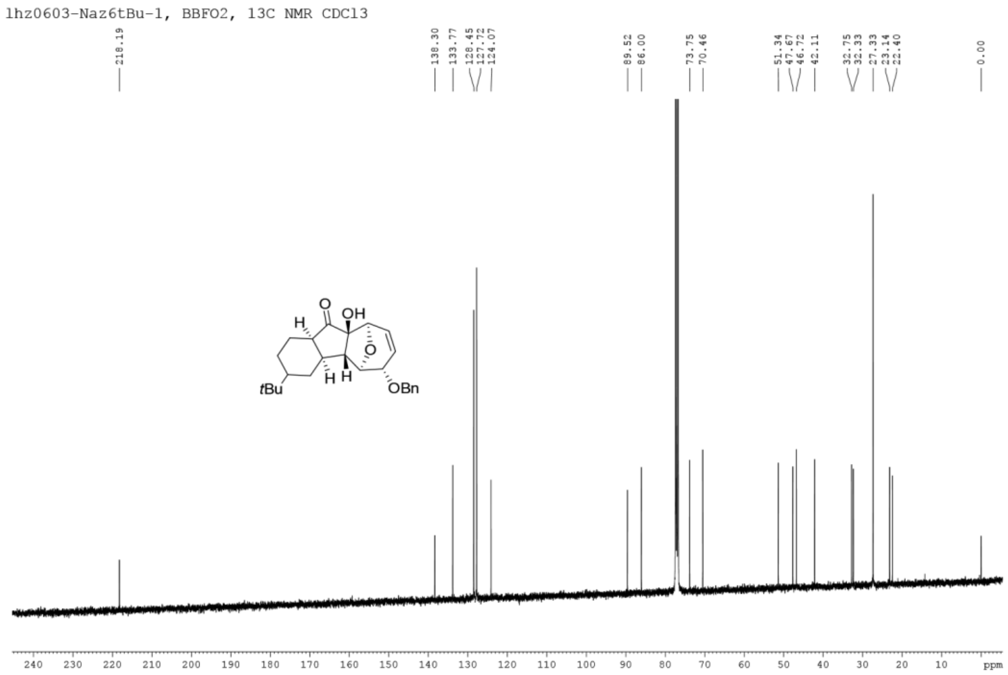

Supplementary Figure 60. ^{1}H and ^{13}C NMR spectrum for 3j.

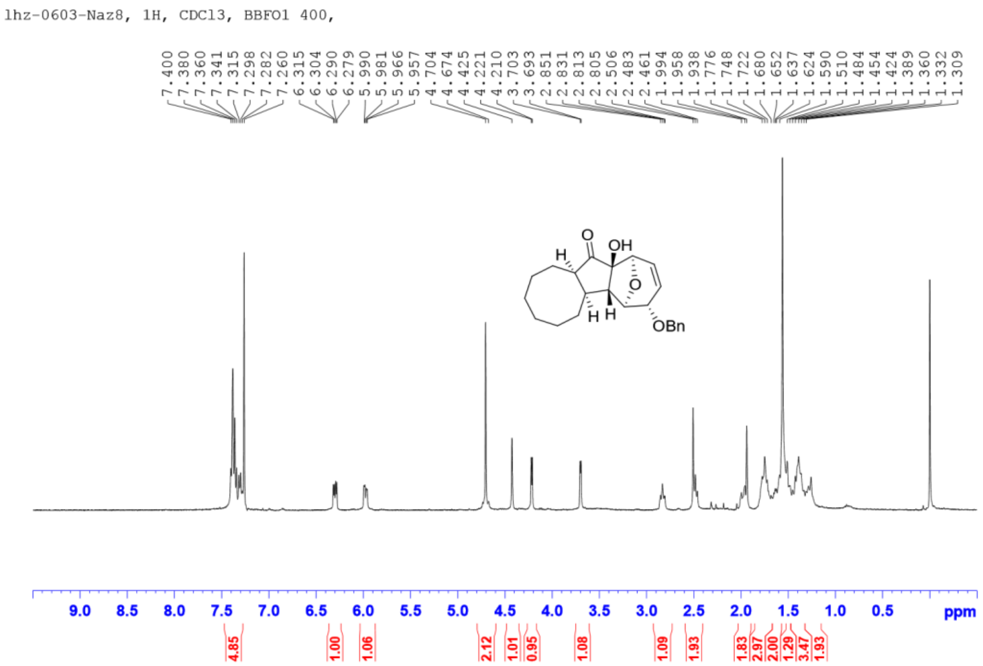

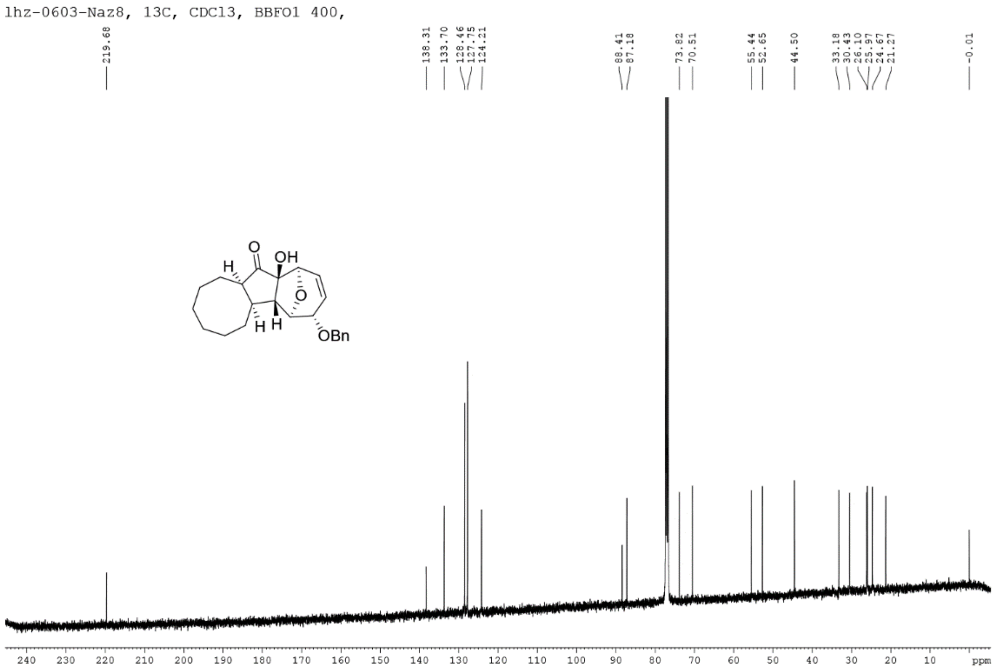

Supplementary Figure 61. ^{1}H and ^{13}C NMR spectrum for 3k.

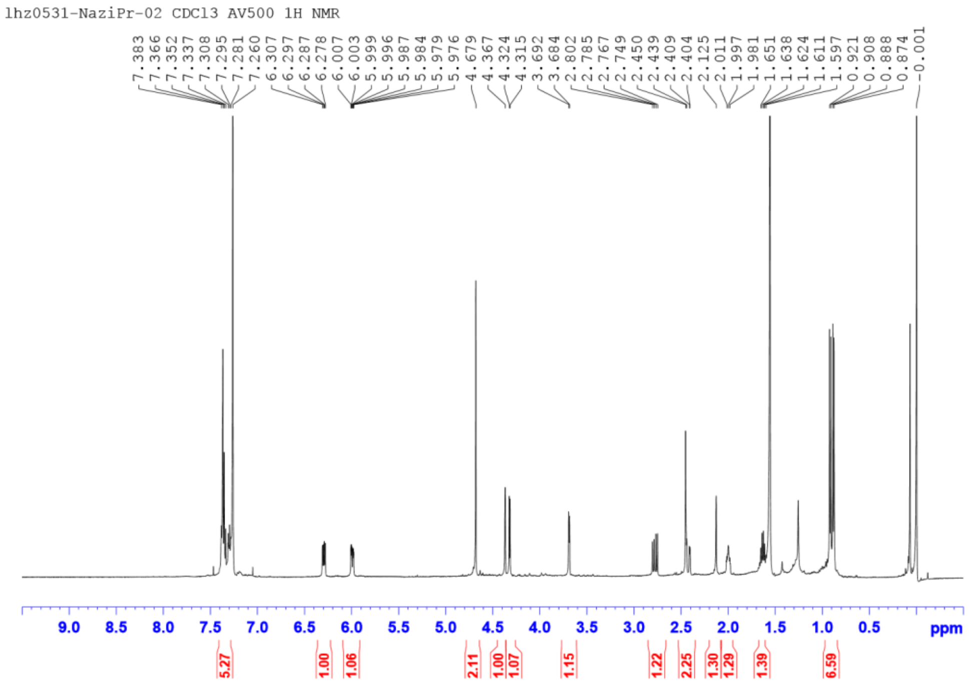

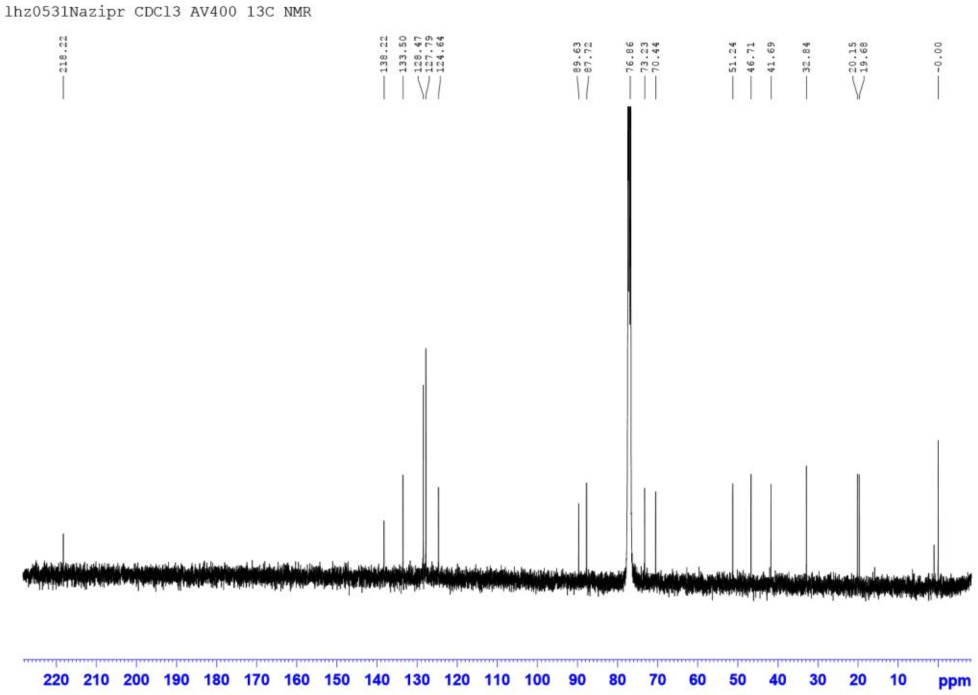

Supplementary Figure 62. ^{1}H and ^{13}C NMR spectrum for 3m.

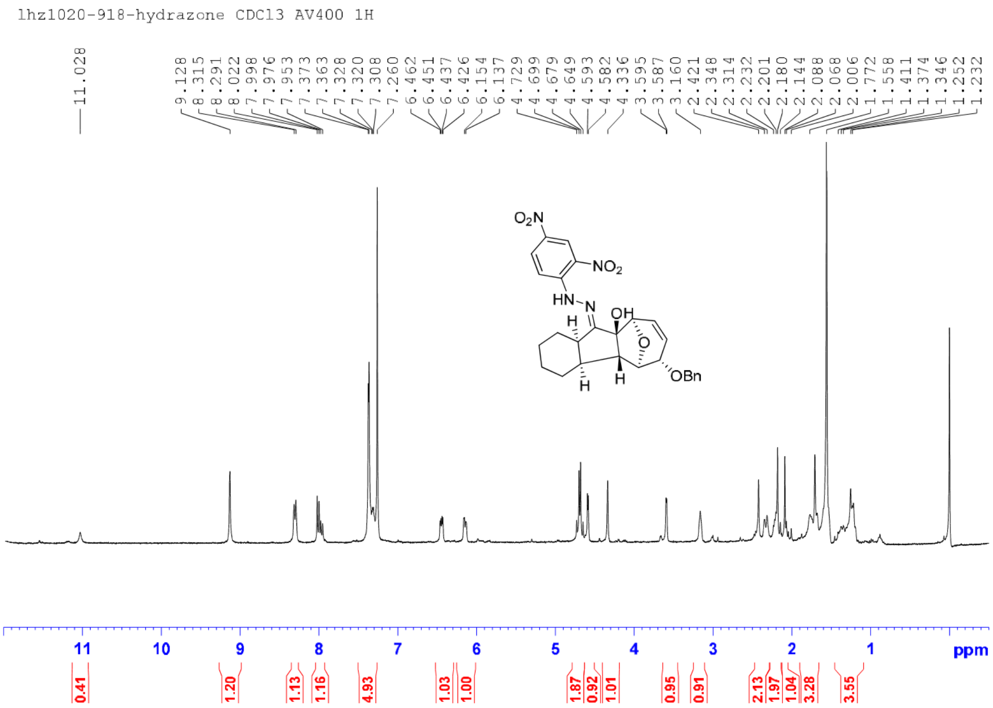

Supplementary Figure 63. **^{1}H spectrum for 9.**

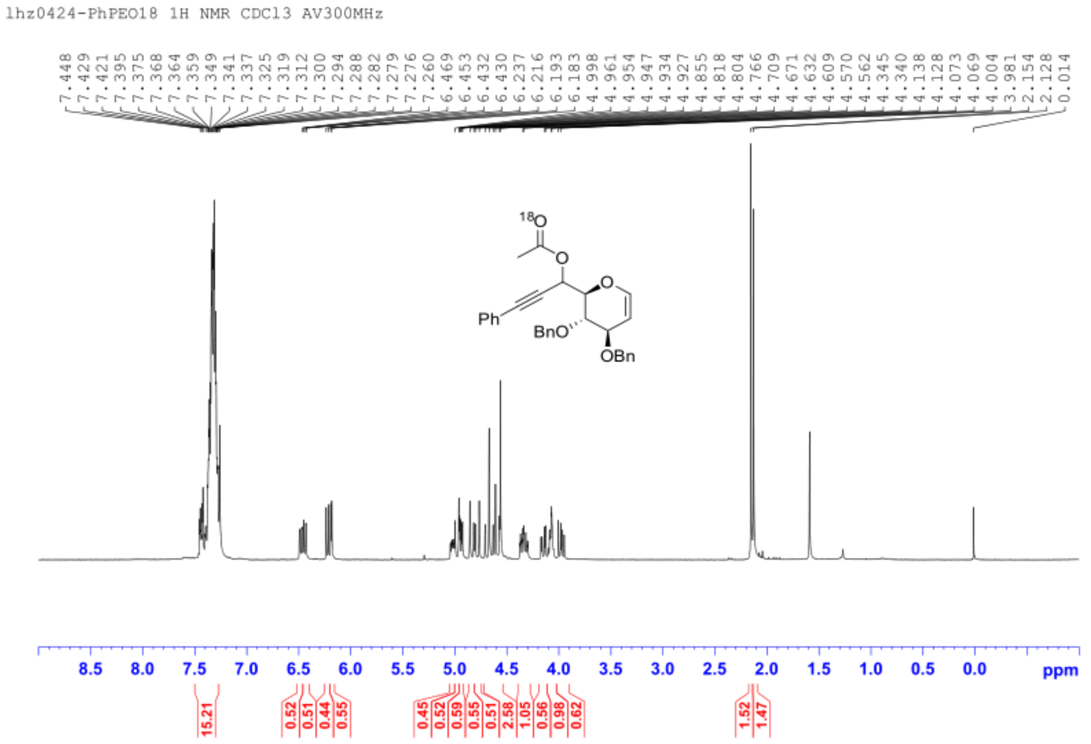

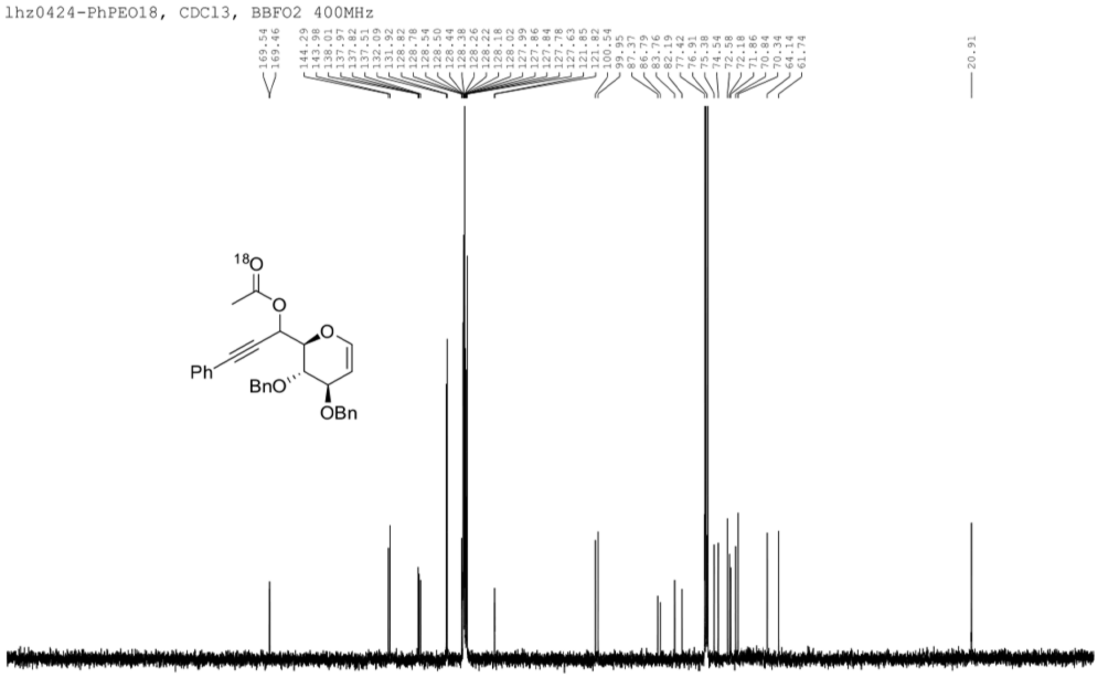

Supplementary Figure 64. ^{1}H and ^{13}C NMR spectrum for 1a-^{18}O.

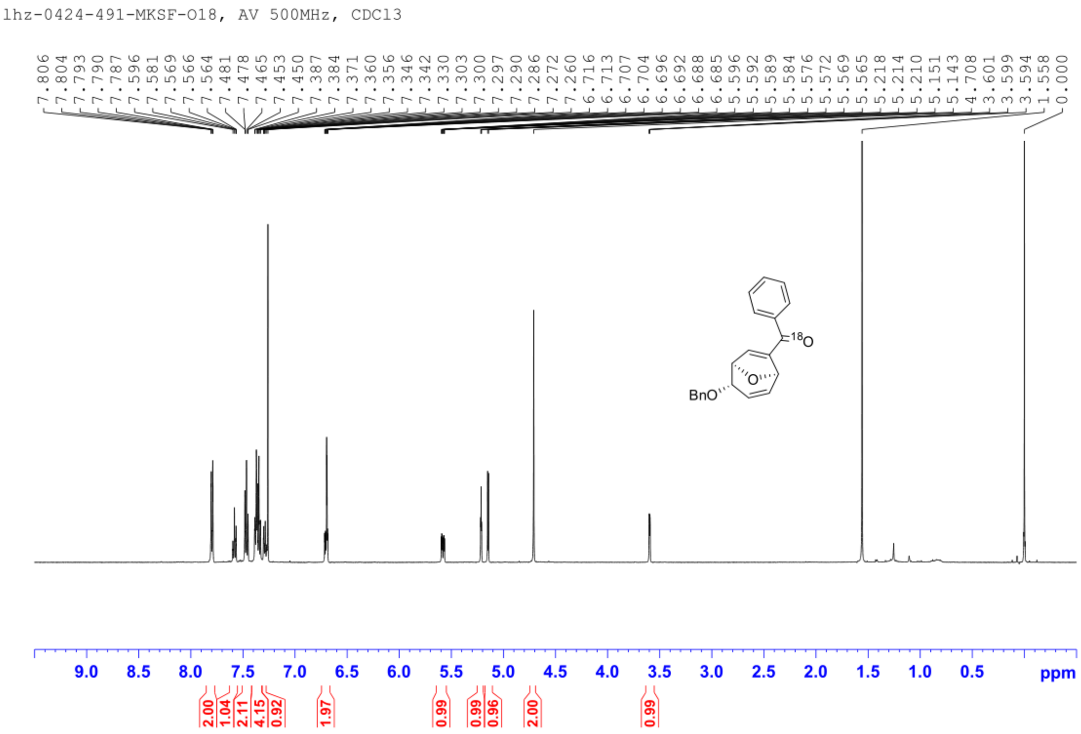

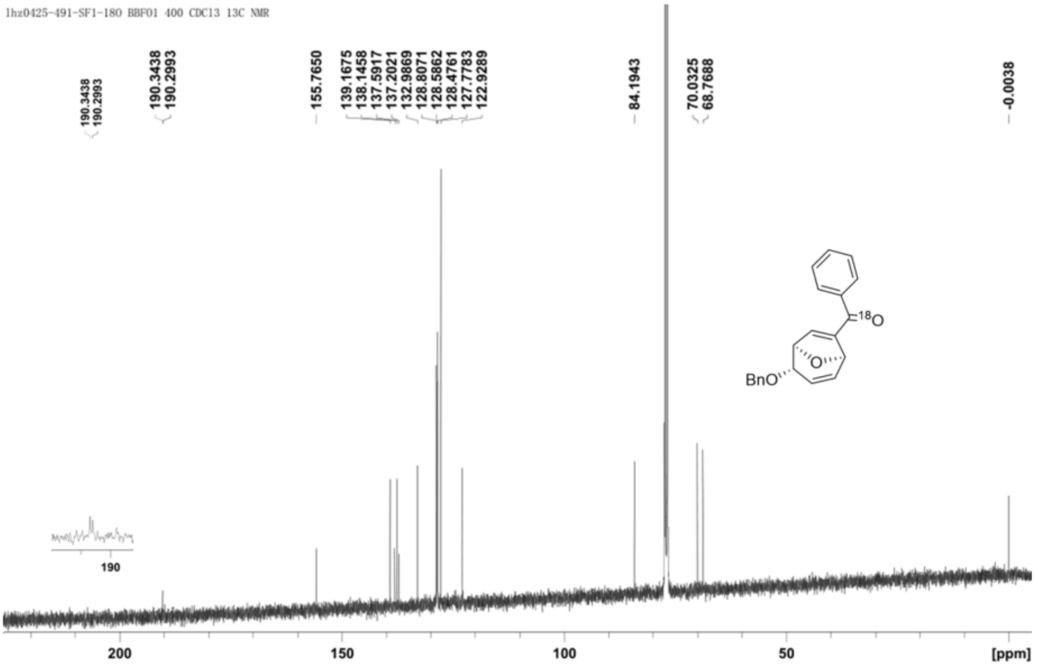

Supplementary Figure 65. ^{1}H and ^{13}C NMR spectrum for 2a-^{18}O.

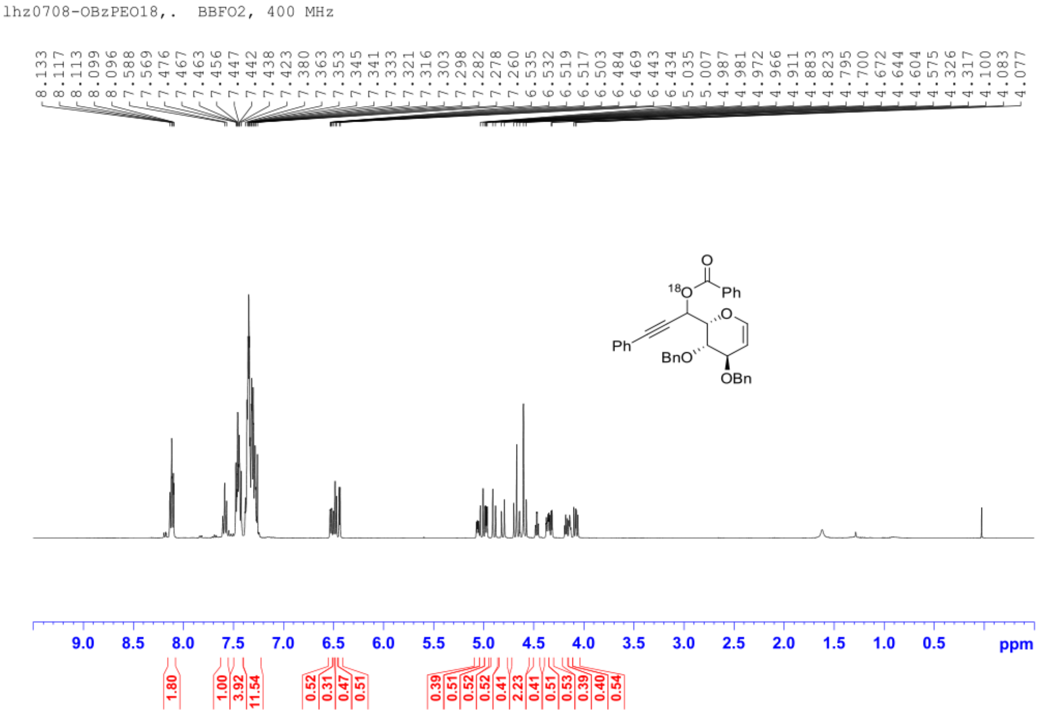

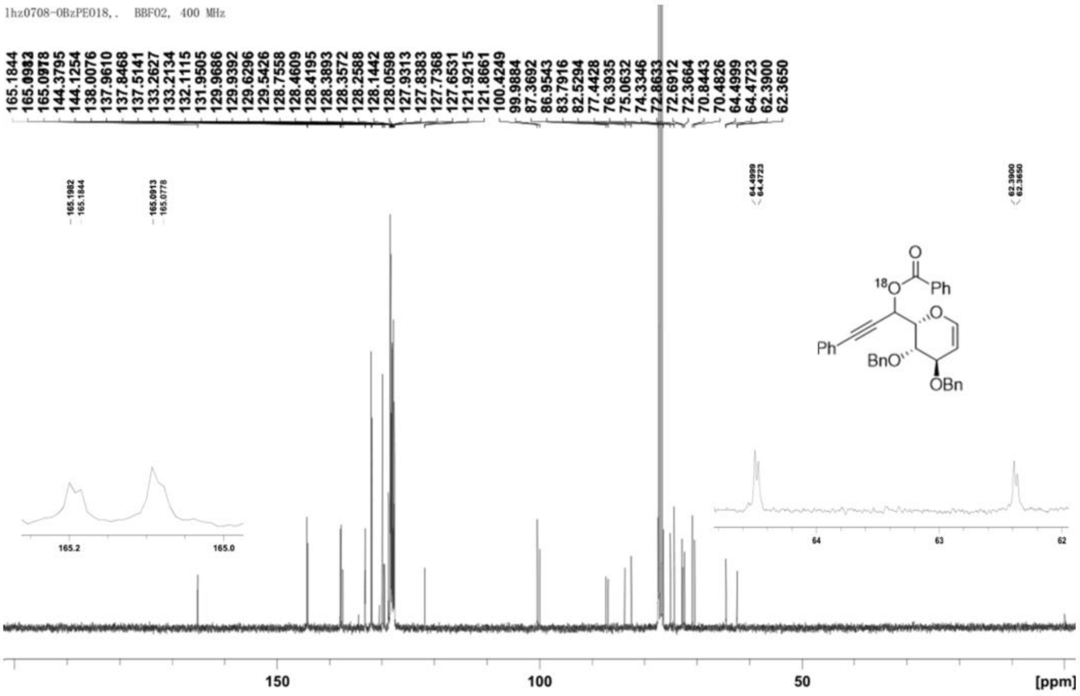

Supplementary Figure 66. ^{1}H and ^{13}C NMR spectrum for 1p-^{18}O.

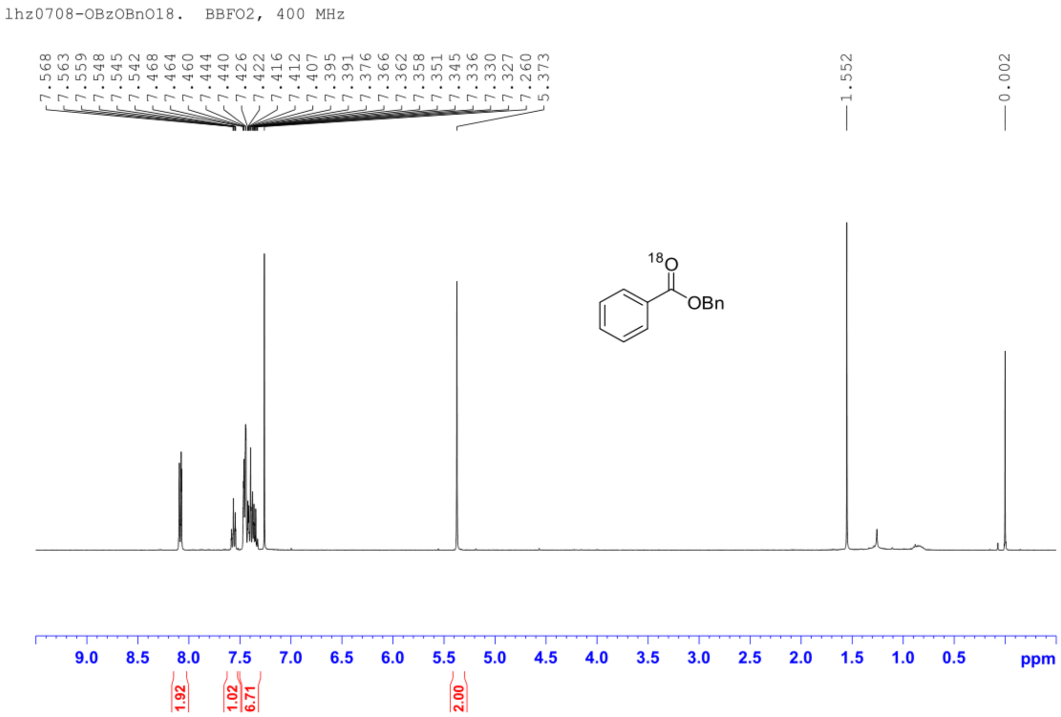

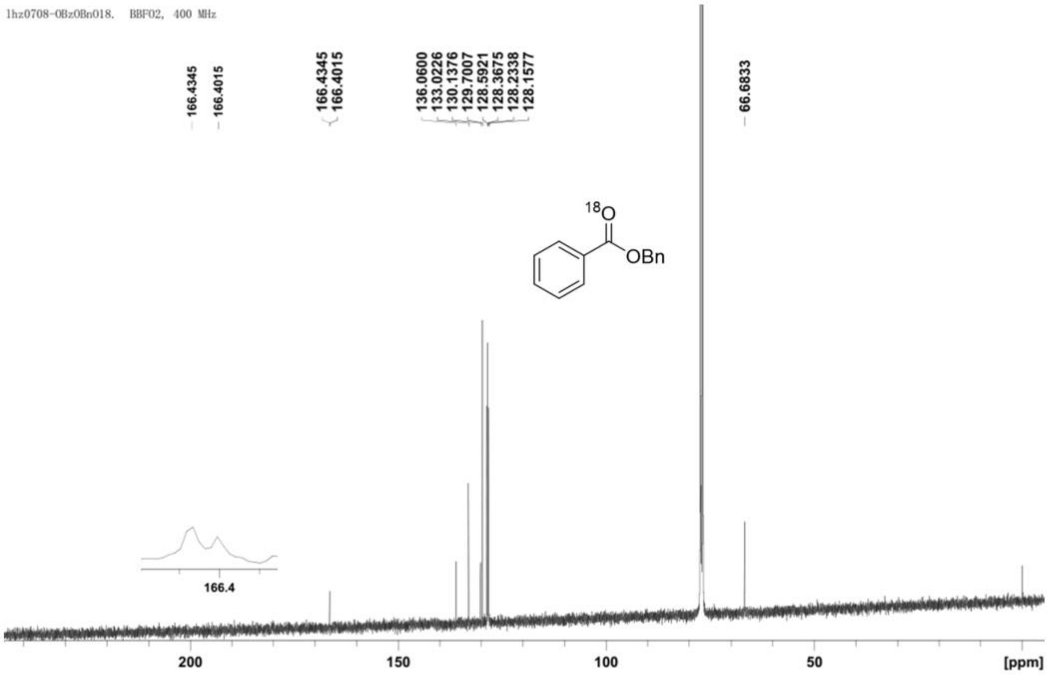

Supplementary Figure 67. ^{1}**H and** ^{13}**C NMR spectrum for 6-**^{18}**O.**
